# Diversification through gustatory courtship: an X-ray micro-computed tomography study on dwarf spiders

**DOI:** 10.1186/s12983-021-00435-8

**Published:** 2021-09-28

**Authors:** Shou-Wang Lin, Lara Lopardo, Gabriele Uhl

**Affiliations:** Zoological Institute and Museum, General and Systematic Zoology, University of Greifswald, Greifswald, Germany

**Keywords:** Nuptial feeding, Trait lability, Divergent evolution, Sexual selection, Micro-CT, Phylogeny, Araneae

## Abstract

**Background:**

Sexual selection has been considered to promote diversification and speciation. Sexually dimorphic species have been used to explore the supposed effect, however, with mixed results. In dwarf spiders (Erigoninae), many species are sexually dimorphic—males possess marked prosomal modifications. These male traits vary from moderate elevations to bizarre shapes in various prosomal regions. Previous studies established that male dwarf spiders produce substances in these prosomal modifications that are taken up by the females. These substances can act as nuptial gifts, which increase the mating probability of males and the oviposition rate in females. Therefore, these dimorphic traits have evolved in the context of sexual selection. Here, we explore the evolutionary lability of this gustatory trait complex with the aim of assessing the role of this trait complex in species divergence by investigating (1) if erigonine modified prosomata are inherently linked to nuptial-gift-producing glands, (2) if the evolution of the glands evolution preceded that of the modified prosomal shapes, and by assessing (3) the occurrence of convergent/divergent evolution and cryptic differentiation.

**Results:**

We reconstructed the position and extent of the glandular tissue along with the muscular anatomy in the anterior part of the prosoma of 76 erigonine spiders and three outgroup species using X-ray micro-computed tomography. In all but one case, modified prosomata are associated with gustatory glands. We incorporated the location of glands and muscles into an existing matrix of somatic and genitalic morphological traits of these taxa and reanalyzed their phylogenetic relationship. Our analysis supports that the possession of glandular equipment is the ancestral state and that the manifold modifications of the prosomal shape have evolved convergently multiple times. We found differences in gland position between species with both modified and unmodified prosomata, and reported on seven cases of gland loss.

**Conclusions:**

Our findings suggest that the occurrence of gustatory glands in sexually monomorphic ancestors has set the stage for the evolution of diverse dimorphic external modifications in dwarf spiders. Differences among congeners suggest that the gland position is highly susceptible to evolutionary changes. The multiple incidences might reflect costs of glandular tissue maintenance and nuptial feeding. Our results indicate divergent evolutionary patterns of gustatory-courtship-related traits, and thus a likely facilitating effect of sexual selection on speciation.

**Supplementary Information:**

The online version contains supplementary material available at 10.1186/s12983-021-00435-8.

## Background

The great diversity of secondary sexual traits in the animal world has been the primary inspiration for Darwin’s hypothesis of sexual selection [1, 2]. These dimorphic traits come in the form of coloration, ornamentation, behavior, size and shape [3]. Examples of sexually dimorphic male traits evolved under mate choice or intrasexual competition, such as the feather ornaments of peacocks [4], the enlarged mandibles of stag beetles [5],and the antlers of deer [6]. Differences between populations in their mate preferences and in secondary sexual traits can lead to reproductive isolation [7]. Therefore, sexual selection has long been regarded as a driving force behind speciation [1, 8–10]. Alternatively, sexual dimorphism may also have evolved under the influence of ecological selection mechanisms. These include niche divergence between the sexes [11], such as the larger posterior salivary glands in male octopod *Eledonella pygmaea* due to intersexual vertical habitat partitioning in the water column and resulting differences in feeding habits [12]; and reproductive role division [13], like the female gigantism in many orb-weaving spiders selected for increased fecundity [14, 15].

Sexually dimorphic morphology has evolved in different spider groups several times independently, e.g., in some Theridiidae species (“cobweb spiders”) [16, 17], a pholcid (“daddy long leg spiders”) [18] and very pronounced so in the Erigoninae, a large subfamily of linyphiid spiders [19–21]. In these species, the shape and anatomy of the front body part (prosoma) of the males differ from those of the females and are highly species-specific. Moreover, in the species investigated thus far, the sexually dimorphic male prosomata play a role in nuptial feeding: females contact the specific structures and take up male glandular secretions during the mating sequence. Nuptial feeding during mating has been observed in spiders [19–22] as well as in insects [23]. In many cases, the secretions entice females to copulate and prolong copulation duration, which can increase sperm transfer [23]. There is ample evidence that these traits are involved in male-male competition, are subject to female choice, and might even represent sensory traps [24]. Therefore, it is likely that the evolution of these gustatory sexually dimorphic traits has been driven by sexual selection.

In erigonine spiders, the most speciose subfamily of Linyphiidae, which is in turn, the second-most diverse spider family [25], several morphological and behavioral studies on sexually dimorphic prosomal structures have been undertaken. In contrast to other linyphiid subfamilies, erigonines exhibit striking variations in male prosomata between and within taxa, including grooves, lobes, humps, turrets, as well as lateral sulci and pits on the prosomata [26]. Prosomal modifications are only found in adult males [27]. At least 223 among the 402 erigonine genera exhibit some degree of prosomal shape modifications, and the degree of variability differs among genera [28, 29]. The modifications can occur anteriorly or posteriorly to the eye region of the prosoma, and are often associated with pores and modified setae [26, 30, 31]. In all species examined, the modified prosomal regions contain extensive secretory epidermal glandular tissues, with only one known exception [32–36]. Further, the cellular composition of the glandular units may vary even within a genus [36].

In all erigonines studied to date, the females contact the male prosomal structures with their mouthparts during courtship and mating and ingest the secretion [19–21, 37, 38]. The secretions released from the glandular tissue function as male mating effort through gustatory courtship, and were also shown to increase brood size [21]. Although these secretions were suspected to produce volatile substances for species recognition or female mate choice [39, 40], behavioral studies have found no indication of such pheromonal function [20, 41]. Since male prosomal structures are highly variable among species not only in position and shape but also in the degree of elaboration and secretory cell types, these male traits and the female preferences are most likely under direct selection. Since there has been no indication of ecological functions of these dimorphic male traits, the diversification is likely the result of sexual selection that is known to promote speciation [7]. Consequently, erigonine spiders are an ideal group for studying the evolution of sexually dimorphic traits and lend themselves to assessing the link between sexual selection and speciation.

Gustatory glandular tissues have also been found in erigonine species that lack pronounced prosomal modifications [32, 36]. It has thus been hypothesized that the glands may have evolved first in sexually monomorphic ancestors, followed by the independent evolution of various external modifications in different lineages [35, 40]. Indeed, recent phylogenetic studies imply parallel evolution of similar external prosomal shapes not only among erigonine genera [26, 42, 43], but also within genera [29]. However, these studies did not examine whether glandular tissues are associated with the respective prosomal structures. Consequently, the relationship between glands and prosomal shape remains to be explored, i.e., whether species without external prosomal modifications are equipped with glandular tissues, whether there are species with prosomal modifications that lack glandular tissues and whether externally similar prosomal shapes are similar in glandular equipment. Assessing the diversity of occurrence and location of glandular tissue and prosomal shape modifications within and between genera will elucidate the probability of convergence and evolvability of this trait complex.

X-ray micro-computed tomography (micro-CT) offers a non-destructive option for scrutinizing and visualizing internal morphological features and organ systems such as musculature, digestive system, nervous system, and glandular tissues [44–53]. Micro-CT has been applied to determine the location of the nuptial-gift-producing organ in a fly [54] as well as the prosomal glands in three erigonine spiders [28]. We use micro-CT to examine the presence/absence and the distribution of epidermal glands in the species included in [29]. The revision and phylogenetic analysis of [29] focused on the erigonine genus *Oedothorax* and its closely related taxa, mainly *Callitrichia* and *Mitrager*. By investigating the internal anatomy of the prosoma, we aim at elucidating the lability of this trait complex and the evolutionary patterns of both glands and prosomal structures. Instead of plotting the glandular features on the existing tree topology, we scored them as new characters and incorporated them into the character matrix, because these characters may also contain phylogenetic information. Since cheliceral and pharyngeal muscles also connect to the prosoma cuticle [28, 55–57], epidermal glands and muscle attachment are mutually exclusive. The cheliceral muscles control the movement of the chelicerae used for prey capture, grasping, chewing, digging burrows, carrying egg cases, and during courtship [58]. The pharyngeal muscles together with the sucking stomach serve to inject saliva and extract fluid from the prey [59]. There is a potential conflict between feeding and nuptial gift production caused by the limited cuticular surface space for muscle attachments and epidermal glands. We therefore also investigated the course and attachment location of these muscles.

For determining the appearances of male-specific glandular tissues in contrast to other types of tissues in the scans, we compared the scans of female and male *Oedothorax gibbosus*, and applied the derived criteria to the identification of tissue types in other species. We also recorded cuticular structural details revealed by the scans. We assessed the variation in the glandular and muscular anatomy in species with diverse prosomal shapes, in order to address four major questions. 1) Are modified prosomata inherently linked to glands? 2) Did glands evolve before prosomal shape modifications? 3) Did similar external prosomal shapes evolve convergently and 4) are there cryptic differences in internal gland distributions among externally similar species? If prosomal structures as well as the distribution of gustatory glands show divergent evolutionary patterns between and within lineages, and similar prosomal structures evolved convergently in different lineages, we consider this strong support for a diversifying effect of sexual selection in erigonines.

## Methods

### Studied taxa

Among the 79 species included in the study of [29] 77 species were micro-CT-scanned for one male prosoma, except *Oedothorax gibbosus* and *Gongylidiellum latebricola*. In *Oedothorax gibbosus*, two male morphs occur, one with strongly modified prosomal shape (*gibbosus* morph) and one without (*tuberosus* morph) [60]; consequently one male of each morph was scanned. *Gongylidiellum vivum* was scanned instead of *G. latebricola* due to the poor preservation condition of the latter. For *Mitrager noordami* and *Oedothorax gibbosus*, the prosomata of both sexes were scanned to demonstrate the difference between the unmodified female and the modified male prosomata. Voucher information of the investigated specimens is provided in Additional File 1: Table S1.

### Sample preparation, micro-CT scanning and image processing

Samples were dehydrated through a graded ethanol series (70, 80, 90, 95, 99% ethanol). To enhance tissue contrast, specimens were transferred to a 1% iodine solution (iodine, resublimated [Carl Roth GmbH & Co. KG, Karlsruhe, Germany; cat. #X864.1] in 99.8% ethanol) for 48 h [51]. Samples were washed in 99% ethanol twice, in an interval of 24-h and were subsequently mounted inside modified plastic pipette tips [28]. Micro-CT scans were performed using an optical laboratory-scale X-ray microscope (Zeiss XradiaXCT-200). Scans were performed with a 20 × objective lens unit using the following settings: 30 kV voltage/8 W power and an exposure time of 3 s. These settings resulted in scan times of about 2 h and a pixel size between 1 and 1.5 μm. Tomography projections were reconstructed using XMReconstructor (Carl Zeiss Microscopy CmbH, Jena, Germany), resulting in image stacks (TIFF format). All scans were performed using Binning 2 (Camera Binning) for noise reduction and subsequently reconstructed with full resolution (using Binning 1). Since the microCT resolution did not allow for a cellular level identification of the tissue, we compared semi-thin histological sections of *O. gibbosus* males (gland present in both *gibbosus* and *tuberosus* morphs) [36] and females (gland supposed to be absent) to the representation of the tissue on the virtual sections. The decision as to the presence or absence of epidermal glands in the studied species was based on this comparative assessment.

To provide showcase examples of the internal prosomal structures, the following organ systems were digitally labeled in AMIRA 6.0.1 (Visualization Science Group, FEI) for one *Oedothorax gibbosus*-morph male, one *tuberosus*-morph male, and one female: nervous system, muscles, digestive system as well as male-specific epidermal glandular tissues, and an unknown tissue found in different areas in the prosoma. For all examined species (except *Walckenaeria acuminata* due to low resolution caused by tissue shrinkage), the following structures were labeled: dorsal part of prosoma, chelicerae (at least the proximal part), supposed gustatory glandular tissues, and all muscles connecting the dorsal part of the prosoma with the chelicerae and the pharynx. We use the English terms for the muscle as done in [57]. Abbreviations used in the text or figures are given in Table 1. For visualization, the labeled structures were converted to a surface mesh by Fiji [61]. These files were subsequently imported into MeVisLab (*MeVis Medical Solutions AG and Fraunhofer MEVIS*) using the “Scientific3DFigurePDFApp” module, reduced, colored, and exported as.u3D files, which were subsequently inserted into the additional files in the.pdf format (Adobe Acrobat Pro).

**Table 1:** Abbreviations and/or coloration of morphological structures follow mostly Wood and Parkinson (2019)

Structure and abbreviation in present paper	Color in figures
Gustatory glandular tissue	Purple
Anterior median eyes (AME)	
Anterior medial inner muscle (AMI)	Dark Blue
Anterior medial muscle (AM)	Dark purple
Anterior medial outer muscle (AMO)	Light blue
Anterior outer muscle (AO)	Red
Anterior pharyngeal dilator muscle (DA)	Light orange
Inter-cheliceral-sclerite muscle (IC)	Aqua
Lateral anterior muscle (LA)	Yellow
Lateral posterior muscle (LP)	Magenta
Posterior median eyes (PME)	-
Posterior medial muscle (PM)	Green
Posterior pharyngeal dilator muscle (DP)	Dark orange

### Phylogenetic analysis and reconstruction of character state transformations

Parsimony analyses were conducted with TNT Version 1.1 [62] using a traditional search with random seed 1, 500 replications, 1000 trees saved per replication, branch swapping by TBR algorithm. Continuous characters were treated as ordered and analyzed as such [63]. For equal weight analysis, two clade support measures, Bremer support (tree suboptimal by 17 steps during TBR retained from existing trees) and Jackknife support (removal probability = 36%), were also calculated using TNT. For implied-weighting analyses, the constants of concavity *k* were set for 1–6, 10, 15, 20, 30, 100, 1000 for relatively high to relatively low cost of homoplasy [64]. Character optimization and generation of tree images were carried out using Winclada version 1.00.08 [65].

Our character matrix (Additional File 2) is based on Matrix II of [29] (79 taxa, 128 discrete and four continuous morphological characters). Seven new discrete characters were added based on findings from the micro-CT reconstruction of the internal structures (see below for description), resulting in a matrix with 135 discrete and four continuous characters: Ch. 130. gustatory epidermal gland: 0, absent; 1, present; Ch. 131. gustatory epidermal gland at before-eye region: 0, absent; 1, present; Ch. 132. gustatory epidermal gland at eye region: 0, absent; 1, present; Ch. 133. gustatory epidermal gland surrounded by the pharynx muscle: 0, absent; 1, present; Ch. 134. gustatory epidermal gland posterior to the pharynx muscle: 0, absent; 1, present; and 135. gland in the chelicerae: 0, absent; 1, present; Ch. 129. pre-posterior-median-eye (PME) groove muscle attachment (applicable only when the pre-PME groove is present): 0, no muscle attached to the groove; 1, inter-cheliceral-sclerite muscle attached to the groove; 2, inter-cheliceral-sclerite muscle and anterior pharyngeal dilator muscle attached to the groove. After comparative re-examination of specimens, the previous homology interpretation of some male palpal features in two species could not be corroborated and therefore the character scoring was changed to “unknown”. The newly defined characters (Additional File 1: Table S2), other changes in the character matrix, and the observation on the cheliceral and pharyngeal muscles that differed from the previous description [57] are reported in the Additional File 1.

**Table 2:** Summarized results of the implied weights analyses using different *k* values

*k*	Best score	No. of trees	No. of hits	Tree length	Clade 26	Clade 50	Clade 64	No. common clades with equal weight tree
1	60.52497	1	40	533	P	P	P	33
2	49.89028	1	41	526	P	M − *c*	P	35
3	42.88728	1	46	526	P	M − *c*	P	35
4	37.85770	1	50	521	P	M − *c*	M + *n*	38
5	33.96389	1	12	520	P	M − *c*	P	40
6	30.86829	1	3	520	P	M − *c*	P	40
10	22.80454	1	13	515	M	M	P	50
15	17.30398	1	2	511	M + *m*	M	P	47
20	13.96072	1	1	509	M	M	P	53
30	10.08356	1	5	504	M + *m*	M	M + *n*	55
100	3.43621	1	3	503	M	M + *m*	M + *n*	63
1000	0.36323	1	2	503	M	M	M	77

Tree lengths were calculated only by discrete characters with weight = 1

*c*: *Callitrichia convector*; M: monophyletic *m*: *Oedothorax meghalaya incertae sedis*; *n*: *Oedothorax nazareti incertae sedis*; P: polyphyletic

The micro-CT scans and reconstructions led to one character redefinition and revealed two scoring mistakes in one species in matrix II in [29]. Character 91 (i.e., absence/presence of post-PME groove) was redefined as “post-posterior-pharyngeal-dilator-muscle (-DP) groove”: i.e., the post-PME groove is located posteriorly to the posterior pharyngeal dilator muscle attachment. This redefinition rendered the scoring of this character as absent in *Emertongone montifera*, as the groove is located anteriorly to the posterior pharyngeal dilator attachment; and as present in *Mitrager noordami*. Corrections of scoring mistakes for *Mitrager globiceps* comprise character 80 (inter-anterior-median-eye (AME) -PME strong setal group) as absent instead of present; and character 89 (post-/inter-PME strong setal group bending forward) also as absent instead of present.

## Results

### Determining male-specific glandular tissues in the scans

**Fig. 1:**
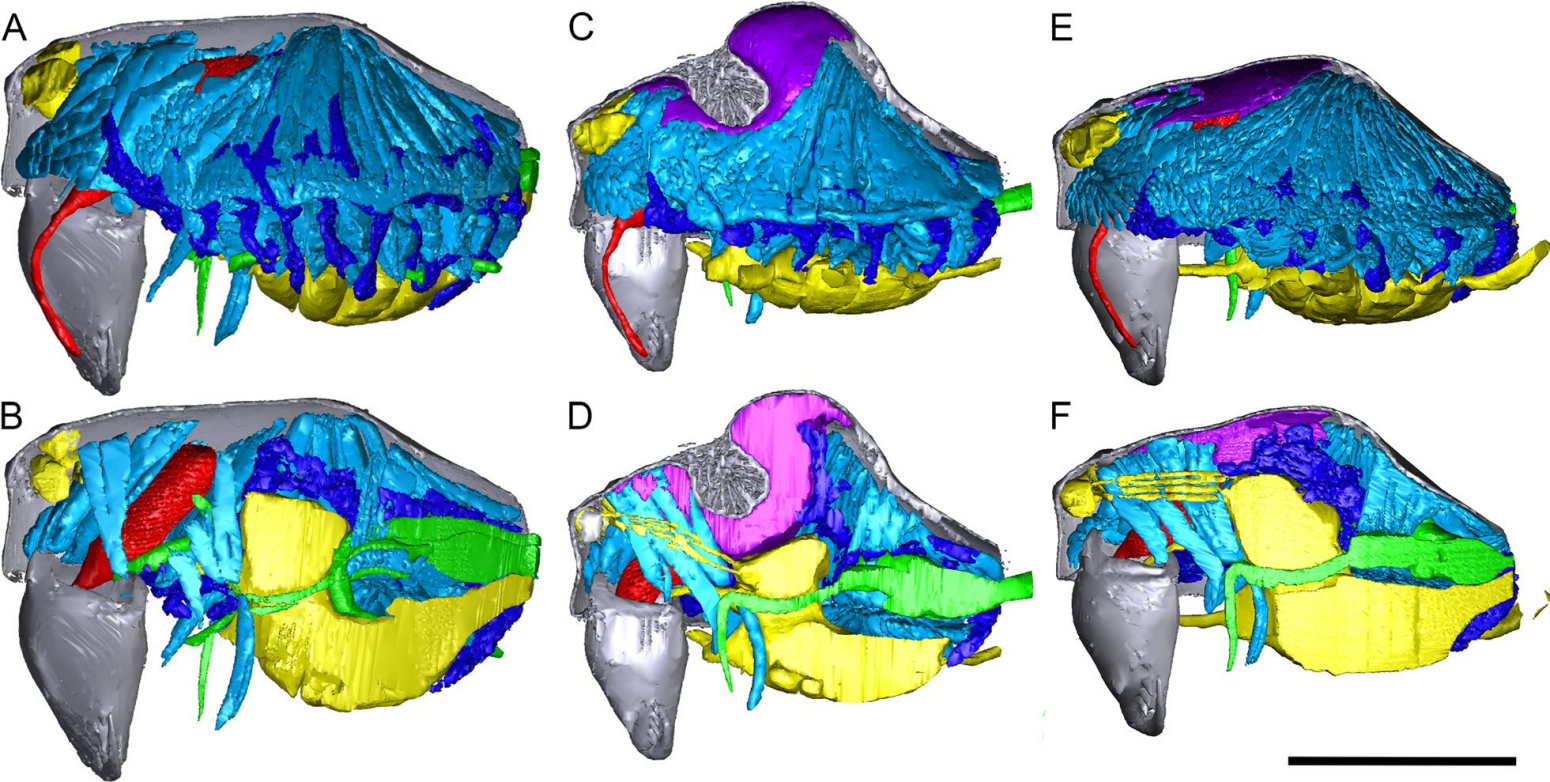
Images of micro-CT scans of *Oedothorax gibbosus*, with central nervous system (yellow), venom glands (red), muscles (light blue), digestive system (green), unknown amorphous tissues (dark blue) and male specific epidermal glands (purple). Interactive 3D images are available in the Additional File 3. Click on the image to activate individual 3D model; to hide/show different structures, right-click and select “show model tree”. **a**, **c**, **e** show the structures on both sides, while **b, d, f** show only the right side. **a, b** female. **c, d** male, *gibbosus* morph. **e, f** male, *tuberosus* morph

**Fig. 2:**
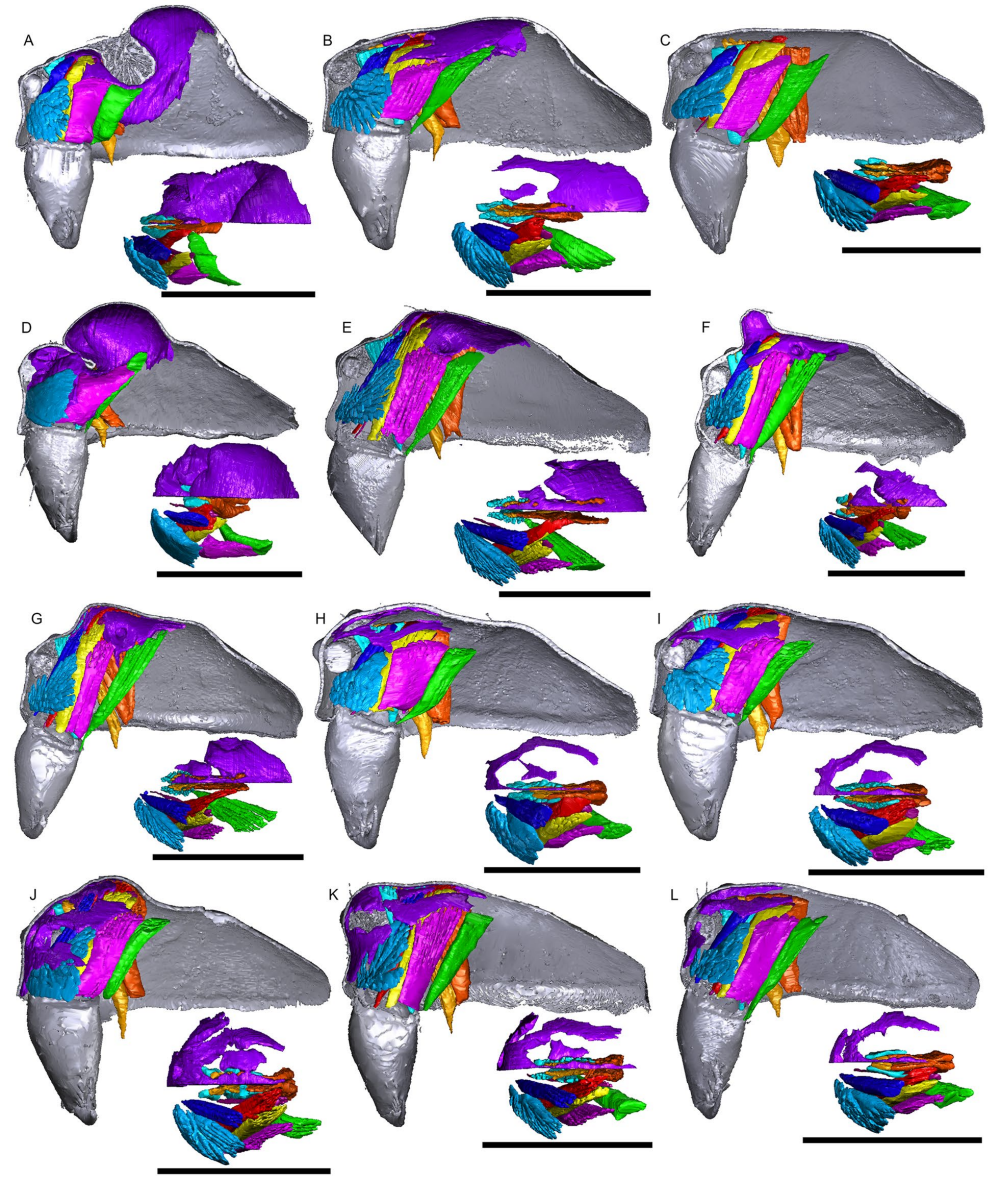
Images of micro-CT scans with gustatory glandular tissues (purple), different sets of cheliceral muscles (left side), pharyngeal dilators (both sides). The right side of the prosomal cuticle digitally is segmented and color-coded following Table 1. Interactive 3D images are available in the Additional File 4. Click on the image to activate individual 3D model; to hide/show different structures, right-click and select “show model tree”. **a** *Oedothorax gibbosus*, *gibbosus* morph. **b** *O. gibbosus*, *tuberosus* morph. **c** *O. gibbosus*, female. **d** *O. trilobatus*. **e** *O. gibbifer*. **f** *O. apicatus*. **g** *O. retusus*. **h** *O. paludigena*. **i** *O. agrestis*. **j** *O. meridionalis*. **k** *O. fuscus*. **l** *O. tingitanus*. Scale bars 0.5 mm

**Fig. 3:**
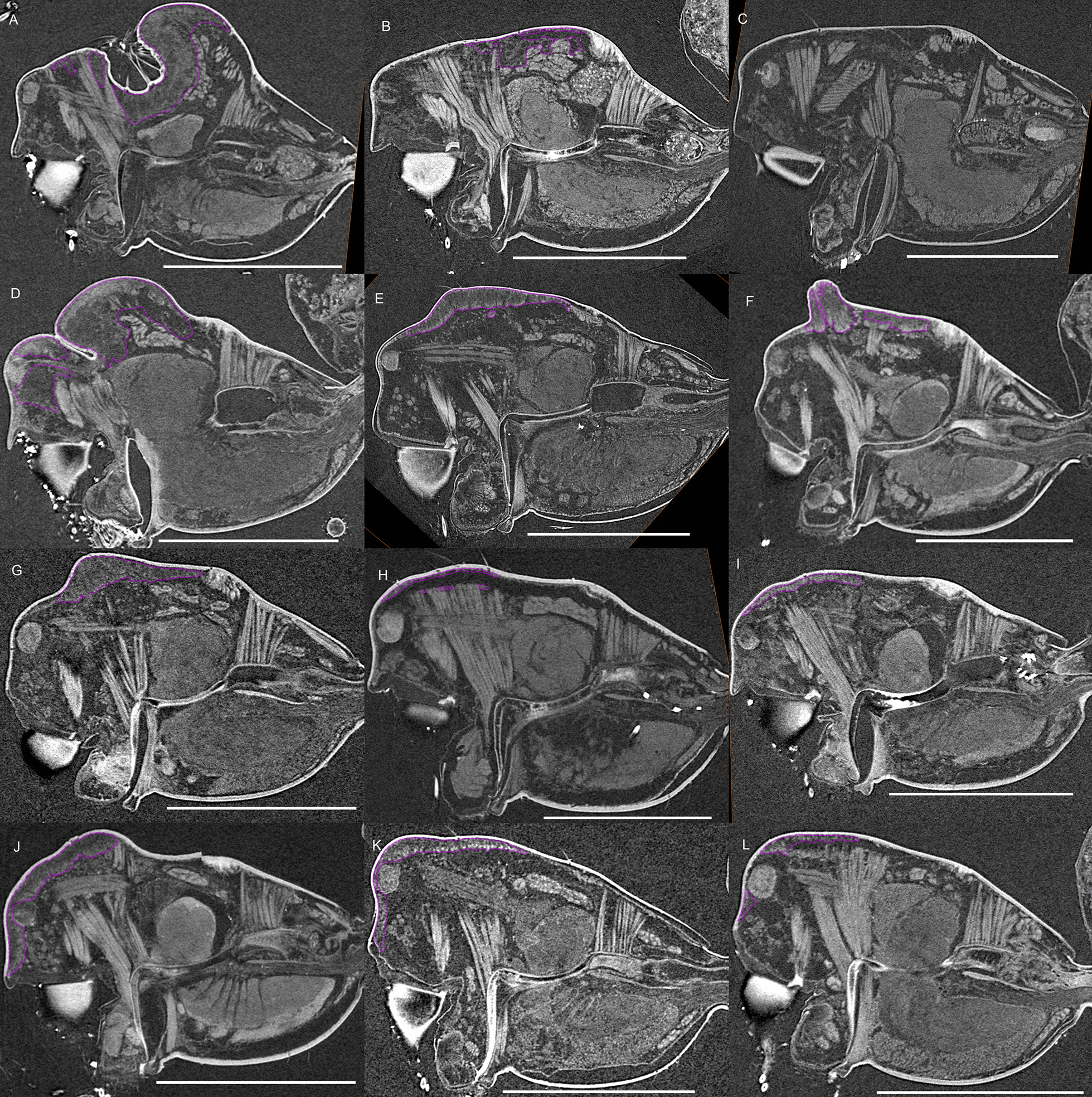
Virtual slices of micro-CT scans on the sagittal plane, with gustatory glandular tissues outlined in purple. **a** *Oedothorax gibbosus*, *gibbosus* morph. **b** *O. gibbosus*, *tuberosus* morph. **c** *O. gibbosus*, female. **d** *O. trilobatus*. **e** *O. gibbifer*. **f** *O. apicatus*. **g** *O. retusus*. **h** *O. paludigena*. **i** *O. agrestis*. **j** *O. meridionalis*. **k** *O. fuscus*. **l** *O. tingitanus*. Scale bars 0.5 mm

**Fig. 4:**
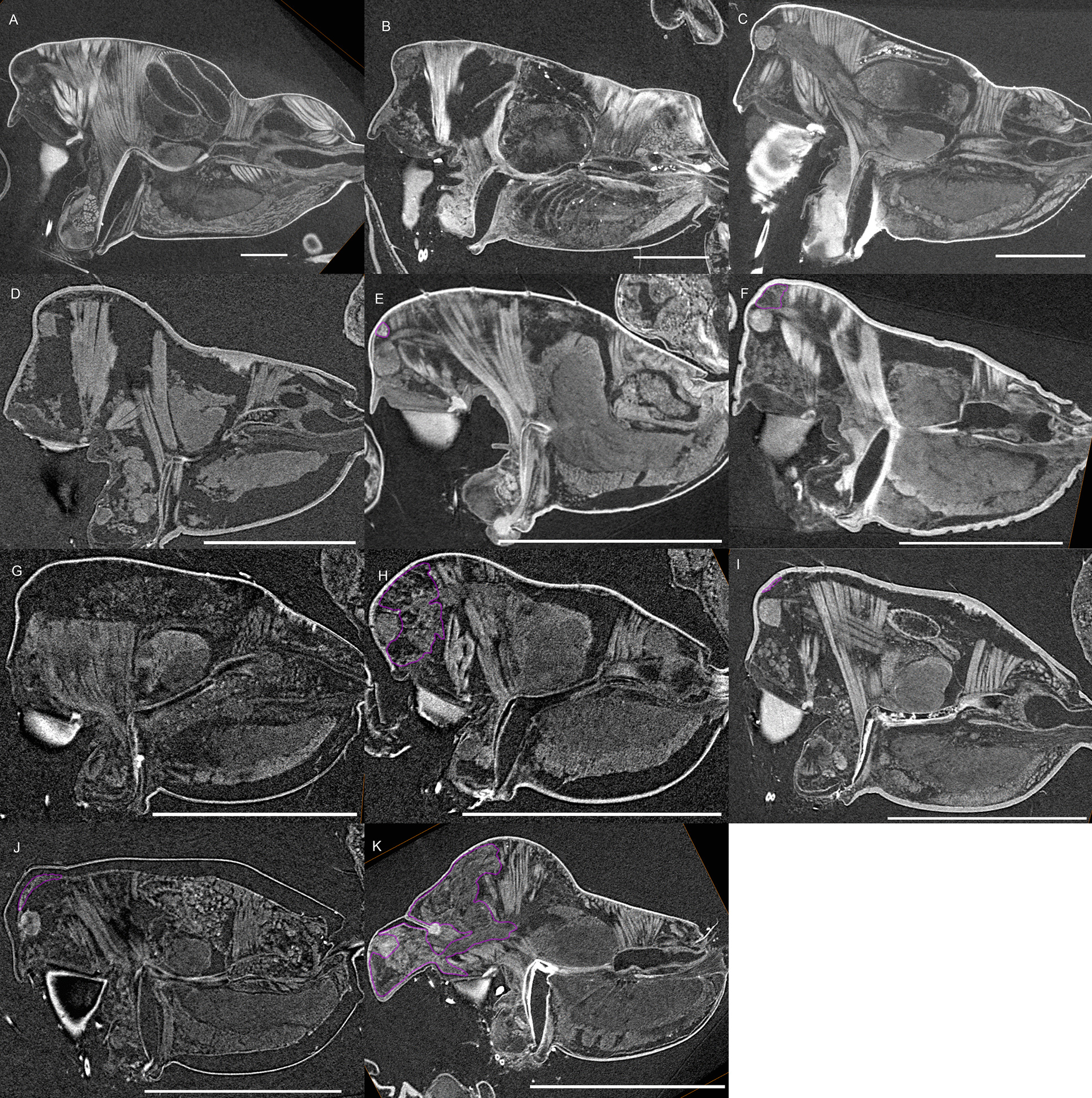
Virtual slices of micro-CT scans on the sagittal plane, with gustatory glandular tissues outlined in purple. **a** *Pimoa autioculata*. **b** *Stemonyphantes lineatus*. **c** *Linyphia triangularis*. **d** *Erigone atra*. **e** *Gongylidiellum vivum*. **f** *Lophomma punctatum*. **g** *Diplocentria bidentata*. **h** *Araeoncus humilis*. **i** *Jilinus hulongensis*. **j** *Cornitibia simplicithorax*. **k** *Emertongone montifera*. Scale bars 0.5 mm

**Fig. 5:**
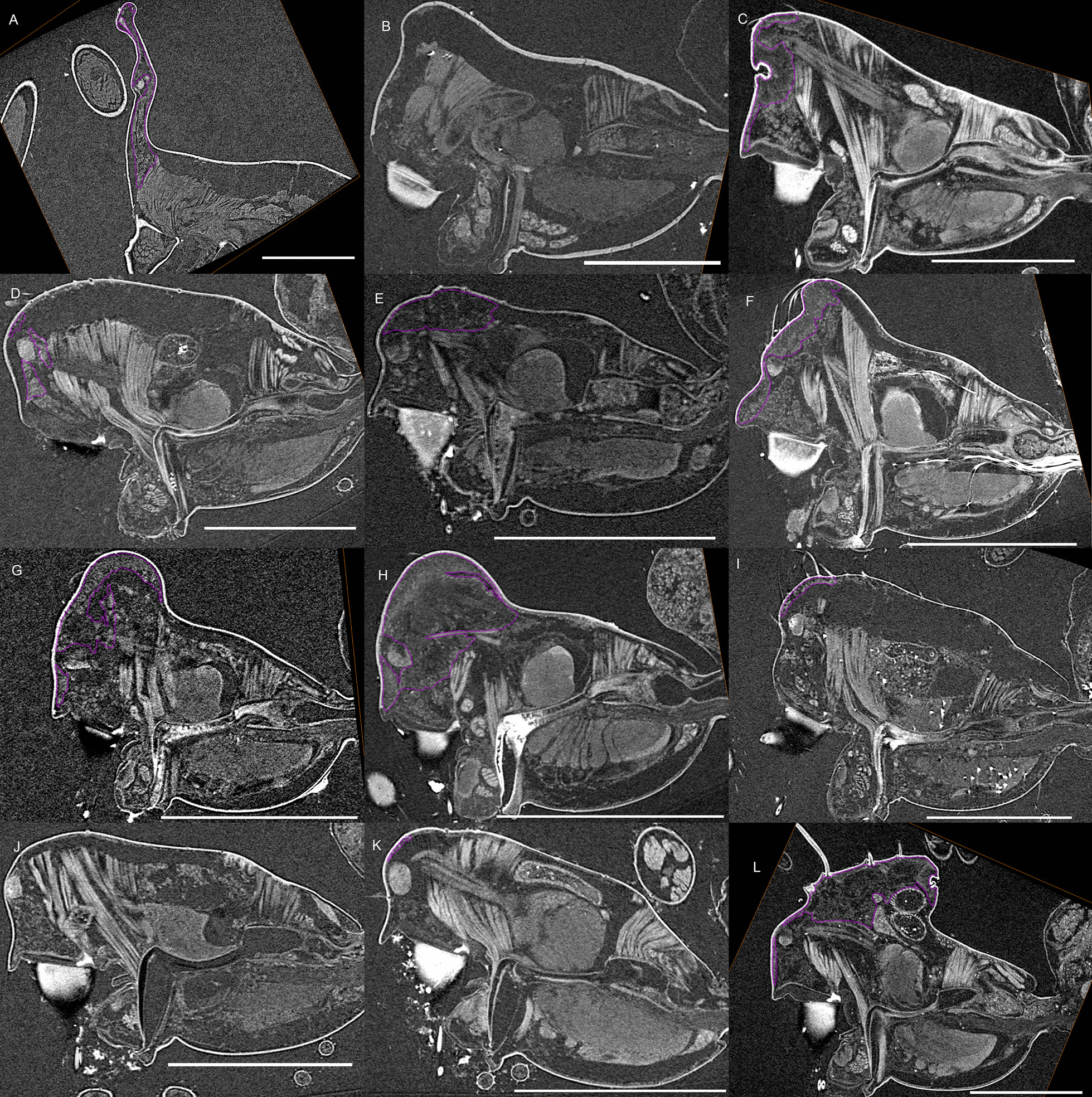
Virtual slices of micro-CT scans on the sagittal plane, with gustatory glandular tissues outlined in purple. **a** *Walckenaeria acuminata*. **b** *Gonatium rubellum*. **c** *Shaanxinus mingchihensis*. **d** *Oedothorax kodaikanal incertae sedis*. **e** *O. paracymbialis incertae sedis*. **f** *O. meghalaya incertae sedis*. **g** *Atypena cirrifrons*. **h** *A. formosana*. **i** *O. uncus incertae sedis*. **j** *O. cunur incertae sedis*. **k** *O. stylus incertae sedis*. **l** *Nasoona setifera*. Scale bars 0.5 mm

**Fig. 6:**
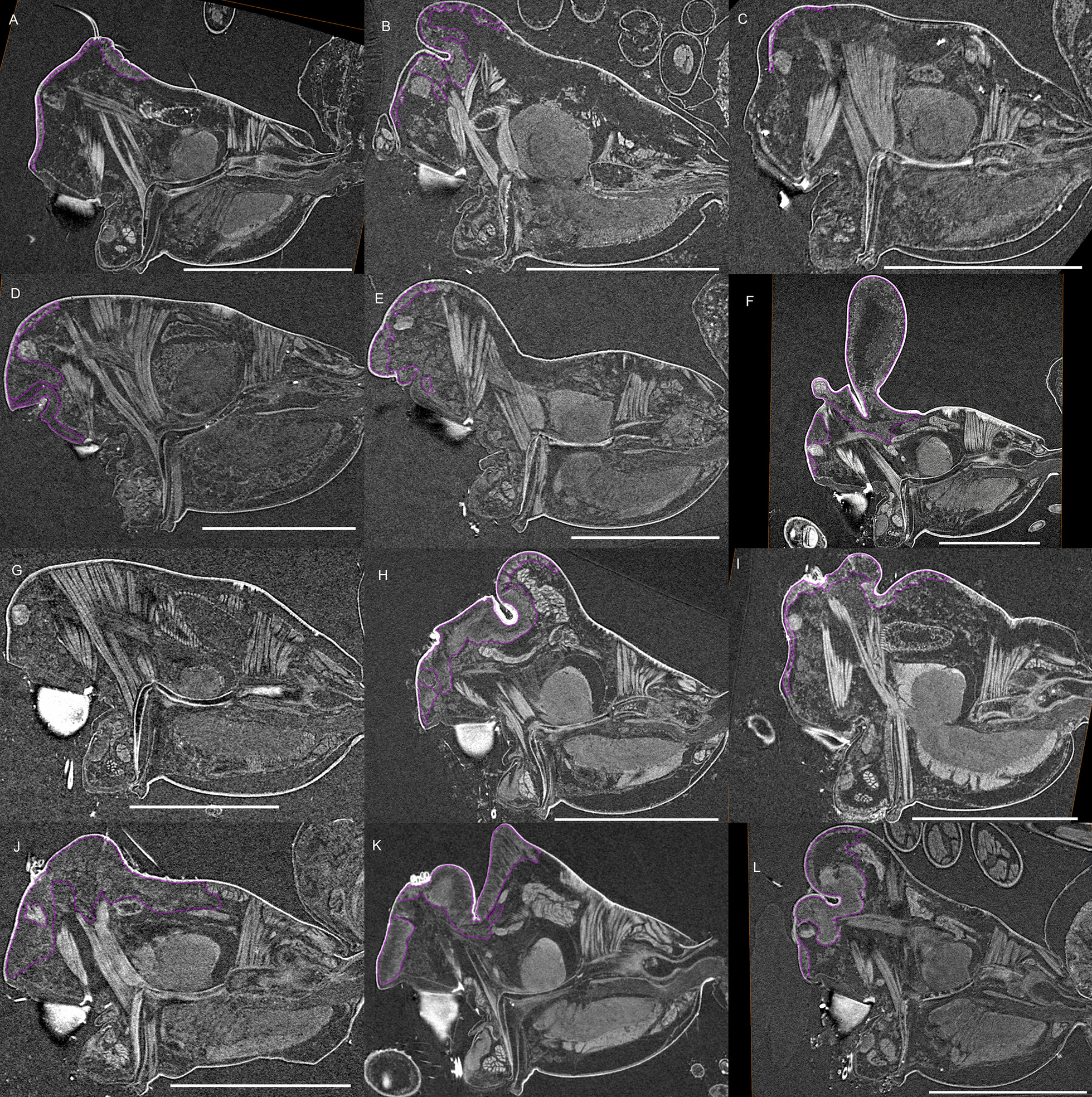
Virtual slices of micro-CT scans on the sagittal plane, with gustatory glandular tissues outlined in purple. **a** *Nasoona crucifera*. **b** *Mitrager globiceps*. **c** *M. hirsuta*. **d** *M. clypeellum*. **e** *M. elongata*. **f** *M. noordami*, male. **g** *M. noordami*, female, **h** *M. cornuta*. **i** *M. villosa*. **j** *M. angela*. **k** *M. coronata*. **l** *M. sexoculorum*. Scale bars 0.5 mm

**Fig. 7:**
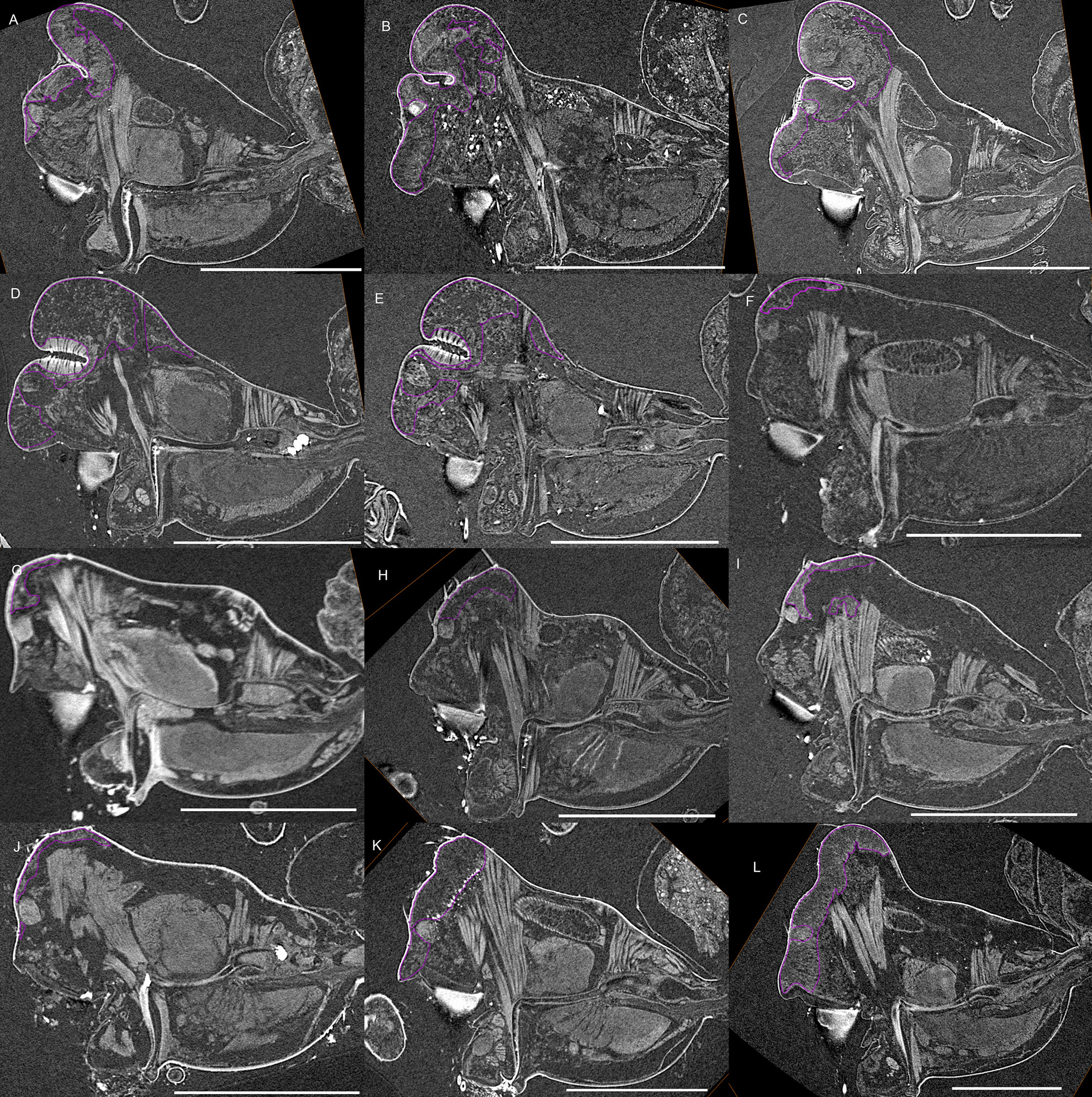
Virtual slices of micro-CT scans on the sagittal plane, with gustatory glandular tissues outlined in purple. **a** *Mitrager lineata*. **b** *M. dismodicoides*. **c** *M. tholusa*. **d** *M. lucida*. **e** *M. sexoculata*. **f** *M. unicolor*. **g** *M. rustica*. **h** *M. assueta*. **i** *M. malearmata*. **j** *M. lopchu*. **k** *M. falciferoides*. **l** *M. falcifer*. Scale bars 0.5 mm

**Fig. 8:**
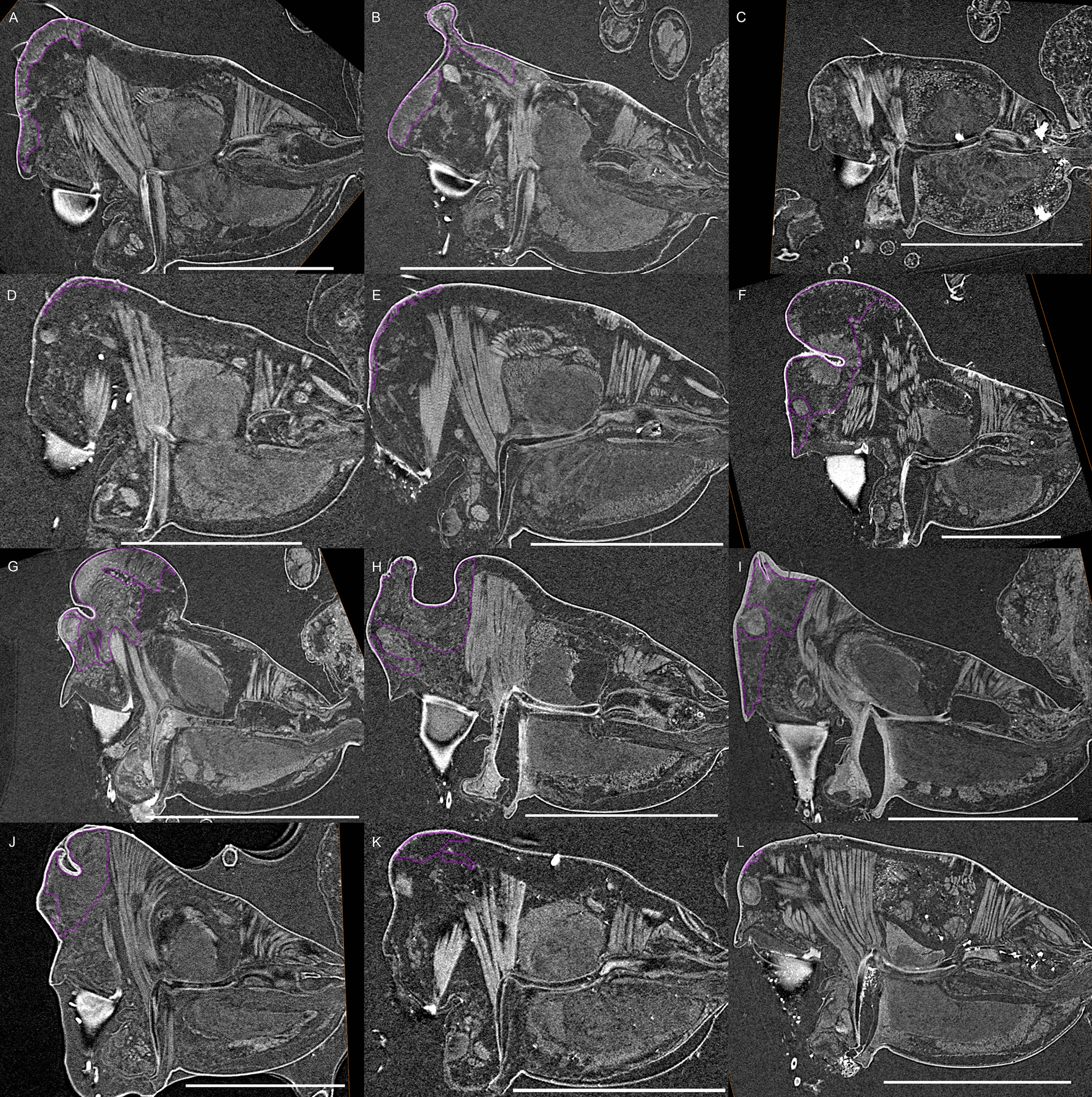
Virtual slices of micro-CT scans on the sagittal plane, with gustatory glandular tissues outlined in purple. **a** *Mitrager modesta* (Tanasevitch, 1998). **b** *M. savigniformis* (Tanasevitch, 1998). **c** *Holmelgonia basalis*. **d** *Callitrichia holmi*. **e** *Ca. picta*. **f** *Ca. gloriosa*. **g** *Ca. convector*. **h** *Ca. sellafrontis*. **i** *Ca. juguma*. **j** *Ca. uncata*. **k** *Ca. pilosa*. **l** *Ca. muscicola*. Scale bars 0.5 mm

**Fig. 9:**
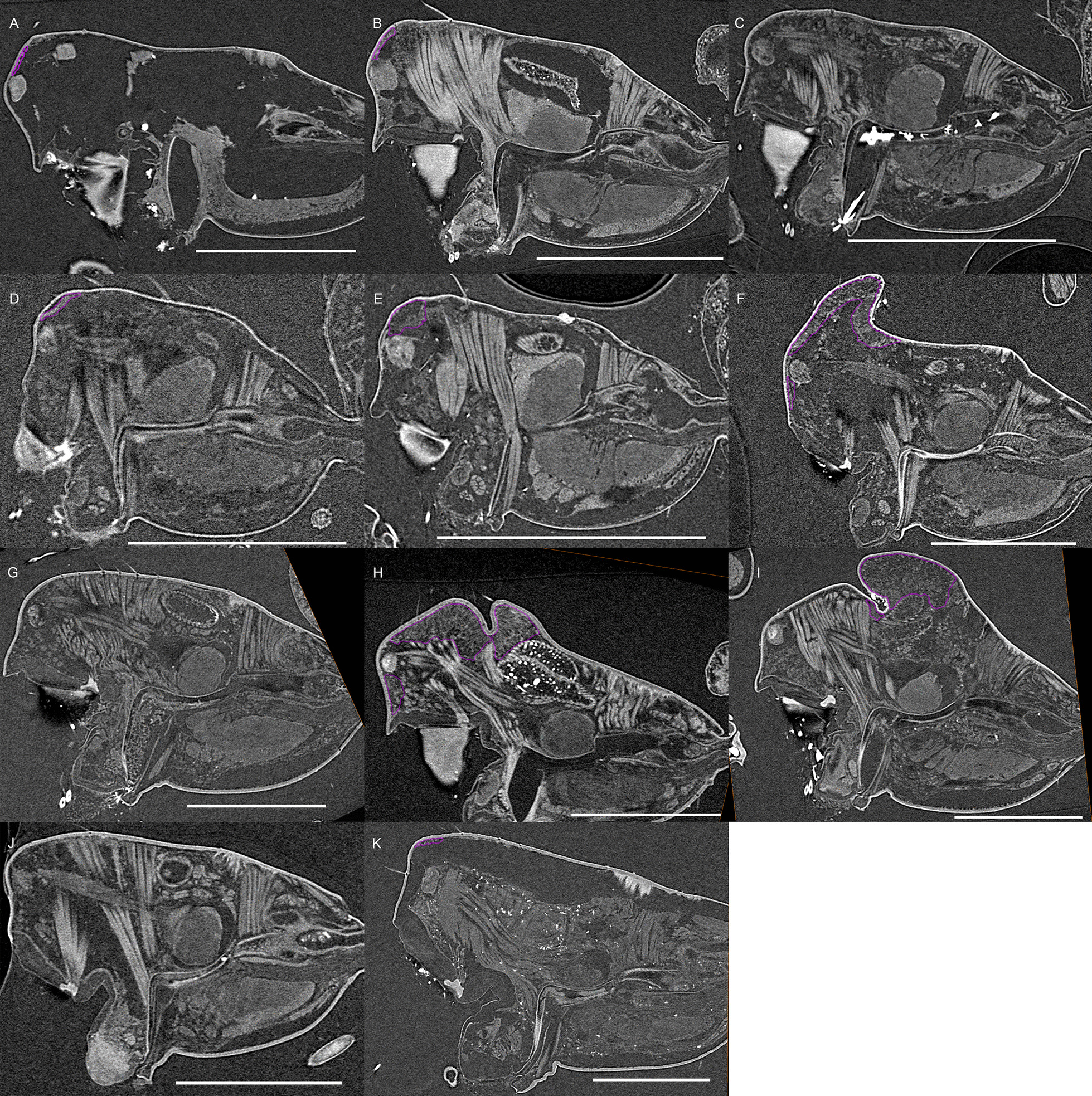
Virtual slices of micro-CT scans on the sagittal plane, with gustatory glandular tissues outlined in purple. **a**. *Callitrichia latitibialis*. **b** *Ca. longiducta*. **c** *Ca. usitata*. **d** *Ca. legrandi*. **e** *Ca. macropthalma*. **f** *Oedothorax nazareti incertae sedis*. **g** *Gongylidium rufipes*. **h** *Ummeliata insecticeps*. **i** *U. esyunini*. **j** *Hylyphantes graminicola*. **k** *Tmeticus tolli*. Scale bars 0.5 mm

**Fig. 10:**
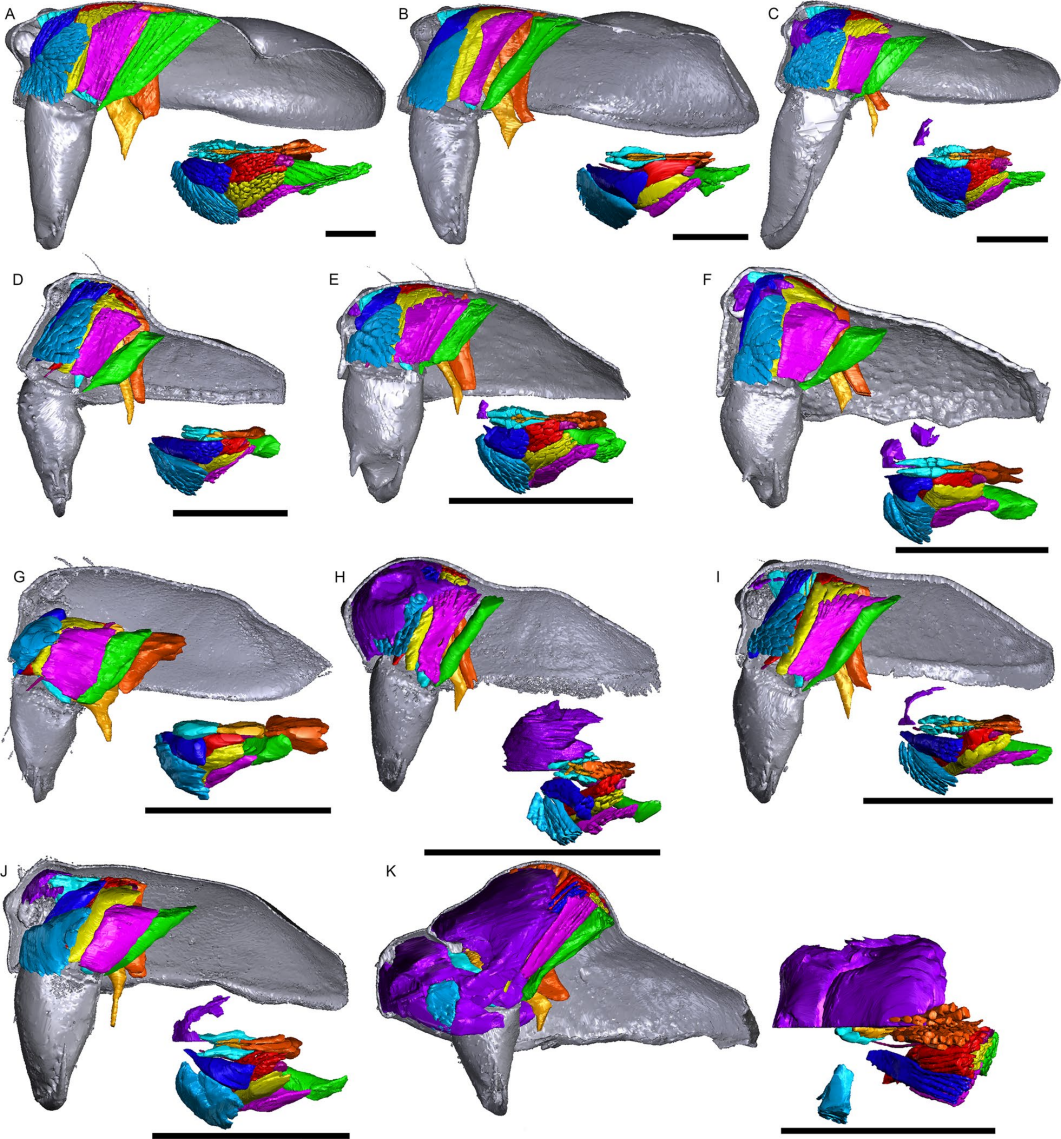
Images of micro-CT scans with gustatory glandular tissues (purple), different sets of cheliceral muscles (left side), pharyngeal dilators (both sides). The right side of the prosomal cuticle is digitally segmented and color-coded following Table 1. Interactive 3D images are available in the Additional File 5. Click on the image to activate individual 3D model; to hide/show different structures, right-click and select “show model tree”. **a** *Pimoa autioculata*. **b** *Stemonyphantes lineatus*. **c** *Linyphia triangularis*. **d** *Erigone atra*. **e** *Gongylidiellum vivum*. **f** *Lophomma punctatum*. **g** *Diplocentria bidentata*. **h** *Araeoncus humilis*. **i** *Jilinus hulongensis*. **j** *Cornitibia simplicithorax*. **k** *Emertongone montifera*. Scale bars 0.5 mm

**Fig. 11:**
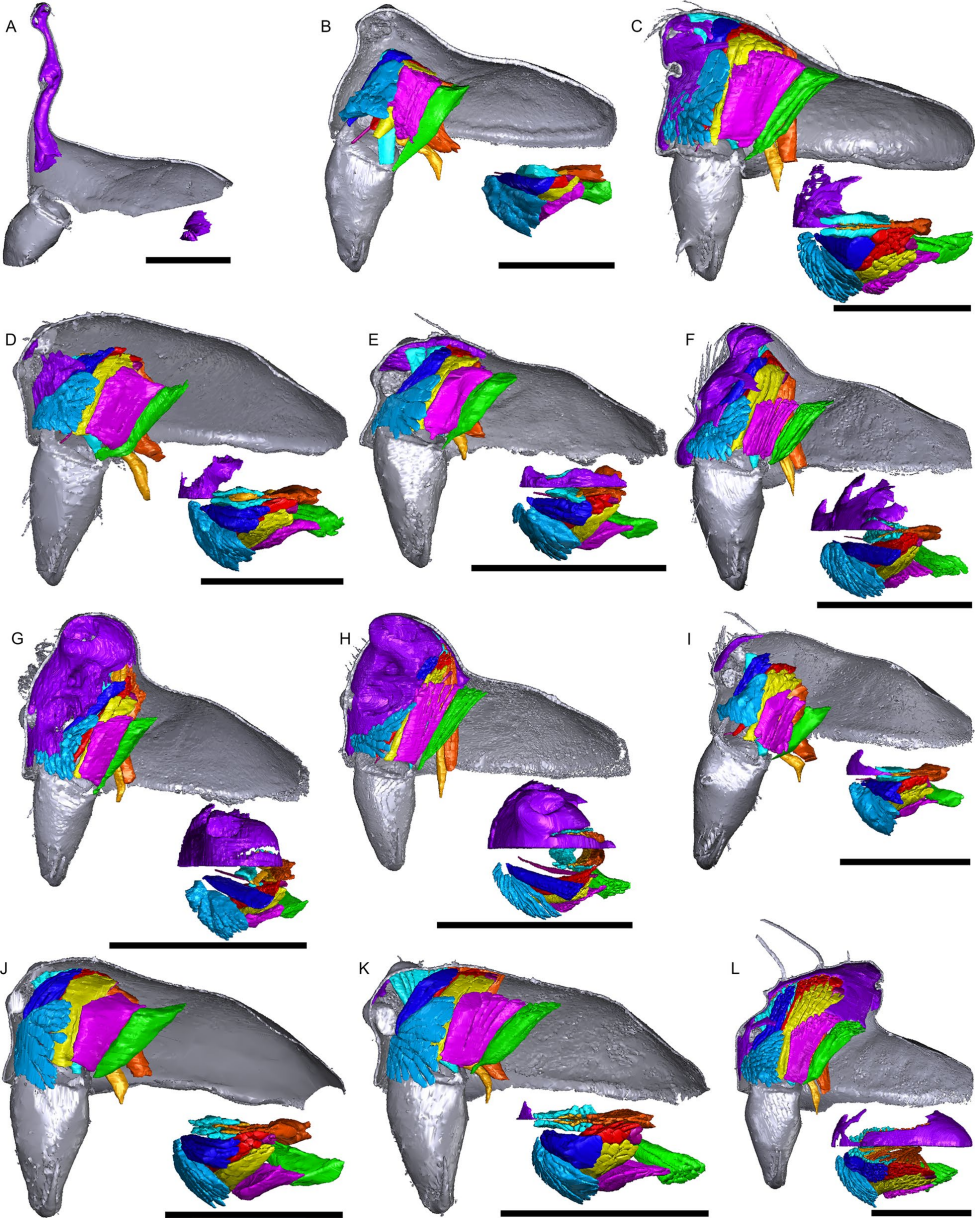
Images of micro-CT scans with gustatory glandular tissues (purple), different sets of cheliceral muscles (left side), pharyngeal dilators (both sides). The right side of the prosomal cuticle is digitally segmented and color-coded following Table 1. Interactive 3D images are available in the Additional File 6. Click on the image to activate individual 3D model; to hide/show different structures, right-click and select “show model tree”. **a** *Walckenaeria acuminata*. **b** *Gonatium rubellum*. **c** *Shaanxinus mingchihensis*. **d** *Oedothorax kodaikanal incertae sedis*. **e** *O. paracymbialis incertae sedis*. **f** *O. meghalaya incertae sedis*. **g** *Atypena cirrifrons*. **h** *A. formosana*. **i** *Oedothorax uncus incertae sedis*. **j** *O. cunur incertae sedis*. **k** *O. stylus incertae sedis*. **l** *Nasoona setifera*. Scale bars 0.5 mm

**Fig. 12:**
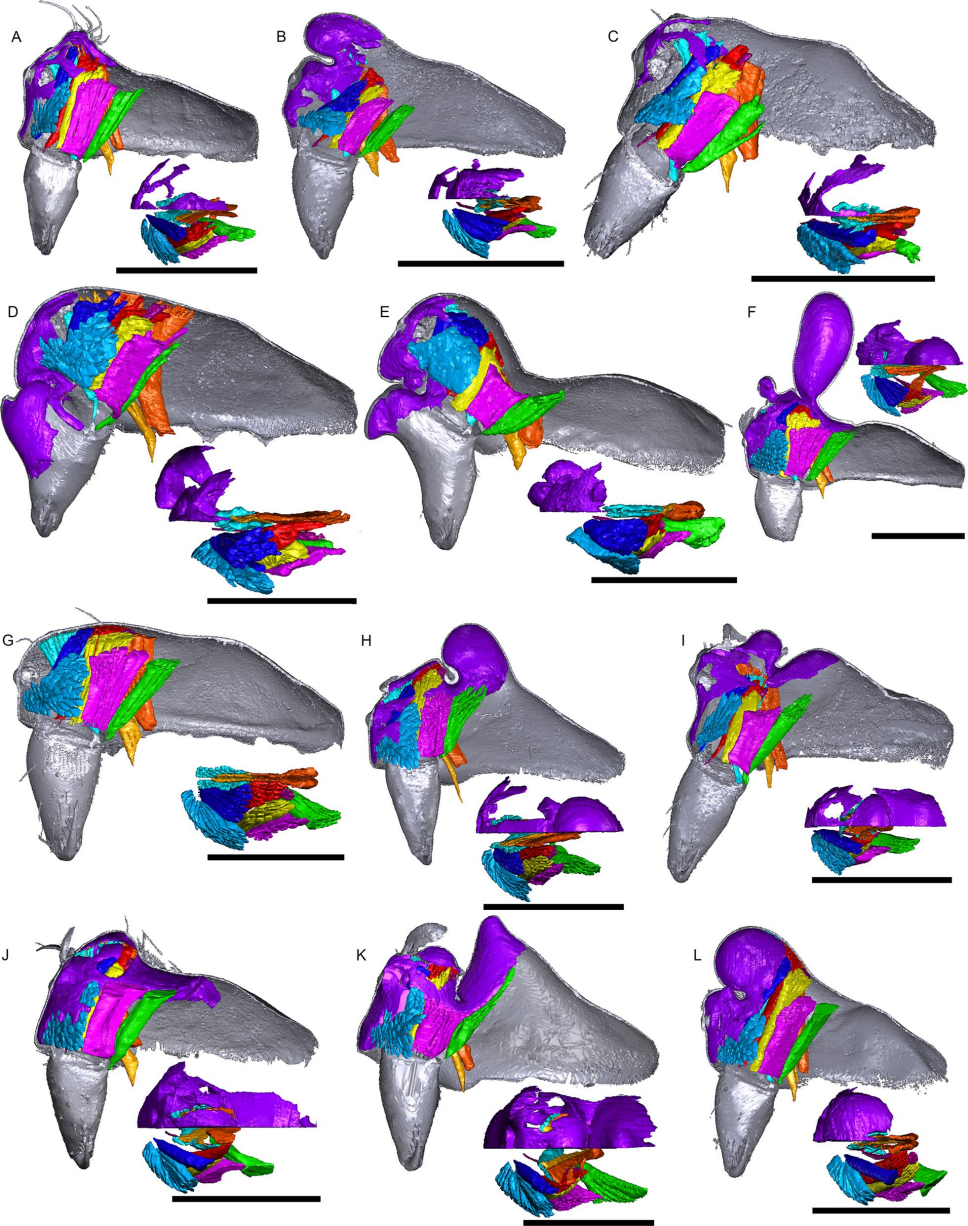
Images of micro-CT scans with gustatory glandular tissues (purple), different sets of cheliceral muscles (left side), pharyngeal dilators (both sides). The right side of prosomal cuticle is digitally segmented and color-coded following Table 1. Interactive 3D images are available in the Additional File 7. Click on the image to activate individual 3D model; to hide/show different structures, right-click and select “show model tree”. **a** *Nasoona crucifera*. **b** *Mitrager globiceps*. **c** *M. hirsuta*. **d** *M. clypeellum*. **e** *M. elongata*. **f** *M. noordami*, male. **g** *M. noordami*, female **h** *M. cornuta*. **i** *M. villosa*. **j** *M. angela*. **k** *M. coronata*. **l** *M. sexoculorum*. Scale bars 0.5 mm

**Fig. 13:**
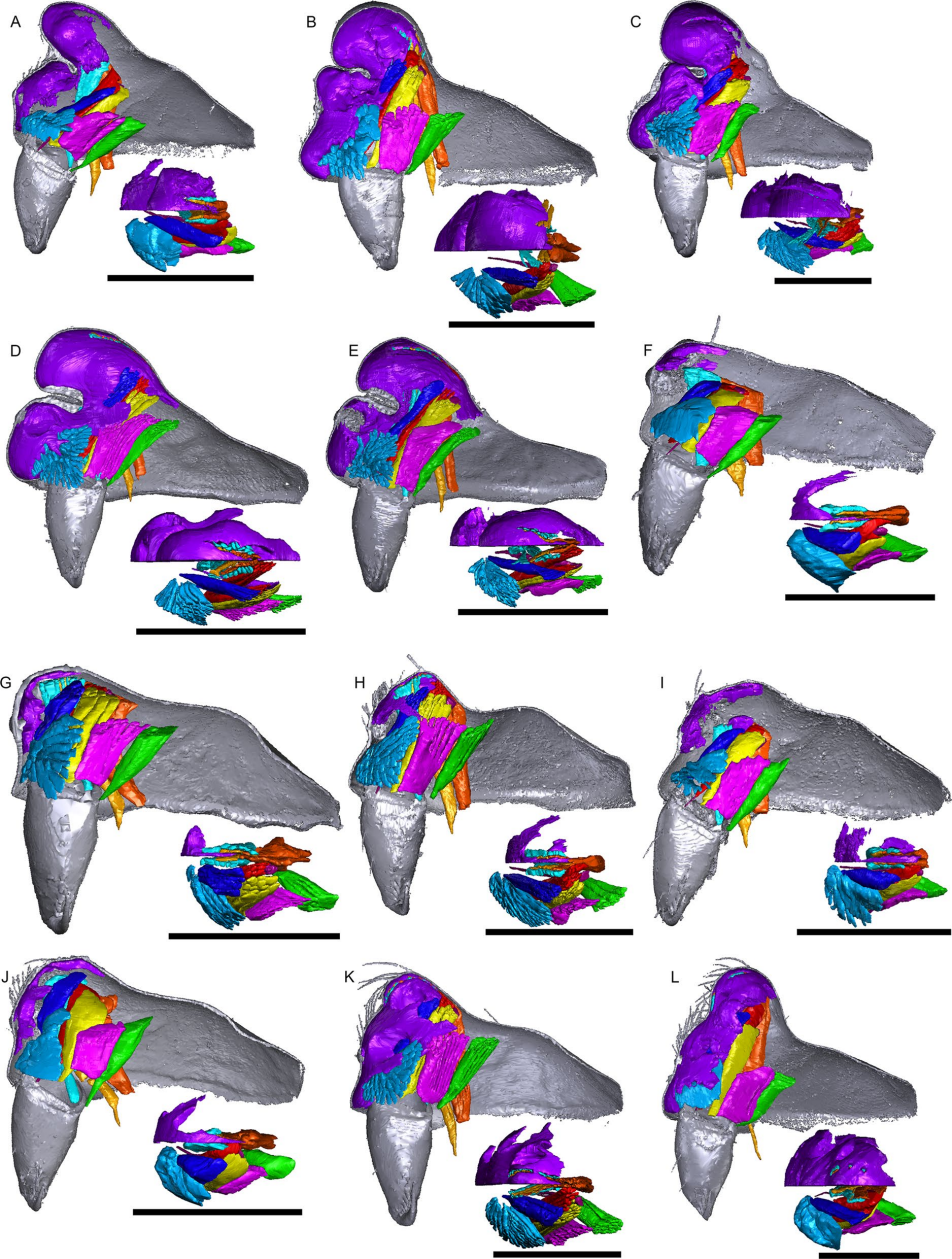
Images of micro-CT scans with gustatory glandular tissues (purple), different sets of cheliceral muscles (left side), pharyngeal dilators (both sides). The right side of the prosomal cuticle is digitally segmented and color-coded following Table 1. Interactive 3D images are available in the Additional File 8. Click on the image to activate individual 3D model; to hide/show different structures, right-click and select “show model tree”. **a** *Mitrager lineata*. **b** *M. dismodicoides*. **c** *M. tholusa*. **d** *M. lucida*. **e** *M. sexoculata*. **f** *M. unicolor*. **g** *M. rustica*. **h** *M. assueta*. **i** *M. malearmata*. **j** *M. lopchu*. **k** *M. falciferoides*. **l** *M. falcifer*. Scale bars 0.5 mm

**Fig. 14:**
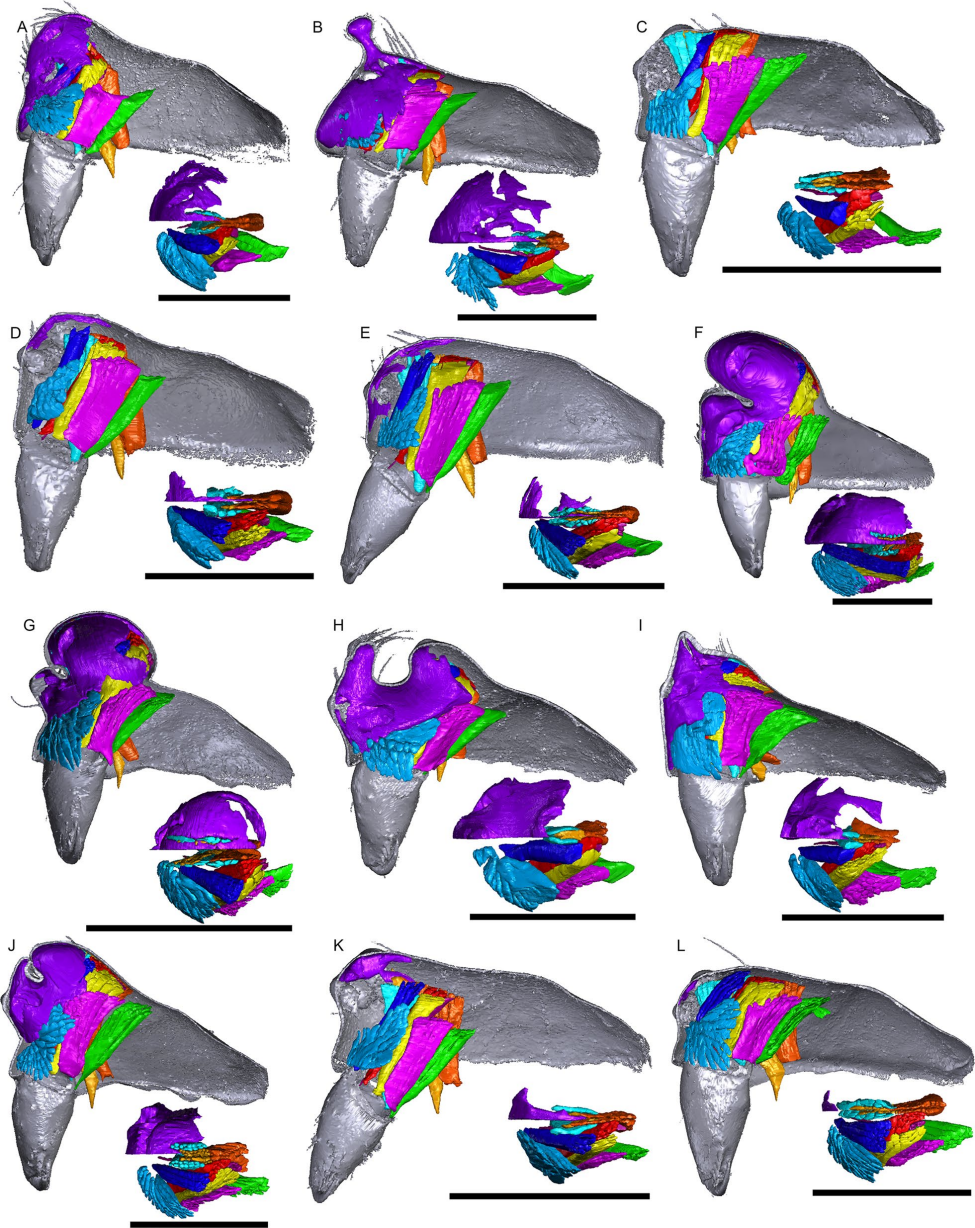
Images of micro-CT scans with gustatory glandular tissues (purple), different sets of cheliceral muscles (left side), pharyngeal dilators (both sides). The right side of the prosomal cuticle is digitally segmented and color-coded following Table 1. Interactive 3D images are available in the Additional File 9. Click on the image to activate individual 3D model; to hide/show different structures, right-click and select “show model tree”. **a** *Mitrager modesta* (Tanasevitch, 1998). **b** *M. savigniformis* (Tanasevitch, 1998). **c** *Holmelgonia basalis*. **d** *Callitrichia holmi*. **e** *Ca. picta*. **f** *Ca. gloriosa*. **g** *Ca. convector*. **h** *Ca. sellafrontis*. **i** *Ca. juguma*. **j** *Ca. uncata*. **k** *Ca. pilosa*. **l** *Ca. muscicola*. Scale bars 0.5 mm

**Fig. 15:**
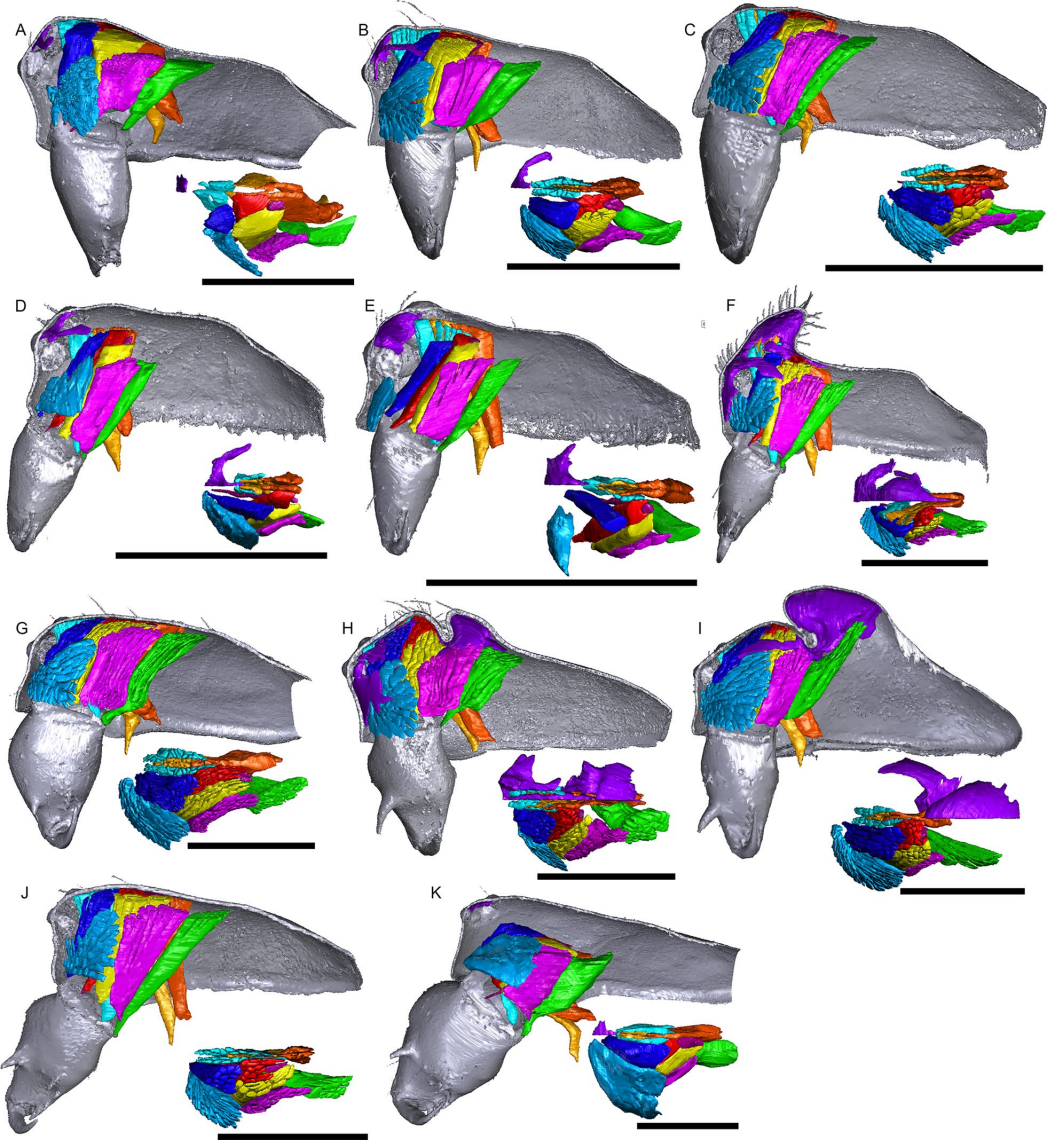
Images of micro-CT scans with gustatory glandular tissues (purple), different sets of cheliceral muscles (left side), pharyngeal dilators (both sides). The right side of the prosomal cuticle is digitally segmented and color-coded following Table 1. Interactive 3D images are available in the Additional File 10. Click on the image to activate individual 3D model; to hide/show different structures, right-click and select “show model tree”. **a** *Callitrichia latitibialis*. **b** *Ca. longiducta*. **c** *Ca. usitata*. **d** *Ca. legrandi*. **e** *Ca. macropthalma*. **f** “*Oedothorax*” *nazareti*. **g** *Gongylidium rufipes*. **h** *Ummeliata insecticeps*. **i** *U. esyunini*. **j** *Hylyphantes graminicola*. **k** *Tmeticus tolli*. Scale bars 0.5 mm

Figure 1 shows the internal structures of males and females of *Oedothorax gibbosus*: the glandular tissues (purple), the central nervous system (magenta), the venom glands (red), the muscles (light blue), and the digestive system (green). Epidermal tissue that appeared homogenous was found only in the males, closely associated with the modified prosomal area (Fig. 1c–f, purple). The distribution of this type of tissue in the scans of both morphs of *Oedothorax gibbosus* male (Figs. 1c–f, 2a, b, 3a, b) is in congruence with the area marked as possessing the glandular epithelium (Figs. 7a, 9a in [36]). In this reference paper [36], the occurrence and cellular setup of the glandular tissue in several *Oedothorax* species was described with semithin histology and transmission electron microscopy. The comparison allowed us to infer from the appearance of a given tissue in the micro-CT scan to the presence or absence of gustatory epithelial glands. We then delineated the tissue as such in the erigonine males of the current study (outlined in purple; Figs. 3, 4, 5, 6, 7, 8, 9 3D reconstructions, Figs. 2, 10, 11, 12, 13, 14, 15). An amorphous tissue for unknown function occurring in both males and females was coloured in dark blue (Fig. 1). This tissue generally occurs around the organs and between muscles and does not show sexual dimorphism.

### Variation in the distribution of glandular tissues and cheliceral/pharyngeal muscles

**Fig. 16:**
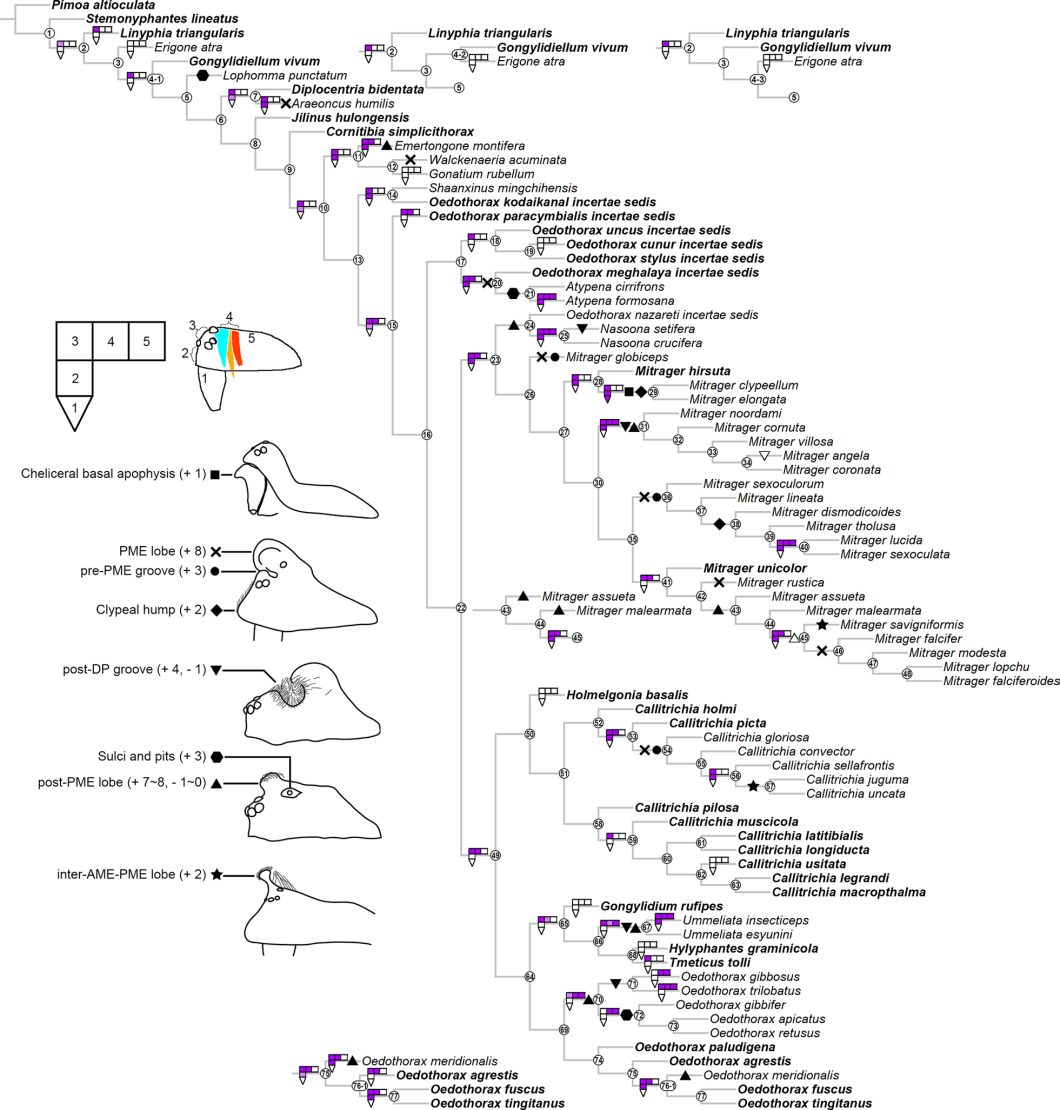
Phylogenetic tree of the studied taxa, with the character state transformation of prosomal gustatory gland distribution and external modifications shown at the nodes. Species without external prosomal modifications are marked in bold. The prosoma is divided into five regions of gustatory gland distribution as shown in the schematics: 1, chelicerae; 2, before-eye region; 3, eye region; 4, space between both sides of inter-cheliceral-sclerite muscles (light blue) and anterior/posterior pharyngeal dilators (light/dark orange); 5, posterior to posterior pharyngeal dilators

**Fig. 17:**
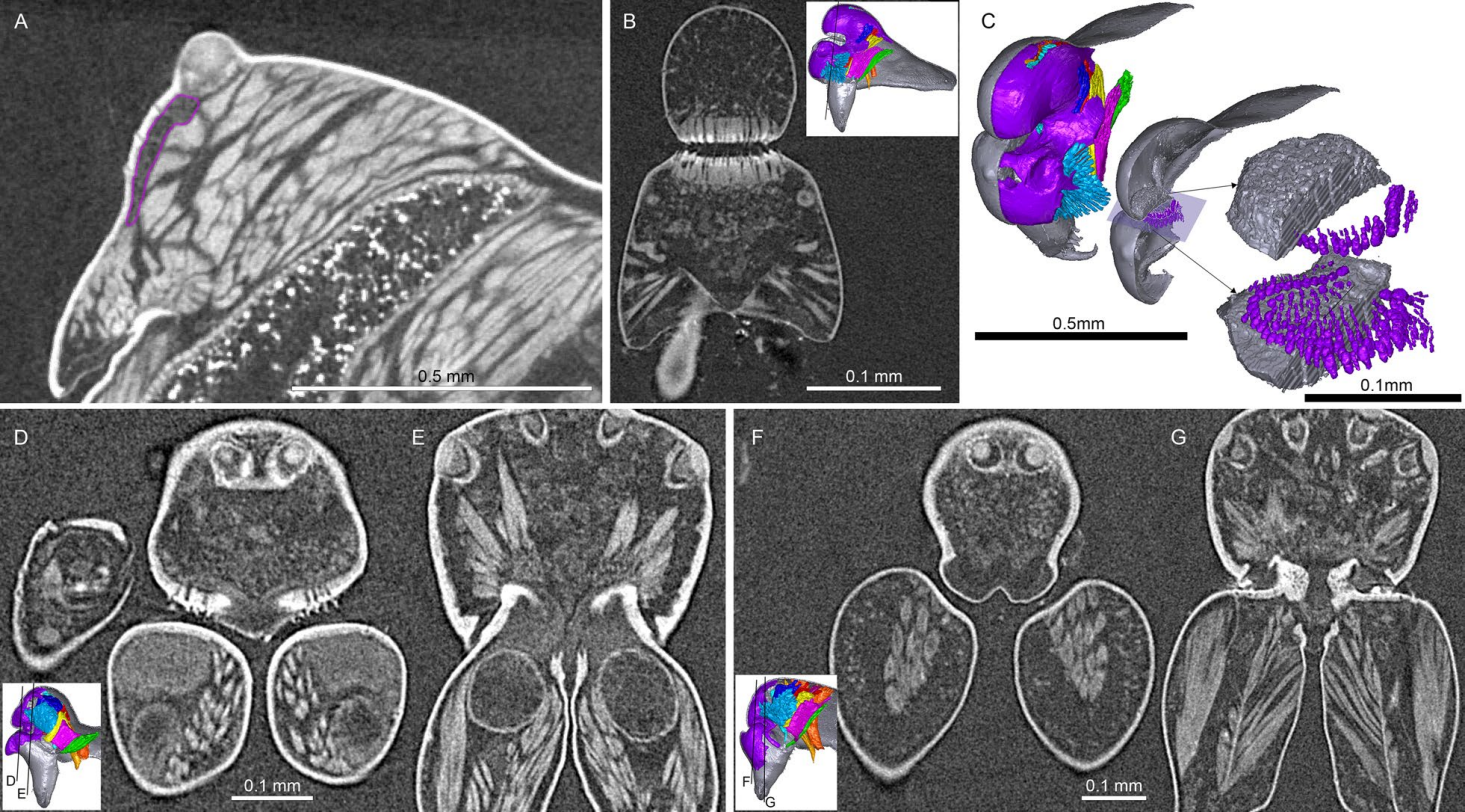
Virtual slices and 3D models reconstructed from micro-CT scans. **a** *Linyphia triangularis*, virtual slice parallel to the sagittal plane, showing the epidermal glandular tissue (outlined in purple) anterior to the posterior median eye (PME). **b**, **c** *Mitrager lucida*. **b** frontal plane, showing the cuticular canals at the setal bases in the pre-PME groove. **c** reconstruction of the setal morphology in the pre-PME groove. **d**, **e** two slices on the frontal plane of *M. elongata*, showing the cuticular canals on the ventral side of the clypeus. **f**, **g** two slices on the frontal plane of *M. clypeellum*, showing the cuticular canals on the cheliceral bases

Gustatory glands were found in males of all included 46 species of erigonines with obvious sexually dimorphic prosomal shapes, except for *Erigone atra*. Gustatory glands were also found in 23 out of the 27 males of erigonine species that lack external dimorphic structures (species without external dimorphic structures are given in bold in Fig. 16). In the non-erigonine taxa included in the current study, glandular tissue is present in the eye region of *Linyphia triangularis* (Fig. 17a). However, *L. triangularis* possesses two small glandular areas, one on each side of the prosoma between the anterior median and the anterior lateral eyes. In the erigonines with gustatory glandular tissue in these areas, there is one large glandular area that spans from one side to the other. The effect of tissue shrinkage on the attachment of tissues to the cuticle is reported in the Additional File 1.

The gustatory gland distribution among the studied species varies considerably: from close to the anterior margin of the before-eye region (e.g., *Oedothorax meridionalis*, Fig. 2j) to the region adjacent to the anterior margin of the central posterior infolding of the prosoma (i.e., the fovea; e.g., *O. gibbosus*, Fig. 2a, b). In *Mitrager clypeellum* and *M. elongata*, the gustatory glands extend anteriorly and proximally into the chelicerae and seem to be connected to the gustatory glands in the before-eye and eye regions (Fig. 12d, e respectively). When gustatory glands occur in an area between attachment areas of different muscles, there are increased intervals between these muscles. For instance, the lateral anterior muscle and the lateral posterior muscle are adjacent to each other in *Oedothorax retusus* without gustatory glandular tissue between them (Fig. 2g), while these muscles are spatially separated to different degrees in the *Oedothorax* species in Clade 74 (Fig. 2h–l). In many species, gustatory glandular tissues occur medially in the positions of the inter-cheliceral-sclerite muscle, anterior pharyngeal dilator, and posterior pharyngeal dilator, while the dorsal attachment points of these muscles are symmetrically separated in various degrees along the longitudinal axis (e.g., slightly in *Oedothorax paludigena*, Fig. 2h; strongly in *Mitrager coronata*, Fig. 12k).

**Fig. 18:**
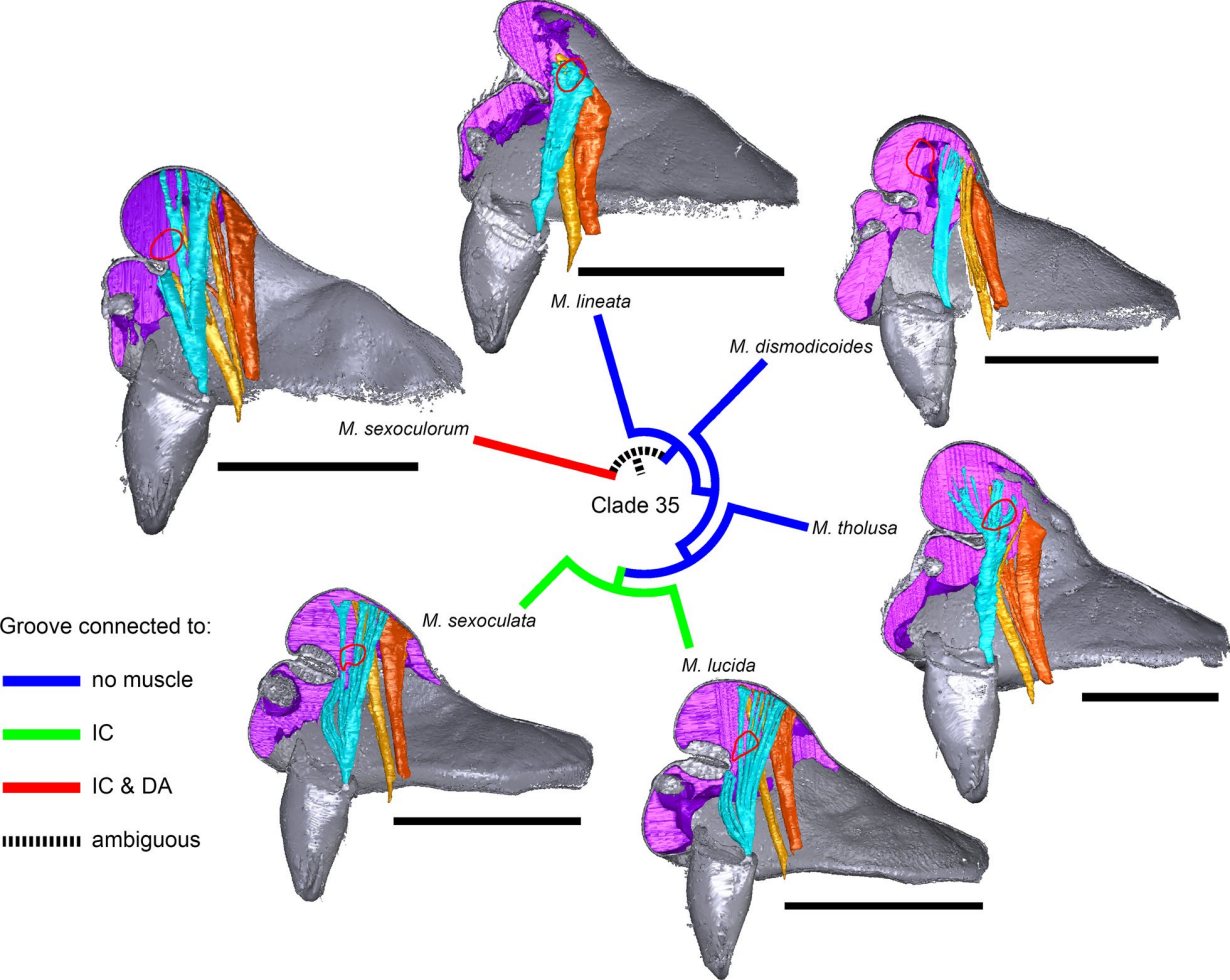
Images of micro-CT scans of *Mitrager sexoculorum, M. lineata*, *M. dismodicoides*, *M. tholusa*, *M. lucida* and *M. sexoculata*, showing the right side of cuticle (grey) and gustatory glandular tissues (purple), and the inter-cheliceral-sclerite muscle (light blue), the anterior (light orange) and posterior (dark orange) pharyngeal dilators. The position of the right posterior medial eye is outlined with red. Scale bars 0.5 mm

**Fig. 19:**
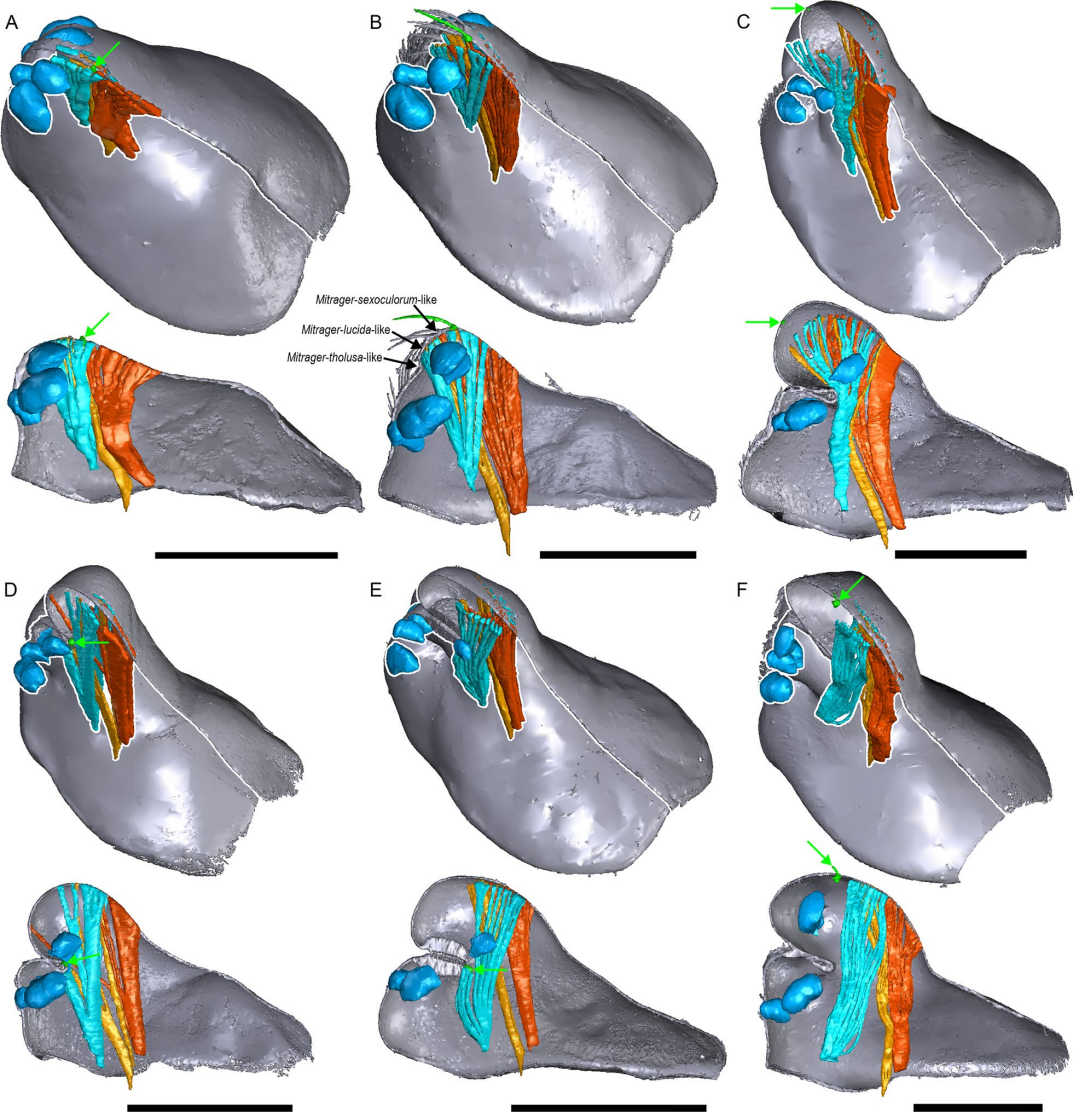
Comparison between several *Mitrager* species and one *Callitrichia* species with different degrees and types of prosomal modifications. The filaments of the inter-cheliceral-sclerite muscle (aqua) and the anterior (light orange) and posterior (dark orange) pharyngeal dilators are extrapolated onto the cuticle surface for visually presenting the places of the muscle attachments; the macroseta on the central axis positioned behind the ocular region is marked in green and pointed at by green arrows; The black arrows in B mark the hypothesized points in the eye region, where the cuticle might have invaginated and formed the pre-PME groove in different species; the eyes are marked in light blue. **a** *M. rustica*. **b** *M. falciferoides*. **c** *M. tholusa*. **d** *M. sexoculorum*. **e** *M. lucida*. **f** *Ca. gloriosa*. Scale bars 0.5 mm

In species with prosomal modifications, the extent of the dorsal attachment of the pharyngeal dilators varies along the longitudinal axis, ranging from narrow (*Oedothorax gibbosus*, Fig. 2a) to wide (*O. gibbifer*, Fig. 2e). In addition, externally similar shapes of the male prosomata may present differences in internal attachments of gustatory glands and muscles. For example, in species with a pre-PME groove, three patterns of muscle attachments related to the groove are observed (see Fig. 18): (1) no muscle attached to the groove (e.g., *Mitrager dismodicoides*); (2) one branch of the inter-cheliceral-sclerite muscle attached to the groove (e.g., *M. lucida*); (3) one branch of both the inter-cheliceral-sclerite muscle and the anterior pharyngeal dilator attached to the groove (e.g., *M. sexoculorum*). In the species with the inter-cheliceral-sclerite muscle or the inter-cheliceral-sclerite muscle and anterior pharyngeal dilator attached to the groove, the PMEs are close to the upper side of the groove and not exposed. The spatial relationships between the PMEs, the inter-cheliceral-sclerite muscle, the anterior pharyngeal dilator and the central macroseta are consistent across erigonine taxa with different degrees of prosomal modification (Fig. 19). For instance, in *Mitrager tholusa* (Fig. 19c), the attachments of the inter-cheliceral-sclerite muscle, anterior pharyngeal dilator and posterior pharyngeal dilator have more anterior positions in the PME lobe, which coincide with the more anterior position of the central macroseta compared to that in *M. rustica* and *M. falciferoides* (Fig. 19a, b respectively) and *Callitrichia gloriosa* (Fig. 19f); in *M. sexoculorum* and *M. lucida*, in which the anterior filaments of the inter-cheliceral-sclerite muscle or both the inter-cheliceral-sclerite muscle and posterior pharyngeal dilator are attached to the groove, the central macroseta is located inside the groove (Fig. 19d, e respectively).

**Fig. 20:**
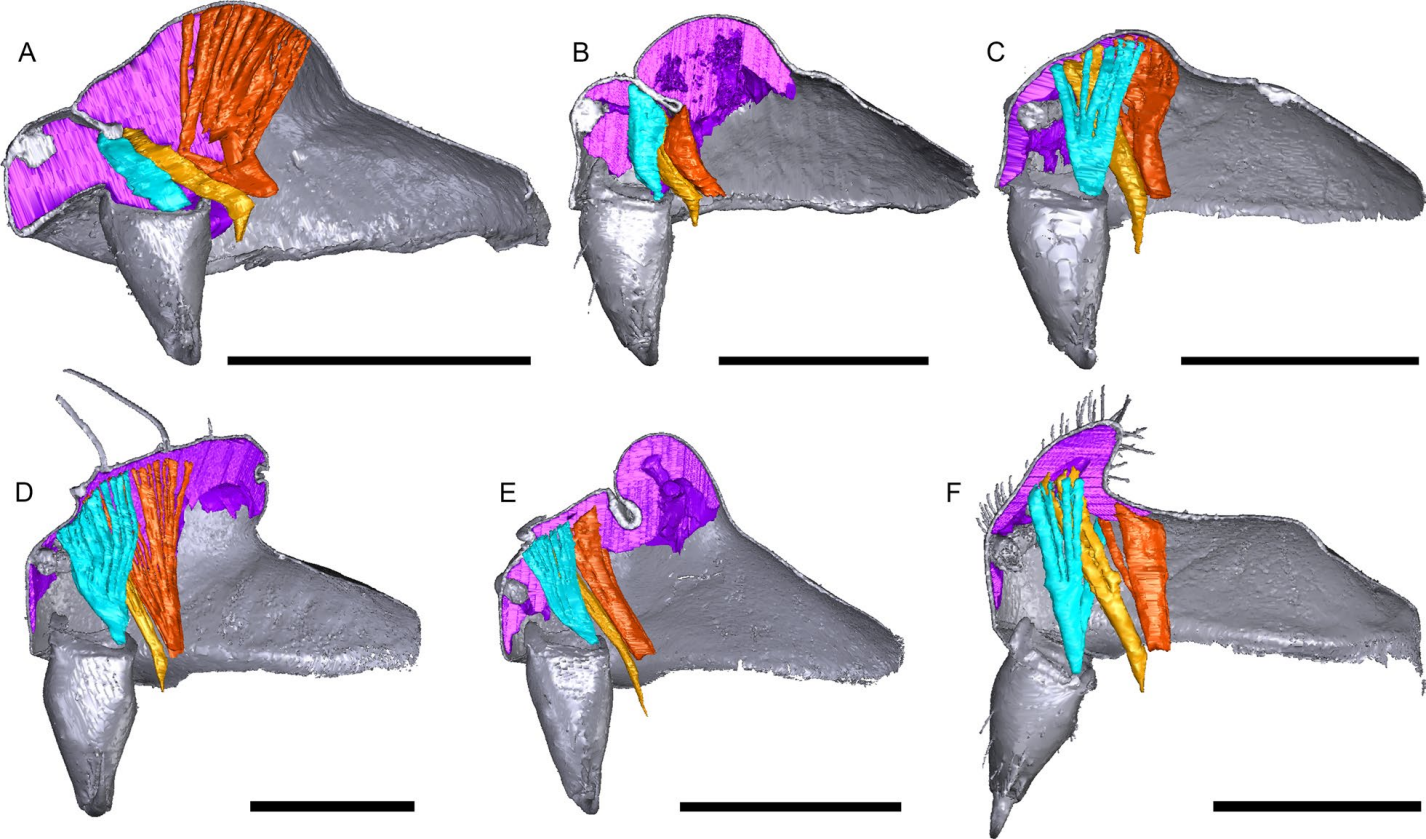
Images of micro-CT scans of species possessing post-PME lobes, showing the right side of cuticle (grey) and gustatory glandular tissues (purple), and the inter-cheliceral-sclerite muscle (light blue), the anterior (light orange) and posterior (dark orange) pharyngeal dilators. **a** *Emertongone montifera*. **b** *Oedothorax trilobatus*. **c** *O. meridionalis*. **d** *Nasoona setifera*. **e** *Mitrager cornuta*. **f** *O. nazareti incertae sedis*. Scale bars 0.5 mm

In species with a post-PME lobe, the internal attachment of the inter-cheliceral-sclerite muscle, anterior pharyngeal dilator and posterior pharyngeal dilator varies greatly among species. For instance, all three muscles are attached anterior to the post-PME groove (*Mitrager cornuta*, Fig. 20e); all three muscles are attached to the anterior side of the post-PME groove (*Oedothorax trilobatus*, Fig. 20b); only the posterior pharyngeal dilator is attached to the posterior half of the post-PME lobe, but neither the inter-cheliceral-sclerite muscle nor the anterior pharyngeal dilator (*Emertongone montifera*, Fig. 20a); all three muscles are attached to the anterior half of the post-PME lobe (*Nasoona setifera*, Fig. 20d); all three muscles are attached to most of the extent of the post-PME lobe (*O. meridionalis*, Fig. 20c); the inter-cheliceral-sclerite muscle and anterior pharyngeal dilator are attached to most of the extent of the post-PME lobe, the posterior pharyngeal dilator is attached to the posterior side of the lobe and its attachment extends further posteriorly into the prosoma (*O. nazareti incertae sedis*, Fig. 20f).

### Cuticular structures revealed in micro-CT reconstruction

The resolution of our Micro-CT analysis did not allow to detect minute prosomal cuticular pores as were found using scanning electron microscopy (SEM) by [26] (see e.g. plate 20B, C, E, F in [26]). These pores are present in isolation or in groups and are not associated with other cuticular structures such as setae [26]. However, larger canals at the base of setae were discernable by micro-CT; their distribution varies among species. For instance, in the two closely related species *Mitrager clypeellum* and *M. elongata*, both of which with cheliceral apophyses, cuticular canals are present close to the junction of the clypeus and chelicerae (Fig. 17d–g): whereas they are found on the underside of the elevated clypeus in *M. elongata*, similar canals occur in *M. clypeellum* at the basal-most part of the chelicerae. Virtual sections on the sagittal plane of *Mitrager lucida* and *M. sexoculata* also show such canals in the thickened cuticle on the upper and lower sides of their inter-AME-PME grooves (Fig. 7d, e; see virtual slice on the frontal plane in Fig. 17b). These canals are located at the bases of the modified stout setae, which so far have only been found in these two species (modified setae are reconstructed in Fig. 17c). Whether these canals function as openings for the secretion of glandular products remains to be investigated by histological methods.

### Clade stability and character evolution

**Fig. 21:**
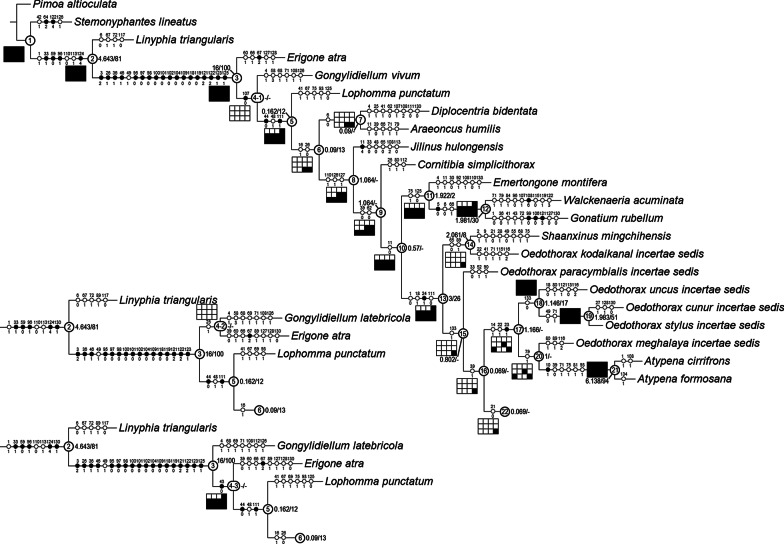
The first part of the six most parsimonious trees from the phylogenetic analysis, with unambiguous character optimization (circles on branches), clade numbers (in circles on nodes) and Bremer/Jackknife support values (beside the nodes). The presence/absence of clades in the trees of the implied weights analysis with different *k* values are shown in the boxes under/above/on the branches: black for presence, white for absence

**Fig. 22:**
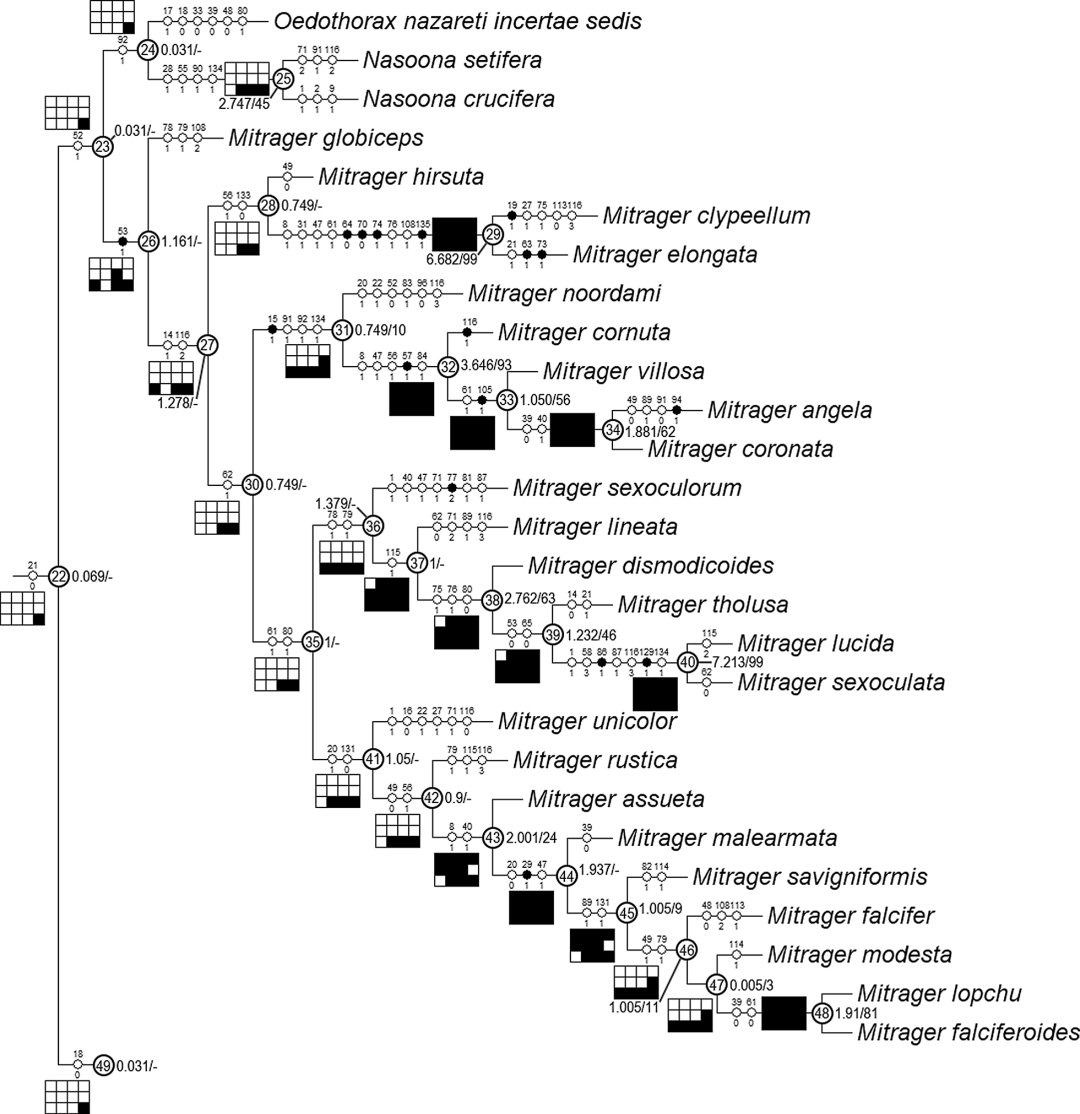
Continuation of Fig. 21, showing Clade 22 (without Clade 49) to 48

**Fig. 23:**
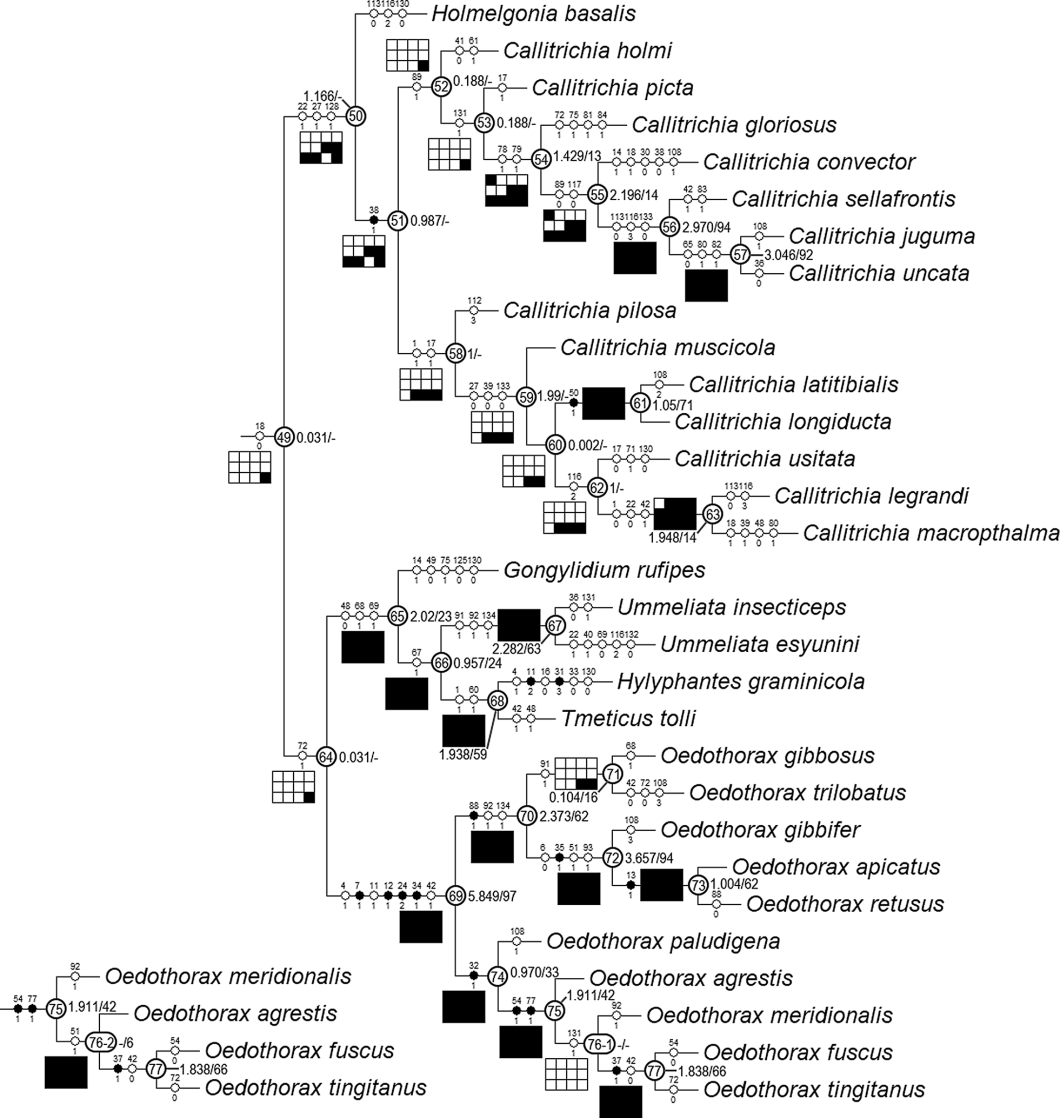
Continuation of Figs. 21 and 22, showing Clade 49

The equal weight parsimony analysis resulted in six most parsimonious trees (MPT, tree length = 531.37, CI = 0.312, RI = 0.637, Figs. 21, 22, 23), in which Clade 1 to Clade 13 are identical to the topologies of the MPTs from the analysis of Matrix II in [29]; three major clades (*Mitrager*, Clade 26; *Holmelgonia* + *Callitrichia, Clade* 50; *Oedothorax* (Clade 69, monophyletic) + *Gongylidium* + *Ummeliata* + *Hylyphantes* + *Tmeticus*, Clade 64) each appear to be monophyletic.

The resulting trees from different implied-weighting schemes are summarized in Table 2, reporting the monophyly/polyphyly of three major clades: Mitrager, Clade 26; *Holmelgonia* + *Callitrichia, Clade* 50; *Oedothorax* + *Gongylidium* + *Ummeliata* + *Hylyphantes* + *Tmeticus*, Clade 64. *Mitrager* appeared polyphyletic under strong to moderate *k* values (1–6); when *k* = 15 and 30, *O. meghalaya incertae sedis* occurred within a clade of *Mitrager*; *Ca. convector* was placed outside Clade 50 under strong to moderate *k* values (2–6), while it remained within Clade 50 under moderate to gentle *k* values (10–1000). With *k* = 4, 30 and 100, *O. nazareti incertae sedis* was placed in Clade 64.

Character state transformation optimization based on parsimony is summarized in Fig. 16 for both the external modifications and the internal gland distribution characters. Our evolutionary hypothesis suggests that the presence of gustatory glandular tissues in the eye region is the ancestral condition for either all erigonines or for all erigonines except *Erigone atra*. The expansion of gustatory glandular tissue from the eye region into the before-eye and/or pharynx muscle region occurred multiple times, as well as its retraction/reduction (e.g., the gustatory glands expanded into the before-eye region between nodes 10 and 23, then into the pharynx muscle region at node 23, and further into the post-DP region at node 25; but retracted from before-eye region at node 41 and expanded into this region again at node 45). In Clade 51, the distribution of gustatory gland shifted anteriorly at node 53 and the prosomal modifications occurred at node 54, whereas in Clade 59 the distribution reduced to only in the before-eye region (absent in *Callitrichia usitata*), and no external modification evolved. In *Oedothorax* (Clade 69), the gustatory gland distribution shifted or expanded posteriorly into the post-DP region at node 70, whereas it extended anteriorly into the before-eye region at either node 75 or 76-1. All prosomal modifications that evolved within clades are based on the presence of gustatory glandular tissues in the corresponding prosomal area at a more basal node, except for the cheliceral apophyses and the cheliceral gustatory gland. Based on the current taxon sample, it cannot be determined whether gustatory glands evolved prior to the occurrence of the apophyses in this region. Loss of gustatory glandular tissue occurred frequently during the evolution of erigonines, as seven instances of gustatory gland reduction can be inferred, all of which occur in terminal branches (indicated by the non-colored prosoma schematics in Fig. 16; loss/gain ratio = 7/1). As it was found in [29], most of the prosomal external modifications have multiple origins except the cheliceral basal apophyses. For differences in the degree of homoplasy of prosomal structures, see Additional File 1.

In Clade 36 within *Mitrager*, where all six species possess a pre-PME groove, the ancestral state of the inter-cheliceral-sclerite muscle and anterior pharyngeal dilator attachment to the internal surface of the groove is ambiguous (Fig. 18); a shift of the anterior part of the cuticular attachment of the inter-cheliceral-sclerite muscle from posterior to the groove onto the internal surface of the groove occurred in Clade 40.

## Discussion

The astonishing diversity of erigonine male prosomal modifications has been the focus of many studies on this spider group [19, 29, 38]. Their function in gustatory courtship has been established in behavioral studies [20, 21], and a close association with extensive gustatory glandular tissues has been demonstrated by previous histological studies [36, 40]. An evolutionary scenario depicting an origin of internal gustatory glandular tissues prior to the diversification of external morphologies [36, 40] has been proposed based on several erigonine phylogenetic frameworks in which external morphological characters were analyzed [26, 66, 67]. The current study provides the first phylogenetic analysis that incorporates both the external morphology and the internal gustatory gland distribution for reconstructing their evolutionary pattern and testing the aforementioned hypothesis. These results shed light on the lability of sexually selected gustatory traits and their potential to influence speciation in erigonine spiders.

### Implications of differences in muscle connections to prosomal structures

We discovered the diversity of muscle connections to the pre-PME groove in the six species in Clade 36 (Fig. 18). In species with different degrees and forms of prosomal modifications (e.g., without modification, *Mitrager rustica*, and with PME lobe, *M. falciferoides*, Fig. 19a, c, respectively), the PMEs are always approximately aligned with the anterior filaments of the inter-cheliceral-sclerite muscle and the posterior pharyngeal dilator along the longitudinal axis. The connections of the anterior filaments of the inter-cheliceral-sclerite muscle and the anterior pharyngeal dilator to the pre-PME groove in species like *Mitrager sexoculorum* and *M. lucida* seem to be related to the internal position of the PMEs close to the upper side of the groove (see the positions of the eyes outlined with red in Fig. 18; Fig. 19d, e). Therefore, it seems plausible that during the ontogenesis of species that differ in muscle connections to the groove, the anchor point between the anterior and posterior elevations (i.e., the groove) differs also in the eye region (Fig. 18). In the case of *Mitrager sexoculorum* (connected to the inter-cheliceral-sclerite muscle and DA), the anchor point is located between the PMEs in a position on the longitudinal axis aligned with the posterior edge of the PMEs (Fig. 19b, upper black arrow); in *M. sexoculata* and *M. lucida* (connected to IC, Fig. 18), this point is located slightly more anteriorly, approximately in a position aligned with the center of the PMEs, not beyond the anterior-most attachment of the inter-cheliceral-sclerite muscle (Fig. 19b, middle black arrow); in the case of *M. lineata*, *M. tholusa*, *M. dismodicoides* and the *Callitrichia* species in Clade 54 (no muscle attachment, e.g. *Ca. gloriosa*, Fig. 19f), the center of the groove is situated between the AMEs and PMEs, anterior to the inter-cheliceral-sclerite muscle and the anterior pharyngeal dilator (Fig. 19b, lower black arrow). We like to propose two evolutionary scenarios describing how these pre-PME grooves may have developed at different locations along the longitudinal axis. Firstly, independent (i.e., non-homologous) formations of a groove may have occurred in species with a PME lobe, like *M. falciferoides*, at different locations along the longitudinal axis (Fig. 19b, black arrows). Alternatively, shifts of the central point of the pre-PME groove along the longitudinal axis could have occurred after the evolution of this groove. Our results imply two shifts in position of the central point of the pre-PME groove in Clade 36, suggesting that the differences in its position among species do not necessarily imply independent origins of this groove (i.e., the first abovementioned scenario). A possible cause of these shifts could be changes in the positions at which female mouthparts contact the male prosomal lobe, in concert with changes of nuptial-gift-secreting areas to more anterior or posterior positions. This in turn could explain the changes in gustatory gland distribution in other erigonine groups such as *Oedothorax* (Clade 69).

### Evolution of gustatory glandular tissues and external modifications

Our reconstruction of character state transformation reveals a single origin of gustatory glands, and multiple origins of various types of male prosomal external modifications (Fig. 16); the presence of glands in different prosomal areas also preceded the evolution of external structures at their corresponding positions. These findings support the hypothesis that the gustatory glands evolved in sexually monomorphic ancestors before changes in the prosomal shapes occurred [40]. We also present evidence for multiple instances of gustatory gland reduction. Interestingly, the pattern of character state transformation on the current phylogenetic tree strongly suggests that after prosomal modifications had evolved in a clade, none of its members lost the trait complex of shapes and gustatory glands. However, in the cases of total reduction of gustatory glandular tissue, it is unlikely that the ancestral state possessed prosomal modifications. This may imply that once more elaborate male prosomal structures had evolved in a species, females became less likely to lose the preference for nuptial-gift-providing males. The benefits of nuptial feeding might exceed the costs of developing these traits, and thus they are more likely to be retained.

The cases of loss of gustatory gland suggest selective scenarios in the course of evolution that favored a decreased investment in gustatory courtship. The loss or reduction of sexually selected male traits has been demonstrated in insects and all major groups of vertebrates [68], and the loss/gain ratios can be high (5:1 for male coloration in tanagers [69]; 4:1 for colorful male ventral patches in phrynosomatid lizards [70]; 4:1 for clasping genitalia in water striders [71]). Although sexual selection may have been the primary force for the evolution and maintenance of gustatory glands and male prosomal modifications, natural selection and genetic drift might also have played a role [68]. Studies on *Oedothorax gibbosus,* in which two male morphs occur, provide insights into the costs and benefits of the male trait complex. The *gibbosus* morph, which possesses a hump and a groove and extensive gustatory glandular tissue, requires a longer developmental time and lives shorter after maturation than the less modified *tuberosus* morph [72, 73]. Individual-based simulations based on the scenario in a *Oedothorax gibbosus* population also demonstrated that males investing more in attracting females miss out on mating opportunities due to longer development, thereby opening a mating niche for less elaborate male morphs [74]. Under less stable environmental conditions, shorter mating seasons and restricted resources, males that invest less in costly traits may be at a selective advantage. When species distribution becomes patchy and gene flow between local populations is low, the probability of loss of these male traits might further increase.

### Sexual selection on dimorphic male prosomal structure and speciation

Although the effect of sexual selection on population divergence has the potential to drive speciation, disagreements exist around whether sexual selection alone influences reproductive isolation, or whether it mostly acts alongside or in the shadow of natural selection [7, 10, 75]. Comparative studies that correlate estimates of the strength of sexual selection and species richness accounting for phylogenetic relatedness do not generally support the supposed association [10]. A meta-analysis found a small but significant overall correlation between sexual selection and speciation rate and a strong dependence on methodology and proxies for sexual selection [76]. Sexual dimorphism, which is often used as a proxy for sexual selection (40 of 64 studies), yielded inconsistent results. For example, a meta-analysis examined mammals, butterflies and spiders for associations between the degree of sexual size dimorphism and variance in species richness, and found no significant association [77]. This might be because sexual size dimorphism can result from various selective scenarios, such as intersexual competition for food resources [2, 11] and selection for larger females with higher fecundity [2, 78, 79]. In spiders, fecundity selection on females is the most likely explanation for the evolution of sexual size dimorphism [14, 15, 80, 81]. For assessing the impact of sexual selection on speciation, labile traits are required that are under sexual selection with little effect of various other sources of selection in generating trait diversity [76, 82, 83]. On the other hand, sexual selection does not necessarily accelerate diversification. For instance, when the trait optima under natural selection are more divergent than those under sexual selection, the latter may even show inhibitory effects on trait divergence among populations [84]. Female preference may drive male trait evolution, but whether it leads to species divergence depends on whether female mate preferences differ between populations [85]. Therefore, the influence of sexual selection on speciation rate lies more in its diversifying property than in its strength. The equivocal results of the comparative studies (reviewed in [9] and [76]) may partly be due to the negligence of this aspect [7, 85]. Therefore, instead of treating sexual selection in general, comparative studies trying to address its effect on speciation should distinguish between different selective scenarios for both male traits and female preferences [7, 76].

As demonstrated by our investigation on the prosoma of erigonine spiders, the gustatory trait complex is not only externally diverse in location and shape, but the internal gland distribution also varies greatly, even across species with moderate or no external modifications. This is well exemplified by the genus *Oedothorax*, in which species with no prominent prosomal elevations have gustatory glands located anteriorly (*O. fuscus*, Fig. 2k), medially (*O. agrestis*, Fig. 2i), or posteriorly (*O. gibbosus, tuberosus* morph, Fig. 2b). It is unlikely that factors other than sexual selection influence the lability of this trait complex, such as differences in niche use between sexes [11] and exposure to predation [86]. Difference in niche use between sexes are unknown in erigonines and unlikely to play a role during the major part of development since the traits are only expressed in adult males. Further, in species without external modifications, the divergent evolution in their gustatory gland distribution is even less likely to be influenced by differential niche use. As for mate selectivity, which causes the isolating effects of sexual selection, it has been demonstrated in *Oedothorax gibbosus* that non-virgin females are more likely to mate with *gibbosus*-morph males, which possess more elaborated prosomal traits, whereas the *tuberosus*-morph males have higher mating probability when exposed to virgin females [73]. Male *Oedothorax retusus* that had their nuptial-gift-secreting region experimentally covered were significantly less accepted for mating compared to a control group [21]. In addition, female ingestion of male secretion continues during mating, the spatial match of the structures involved in gustation and mating is supposedly under strong selection. Given the evidence for the importance of nuptial gift to male mating success and the divergent evolutionary patterns of the position of the gustatory glands associated with various external modifications, we suggest erigonines as a suitable target group for studies on the effect of sexual selection on speciation.

Our results point out erigonine clades that are of particular interest for future studies on sexual selection and speciation. *Callitrichia* – now divided into two clades—is represented by one clade with more prominent prosomal modification and wider gustatory gland distribution (Clade 52), and another clade with reduced gustatory gland distribution and no external modification (Clade 58). In addition, *Callitrichia* (55 species) [29] is sister to *Holmelgonia* (17 species) [87], which has no prosomal modification [29, 87] and no gustatory glandular tissue. Among the 48 *Callitrichia* species for which males have been described (not including *C. celans incertae sedis*), 30 species show various degrees of prosomal modifications, while 18 species have no external modification, among some of them possess gustatory glands. Another potential target group is Clade 65, which includes *Gongylidium* (without external modification and gustatory gland, 3 species), *Ummeliata* (with both external modification and gustatory gland, 10 species), *Tmeticus* (without external modification, with gustatory gland, 7 species) and *Hylyphantes* (without external modification and gustatory gland, 5 species) [25]. Given their species numbers and differences in their prosomal features, these taxa might lend themselves as suitable targets for comparative studies. The questions could be on the adaptive advantage of losing the gustatory glands, as well as whether lineages with more prominent prosomal modifications display higher speciation rates. Future sister group comparisons will require phylogenetic analyses with a more comprehensive taxon sampling that allows estimating speciation rates, combined with investigations of internal structures and ecological and behavioral aspects.

## Conclusions

The distribution pattern of gustatory glands revealed by the micro-CT investigation provided a new set of characters for phylogenetic analyses, as well as revealing further aspects of lability of the gustatory traits in dwarf spiders. The results of our phylogenetic analyses suggest an evolutionary scenario consistent with the hypothesis that the occurrence of the glandular tissues preceded the evolution of external prosomal modifications. For most external elevations (humps, lobes, and turrets), gustatory glandular tissues in the corresponding prosomal areas occurred already earlier in the phylogenetic tree. Incidences of glandular tissue loss indicate the cost of developing and maintaining the gustatory equipment. Even among species without obvious external prosomal modifications, differences in the distribution of gustatory glandular tissues were found. Our study provides a glimps into the dynamics of the evolution of sexually selected gustatory structures in erigonines. We suggest several erigonine target groups for comparative studies on and the effect of sexual selection on species divergence.

## Supplementary Information

**Additional file 1. Table S1**: Specimens used for microcomputed-tomography scans; differences between Matrix II in [29] and the current matrix; **Table S2**: Character matrix for the newly scored characters.

**Additional file 2.** Character matrix of the phylogenetic analysis.

**Additional file 3.** Interactive 3D images of Figs. 1A, C, E.

**Additional file 4.** Interactive 3D images of Figs. 2A-L.

**Additional file 5.** Interactive 3D images of Figs. 10A-K.

**Additional file 6.** Interactive 3D images of Figs. 11A-L.

**Additional file 7.** Interactive 3D images of Figs. 12A-L.

**Additional file 8.** Interactive 3D images of Figs. 13A-L.

**Additional file 9.** Interactive 3D images of Figs. 14A-L.

**Additional file 10.** Interactive 3D images of Figs. 15A-K.

## Data Availability

All data generated or analyzed during this study are included in this published article and its additional files.
