# Supplementary material for: Diversification through gustatory courtship: an X-ray micro-computed tomography study on dwarf spiders
Source: Front Zool. 2021 Sep 28;18:51. doi: 10.1186/s12983-021-00435-8 (PMC8480068; doi:10.1186/s12983-021-00435-8)
Supplement: Supplementary file 1 — Additional file 1. Table S1: Specimens used for microcomputed-tomography scans; differences between Matrix II in [29] and the current matrix; Table S2: Character matrix for the newly scored characters. [file 12983_2021_435_MOESM1_ESM.docx]

### Additional file 1

Supplementary table 1. Specimens used for microcomputed-tomography scans

| Genus | species | Author | Sex | Collection locality, year | Voucher number | Objective | Pixel size | Prosoma width (mm) |
| --- | --- | --- | --- | --- | --- | --- | --- | --- |
| *Araeoncus* | *humilis* | (Blackwall, 1841) | ♂ | Germany, 2014 | ZIMG | 10X | 0.9546 | 0.56 |
| *Atypena* | *cirrifrons* | (Heimer, 1984) | ♂ | Laos, 2009 | SMF 64983-124 | 10X | 1.0329 | 0.59 |
| *Atypena* | *formosana* | (Oi, 1977) | ♂ | Japan, 1995 | SMF 56485 | 20X | 1.0404 | 0.62 |
| *Callitrichia* | *convector* | (Tanasevitch, 2014) | ♂ | Thailand, 1987 | MHNG holotype | 20X | 1.1291 | 0.55 |
| *Callitrichia* | *gloriosa* | (Jocqué 1984) | ♂ | South Africa, 2002 | RMCA | 10X | 1.5040 | 0.94 |
| *Callitrichia* | *holmi* | (Wunderlich, 1978) | ♂ | Tanzania, 1995 | ZMUC | -* | -* | 0.70 |
| *Callitrichia* | *juguma* | (Scharff, 1990) | ♂ | Tanzania, no date | ZMUC | 20X | 1.0404 | 0.75 |
| *Callitrichia* | *latitibialis* | (Bosmans, 1988) | ♂ | Cameroon, 1983 | RMCA 165079 holotype | 10X | 1.2891 | 0.89 |
| *Callitrichia* | *legandi* | (Jocqué, 1985) | ♂ | Comoros, 1983 | RMCA paratype | 10X | 0.9148 | 0.58 |
| *Callitrichia* | *longiducta* | (Bosmans, 1988) | ♂ | Guinea, 1956 | SMF holotype | 20X | 1.1560 | 0.84 |
| *Callitrichia* | *macropthalma* | (Locket & Russell-Smith, 1980) | ♂ | Nigeria, 1973 | NHM holotype | 20X | 1.0789 | 0.51 |
| *Callitrichia* | *muscicola* | (Bosmans, 1988) | ♂ | Cameroon, 1983 | RMCA 165091 paratype | 20X | 1.1560 | 0.76 |
| *Callitrichia* | *picta* | (Caporiacco, 1949) | ♂ | Congo, 1967 | RMCA | 10X | 1.1435 | 0.68 |
| *Callitrichia* | *pilosa* | (Wunderlich, 1978) | ♂ | Ethiopia, 1977 | SMF 11353 holotype | -* | -* | 0.64 |
| *Callitrichia* | *sellafrontis* | Scharff, 1990 | ♂ | Tanzania, 1996 | ZMUC | 20X | 1.1097 | 0.70 |
| *Callitrichia* | *uncata* | (Jocqué and Scharff, 1986) | ♂ | Tanzania, no date | ZMUC | 10X | 1.0292 | 0.79 |
| *Callitrichia* | *usitata* | (Jocqué & Scharff, 1986) | ♂ | Tanzania, 1980 | RMCA 160.013 paratype | 20X | 1.0789 | 0.64 |
| *Cornitibia* | *simplicithorax* | (Tanasevitch, 1998) | ♂ | Nepal, 1988 | SMF 38861 holotype | -* | -* | 0.78 |
| *Diplocentria* | *bidentata* | (Emerton, 1882) | ♂ | Germany, 1958 | SMF | 10X | 1.0805 | 0.62 |
| *Emertongone* | *montifera* | (Emerton, 1882) | ♂ | USA, 1954 | AMNH | 20X | 0.9537 | 0.67 |
| *Erigone* | *atra* | Blackwall, 1833 | ♂ | Germany, 2014 | ZIMG | 4X | 1.7607 | 0.89 |
| *Gonatium* | *rubellum* | (Blackwall, 1841) | ♂ | Denmark 2010 | ZIMG SEQ-LL-01-43 | 10X | 1.5683 | 1.07 |
| *Gongylidiellum* | *vivum* | (O. Pickard-Cambridge, 1875) | ♂ | Denmark, 2010 | ZIMG SEQ-LL-01-46 | 20X | 1.0951 | 0.54 |
| *Gongylidium* | *rufipes* | (Linnaeus, 1758) | ♂ | Germany, 2014 | ZIMG | 10X | 1.4598 | 1.05 |
| *Holmelgonia* | *basalis* | (Jocqué & Scharff, 1986) | ♂ | Tanzania, 1982 | ZMUC | 20X | 0.8828 | 0.61 |
| *Hylyphantes* | *graminicola* | (Sundevall, 1830) | ♂ | Taiwan, 2014 | ZIMG | 10X | 1.4970 | 0.77 |
| *Jilinus* | *hulongensis* | (Zhu & Wen, 1980) | ♂ | China, 1985 | IZCAS-Ar. 385 | 20X | 1.0150 | 0.71 |
| *Linyphia* | *triangularis* | (Clerck, 1757) | ♂ | Denmark, 1994 | ZMUC 00011150 | 10X | 2.4215 | 1.38 |
| *Lophomma* | *punctatum* | (Blackwall, 1841) | ♂ | Greifswald, 2015 | ZIMG | 10X | 1.3069 | 0.80 |
| *Mitrager* | *angela* | (Tanasevitch, 1998) | ♂ | Nepal, 1988 | SMF 38853 paratype | 10X | 1.2556 | 0.73 |
| *Mitrager* | *assueta* | (Tanasevitch, 1998) | ♂ | Nepal, 1980 | SMF 38852 paratype | -* | -* | 0.77 |
| *Mitrager* | *clypeellum* | (Tanasevitch, 1998) | ♂ | Nepal, 1980 | SMF 38857 holotype | -* | -* | 0.87 |
| *Mitrager* | *cornuta* | (Tanasevitch, 2015) | ♂ | India, 1978 | MHNG holotype | 20X | 1.1406 | 0.70 |
| *Mitrager* | *coronata* | (Tanasevitch, 1998) | ♂ | Nepal, 1988 | SMF 38832 paratype | 10X | 1.6188 | 0.91 |
| *Mitrager* | *dismodicoides* | (Wunderlich, 1974) | ♂ | Nepal, 1969 | SMF 28902 holotype | 20X | 1.1324 | 0.71 |
| *Mitrager* | *elongata* | (Wunderlich, 1974) | ♂ | Nepal, 1973 | SMF 28894 paratype | 10X | 1.2823 | 0.87 |
| *Mitrager* | *falcifer* | (Tanasevitch, 1998) | ♂ | Nepal, 1988 | SMF 38856 holotype | -* | -* | 0.87 |
| *Mitrager* | *falciferoides* | (Tanasevitch, 2015) | ♂ | India, 1978 | MHNG holotype | 20X | 1.1291 | 0.67 |
| *Mitrager* | *globiceps* | (Thaler, 1987) | ♂ | India, 1976 | SMF 33832 holotype | 20X | 1.1324 | 0.70 |
| *Mitrager* | *hirsuta* | (Wunderlich, 1974) | ♂ | Nepal, 1973 | SMF 28892 holotype | -* | -* | 0.67 |
| *Mitrager* | *lineata* | (Wunderlich, 1974) | ♂ | Nepal, 1969 | SMF 28899 holotype | -* | -* | 0.70 |
| *Mitrager* | *lopchu* | (Tanasevitch, 2015) | ♂ | India, 1978 | MHNG paratype | 20X | 1.0671 | 0.75 |
| *Mitrager* | *lucida* | (Wunderlich, 1974) | ♂ | Nepal, 1973 | SMF 28898 paratype | 20X | 1.1406 | 0.67 |
| *Mitrager* | *malearmata* | (Tanasevitch, 1998) | ♂ | Nepal, 1988 | SMF 38843 holotype | 10X | 1.3126 | 0.71 |
| *Mitrager* | *modesta* | (Tanasevitch, 1998) | ♂ | Nepal, 1988 | SMF 38830 holotype | -* | -* | 0.82 |
| *Mitrager* | *rustica* | (Tanasevitch, 2015) | ♂ | India, 1972 | MHNG paratype | 10X | 1.1679 | 0.75 |
| *Mitrager* | *savigniformis* | (Tanasevitch, 1998) | ♂ | Nepal, 1988 | SMF 38834 holotype | ??? | ??? | 0.79 |
| *Mitrager* | *sexoculata* | (Wunderlich, 1974) | ♂ | Nepal, 1974 | SMF 28895 holotype | -* | -* | 0.73 |
| *Mitrager* | *sexoculorum* | (Tanasevitch, 1998) | ♂ | Nepal, 1983 | SMF 38842 holotype | -* | -* | 0.69 |
| *Mitrager* | *tholusa* | (Tanasevitch, 1998) | ♂ | Nepal, 1980 | SMF 38837 holotype | -* | -* | 0.96 |
| *Mitrager* | *unicolor* | (Wunderlich, 1974) | ♂ | Nepal, 1970 | SMF 28889 holotype | 10X | 1.1888 | 0.76 |
| *Mitrager* | *villosa* | (Tanasevitch, 2015) | ♂ | India, 1978 | MHNG paratype | 20X | 1.1560 | 0.78 |
| *Mitrager* | *noordami* | van Helsdingen, 1985 | ♂ | Indonesia, 1977 | paratype, Naturalis Museum, Leiden | 10X | 1.7122 | 0.77 |
| *Mitrager* | *noordami* | van Helsdingen, 1985 | ♀ | Indonesia, 1977 | paratype, Naturalis Museum, Leiden | 10X | 1.6188 | 0.84 |
| *Nasoona* | *crucifera* | (Thorell, 1895) | ♂ | Thailand, 1991 | ZMUC | 20X | 1.1034 | 0.64 |
| *Nasoona* | *setifera* | (Tanasevitch, 1998) | ♂ | Nepal, 1983 | SMF 38844-125 holotype | 10X | 1.5247 | 0.91 |
| *Oedothorax* | *agrestis* | (Blackwall, 1853) | ♂ | USA, 1958 | NHM | 10X | 1.1122 | 0.80 |
| *Oedothorax* | *apicatus* | (Blackwall, 1850) | ♂ | Germany, 2015 | ZIMG | 10X | 1.7605 | 0.73 |
| *Oedothorax* | *fuscus* | (Blackwall, 1834) | ♂ | Denmark, 2009 | ZMUC 00012872 | 10X | 1.3429 | 0.73 |
| *Oedothorax* | *gibbifer* | (Kulczyński, 1882) | ♂ | Locality & date unknown | SMF 59615 | -* | -* | 0.87 |
| *Oedothorax* | *gibbosus,*  *gibbosus* morph | (Blackwall, 1841) | ♂ | Denmark, 1997 | ZMUC 00008860 | 10X | 1.5993 | 0.79 |
| *Oedothorax* | *gibbosus,*  *tuberosus* morph | (Blackwall, 1841) | ♂ | Denmark, 1992 | ZMUC 00007790 | 20X | 1.1756 | 0.77 |
| *Oedothorax* | *gibbosus* | (Blackwall, 1841) | ♀ | Denmark, 1992 | ZMUC 00007790 | 20X | 1.2062 | 0.83 |
| *Oedothorax* | *meridionalis* | Tanasevitch, 1987 | ♂ | Georgia, 1987 | Tanasevitch personal collection | 10X | 1.3697 | 0.73 |
| *Oedothorax* | *paludigena* | Simon, 1926 | ♂ | France, 1984 | MHNG | 20X | 1.0200 | 0.86 |
| *Oedothorax* | *retusus* | (Westring, 1851) | ♂ | Germany, 2009-2012 | ZIMG | 10X | 1.2859 | 0.74 |
| *Oedothorax* | *tingitanus* | (Simon, 1884) | ♂ | Morocco, 1977 | NHM | 10X | 1.1280 | 0.68 |
| *Oedothorax* | *trilobatus* | (Banks, 1896) | ♂ | USA, 1934 | AMNH | 10X | 1.2452 | 0.79 |
| *“Oedothorax”* | *cunur* | Tanasevitch, 2015 | ♂ | India, 1972 | MHNG holotype | 10X | 1.1910 | 0.87 |
| *“Oedothorax”* | *kodaikanal* | Tanasevitch, 2015 | ♂ | India, 1972 | MHNG paratype | 10X | 1.4224 | 0.85 |
| *“Oedothorax”* | *meghalaya* | Tanasevitch, 2015 | ♂ | India, 1978 | MHNG paratype | 20X | 1.1560 | 0.75 |
| *“Oedothorax”* | *nazareti* | Scharff, 1989 | ♂ | Ethiopia, 1988 | RMCA 224.501 | 10X | 1.2529 | 0.74 |
| *“Oedothorax”* | *paracymbialis* | Tanasevitch, 2015 | ♂ | India, 1972 | MHNG holotype | 10X | 0.9464 | 0.63 |
| *“Oedothorax”* | *stylus* | Tanasevitch, 2015 | ♂ | India, 1972 | MHNG paratype | 20X | 1.1406 | 0.67 |
| *“Oedothorax”* | *uncus* | Tanasevitch, 2015 | ♂ | India, 1978 | MHNG holotype | 10X | 1.2846 | 0.80 |
| *Pimoa* | *altioculata* | (Keyserling, 1886) | ♂ | USA, 1987 | ZMUC | 4X | 4.3140 | 3.07 |
| *Shaanxinus* | *mingchihensis* | Lin, 2019 | ♂ | Taiwan: Yilan, 2014 | ZIMG-II-28488 | 10X | 1.7122 | 0.90 |
| *Stemonyphantes* | *lineatus* | (Linnaeus, 1758) | ♂ | Denmark, 2009 | ZMUC | 4X | 3.0814 | 1.97 |
| *Tmeticus* | *tolli* | Kulczyński, 1908 | ♂ | Russia, 1998 | ZMUC 00004649 | 10X | 1.7153 | 0.80 |
| *Ummeliata* | *esyunini* | (Zhang, Zhang & Yu, 2003) | ♂ | China, 1999 | Hebei University, College of life science, paratype | 10X | 1.2915 | 1.03 |
| *Ummaliata* | *insecticeps* | (Bösenberg & Strand, 1906) | ♂ | China, 2002 | IZCAS-Ar8916 paratype | 10X | 1.3722 | 0.93 |
| *Walckenaeria* | *acuminata* | Blackwall, 1833 | ♂ | Germany, 1960 | ZIMG II-28106 | 10X | 1.4680 | 0.97 |

* Missing data due to breakdown of storage device in the department

# Differences between Matrix II (Lin et al. 2021) and the current matrix

1. character scoring

In *Oedothorax meghalaya incertae sedis*, only two of the three palpal radical apophyses in the distal part of embolic division are present, and their primary homologies to two of the three structures cannot be recognized by topological relations or composition of structures; accordingly, the scoring of the anterior radical process (ARP, Chs. 16, 17), the lateral extension of radix (LER, Chs. 18-21) and the ventral radical process (VRP, Chs. 22, 23) was changed to inapplicable. In *Oedothorax kodaikanal* *incertae sedis,* the structure interpreted as the palpal tibial prolateral spike (TPS) in Lin et al. (2021) is seemingly not homologous to TPS, as it is much smaller and located on a different position when compared to the TPS in other species; therefore, the scoring of characters 52 and 53 were also changed to inapplicable. In the tree resulting from the phylogenetic analysis, for *O. meghalaya incertae sedis*, the plesiomorphic character states of all three distal apophyses on the embolic division are present, thus the homologies of the two present apophyses in this species remained unresolved; The plesiomorphic state of the TPS of *O. kodaikanal incertae sedis* is absent, which supports the interpretation of the small elevation at the base of the palpal prolateral apophysis in this species not as the TPS, but rather a different structure.

1. Inferred numbers of individual origins of prosomal structures

Due to the change in the definition and number of characters and thus the tree topology, the numbers of origins of some features differ from the previous study. These include the pre-PME groove (three origins; two origins and one loss in Lin et al. 2021), the newly defined post-DP groove (four origins and one loss; post-PME groove had five origins and one loss in Lin et al. 2021) and post-PME lobe (seven to eight origins and one to zero losses, according to fast or slow optimization, respectively; nine to ten origins and one to zero losses in Lin et al. 2021).

# Muscles related to chelicerae and palp bases

The position of the cheliceral muscles was found in the described specimens as described in Palmgren (1978, 1980) and Wood and Parkinson (2019). The endosternite muscles are positioned ventrally, distant to the dorsal surface where the epidermal gustatory glands are located, and are therefore not addressed in the present study. Abbreviations of the names of muscles and the colors used in figures are given in Table 1. The “IC” is much smaller in all species in the present study than in the palpimanoid species depicted in Wood and Parkinson (2019). Since the shape of the attachment area of the inter-cheliceral-sclerite muscle according to Wood and Parkinson (2019) is similar to the combined attachment area of three muscles in the current study - the IC, the anterior pharyngeal dilator and the posterior pharyngeal dilator – we assume that the distinction was not made in Wood and Parkinson (2019). The latter two muscles, however, are not cheliceral muscles. Furthermore, the inter-cheliceral-sclerite muscle which Wood and Parkinson (2019) regarded as not observed in Palmgren (1978) was present in Palmgren (1978) and Palmgren (1980) under the name “m.medialis retro-descendens (rd)”; the anterior outer muscle (AO) was regarded by them as not observed in Palmgren (1978), but it is most likely equivalent to the “lateralis anterior β (la β)” in Palmgren (1980), fig. 13, while the lateral anterior muscle (LA) is equivalent to “la α”.

# Tissue shrinkage and its effect on muscles and glands

In specimens with apparent tissue shrinkage manifested by the gap between the cuticle and internal tissues, most parts of the gustatory glandular tissue remain attached to the cuticle of the dorsal part of the prosoma, while the muscles are detached from the dorsal part of the prosoma but remain attached to the chelicerae and pharynx (Figs. 4I, 5D, F, G, I, 6B, C, E, 7A, C, F, G, I, J, 8A, D, E, H, K, L, 9D, E, K, 3H, L). In one case the gustatory gland was also separated from the cuticle, probably with the entire epithelium (Fig. 4J).

Supplementary table 2. Character matrix for the newly scored characters, including consistency index and retention index (the complete matrix has been submitted to TreeBASE, submission ID 28232).

|  | 129 pre-PME groove connection: non; IC; IC & DA | 130 prosomal gland: abs; pres. | 131 clypeal gland: abs; pres. | 132 interocular gland: abs; pres. |  | 133 DA-DP gland: abs; pres. | 134 post-DP gland: abs; pres. | 135 cheliceral gland: abs; pres. |  | 129 pre-PME groove connection: non; IC; IC & DA | 130 prosomal gland: abs; pres. | 131 clypeal gland: abs; pres. | 132 interocular gland: abs; pres. | 133 DA-DP gland: abs; pres. | 134 post-DP gland: abs; pres. | 135 cheliceral gland: abs; pres. |
| --- | --- | --- | --- | --- | --- | --- | --- | --- | --- | --- | --- | --- | --- | --- | --- | --- |
| *Pimoa altioculata* | - | 0 | - | - |  | - | - | - | *Mitrager hirsuta* | - | 1 | 1 | 1 | 0 | 0 | 0 |
| *Stemonyphantes lineatus* | - | 0 | - | - |  | - | - | - | *Mitrager lineata* | 0 | 1 | 1 | 1 | 1 | 0 | 0 |
| *Linyphia triangularis* | - | 1 | 0 | 1 |  | 0 | 0 | 0 | *Mitrager lucida* | 1 | 1 | 1 | 1 | 1 | 1 | 0 |
| *Gongylidiellum vivum* | - | 1 | 0 | 1 |  | 0 | 0 | 0 | *Mitrager malearmata* | - | 1 | 0 | 1 | 1 | 0 | 0 |
| *Erigone atra* | - | 0 | - | - |  | - | - | - | *Mitrager modesta* | - | 1 | 1 | 1 | 1 | 0 | 0 |
| *Diplocentria bidentata* | - | 0 | - | - |  | - | - | - | *Mitrager savigniformis* | - | 1 | 1 | 1 | 1 | 0 | 0 |
| *Gongylidium rufipes* | - | 0 | - | - |  | - | - | - | *Mitrager sexoculata* | 1 | 1 | 1 | 1 | 1 | 1 | 0 |
| *Hylyphantes graminicola* | - | 0 | - | - |  | - | - | - | *Mitrager sexoculorum* | 2 | 1 | 1 | 1 | 1 | 0 | 0 |
| *Tmeticus tolli* | - | 1 | 0 | 1 |  | 0 | 0 | 0 | *Cornitibia simplicithorax* | - | 1 | 0 | 1 | 0 | 0 | 0 |
| *Lophomma punctatum* | - | 1 | 0 | 1 |  | 0 | 0 | 0 | *Mitrager tholusa* | 0 | 1 | 1 | 1 | 1 | 0 | 0 |
| *Walckenaeria acuminata* | - | 1 | 1 | 1 |  | 0 | 0 | 0 | *Mitrager unicolor* | - | 1 | 0 | 1 | 1 | 0 | 0 |
| *Gonatium rubellum* | - | 0 | - | - |  | - | - | - | *Callitrichia sellafrontis* | 0 | 1 | 1 | 1 | 0 | 0 | 0 |
| *Araeoncus humilis* | - | 1 | 1 | 1 |  | 0 | 0 | 0 | *Callitrichia holmi* | - | 1 | 0 | 1 | 1 | 0 | 0 |
| *Ummeliata insecticeps* | - | 1 | 1 | 1 |  | 1 | 1 | 0 | *Callitrichia juguma* | 0 | 1 | 1 | 1 | 0 | 0 | 0 |
| *Oedothorax agrestis* | - | 1 | 0 | 1 |  | 1 | 0 | 0 | *Callitrichia uncata* | 0 | 1 | 1 | 1 | 0 | 0 | 0 |
| *Oedothorax apicatus* | - | 1 | 0 | 0 |  | 1 | 1 | 0 | *Holmelgonia basalis* | - | 0 | - | - | - | - | - |
| *Oedothorax fuscus* | - | 1 | 1 | 1 |  | 1 | 0 | 0 | *Callitrichia gloriosa* | 0 | 1 | 1 | 1 | 1 | 0 | 0 |
| *Oedothorax gibbifer* | - | 1 | 0 | 0 |  | 1 | 1 | 0 | *Callitrichia picta* | - | 1 | 1 | 1 | 1 | 0 | 0 |
| *Oedothorax gibbosus* | - | 1 | 0 | 0 |  | 1 | 1 | 0 | *Atypena cirrifrons* | - | 1 | 1 | 1 | 1 | 0 | 0 |
| *Callitrichia latitibialis* | - | 1 | 0 | 1 |  | 0 | 0 | 0 | *Atypena formosana* | - | 1 | 1 | 1 | 1 | 1 | 0 |
| *Callitrichia legrandi* | - | 1 | 0 | 1 |  | 0 | 0 | 0 | *Nasoona setifera* | - | 1 | 1 | 1 | 1 | 1 | 0 |
| *Callitrichia macropthalma* | - | 1 | 0 | 1 |  | 0 | 0 | 0 | *Nasoona crucifera* | - | 1 | 1 | 1 | 1 | 1 | 0 |
| *Oedothorax meridionalis* | - | 1 | 1 | 1 |  | 1 | 0 | 0 | *Shaanxinus mingchihensis* | - | 1 | 1 | 1 | 0 | 0 | 0 |
| *Callitrichia muscicola* | - | 1 | 0 | 1 |  | 0 | 0 | 0 | *Mitrager noordami* | - | 1 | 1 | 1 | 1 | 1 | 0 |
| *Oedothorax nazareti* | - | 1 | 1 | 1 |  | 1 | 0 | 0 | *Mitrager cornuta* | - | 1 | 1 | 1 | 1 | 1 | 0 |
| *Oedothorax paludigena* | - | 1 | 0 | 1 |  | 1 | 0 | 0 | *Mitrager lopchu* | - | 1 | 1 | 1 | 1 | 0 | 0 |
| *Oedothorax retusus* | - | 1 | 0 | 0 |  | 1 | 1 | 0 | *Oedothorax meghalaya* | - | 1 | 1 | 1 | 1 | 0 | 0 |
| *Oedothorax tingitanus* | - | 1 | 1 | 1 |  | 1 | 0 | 0 | *Mitrager rustica* | - | 1 | 0 | 1 | 1 | 0 | 0 |
| *Oedothorax trilobatus* | - | 1 | 0 | 1 |  | 1 | 1 | 0 | *Mitrager villosa* | - | 1 | 1 | 1 | 1 | 1 | 0 |
| *Callitrichia usitata* | - | 0 | - | - |  | - | - | - | *Ummeliata esyunini* | - | 1 | 0 | 0 | 0 | 1 | 0 |
| *Callitrichia pilosa* | - | 1 | 0 | 1 |  | 1 | 0 | 0 | *Callitrichia convector* | 0 | 1 | 1 | 1 | 1 | 0 | 0 |
| *Callitrichia longiducta* | - | 1 | 0 | 1 |  | 0 | 0 | 0 | *Oedothorax kodaikanal* | - | 1 | 1 | 1 | 0 | 0 | 0 |
| *Mitrager angela* | - | 1 | 1 | 1 |  | 1 | 1 | 0 | *Oedothorax cunur* | - | 0 | - | - | - | - | - |
| *Mitrager assueta* | - | 1 | 0 | 1 |  | 1 | 0 | 0 | *Oedothorax uncus* | - | 1 | 0 | 1 | 0 | 0 | 0 |
| *Mitrager clypeellum* | - | 1 | 1 | 1 |  | 0 | 0 | 1 | *Mitrager falciferoides* | - | 1 | 1 | 1 | 1 | 0 | 0 |
| *Mitrager coronata* | - | 1 | 1 | 1 |  | 1 | 1 | 0 | *Oedothorax stylus* | - | 1 | 0 | 1 | 0 | 0 | 0 |
| *Mitrager dismodicoides* | 0 | 1 | 1 | 1 |  | 1 | 0 | 0 | *Oedothorax paracymbialis* | - | 1 | 0 | 1 | 1 | 0 | 0 |
| *Mitrager elongata* | - | 1 | 1 | 1 |  | 0 | 0 | 1 | *Jilinus hulongensis* | - | 1 | 0 | 1 | 0 | 0 | 0 |
| *Mitrager falcifer* | - | 1 | 1 | 1 |  | 1 | 0 | 0 | *Emertongone montifera* | - | 1 | 1 | 1 | 1 | 0 | 0 |
| *Mitrager globiceps* | 2 | 1 | 1 | 1 |  | 1 | 0 | 0 |  |  |  |  |  |  |  |  |
| CI | 0.66 | 0.11 | 0.10 | 0.33 |  | 0.12 | 0.16 | 1 |  |  |  |  |  |  |  |  |
| RI | 0.50 | 0.11 | 0.66 | 0.50 |  | 0.69 | 0.68 | 1 |  |  |  |  |  |  |  |  |
