# Supplementary figures and images for: Diversification through gustatory courtship: an X-ray micro-computed tomography study on dwarf spiders
Source: Front Zool. 2021 Sep 28;18:51. doi: 10.1186/s12983-021-00435-8 (PMC8480068; doi:10.1186/s12983-021-00435-8)

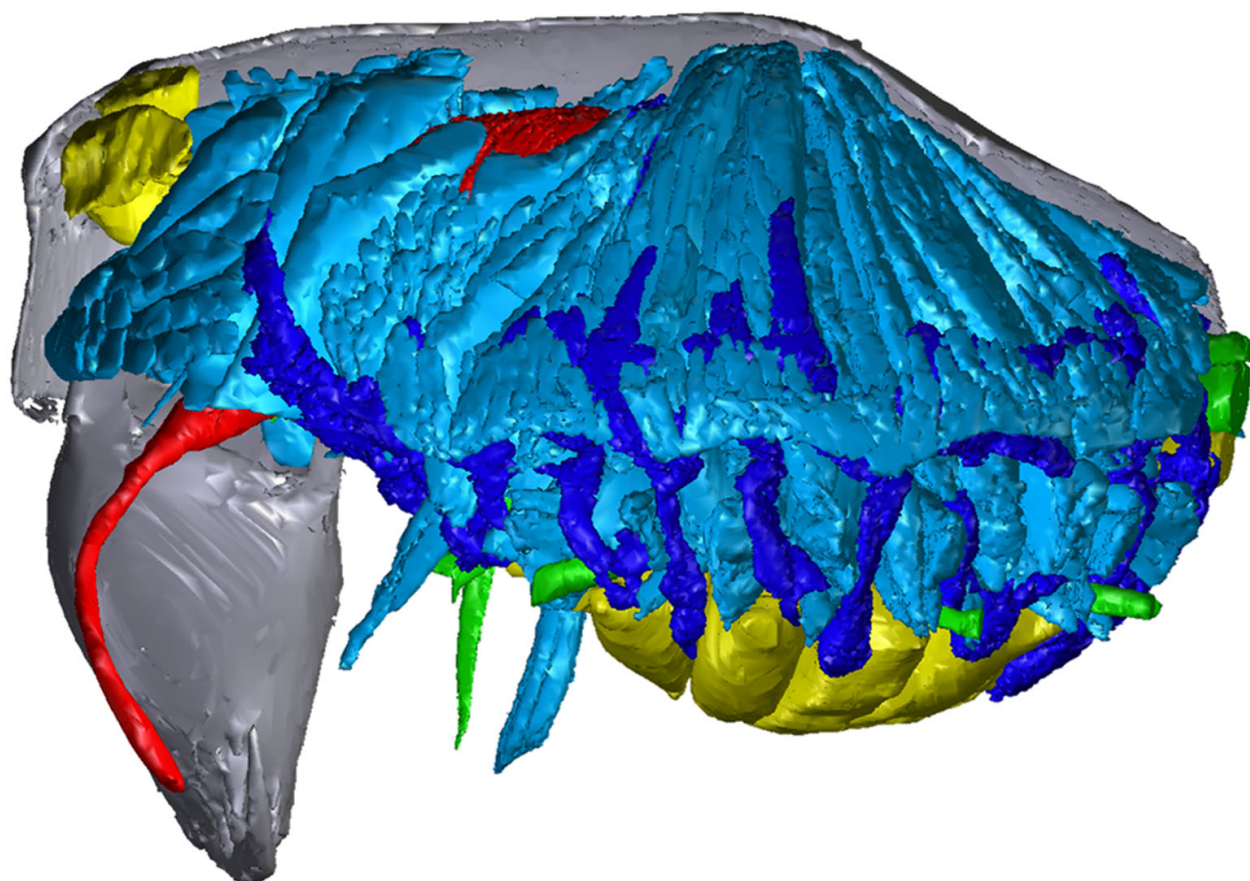

Additional file 2. Interactive 3D image of *Oedothorax gibbosus* female prosoma (Fig. 1A).

Supplement: Supplementary file 3 — Additional file 3. Interactive 3D images of Figs. 1A, C, E. [file 12983_2021_435_MOESM3_ESM.zip › 12983_2021_435_MOESM2_ESM/Additional file 2.pdf]

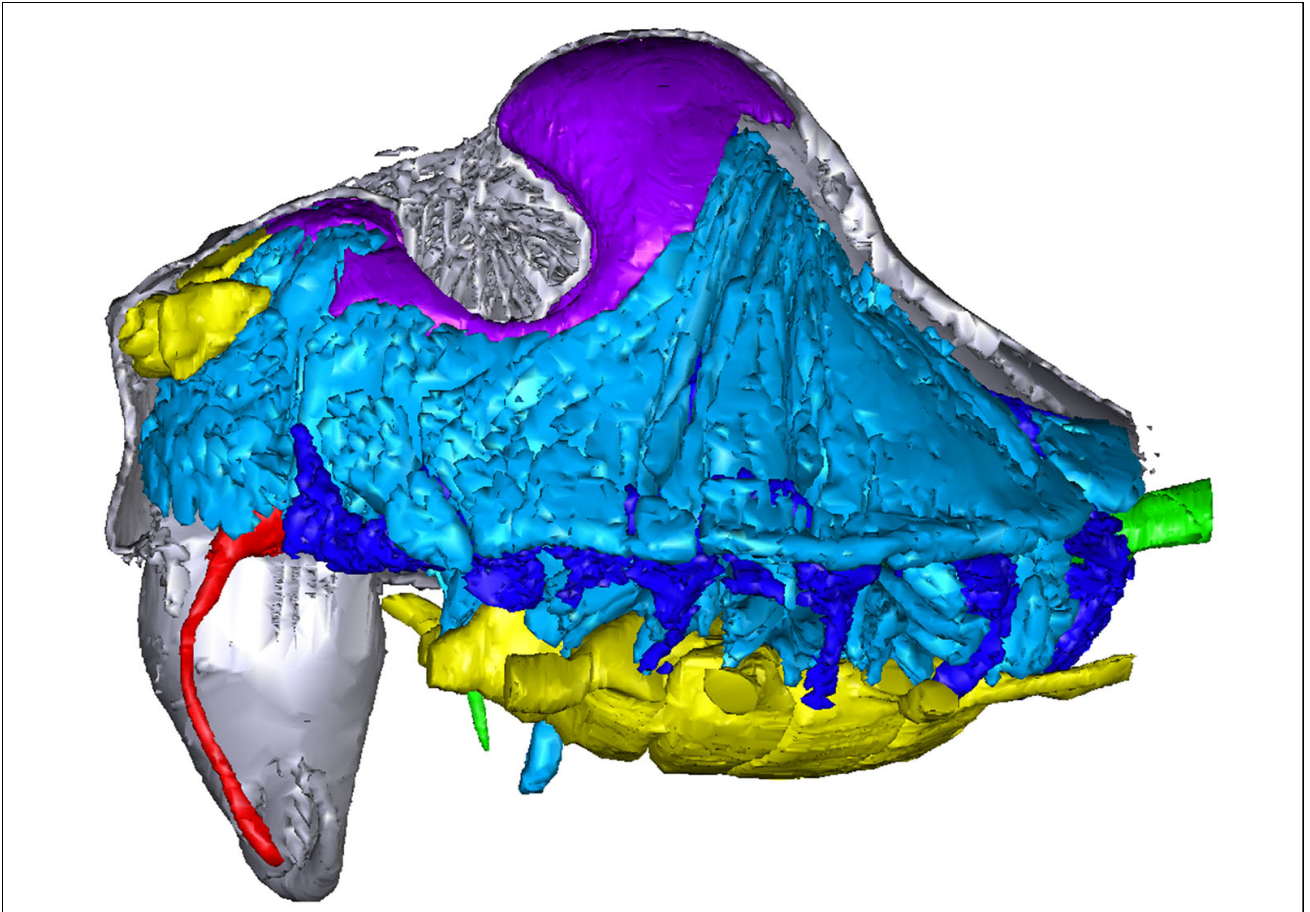

Additional file 3. Interactive 3D image of *Oedothorax gibbosus* male prosoma, *gibbosus* morph (Fig. 1C).

Supplement: Supplementary file 3 — Additional file 3. Interactive 3D images of Figs. 1A, C, E. [file 12983_2021_435_MOESM3_ESM.zip › 12983_2021_435_MOESM2_ESM/Additional file 3.pdf]

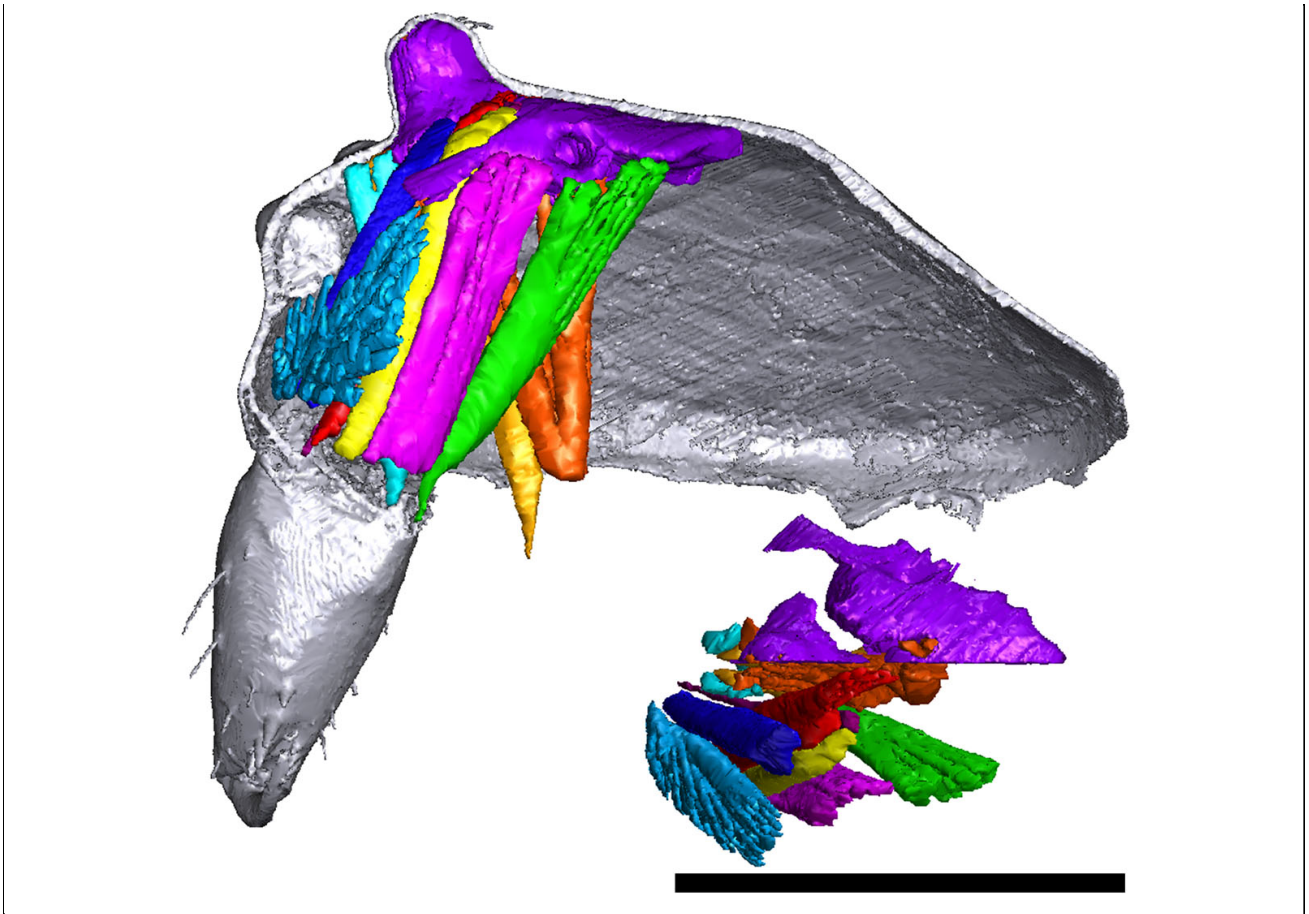

Additional file 10. Interactive 3D image of *Oedothorax apicatus* male prosoma (Fig. 2F).

Supplement: Supplementary file 4 — Additional file 4. Interactive 3D images of Figs. 2A-L. [file 12983_2021_435_MOESM4_ESM.zip › 12983_2021_435_MOESM3_ESM/Additional file 10.pdf]

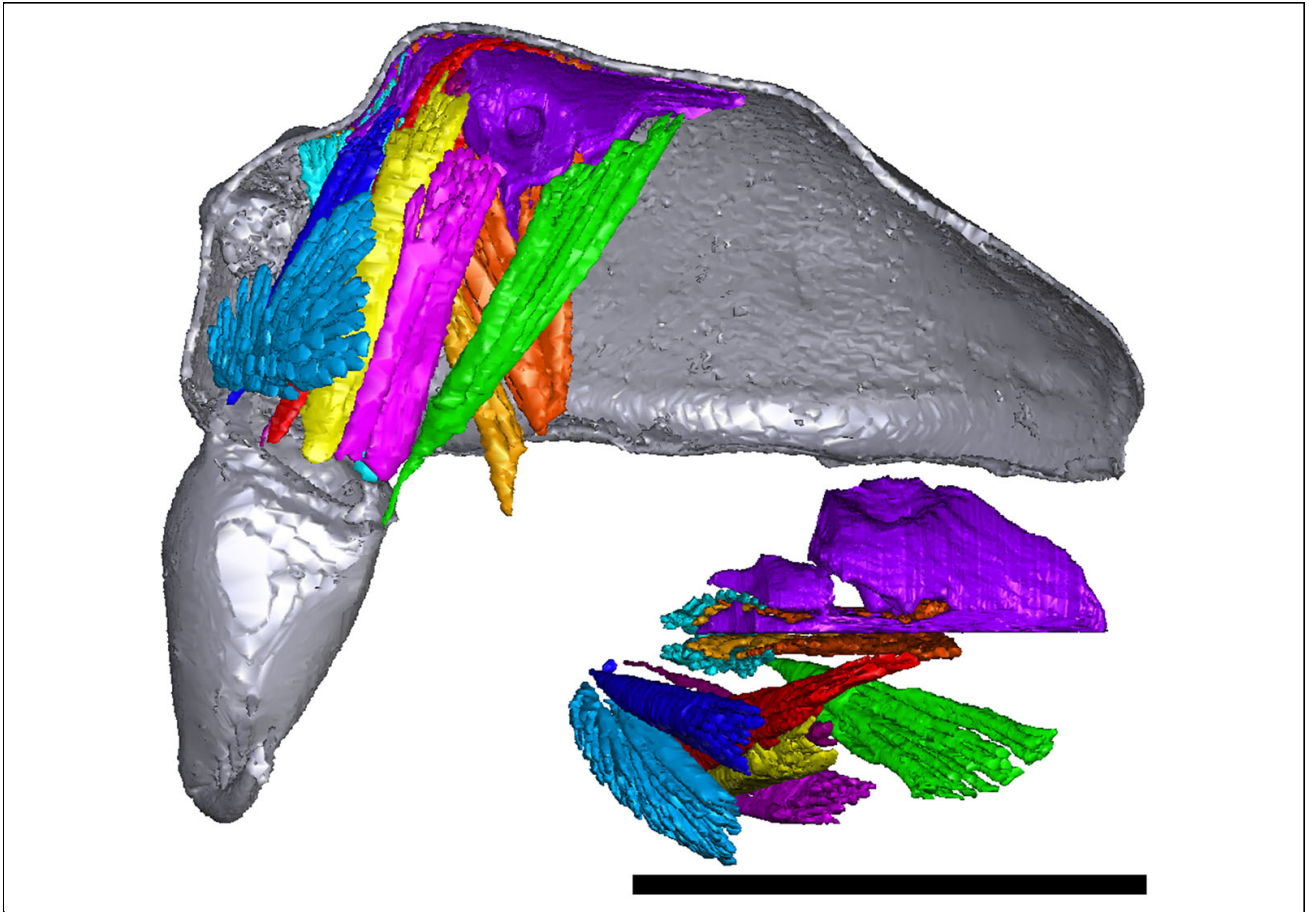

Additional file 11. Interactive 3D image of *Oedothorax retusus* male prosoma (Fig. 2G).

Supplement: Supplementary file 4 — Additional file 4. Interactive 3D images of Figs. 2A-L. [file 12983_2021_435_MOESM4_ESM.zip › 12983_2021_435_MOESM3_ESM/Additional file 11.pdf]

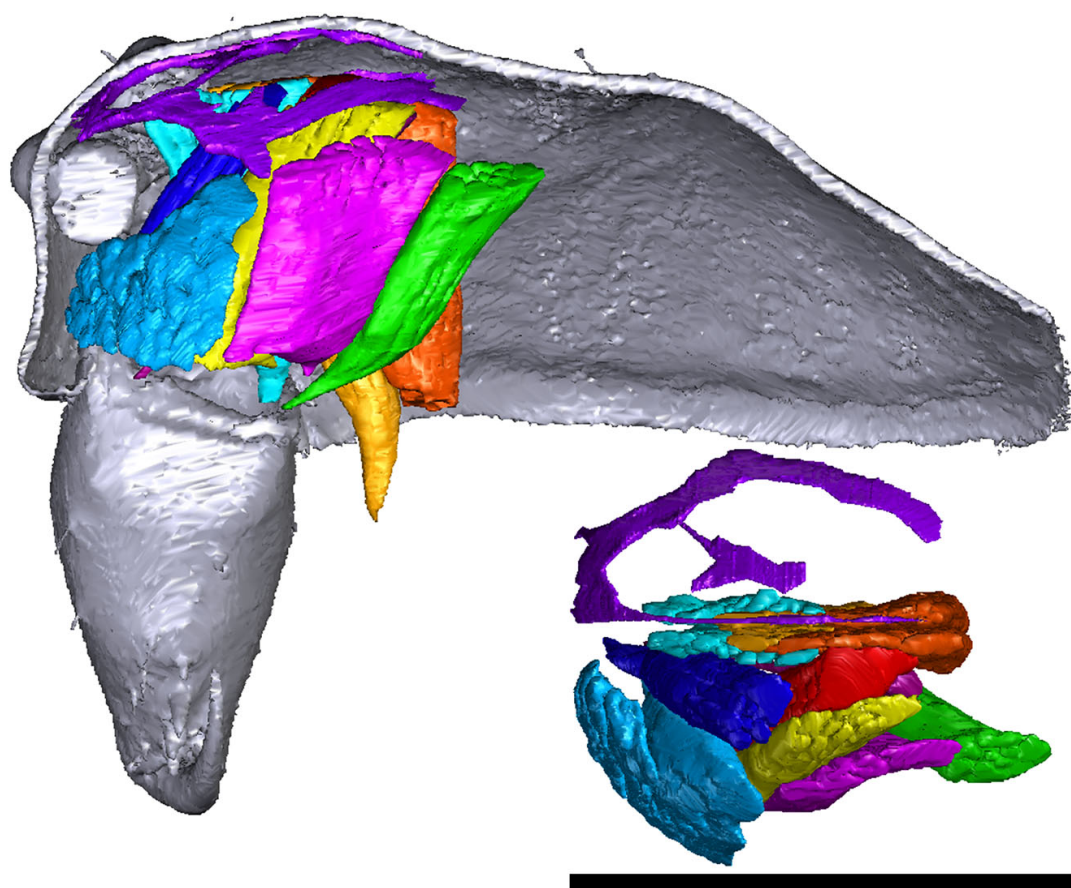

Additional file 12. Interactive 3D image of *Oedothorax paludigena* male prosoma (Fig. 2H).

Supplement: Supplementary file 4 — Additional file 4. Interactive 3D images of Figs. 2A-L. [file 12983_2021_435_MOESM4_ESM.zip › 12983_2021_435_MOESM3_ESM/Additional file 12.pdf]

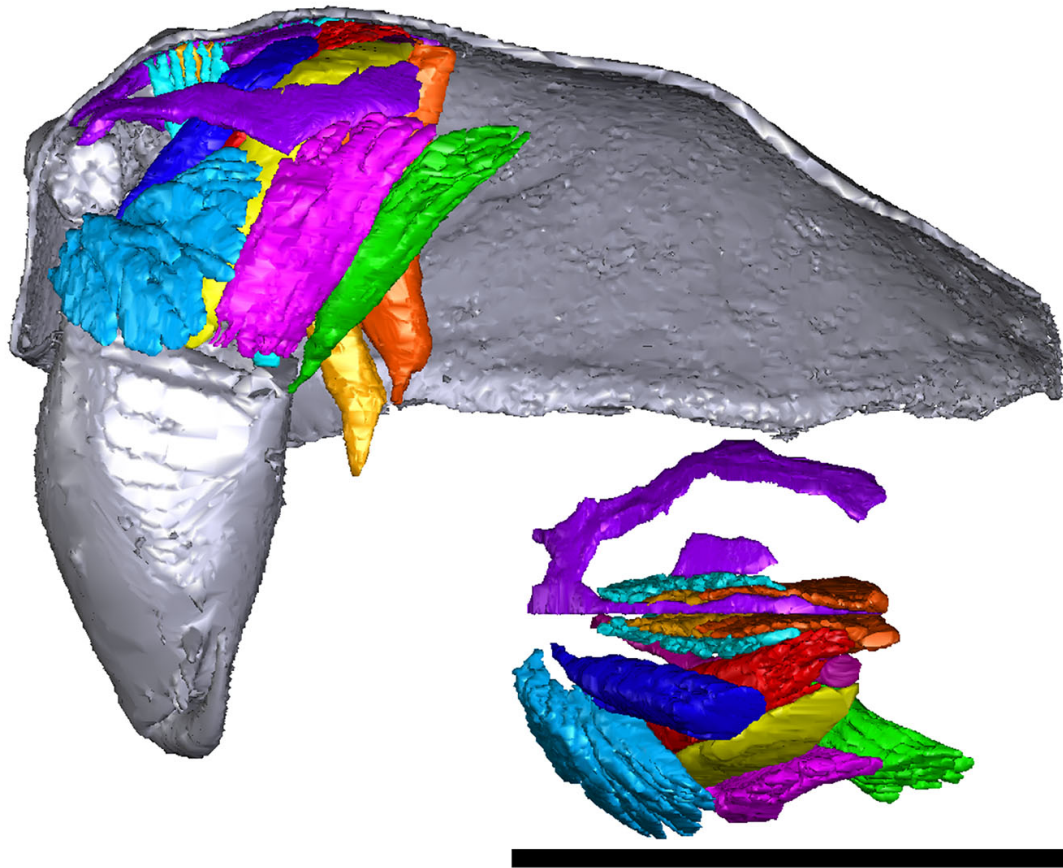

Additional file 13. Interactive 3D image of *Oedothorax agrestis* male prosoma (Fig. 2I).

Supplement: Supplementary file 4 — Additional file 4. Interactive 3D images of Figs. 2A-L. [file 12983_2021_435_MOESM4_ESM.zip › 12983_2021_435_MOESM3_ESM/Additional file 13.pdf]

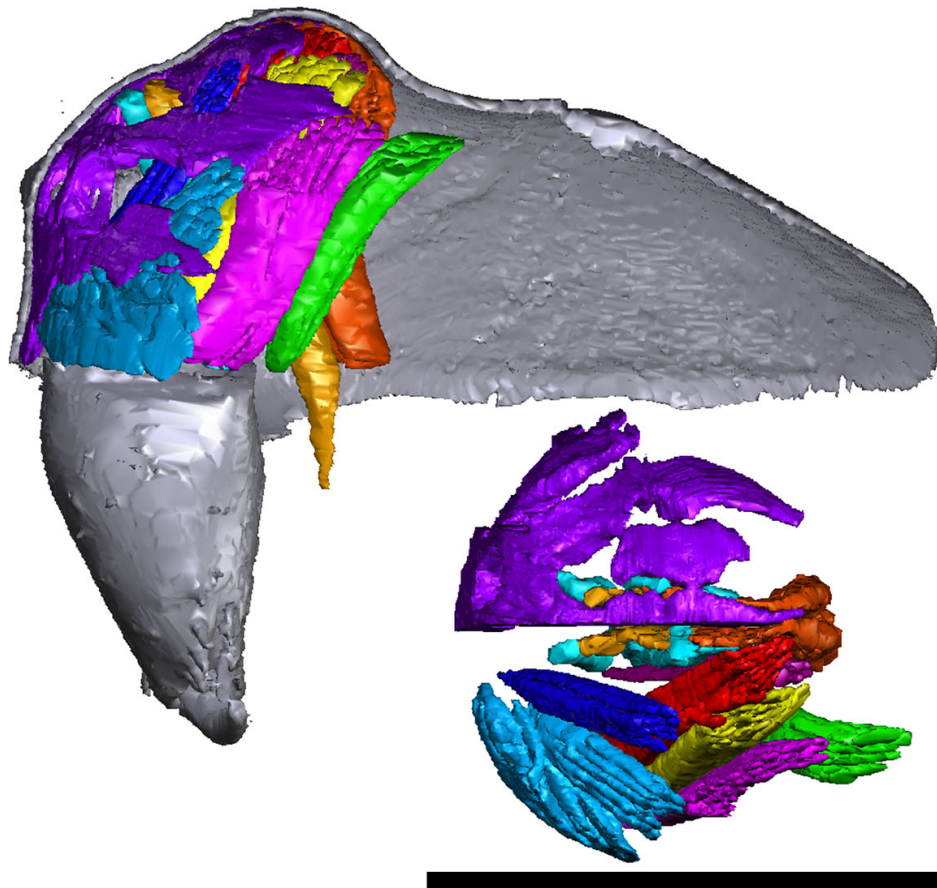

Additional file 14. Interactive 3D image of *Oedothorax meridionalis* male prosoma (Fig. 2J).

Supplement: Supplementary file 4 — Additional file 4. Interactive 3D images of Figs. 2A-L. [file 12983_2021_435_MOESM4_ESM.zip › 12983_2021_435_MOESM3_ESM/Additional file 14.pdf]

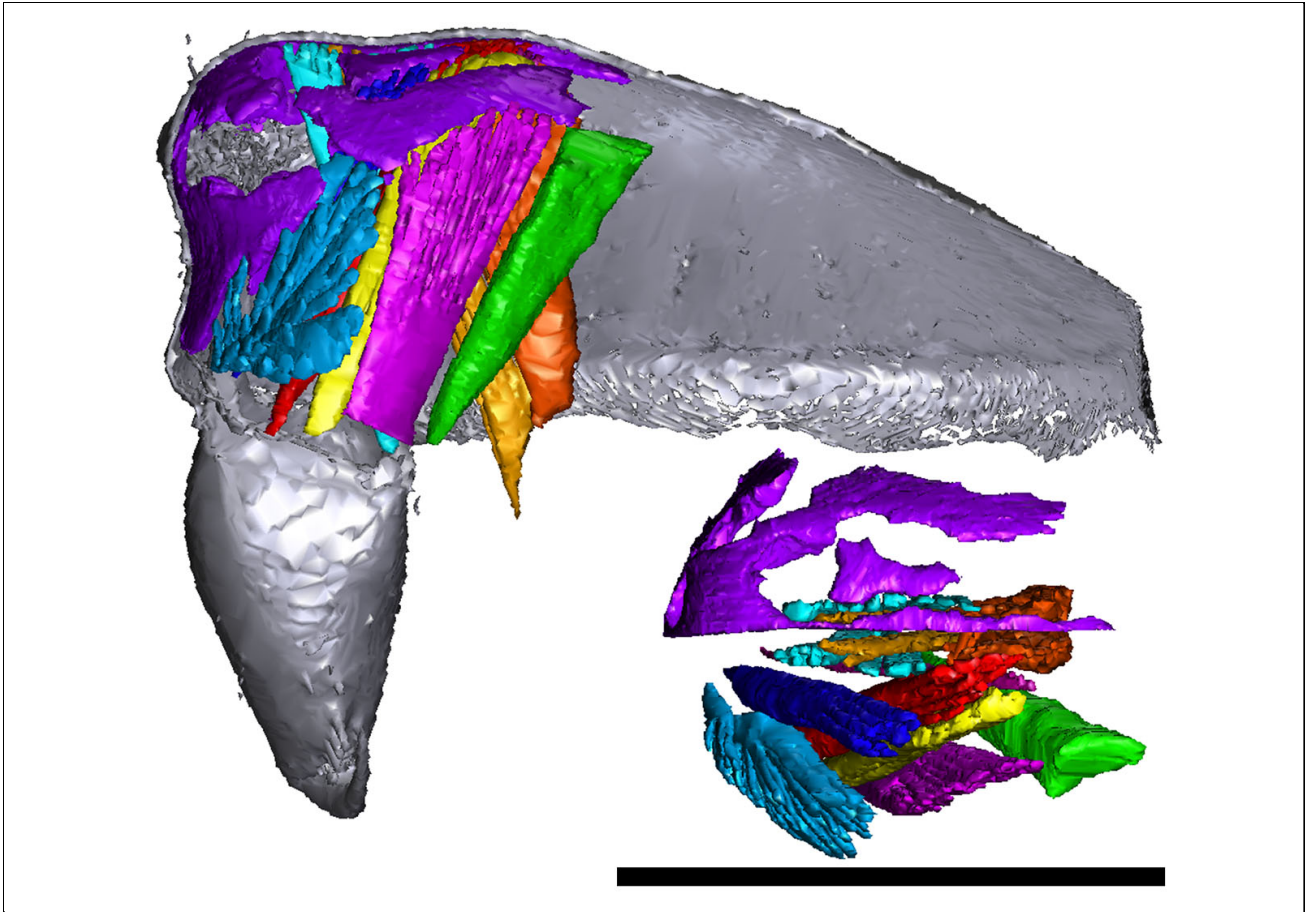

Additional file 15. Interactive 3D image of *Oedothorax fuscus* male prosoma (Fig. 2K).

Supplement: Supplementary file 4 — Additional file 4. Interactive 3D images of Figs. 2A-L. [file 12983_2021_435_MOESM4_ESM.zip › 12983_2021_435_MOESM3_ESM/Additional file 15.pdf]

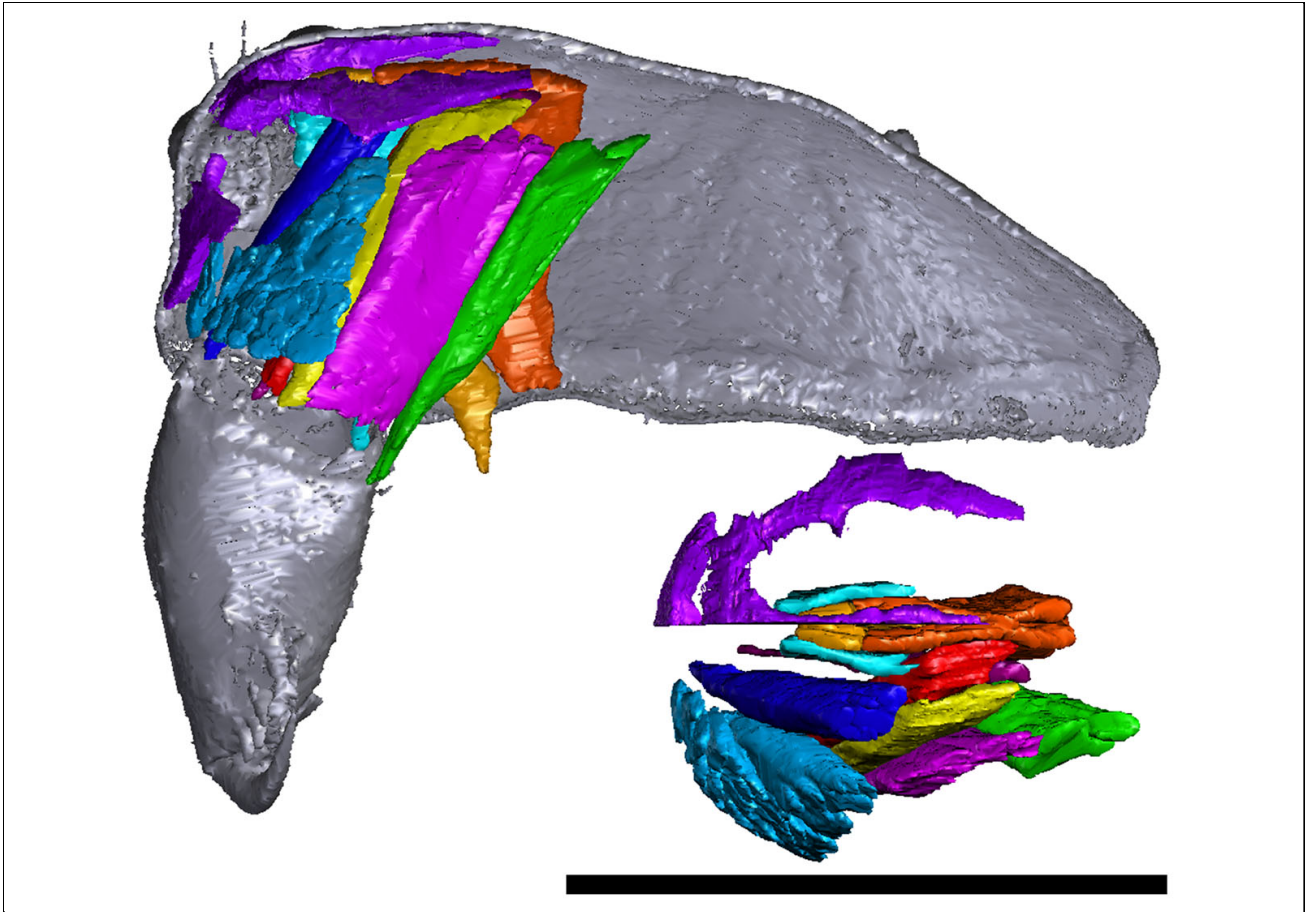

Additional file 16. Interactive 3D image of *Oedothorax tingitanus* male prosoma (Fig. 2L).

Supplement: Supplementary file 4 — Additional file 4. Interactive 3D images of Figs. 2A-L. [file 12983_2021_435_MOESM4_ESM.zip › 12983_2021_435_MOESM3_ESM/Additional file 16.pdf]

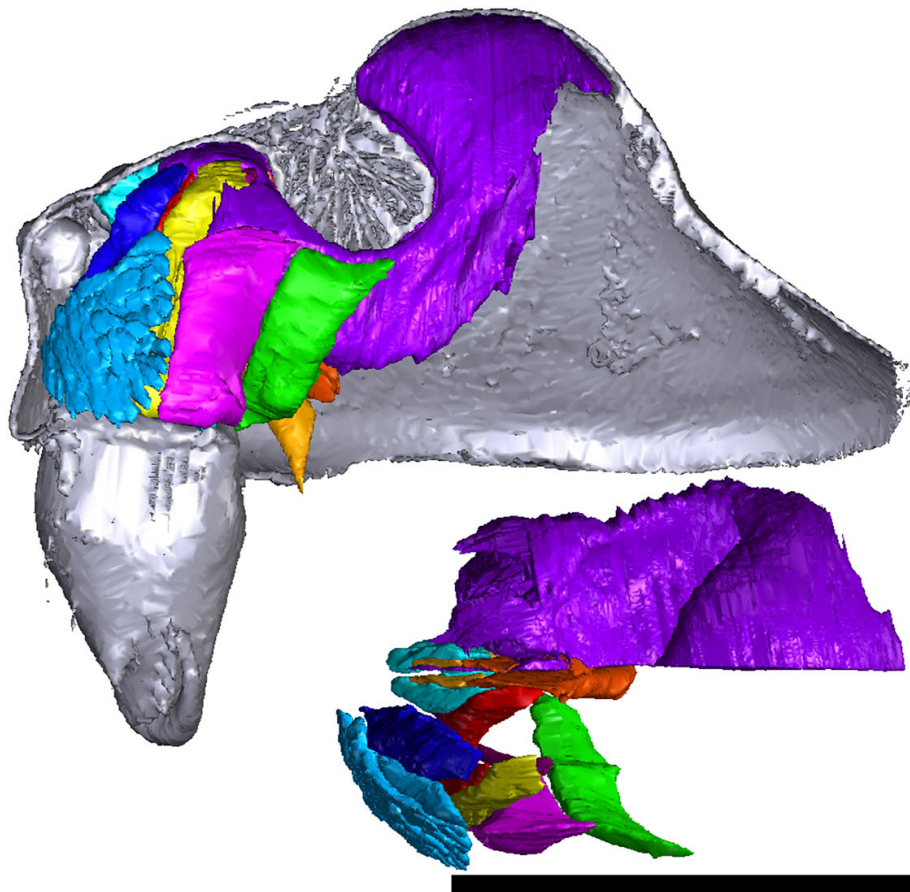

Additional file 5. Interactive 3D image of *Oedothorax gibbosus* male prosoma, *gibbosus* morph (Fig. 2A).

Supplement: Supplementary file 4 — Additional file 4. Interactive 3D images of Figs. 2A-L. [file 12983_2021_435_MOESM4_ESM.zip › 12983_2021_435_MOESM3_ESM/Additional file 5.pdf]

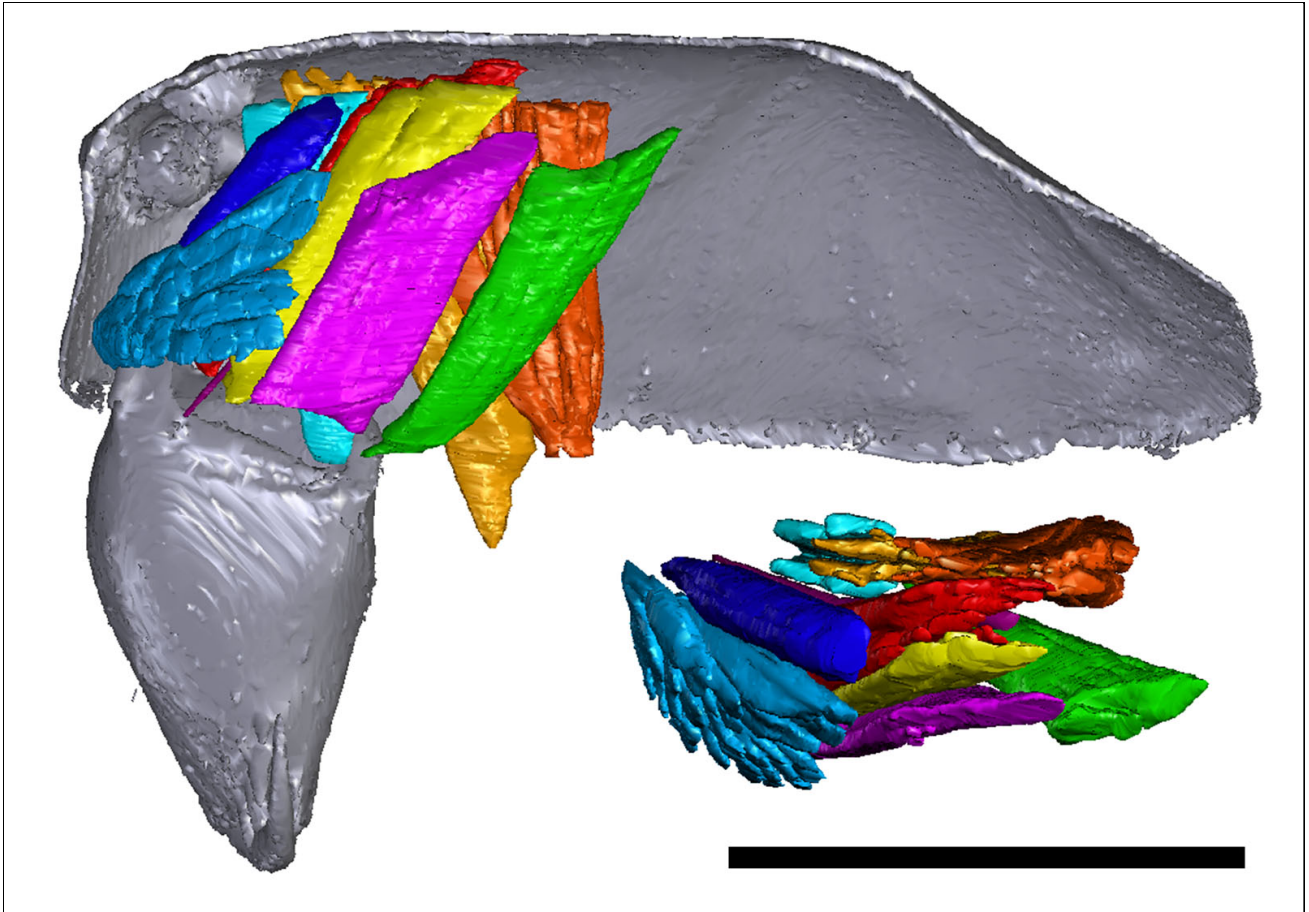

Additional file 7. Interactive 3D image of *Oedothorax gibbosus* female prosoma (Fig. 2C).

Supplement: Supplementary file 4 — Additional file 4. Interactive 3D images of Figs. 2A-L. [file 12983_2021_435_MOESM4_ESM.zip › 12983_2021_435_MOESM3_ESM/Additional file 7.pdf]

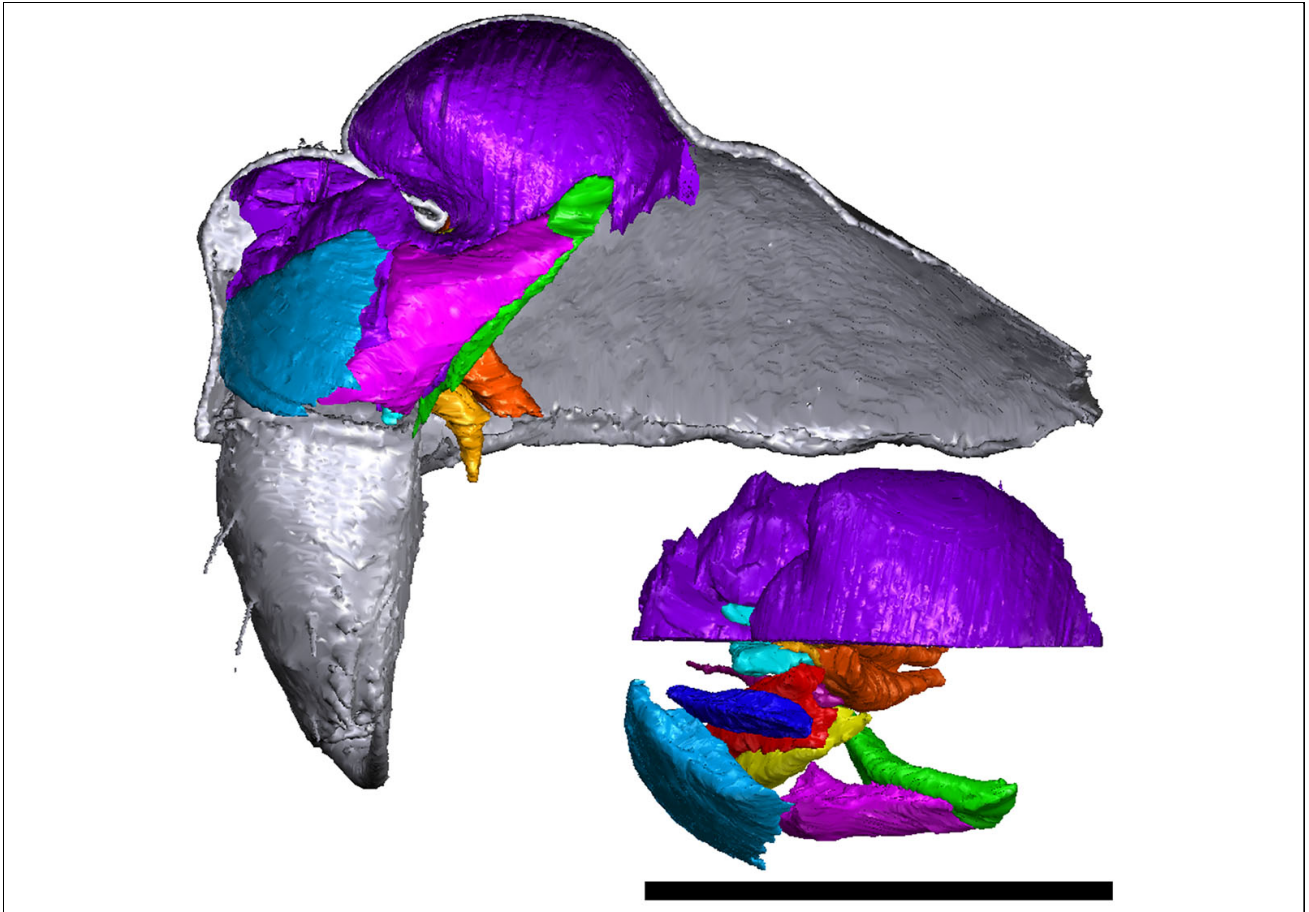

Additional file 8. Interactive 3D image of *Oedothorax trilobatus* male prosoma (Fig. 2D).

Supplement: Supplementary file 4 — Additional file 4. Interactive 3D images of Figs. 2A-L. [file 12983_2021_435_MOESM4_ESM.zip › 12983_2021_435_MOESM3_ESM/Additional file 8.pdf]

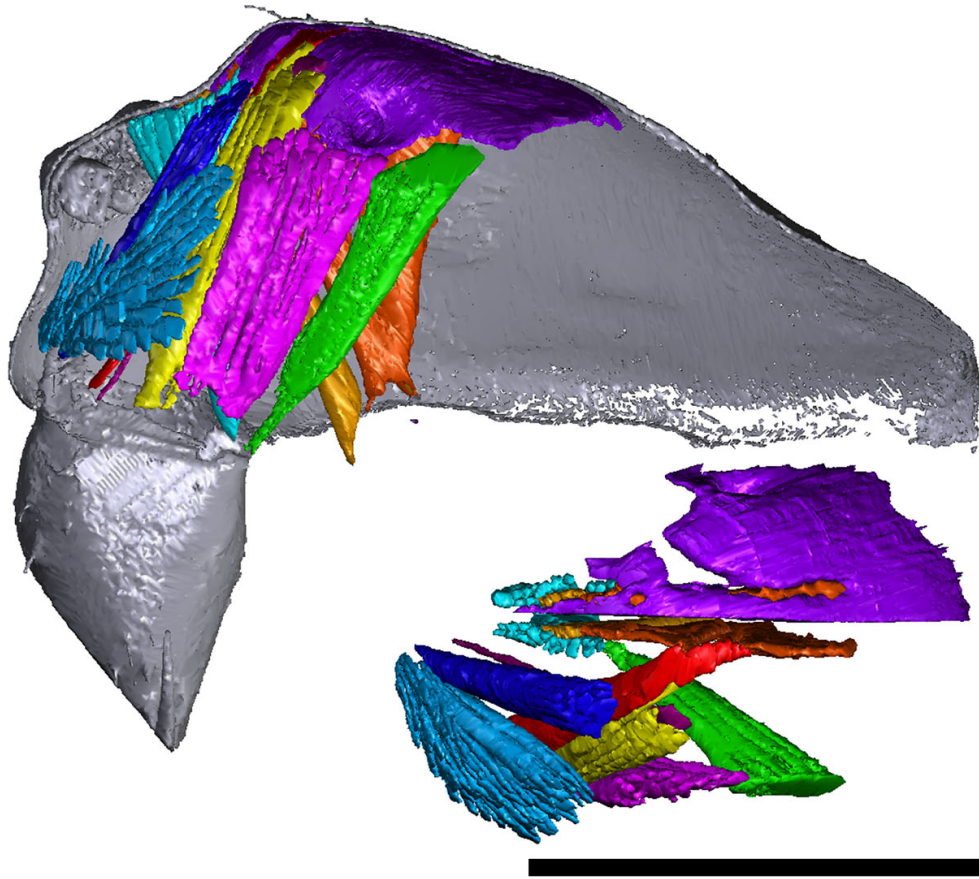

Additional file 9. Interactive 3D image of *Oedothorax gibbifer* male prosoma (Fig. 2E).

Supplement: Supplementary file 4 — Additional file 4. Interactive 3D images of Figs. 2A-L. [file 12983_2021_435_MOESM4_ESM.zip › 12983_2021_435_MOESM3_ESM/Additional file 9.pdf]

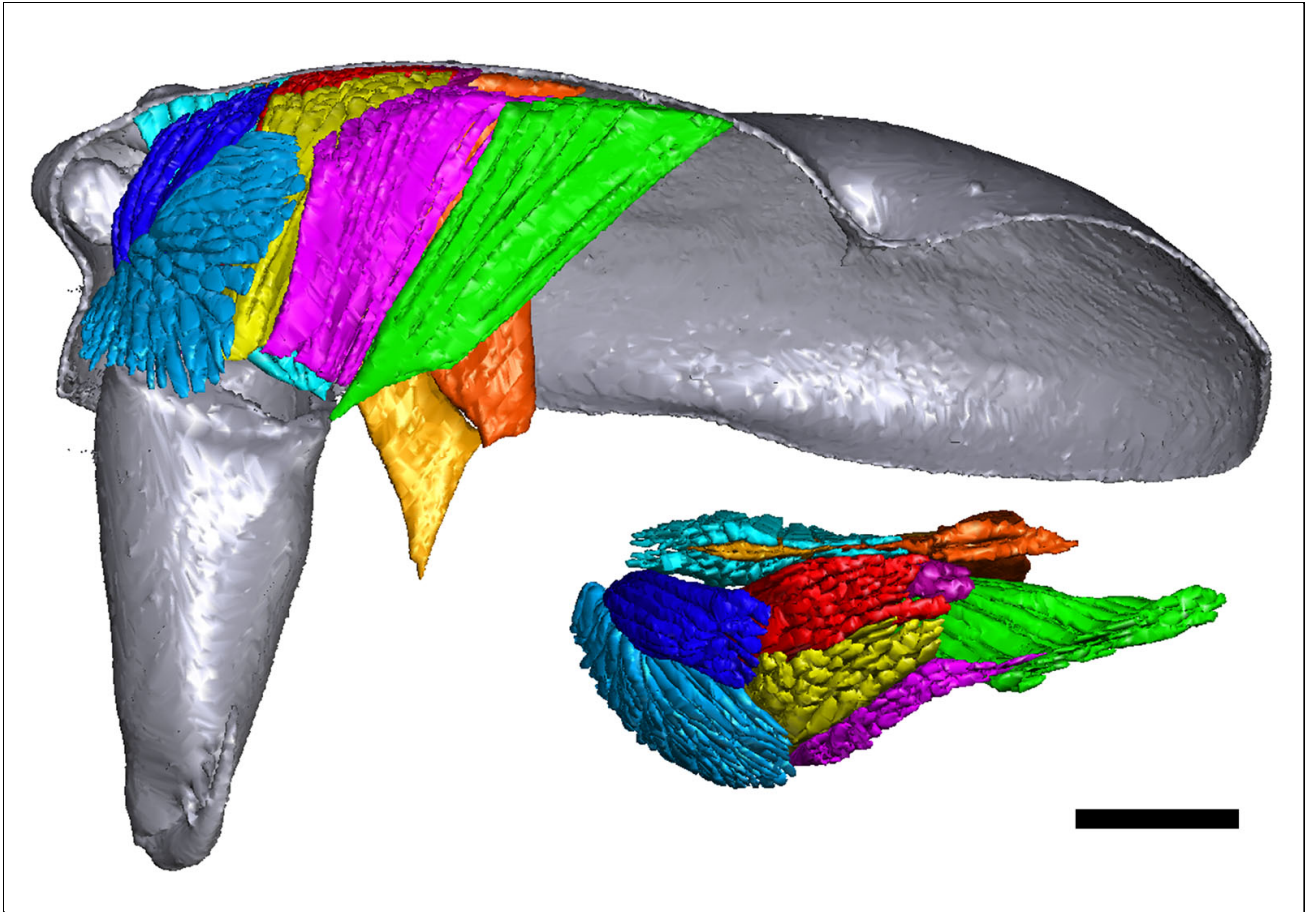

Additional file 17. Interactive 3D image of *Pimoid autiocularata* male prosoma (Fig. 10A).

Supplement: Supplementary file 5 — Additional file 5. Interactive 3D images of Figs. 10A-K. [file 12983_2021_435_MOESM5_ESM.zip › 12983_2021_435_MOESM4_ESM/Additional file 17.pdf]

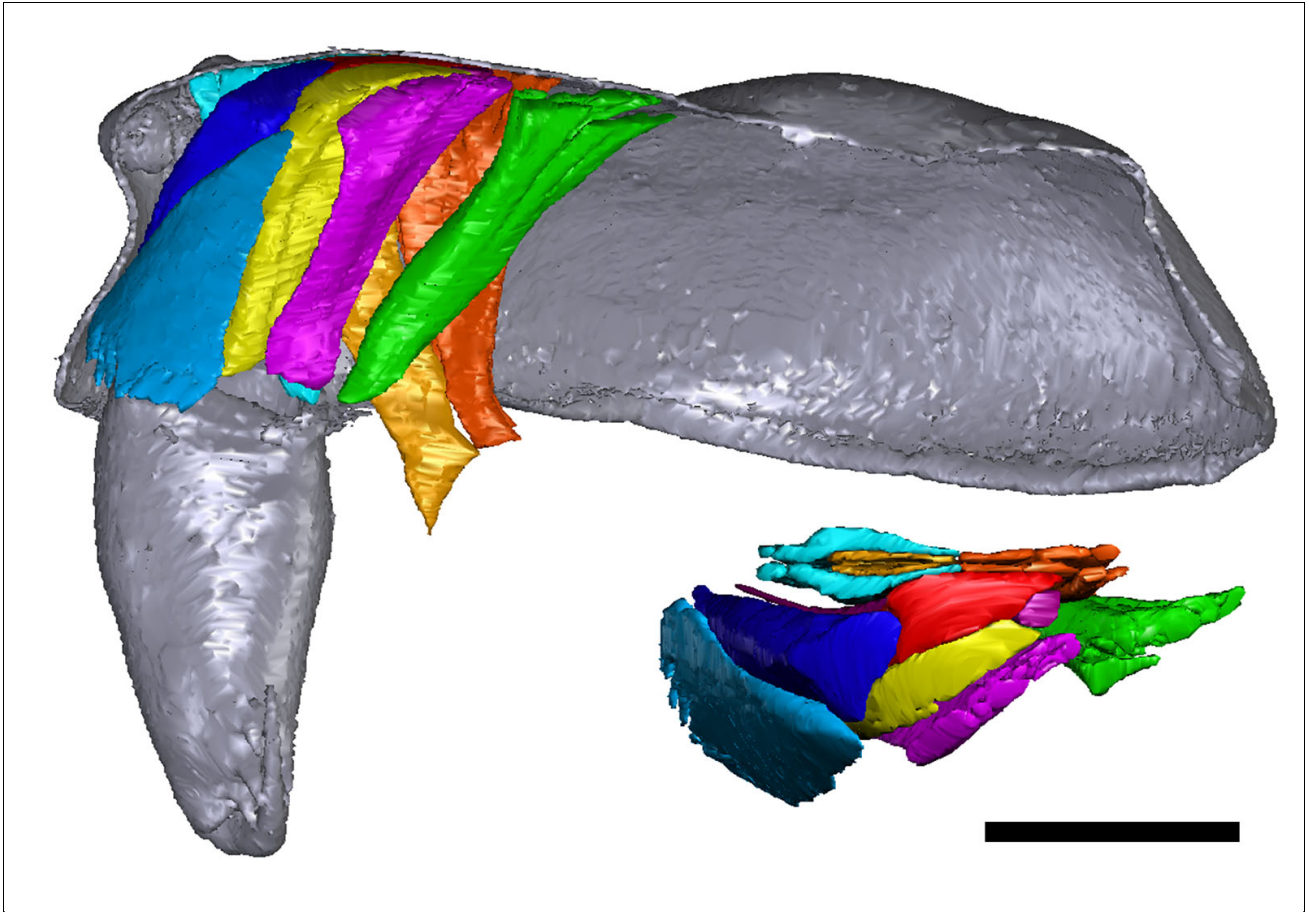

Additional file 18. Interactive 3D image of *Stemonyphantes lineatus* male prosoma (Fig. 10B).

Supplement: Supplementary file 5 — Additional file 5. Interactive 3D images of Figs. 10A-K. [file 12983_2021_435_MOESM5_ESM.zip › 12983_2021_435_MOESM4_ESM/Additional file 18.pdf]

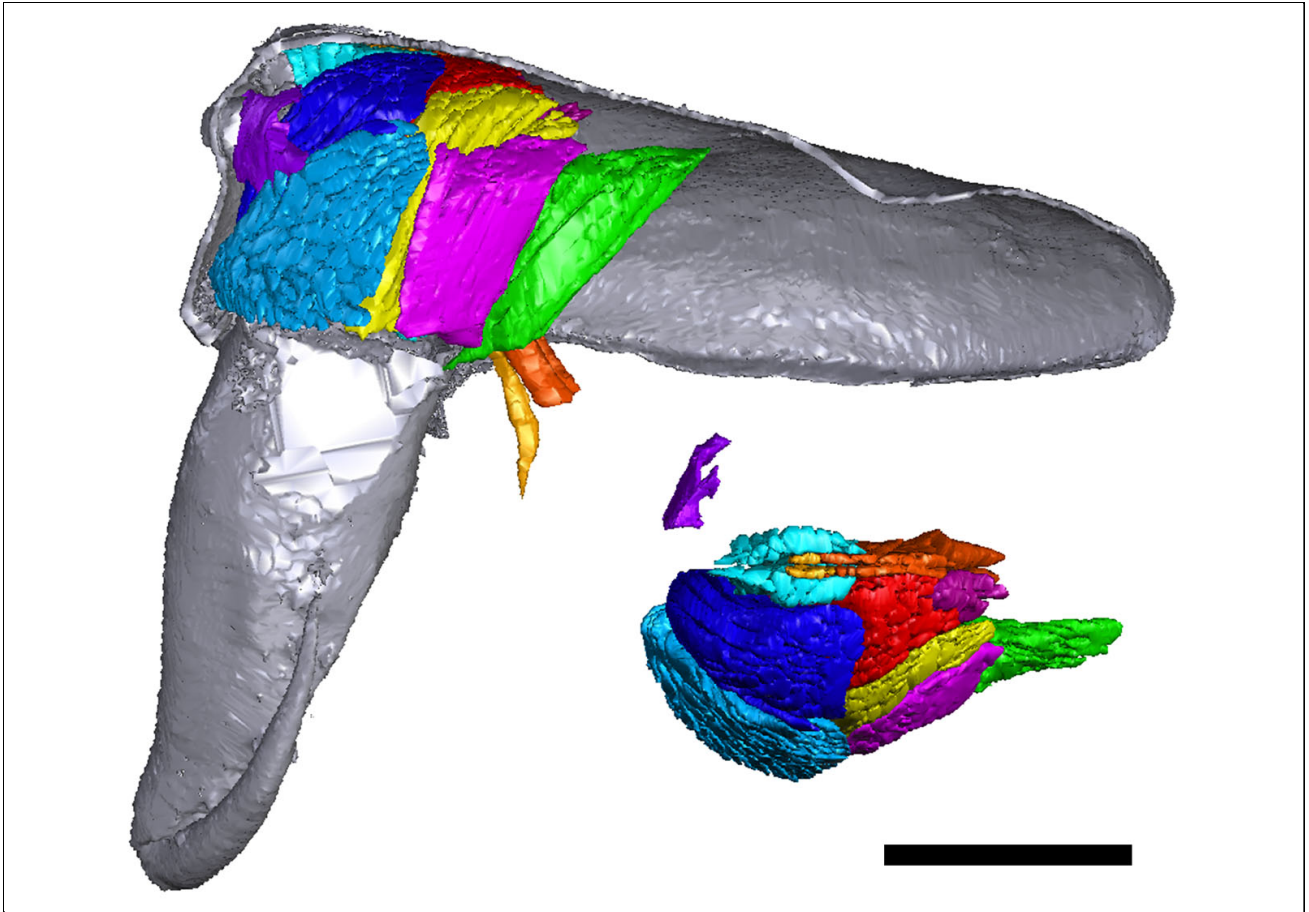

Additional file 19. Interactive 3D image of *Linyphia triangularis* male prosoma (Fig. 10C).

Supplement: Supplementary file 5 — Additional file 5. Interactive 3D images of Figs. 10A-K. [file 12983_2021_435_MOESM5_ESM.zip › 12983_2021_435_MOESM4_ESM/Additional file 19.pdf]

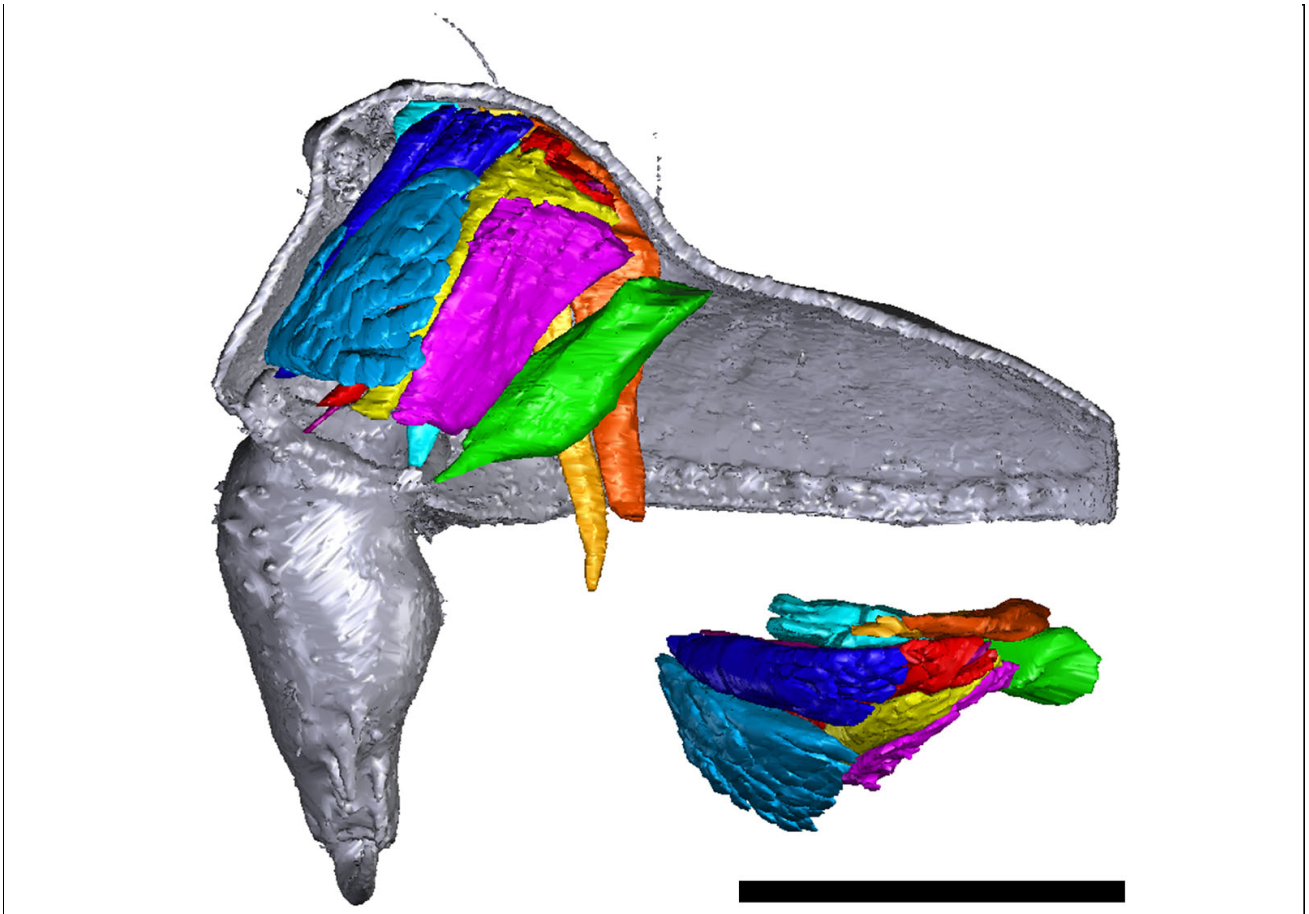

Additional file 20. Interactive 3D image of *Erigone atra* male prosoma (Fig. 10D).

Supplement: Supplementary file 5 — Additional file 5. Interactive 3D images of Figs. 10A-K. [file 12983_2021_435_MOESM5_ESM.zip › 12983_2021_435_MOESM4_ESM/Additional file 20.pdf]

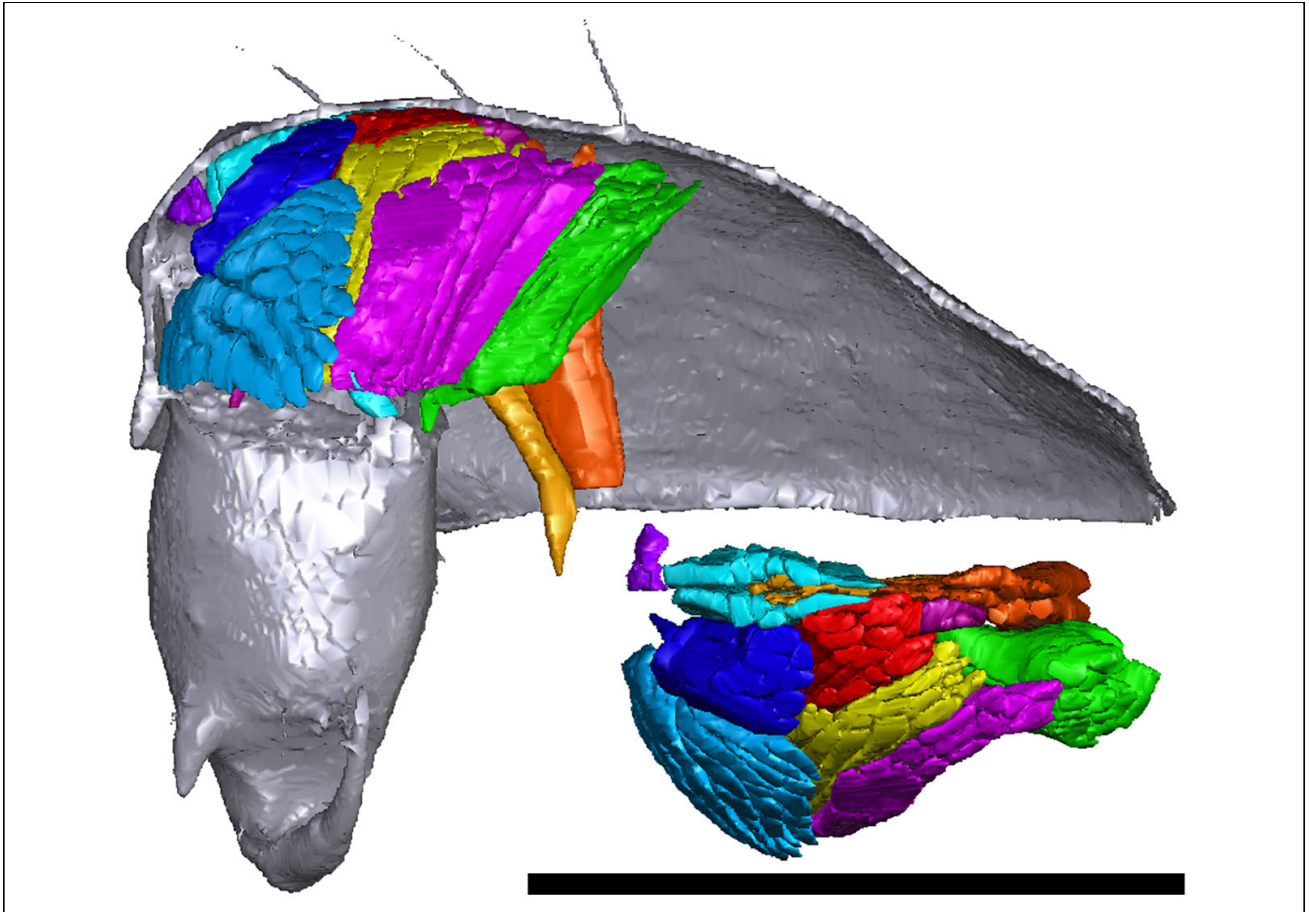

Additional file 21. Interactive 3D image of *Gongylidiellum vivum* male prosoma (Fig. 10E).

Supplement: Supplementary file 5 — Additional file 5. Interactive 3D images of Figs. 10A-K. [file 12983_2021_435_MOESM5_ESM.zip › 12983_2021_435_MOESM4_ESM/Additional file 21.pdf]

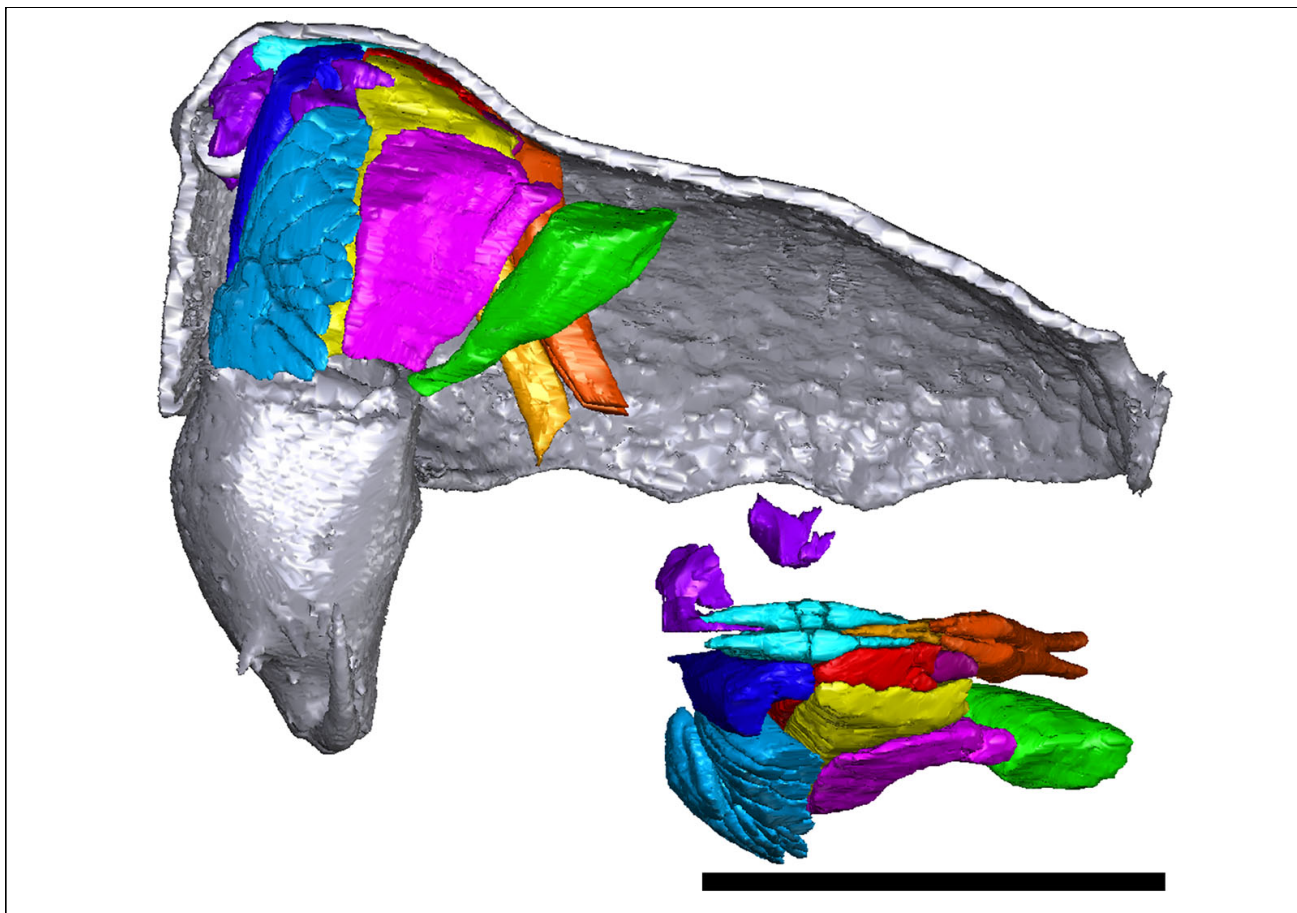

Additional file 22. Interactive 3D image of *Lophomma punctatum* male prosoma (Fig. 10F).

Supplement: Supplementary file 5 — Additional file 5. Interactive 3D images of Figs. 10A-K. [file 12983_2021_435_MOESM5_ESM.zip › 12983_2021_435_MOESM4_ESM/Additional file 22.pdf]

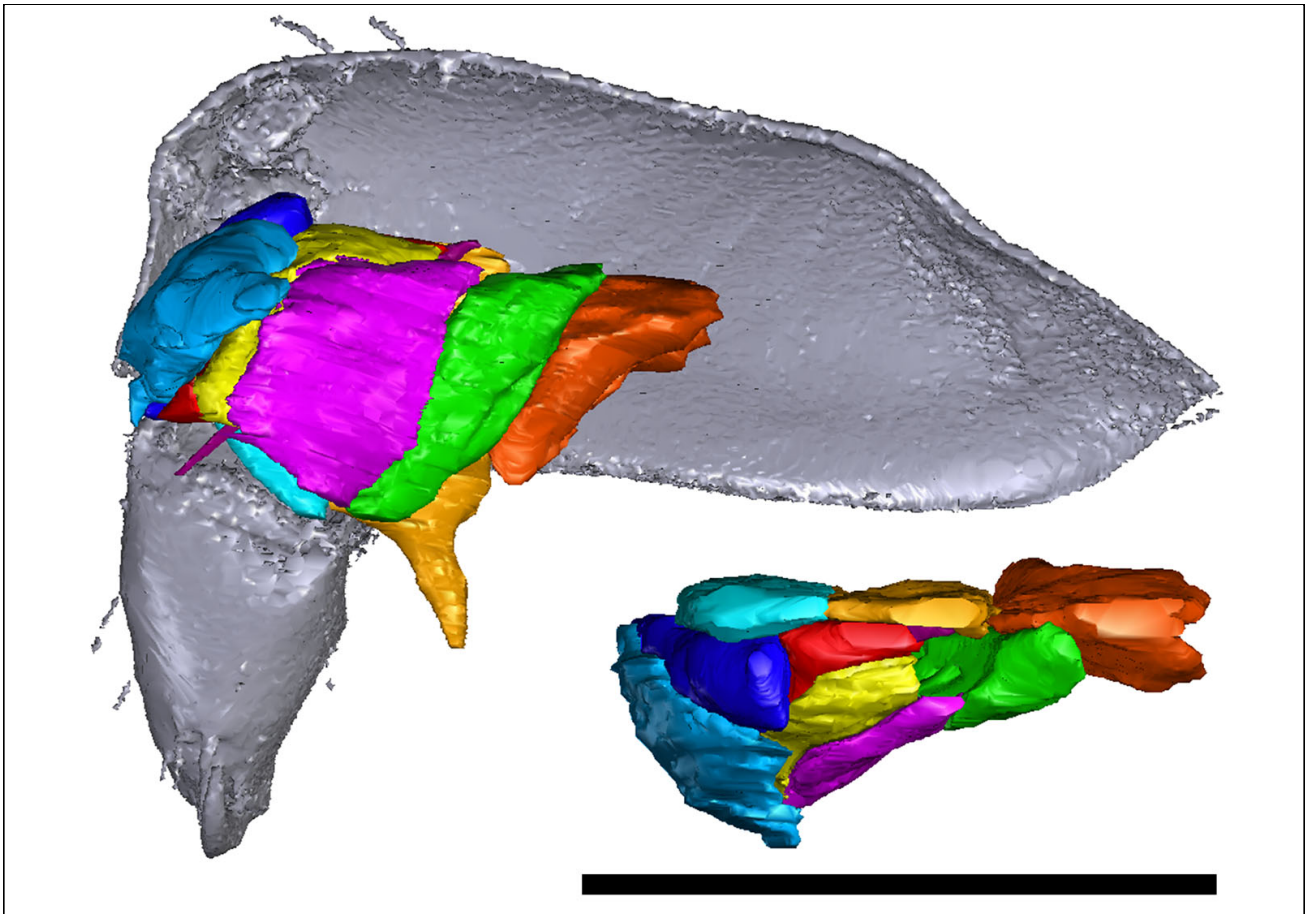

Additional file 23. Interactive 3D image of *Diplocentria bidentata* male prosoma (Fig. 10G).

Supplement: Supplementary file 5 — Additional file 5. Interactive 3D images of Figs. 10A-K. [file 12983_2021_435_MOESM5_ESM.zip › 12983_2021_435_MOESM4_ESM/Additional file 23.pdf]

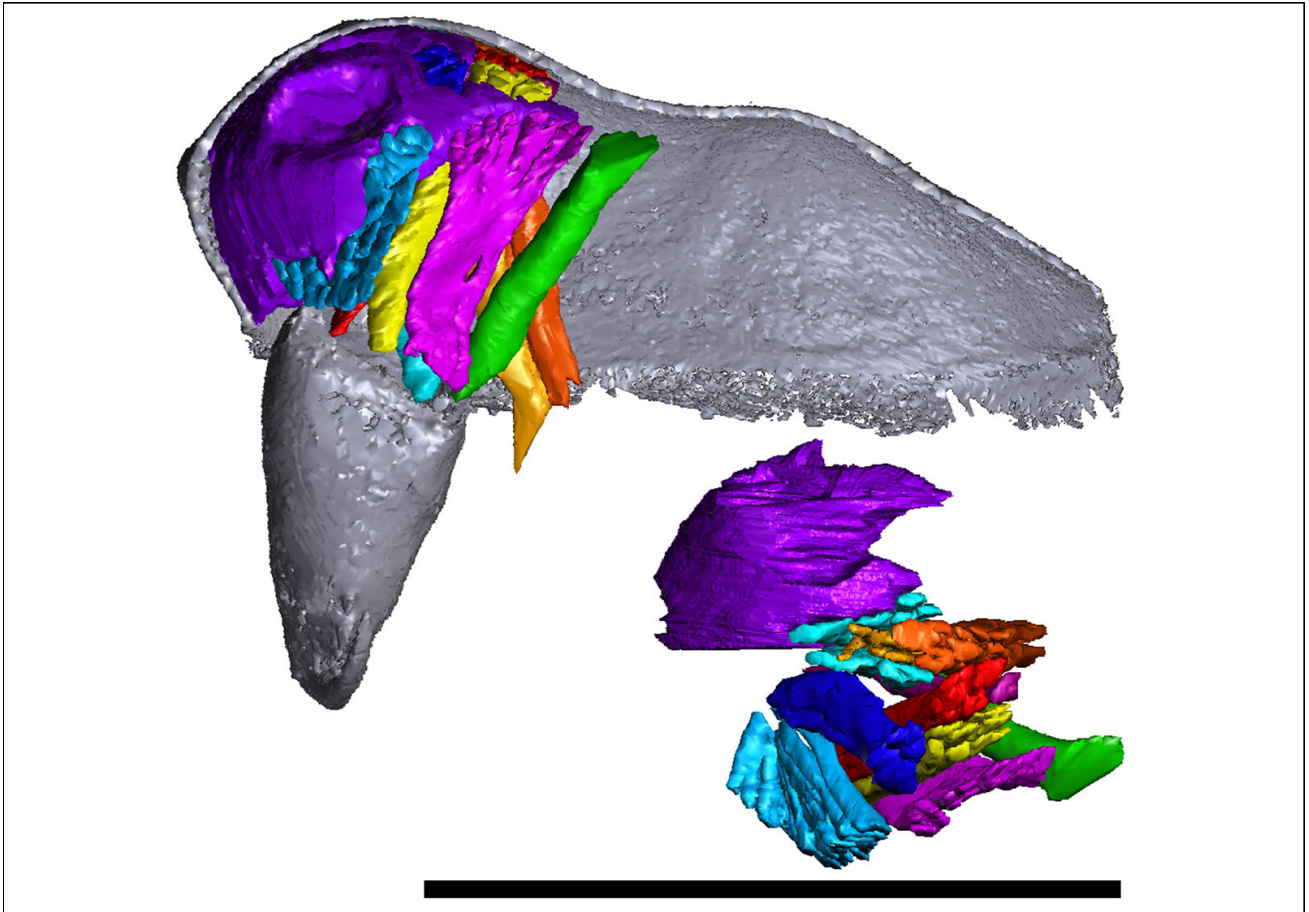

Additional file 24. Interactive 3D image of *Araeoncus humilis* male prosoma (Fig. 10H).

Supplement: Supplementary file 5 — Additional file 5. Interactive 3D images of Figs. 10A-K. [file 12983_2021_435_MOESM5_ESM.zip › 12983_2021_435_MOESM4_ESM/Additional file 24.pdf]

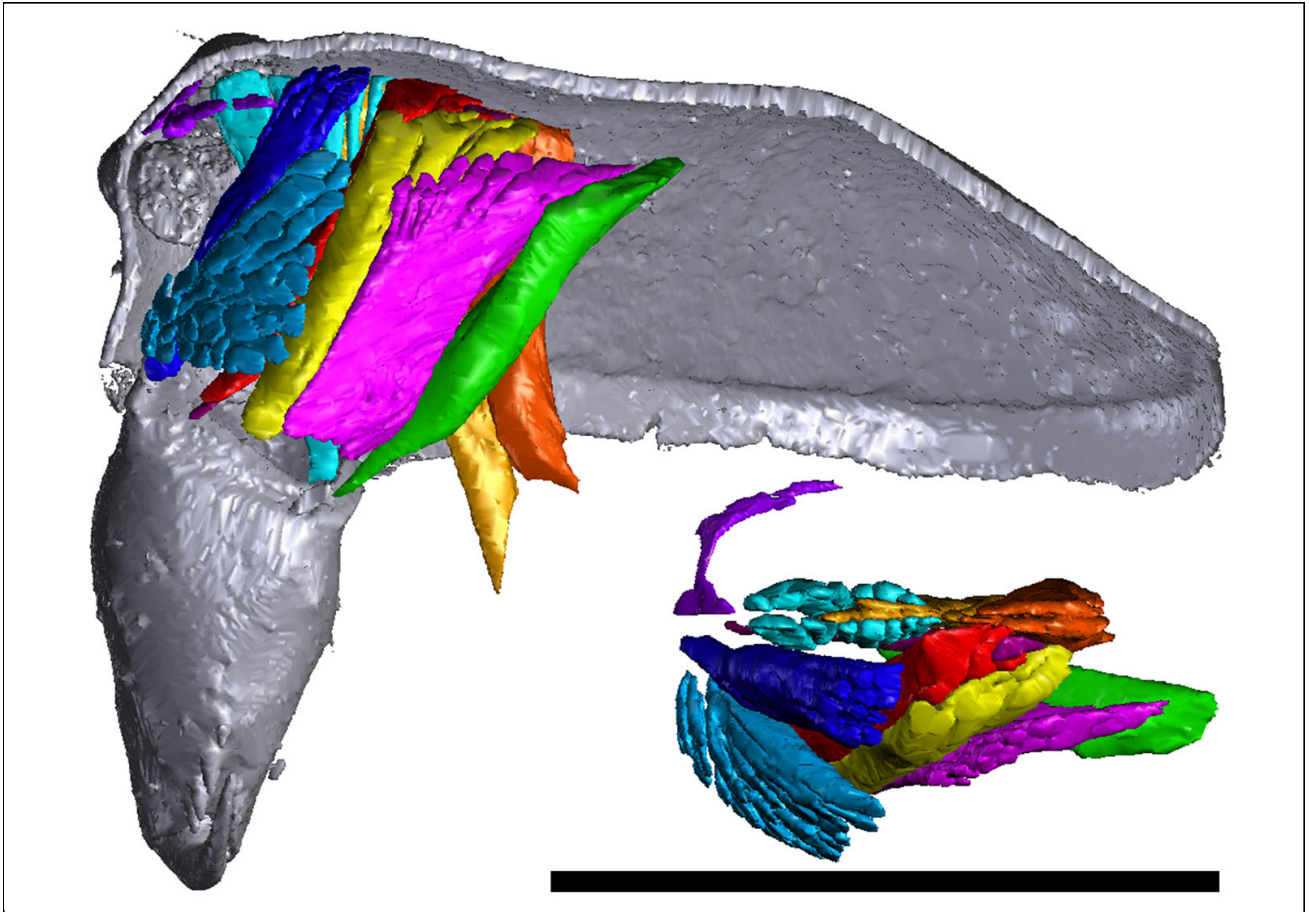

Additional file 25. Interactive 3D image of *Jilinus hulongensis* male prosoma (Fig. 10I).

Supplement: Supplementary file 5 — Additional file 5. Interactive 3D images of Figs. 10A-K. [file 12983_2021_435_MOESM5_ESM.zip › 12983_2021_435_MOESM4_ESM/Additional file 25.pdf]

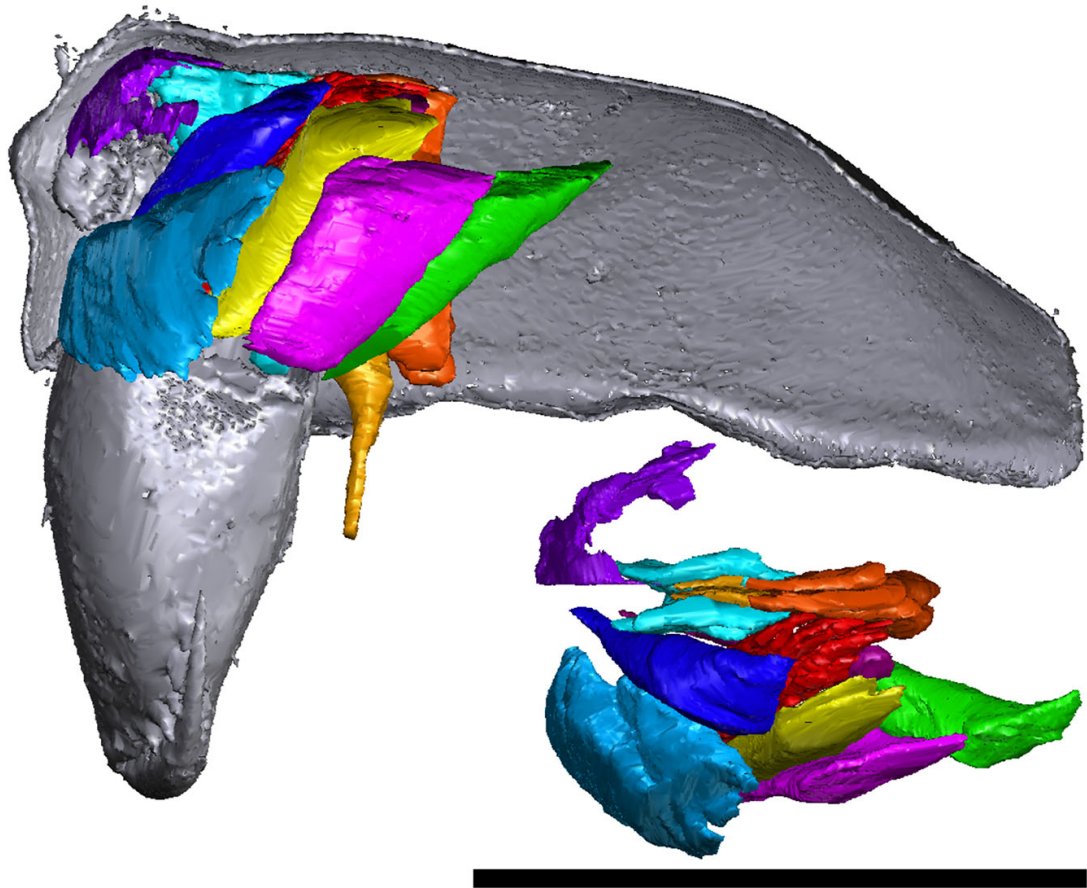

Additional file 26. Interactive 3D image of *Cornitibia simplicithorax* male prosoma (Fig. 10J).

Supplement: Supplementary file 5 — Additional file 5. Interactive 3D images of Figs. 10A-K. [file 12983_2021_435_MOESM5_ESM.zip › 12983_2021_435_MOESM4_ESM/Additional file 26.pdf]

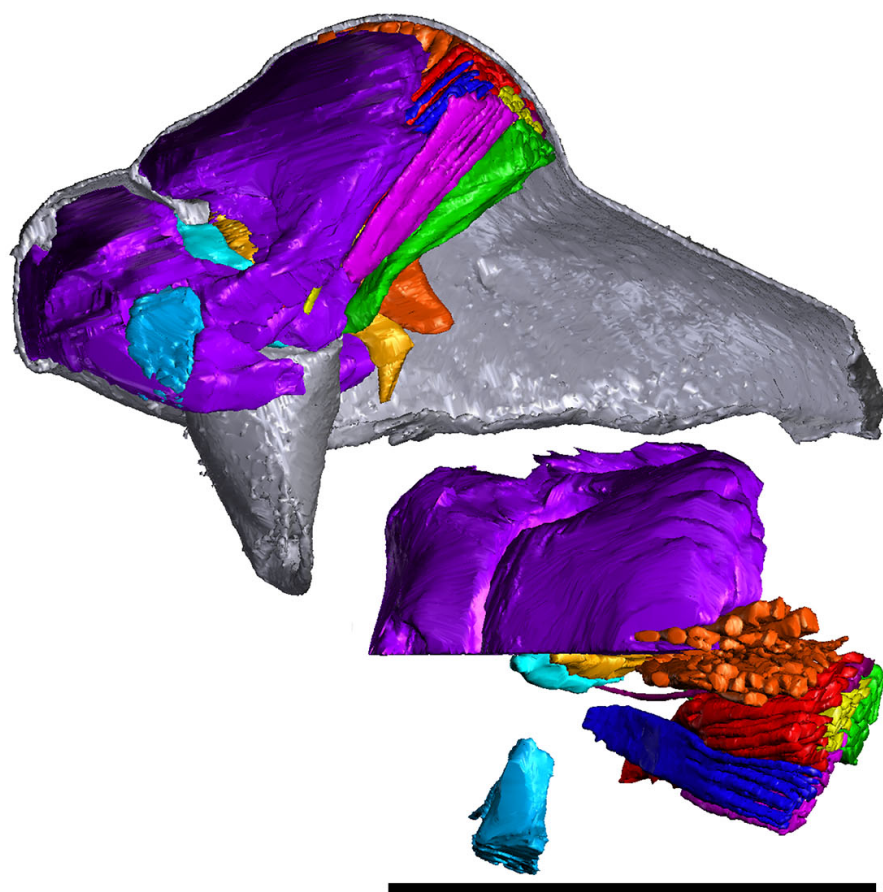

Additional file 27. Interactive 3D image of *Emertongone montifera* male prosoma (Fig. 10K).

Supplement: Supplementary file 5 — Additional file 5. Interactive 3D images of Figs. 10A-K. [file 12983_2021_435_MOESM5_ESM.zip › 12983_2021_435_MOESM4_ESM/Additional file 27.pdf]

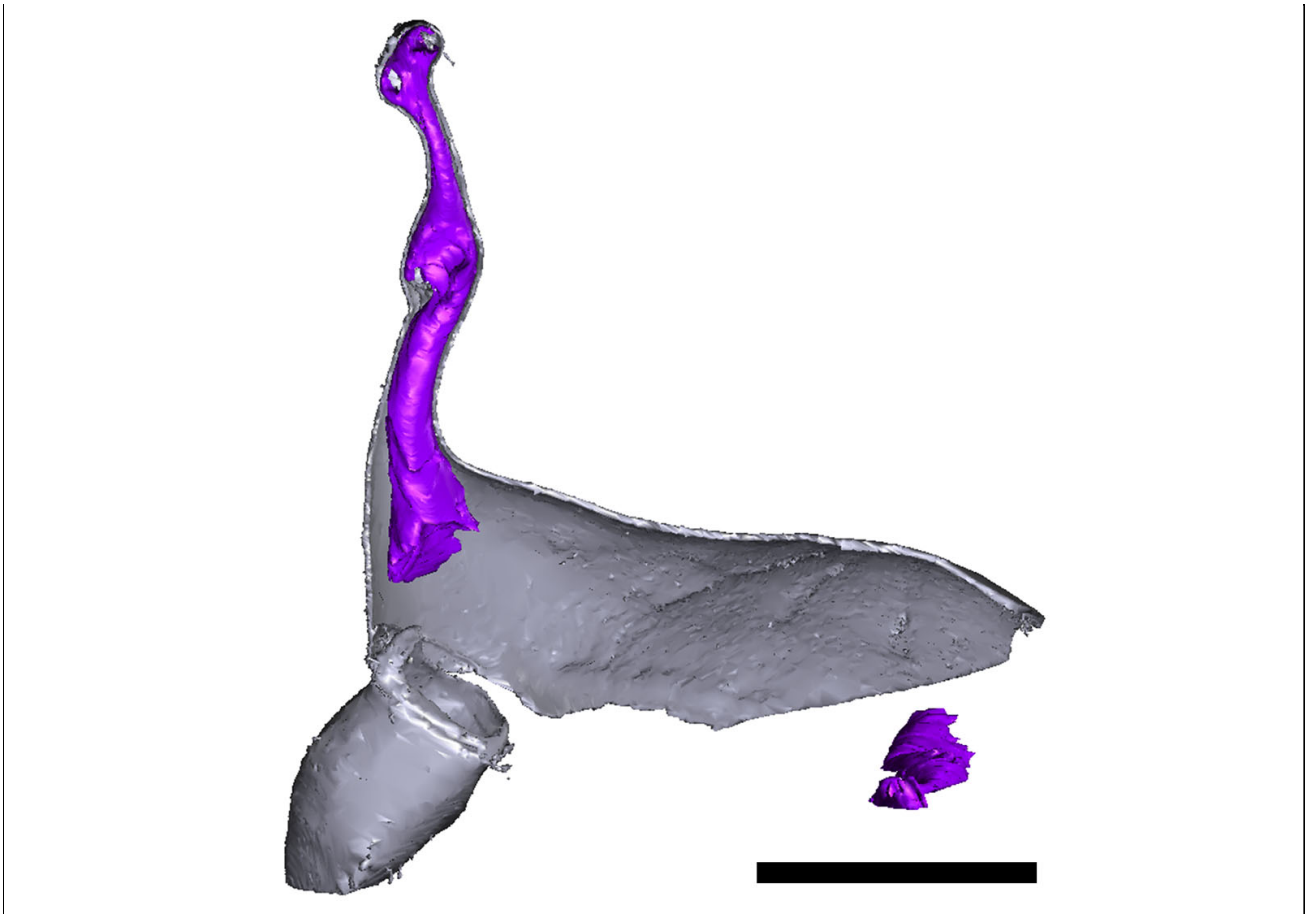

Additional file 28. Interactive 3D image of *Walckenaeria acuminata* male prosoma (Fig. 11A).

Supplement: Supplementary file 6 — Additional file 6. Interactive 3D images of Figs. 11A-L. [file 12983_2021_435_MOESM6_ESM.zip › 12983_2021_435_MOESM5_ESM/Additional file 28.pdf]

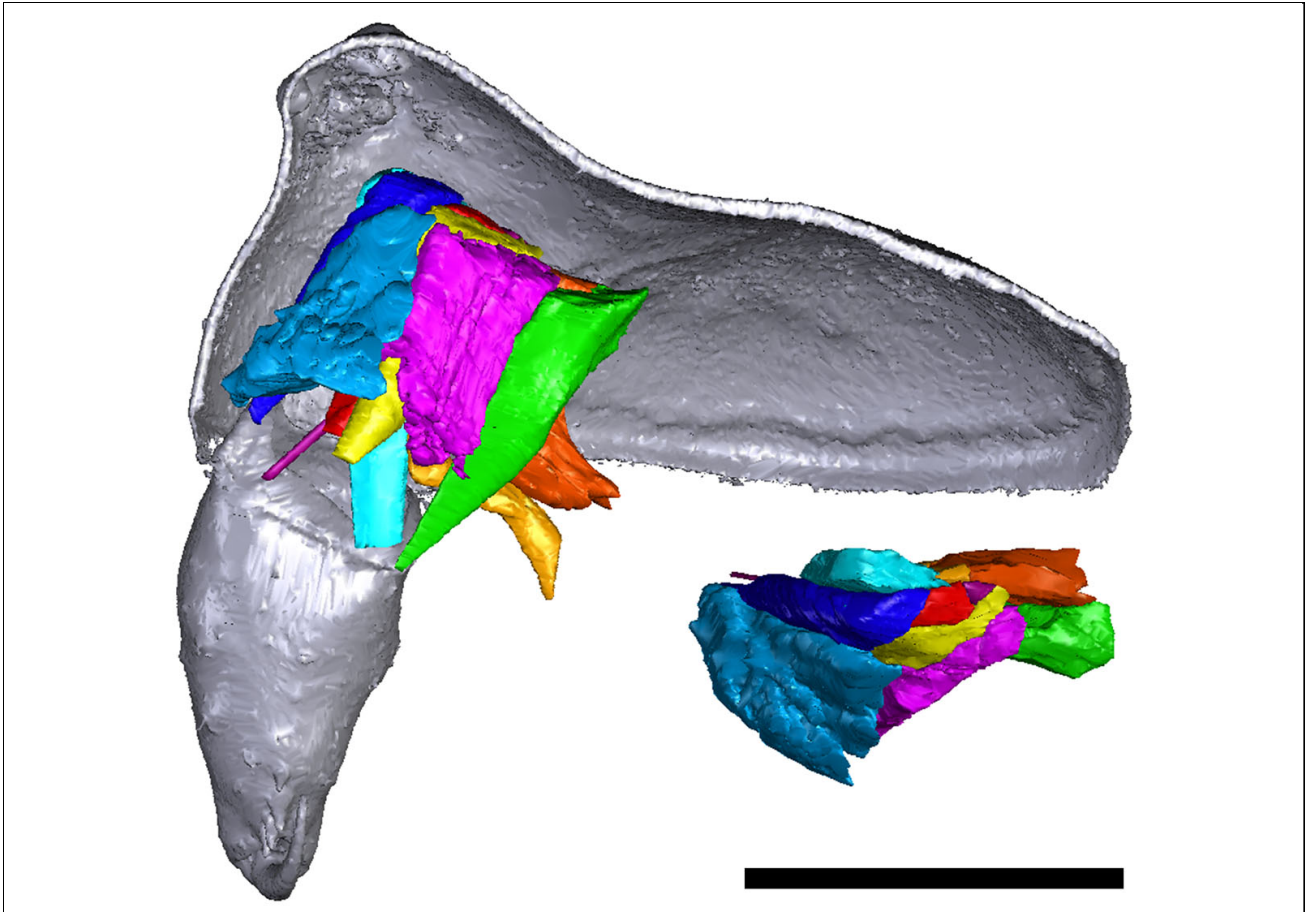

Additional file 29. Interactive 3D image of *Gonatium rubellum* male prosoma (Fig. 11B).

Supplement: Supplementary file 6 — Additional file 6. Interactive 3D images of Figs. 11A-L. [file 12983_2021_435_MOESM6_ESM.zip › 12983_2021_435_MOESM5_ESM/Additional file 29.pdf]

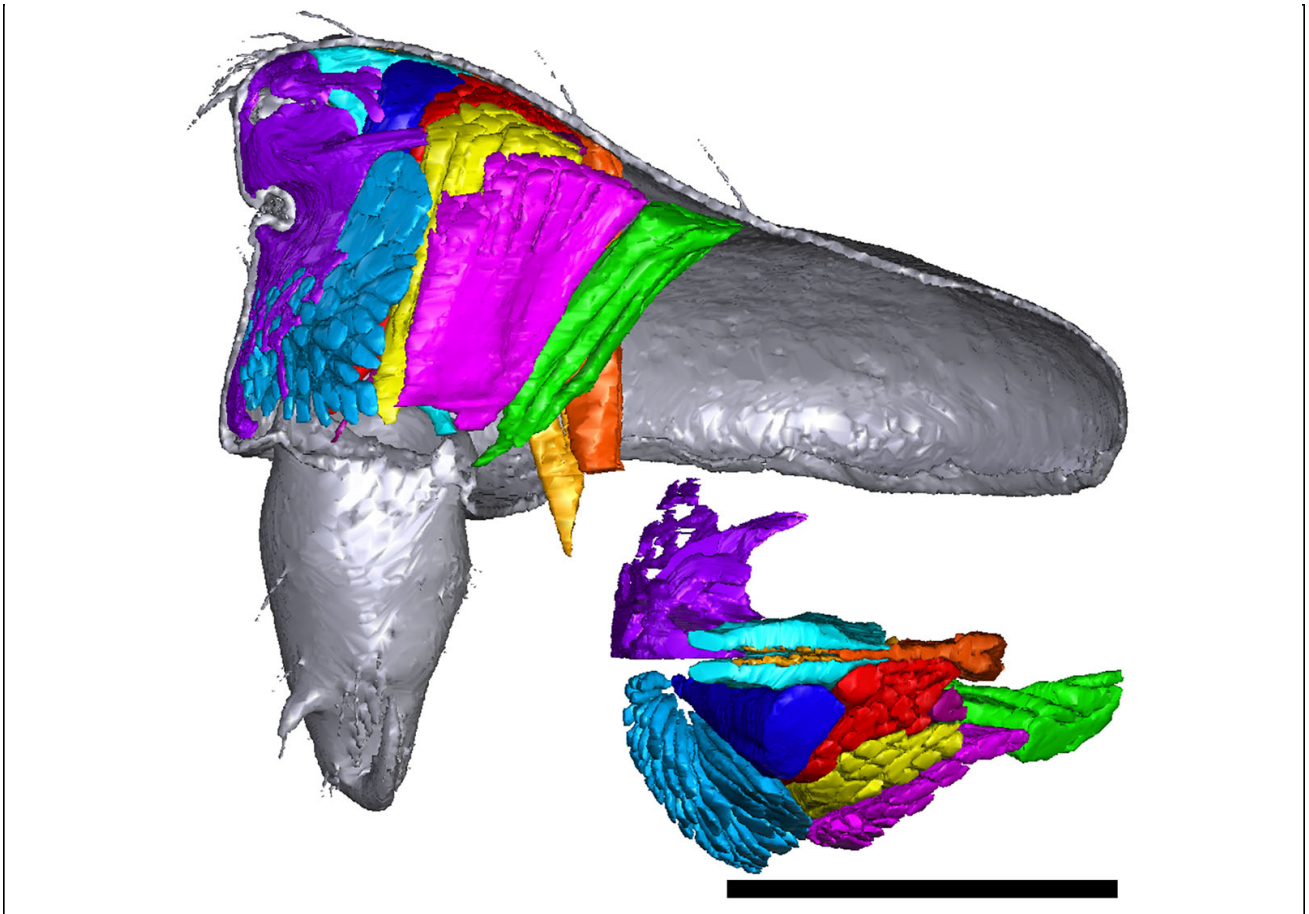

Additional file 30. Interactive 3D image of *Shaanxinus mingchihensis* male prosoma (Fig. 11C).

Supplement: Supplementary file 6 — Additional file 6. Interactive 3D images of Figs. 11A-L. [file 12983_2021_435_MOESM6_ESM.zip › 12983_2021_435_MOESM5_ESM/Additional file 30.pdf]

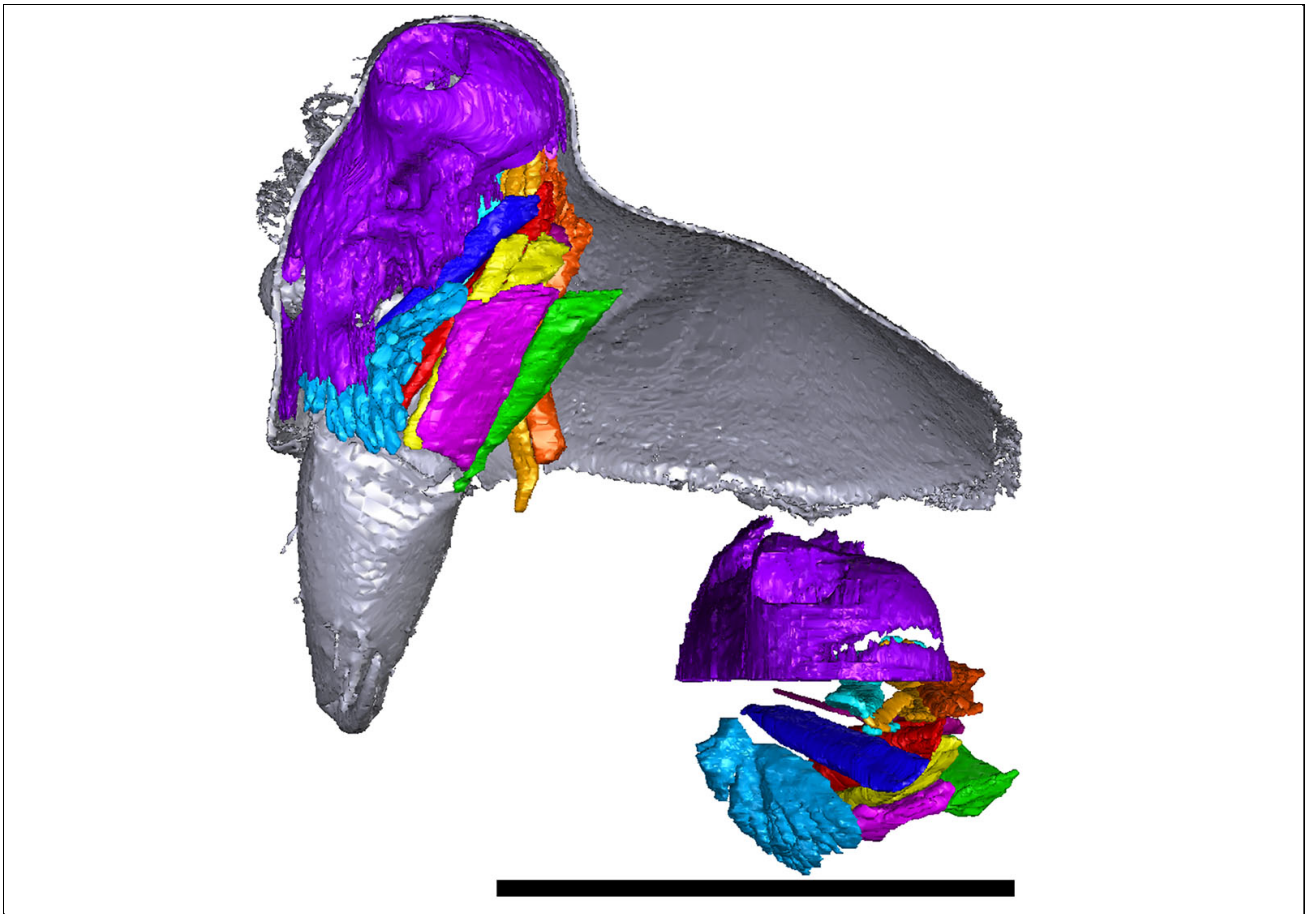

Additional file 34. Interactive 3D image of *Atypena cirrifrons* male prosoma (Fig. 11G).

Supplement: Supplementary file 6 — Additional file 6. Interactive 3D images of Figs. 11A-L. [file 12983_2021_435_MOESM6_ESM.zip › 12983_2021_435_MOESM5_ESM/Additional file 34.pdf]

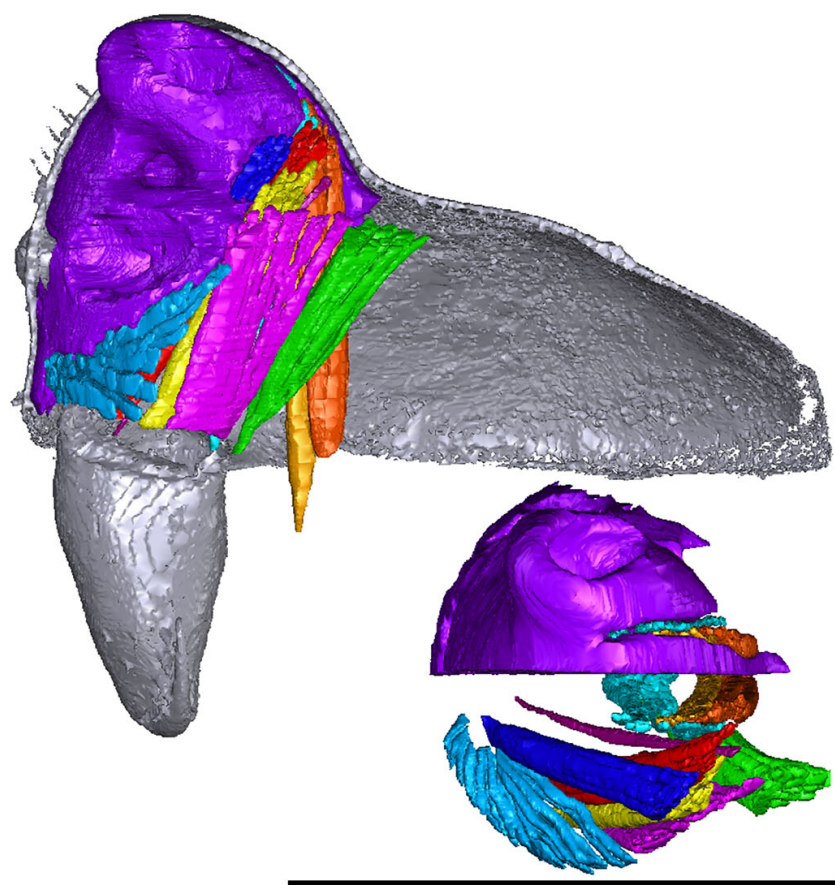

Additional file 35. Interactive 3D image of *Atypena formosana* male prosoma (Fig. 11H).

Supplement: Supplementary file 6 — Additional file 6. Interactive 3D images of Figs. 11A-L. [file 12983_2021_435_MOESM6_ESM.zip › 12983_2021_435_MOESM5_ESM/Additional file 35.pdf]

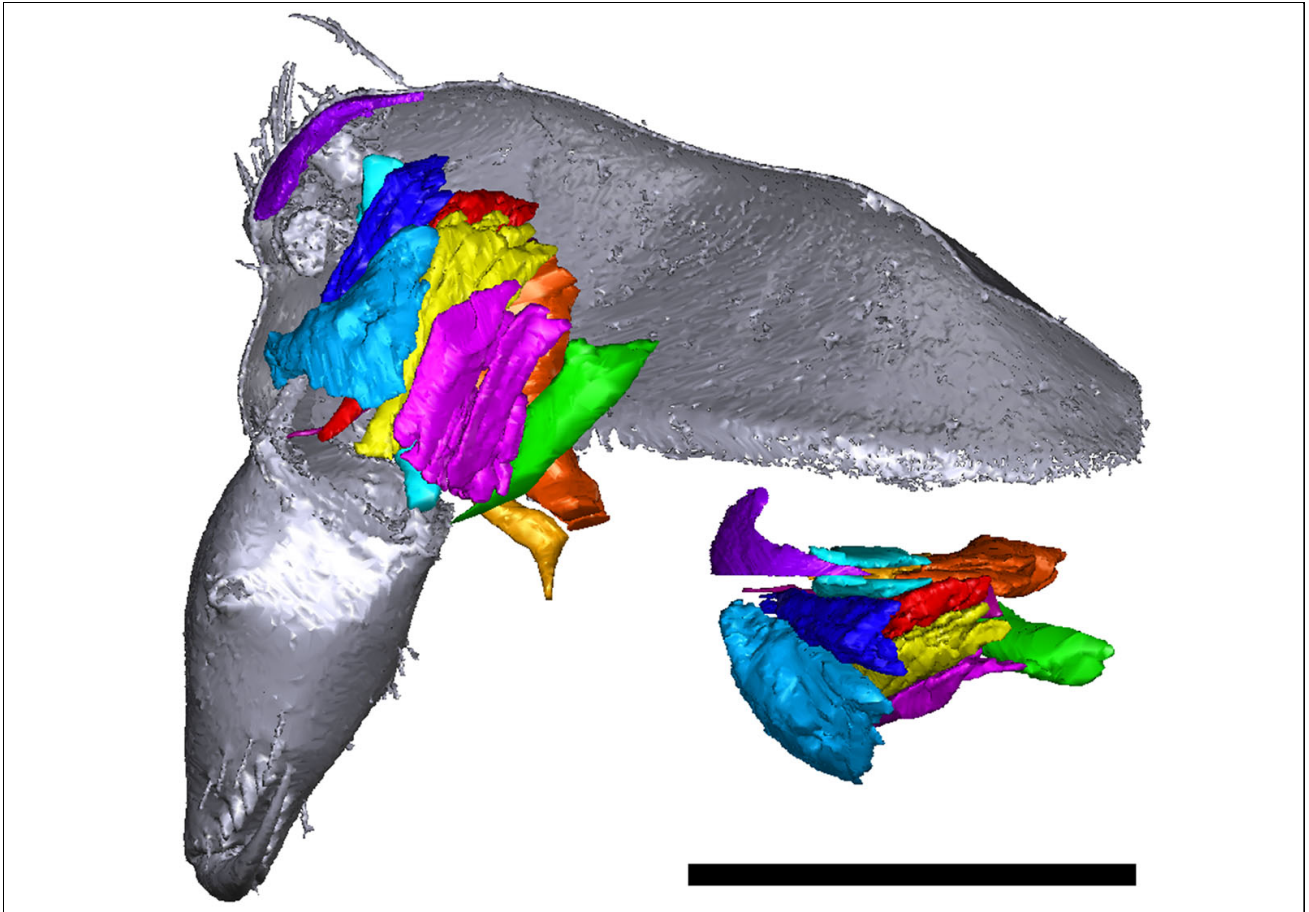

Additional file 36. Interactive 3D image of *Oedothorax uncus incertae sedis* male prosoma (Fig. 11I).

Supplement: Supplementary file 6 — Additional file 6. Interactive 3D images of Figs. 11A-L. [file 12983_2021_435_MOESM6_ESM.zip › 12983_2021_435_MOESM5_ESM/Additional file 36.pdf]

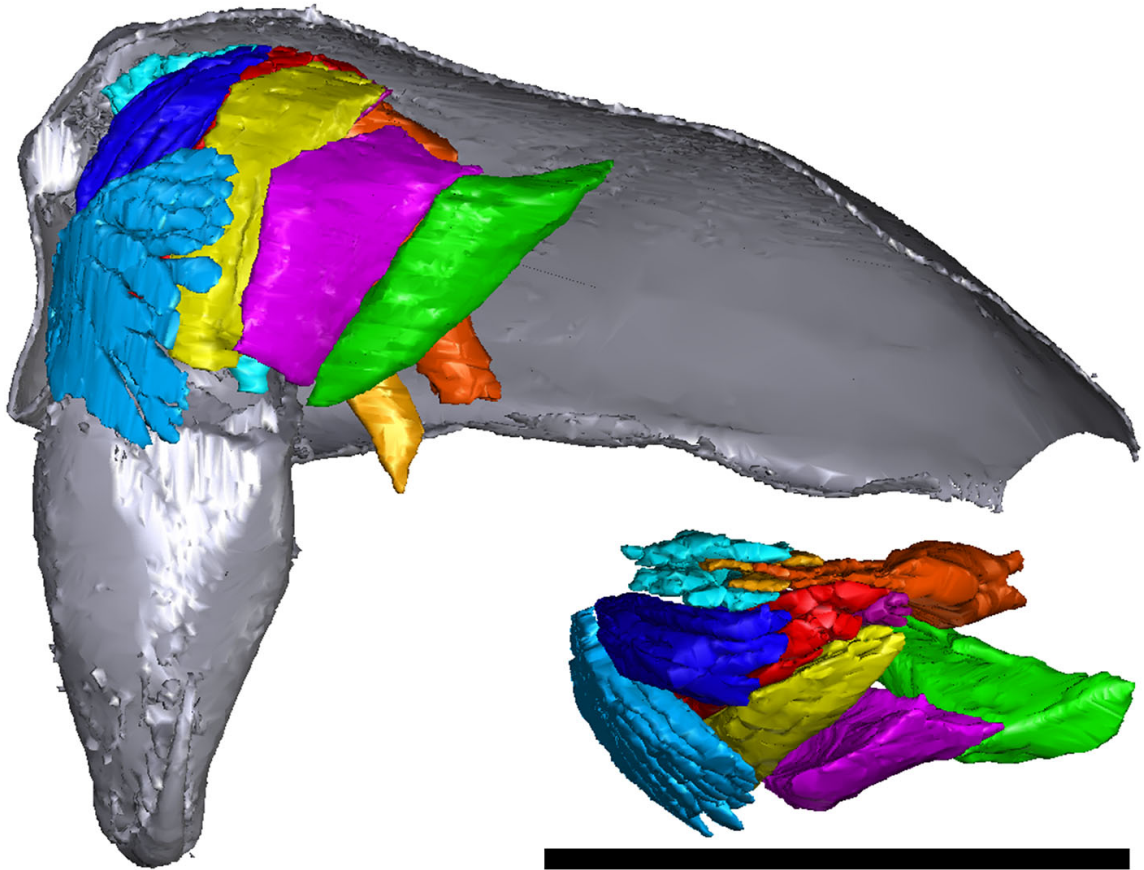

Additional file 37. Interactive 3D image of *Oedothorax cunur incertae sedis* male prosoma (Fig. 11J).

Supplement: Supplementary file 6 — Additional file 6. Interactive 3D images of Figs. 11A-L. [file 12983_2021_435_MOESM6_ESM.zip › 12983_2021_435_MOESM5_ESM/Additional file 37.pdf]

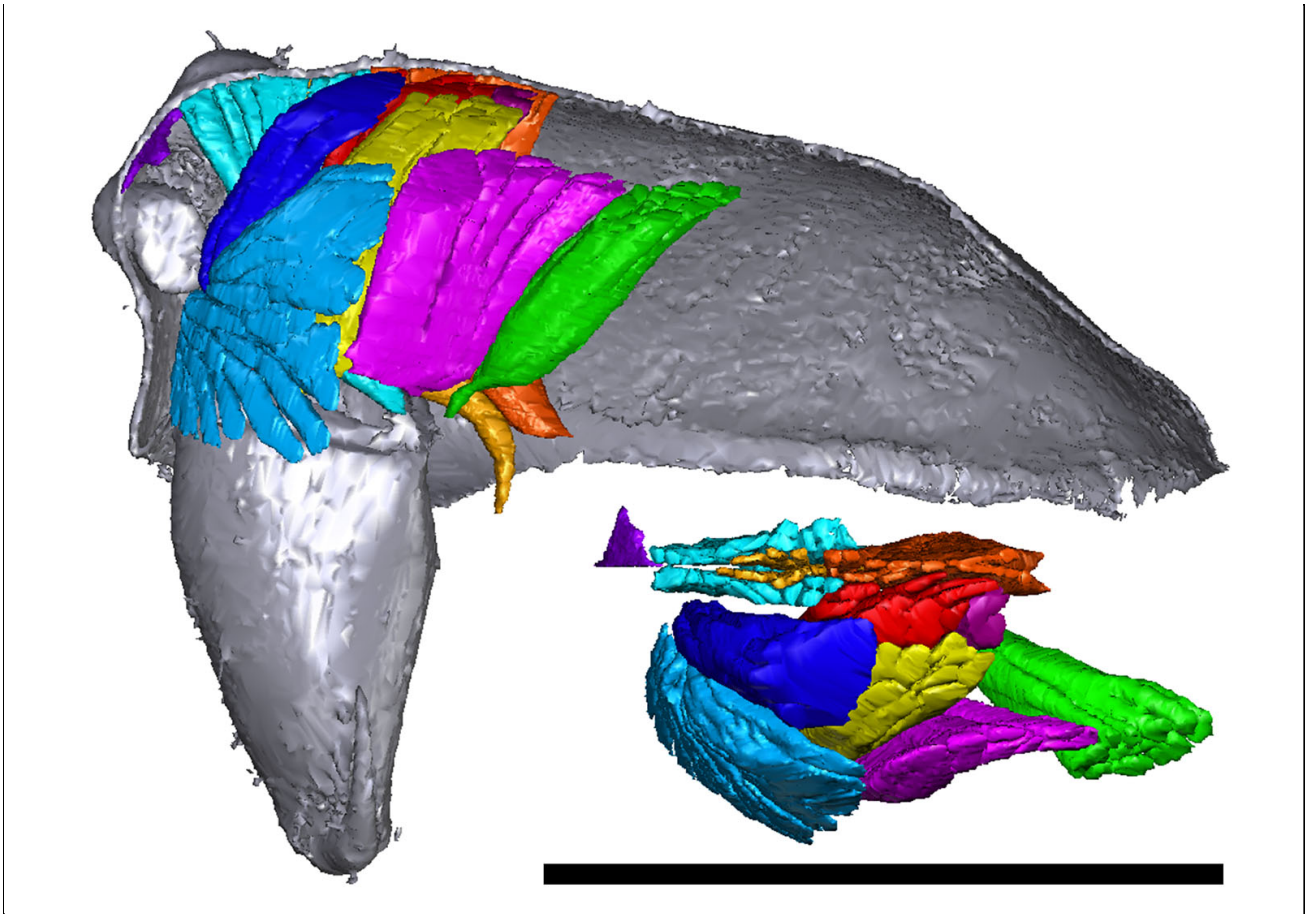

Additional file 38. Interactive 3D image of *Oedothorax stylus incertae sedis* male prosoma (Fig. 11K).

Supplement: Supplementary file 6 — Additional file 6. Interactive 3D images of Figs. 11A-L. [file 12983_2021_435_MOESM6_ESM.zip › 12983_2021_435_MOESM5_ESM/Additional file 38.pdf]

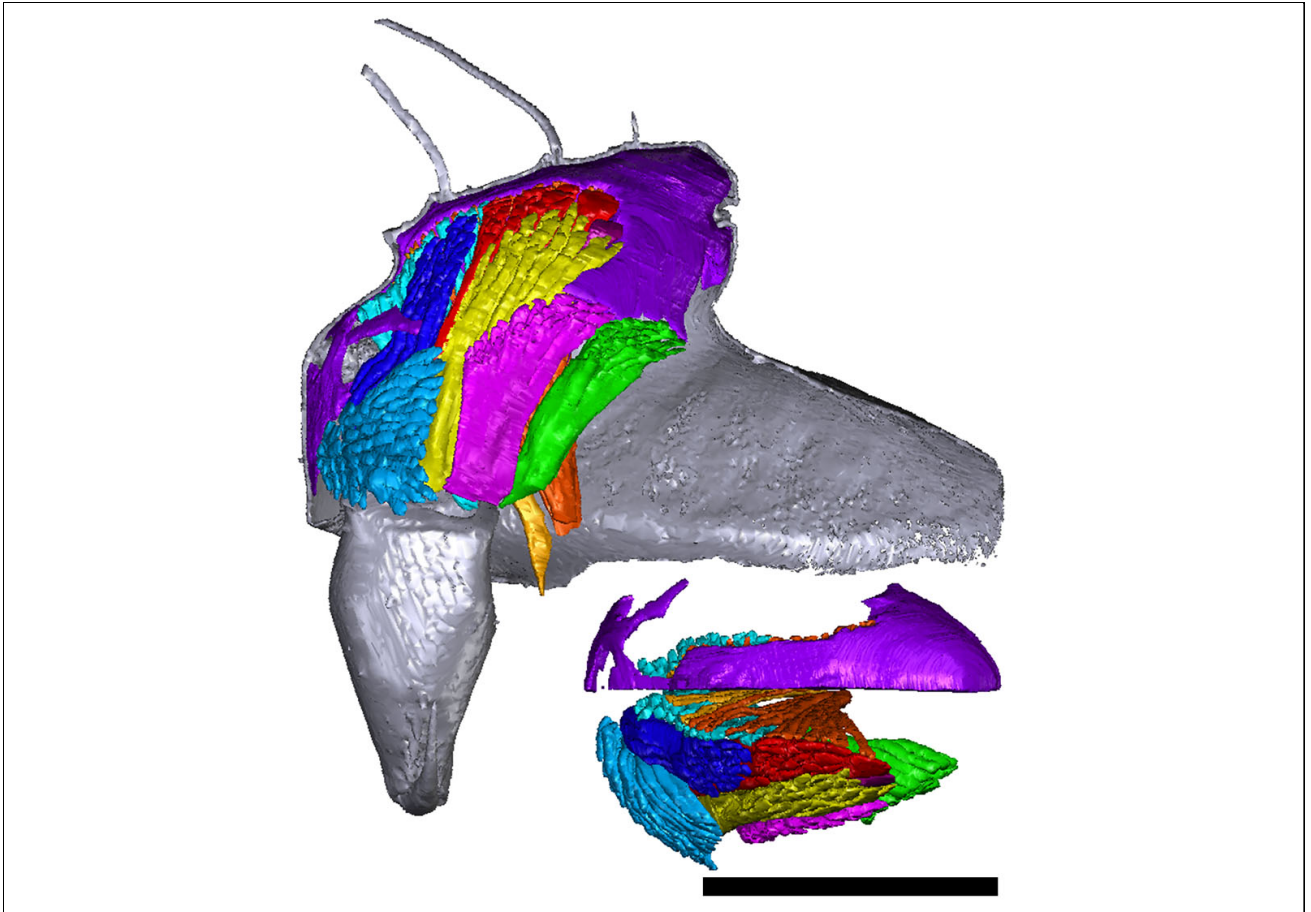

Additional file 39. Interactive 3D image of *Nasoona setifera* male prosoma (Fig. 11L).

Supplement: Supplementary file 6 — Additional file 6. Interactive 3D images of Figs. 11A-L. [file 12983_2021_435_MOESM6_ESM.zip › 12983_2021_435_MOESM5_ESM/Additional file 39.pdf]

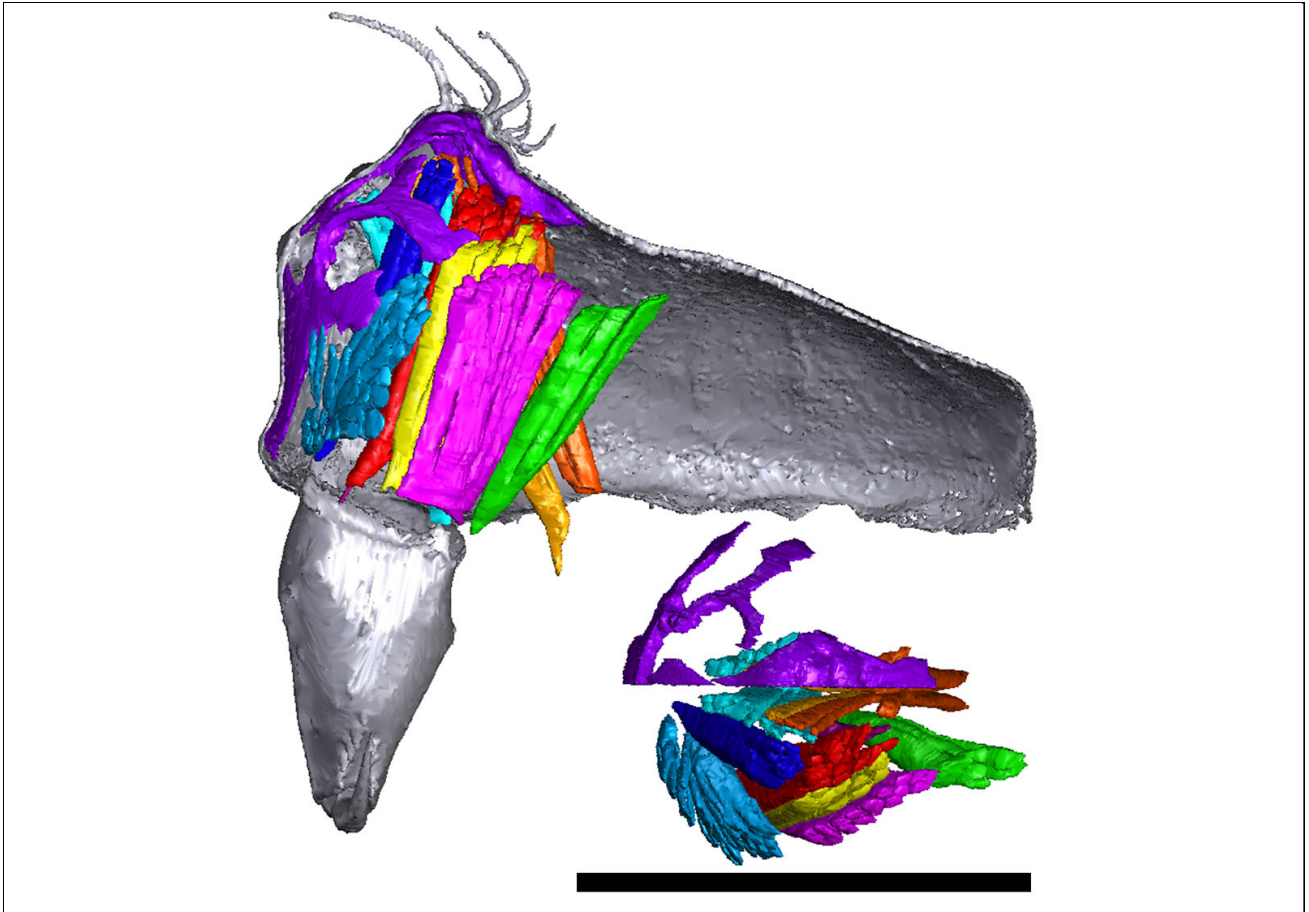

Additional file 40. Interactive 3D image of *Nasoona crucifera* male prosoma (Fig. 12A).

Supplement: Supplementary file 7 — Additional file 7. Interactive 3D images of Figs. 12A-L. [file 12983_2021_435_MOESM7_ESM.zip › 12983_2021_435_MOESM6_ESM/Additional file 40.pdf]

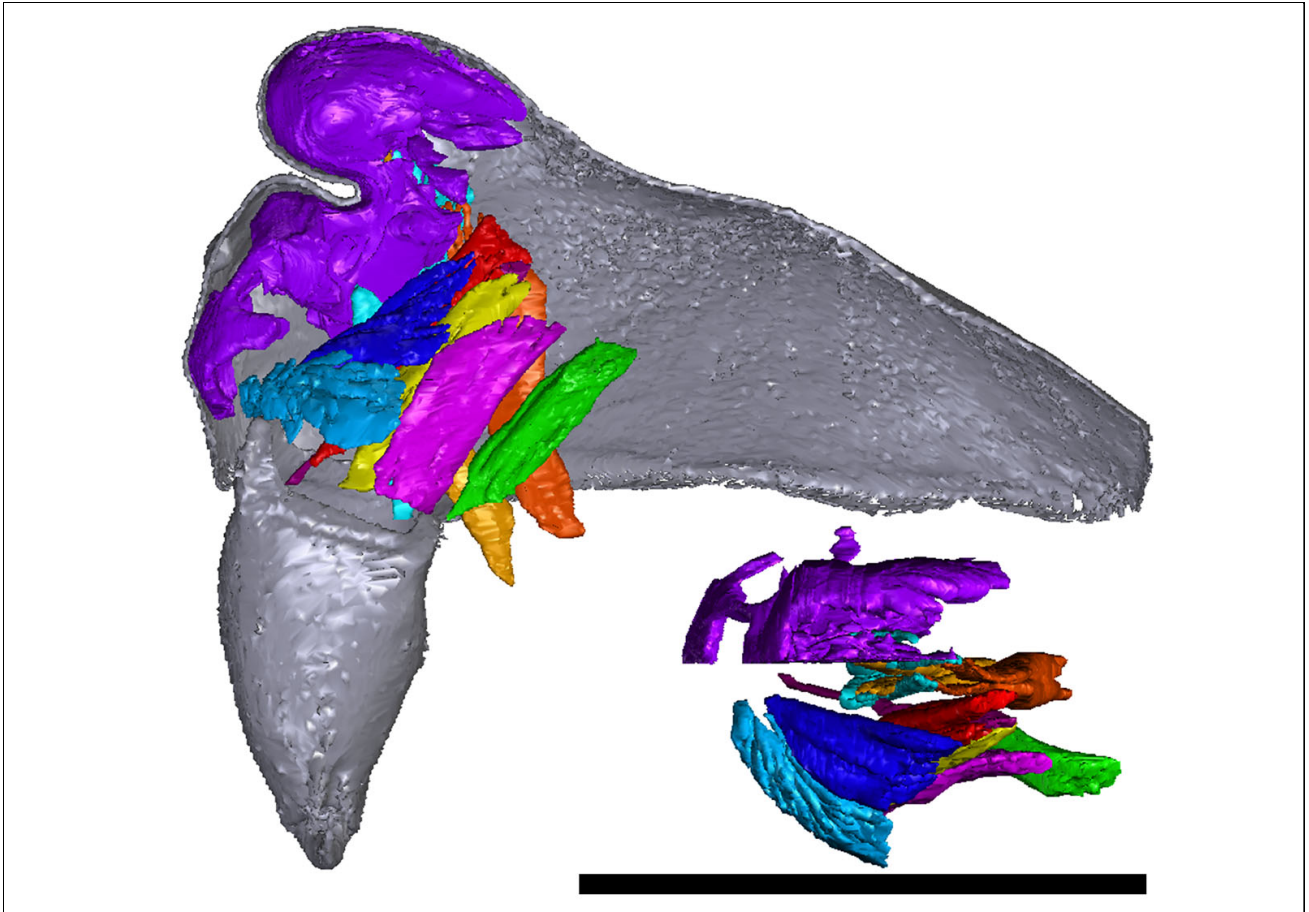

Additional file 41. Interactive 3D image of *Mitragery globiceps* male prosoma (Fig. 12B).

Supplement: Supplementary file 7 — Additional file 7. Interactive 3D images of Figs. 12A-L. [file 12983_2021_435_MOESM7_ESM.zip › 12983_2021_435_MOESM6_ESM/Additional file 41.pdf]

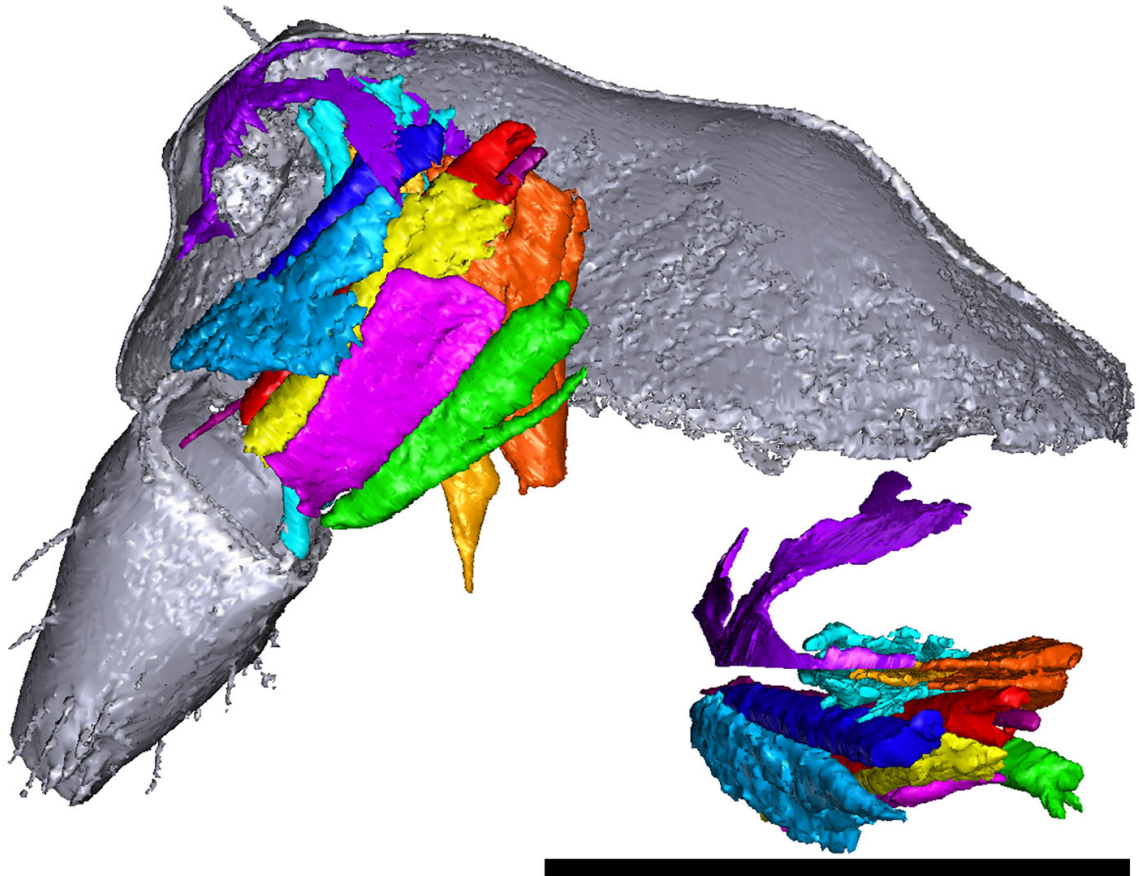

Additional file 42. Interactive 3D image of *Mitrager hirsuta* male prosoma (Fig. 12C).

Supplement: Supplementary file 7 — Additional file 7. Interactive 3D images of Figs. 12A-L. [file 12983_2021_435_MOESM7_ESM.zip › 12983_2021_435_MOESM6_ESM/Additional file 42.pdf]

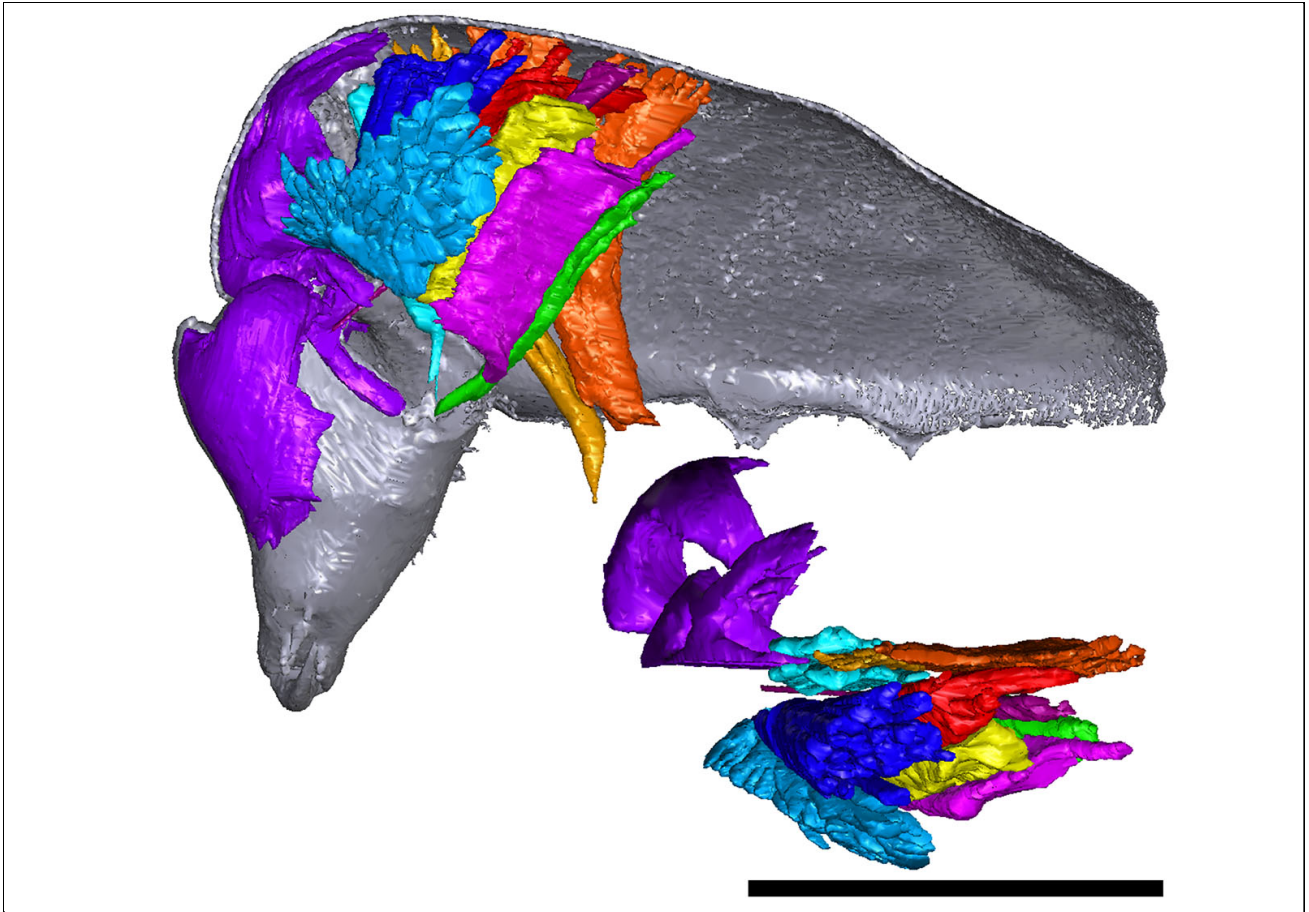

Additional file 43. Interactive 3D image of *Mitrager clypeellum* male prosoma (Fig. 12D).

Supplement: Supplementary file 7 — Additional file 7. Interactive 3D images of Figs. 12A-L. [file 12983_2021_435_MOESM7_ESM.zip › 12983_2021_435_MOESM6_ESM/Additional file 43.pdf]

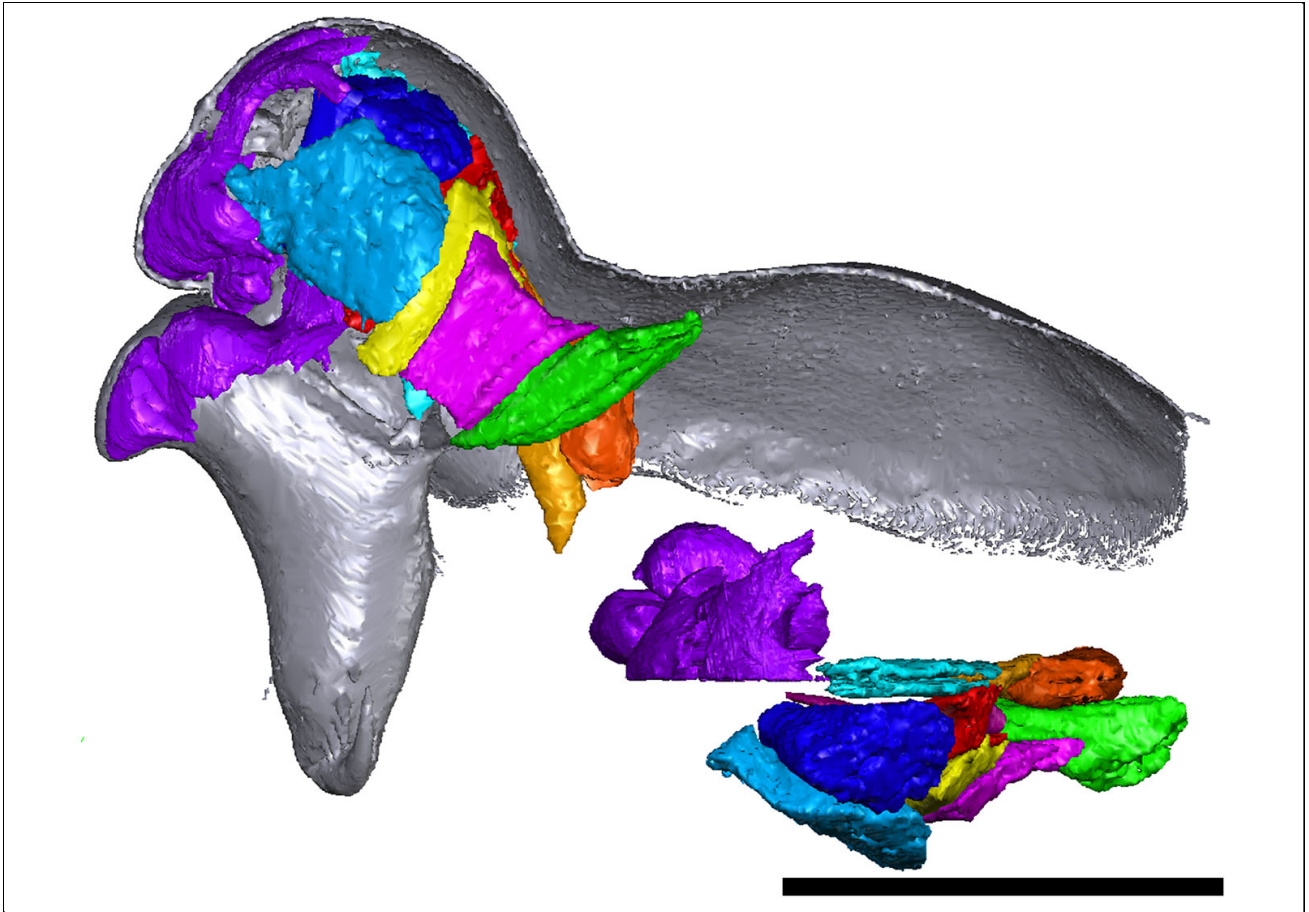

Additional file 44. Interactive 3D image of *Mitrager elongata* male prosoma (Fig. 12E).

Supplement: Supplementary file 7 — Additional file 7. Interactive 3D images of Figs. 12A-L. [file 12983_2021_435_MOESM7_ESM.zip › 12983_2021_435_MOESM6_ESM/Additional file 44.pdf]

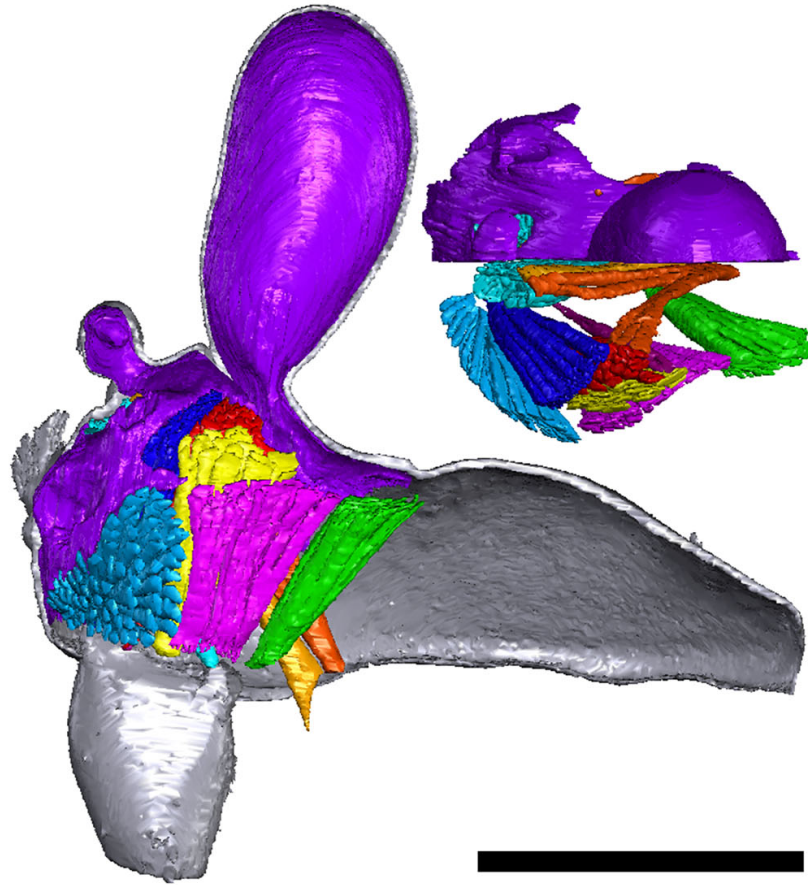

Additional file 45. Interactive 3D image of *Mitrager noordami* male prosoma (Fig. 12F).

Supplement: Supplementary file 7 — Additional file 7. Interactive 3D images of Figs. 12A-L. [file 12983_2021_435_MOESM7_ESM.zip › 12983_2021_435_MOESM6_ESM/Additional file 45.pdf]

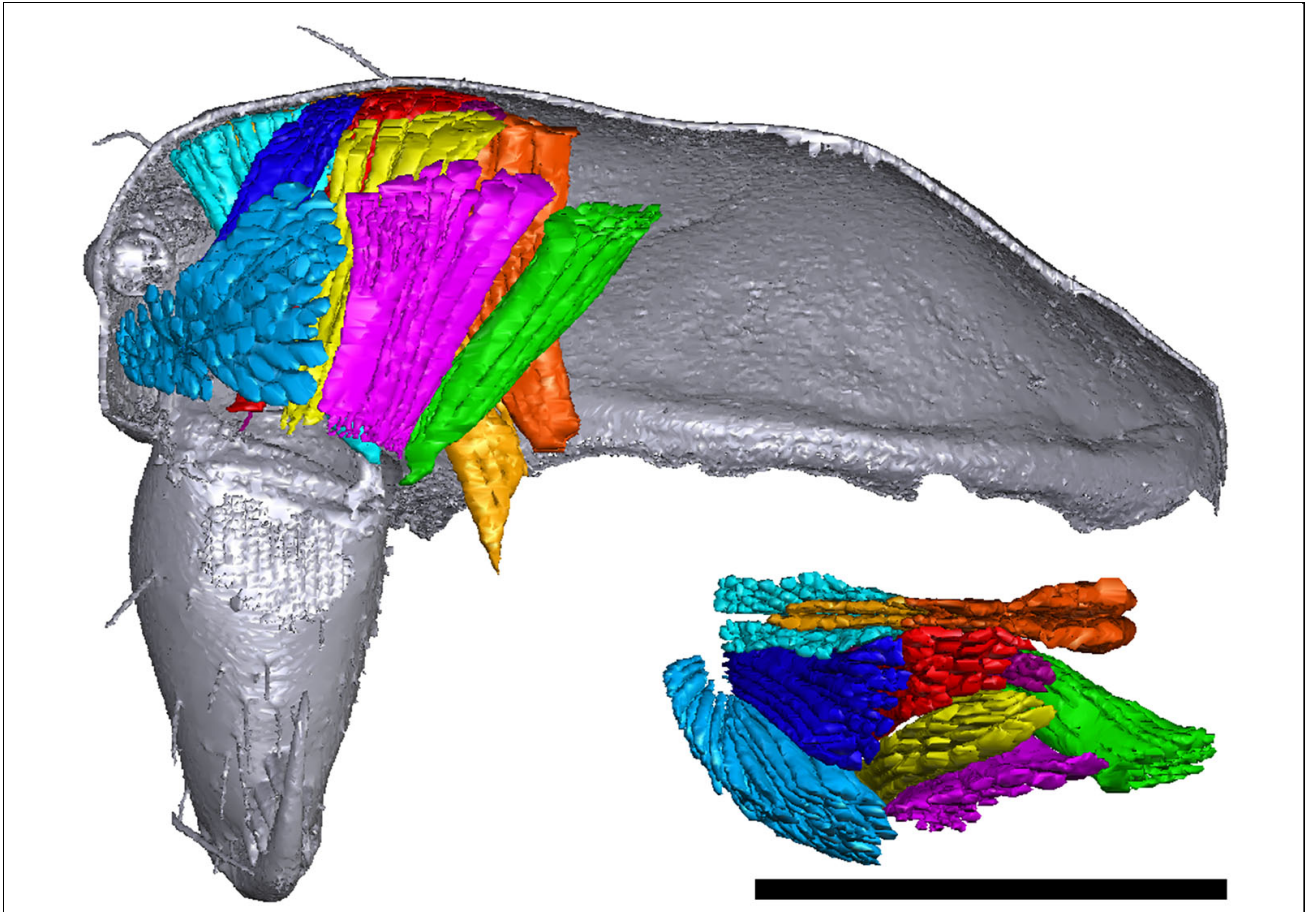

Additional file 46. Interactive 3D image of *Mitrager noordami* female prosoma (Fig. 12G).

Supplement: Supplementary file 7 — Additional file 7. Interactive 3D images of Figs. 12A-L. [file 12983_2021_435_MOESM7_ESM.zip › 12983_2021_435_MOESM6_ESM/Additional file 46.pdf]

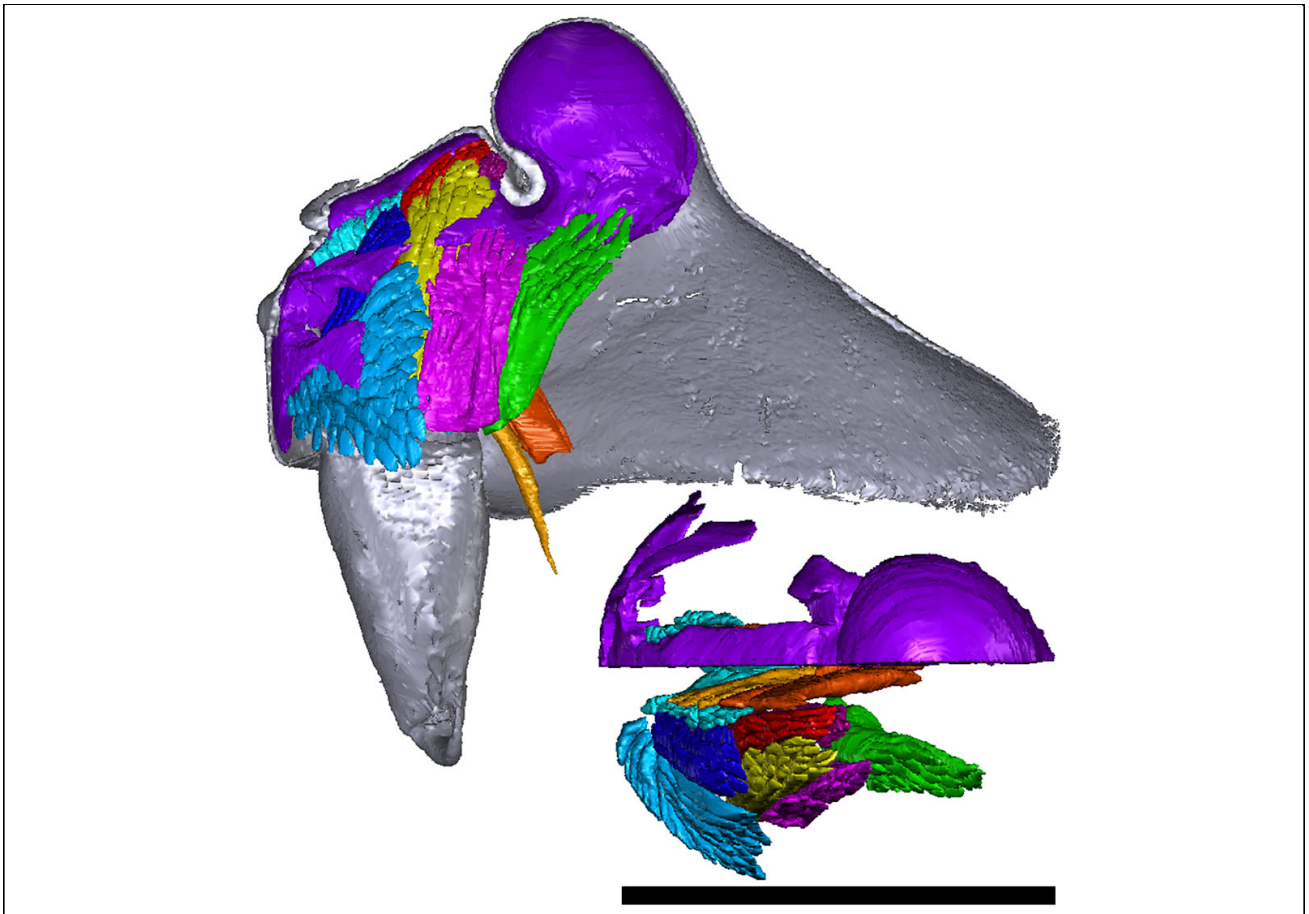

Additional file 47. Interactive 3D image of *Mitrager cornuta* male prosoma (Fig. 12H).

Supplement: Supplementary file 7 — Additional file 7. Interactive 3D images of Figs. 12A-L. [file 12983_2021_435_MOESM7_ESM.zip › 12983_2021_435_MOESM6_ESM/Additional file 47.pdf]

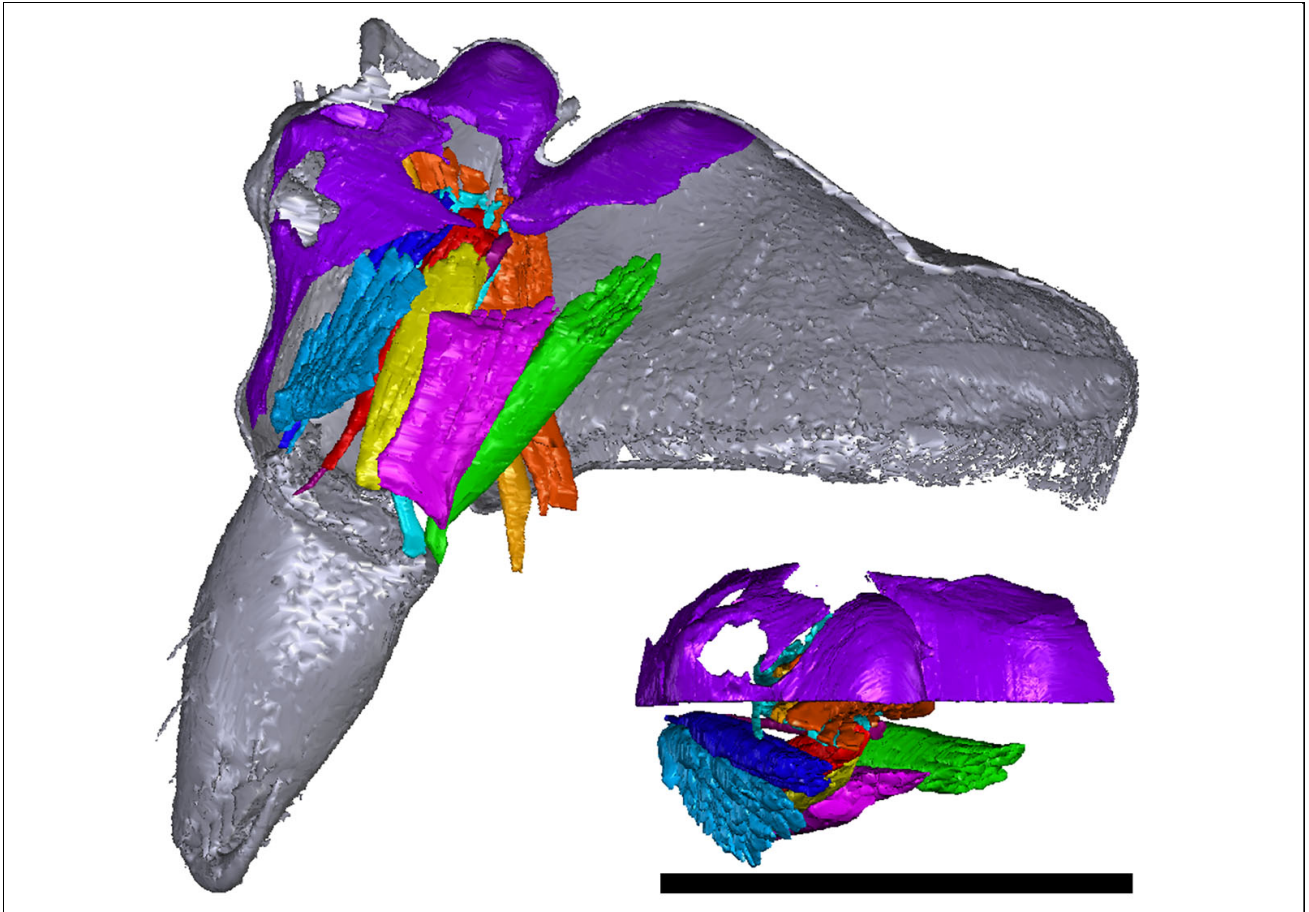

Additional file 48. Interactive 3D image of *Mitrager villosa* male prosoma (Fig. 12l).

Supplement: Supplementary file 7 — Additional file 7. Interactive 3D images of Figs. 12A-L. [file 12983_2021_435_MOESM7_ESM.zip › 12983_2021_435_MOESM6_ESM/Additional file 48.pdf]

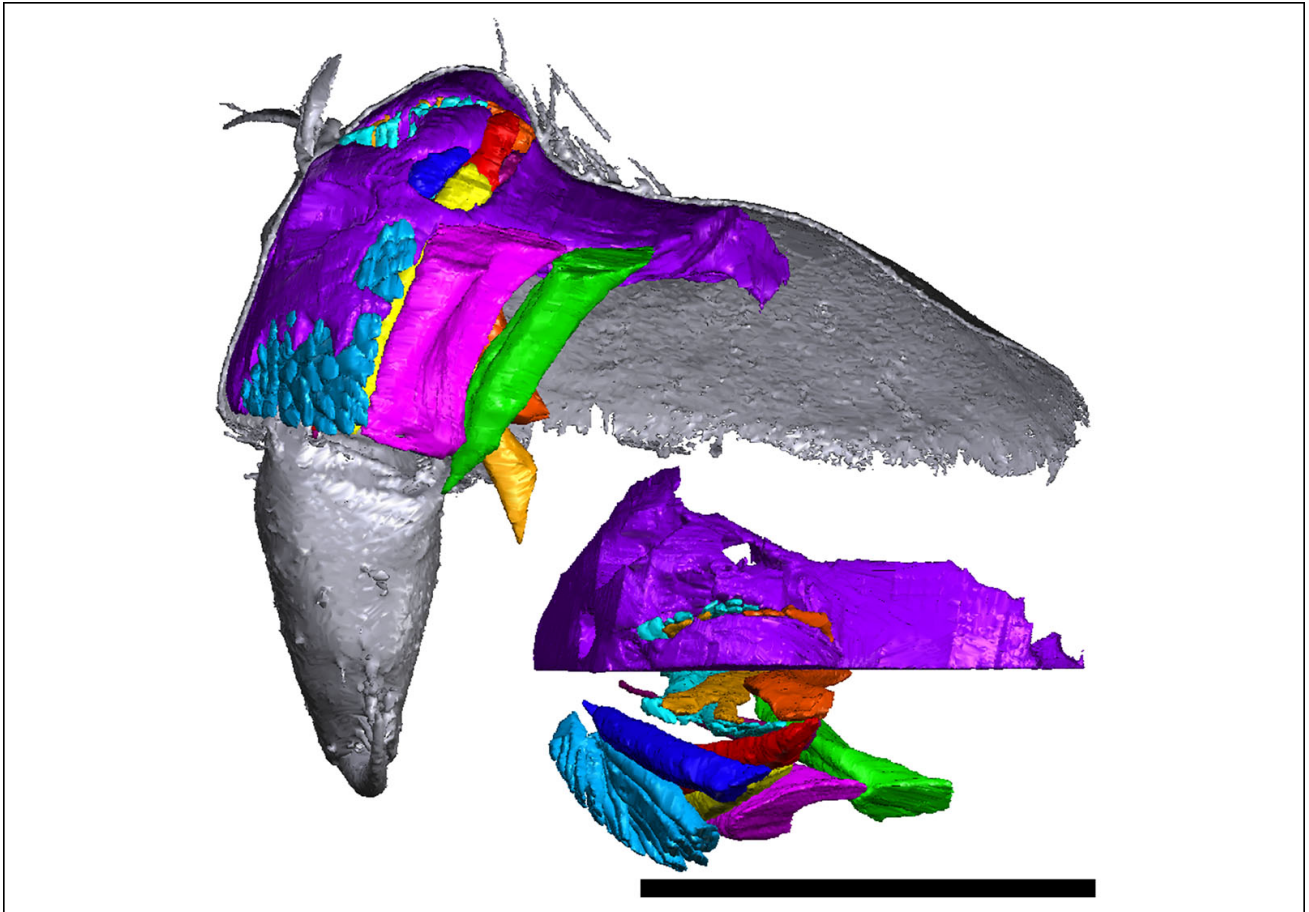

Additional file 49. Interactive 3D image of *Mitrager angela* male prosoma (Fig. 12J).

Supplement: Supplementary file 7 — Additional file 7. Interactive 3D images of Figs. 12A-L. [file 12983_2021_435_MOESM7_ESM.zip › 12983_2021_435_MOESM6_ESM/Additional file 49.pdf]

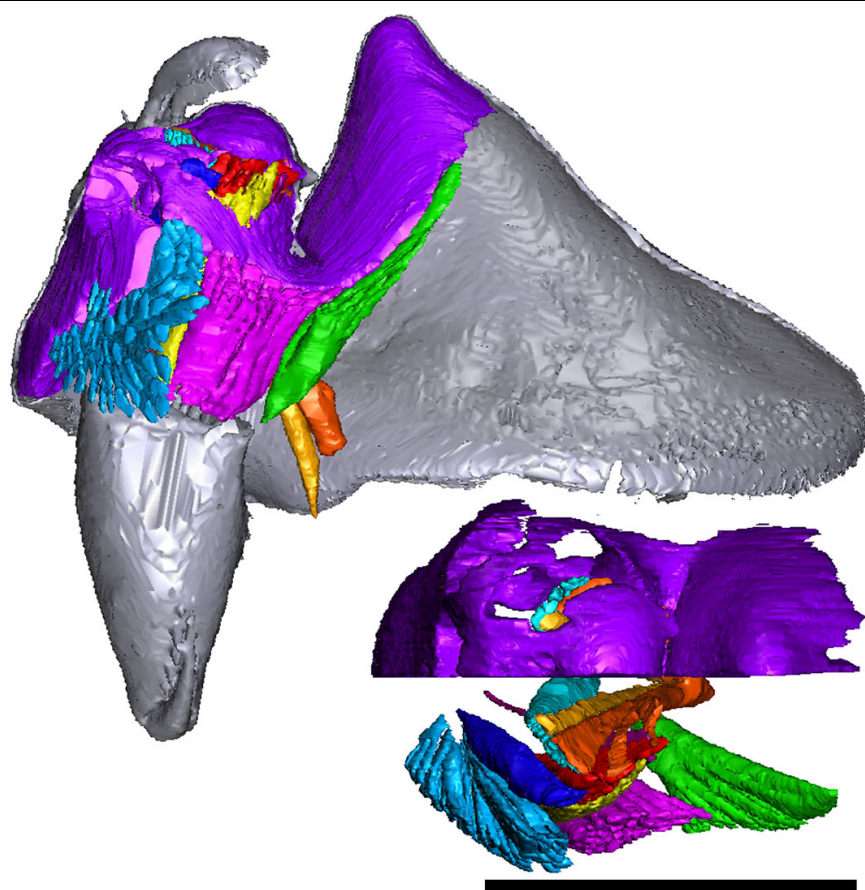

Additional file 50. Interactive 3D image of *Mitrager coronata* male prosoma (Fig. 12K).

Supplement: Supplementary file 7 — Additional file 7. Interactive 3D images of Figs. 12A-L. [file 12983_2021_435_MOESM7_ESM.zip › 12983_2021_435_MOESM6_ESM/Additional file 50.pdf]

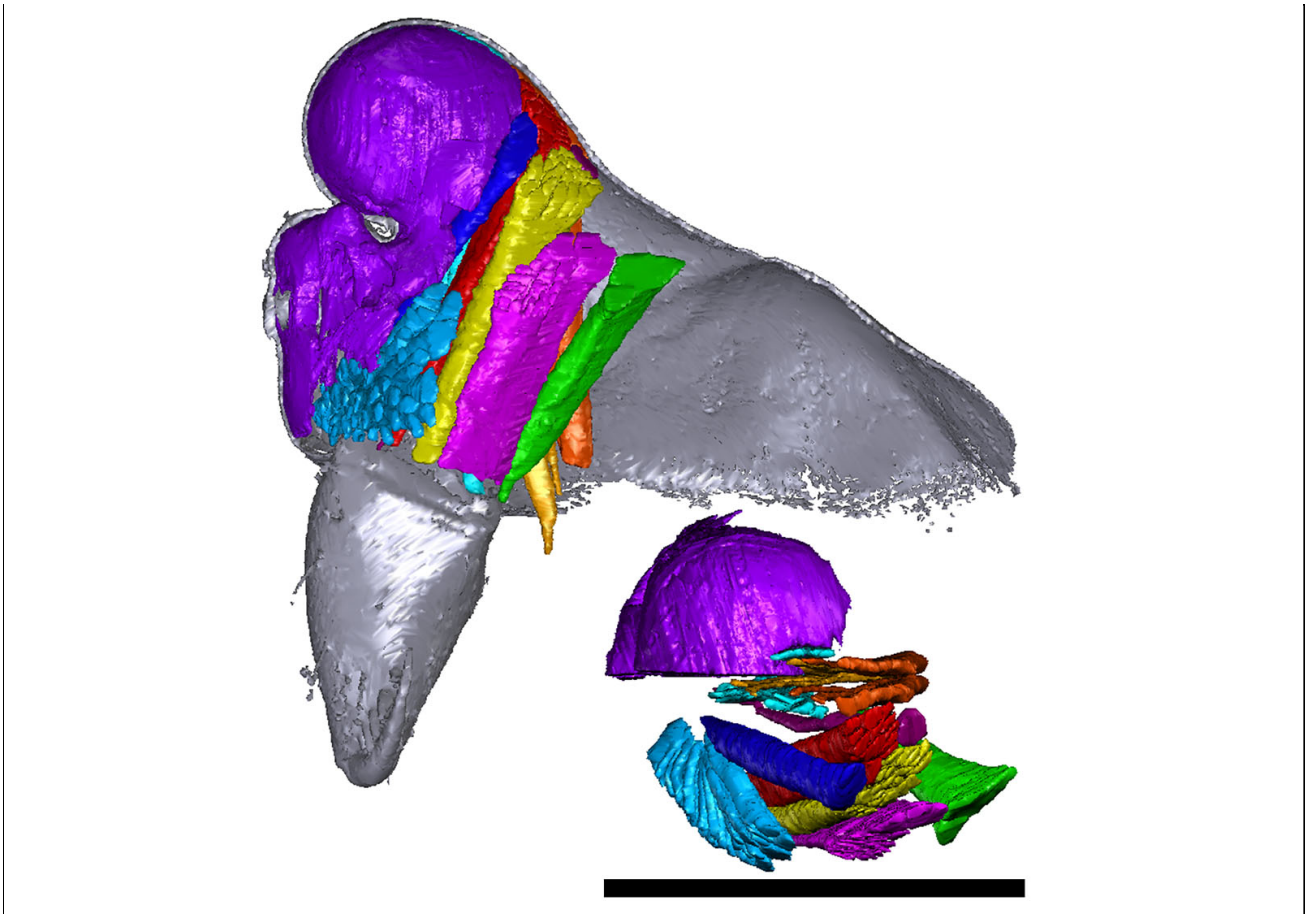

Additional file 51. Interactive 3D image of *Mitrager sexoculorum* male prosoma (Fig. 12L).

Supplement: Supplementary file 7 — Additional file 7. Interactive 3D images of Figs. 12A-L. [file 12983_2021_435_MOESM7_ESM.zip › 12983_2021_435_MOESM6_ESM/Additional file 51.pdf]

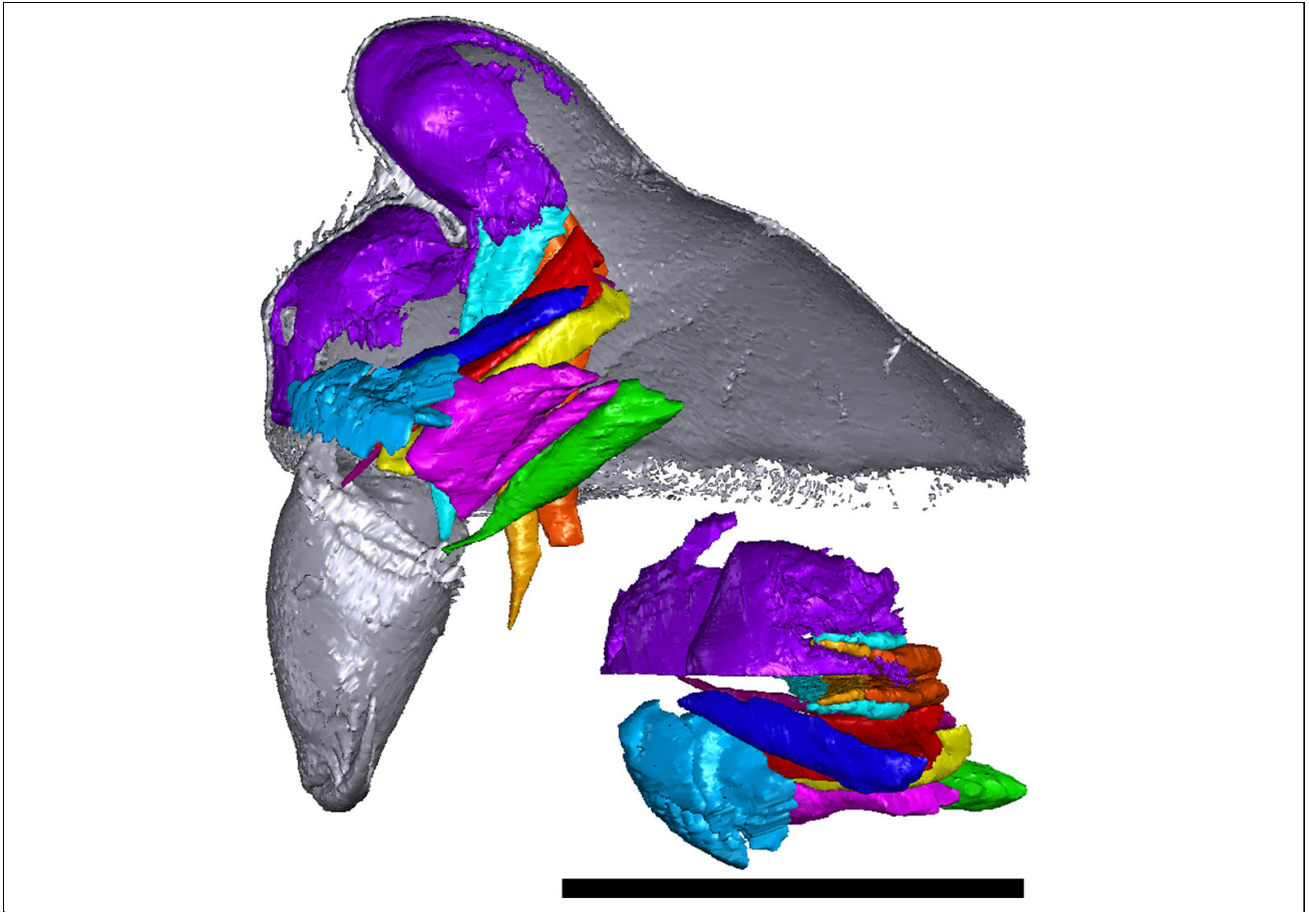

Additional file 52. Interactive 3D image of *Mitrager lineata* male prosoma (Fig. 13A).

Supplement: Supplementary file 8 — Additional file 8. Interactive 3D images of Figs. 13A-L. [file 12983_2021_435_MOESM8_ESM.zip › 12983_2021_435_MOESM7_ESM/Additional file 52.pdf]

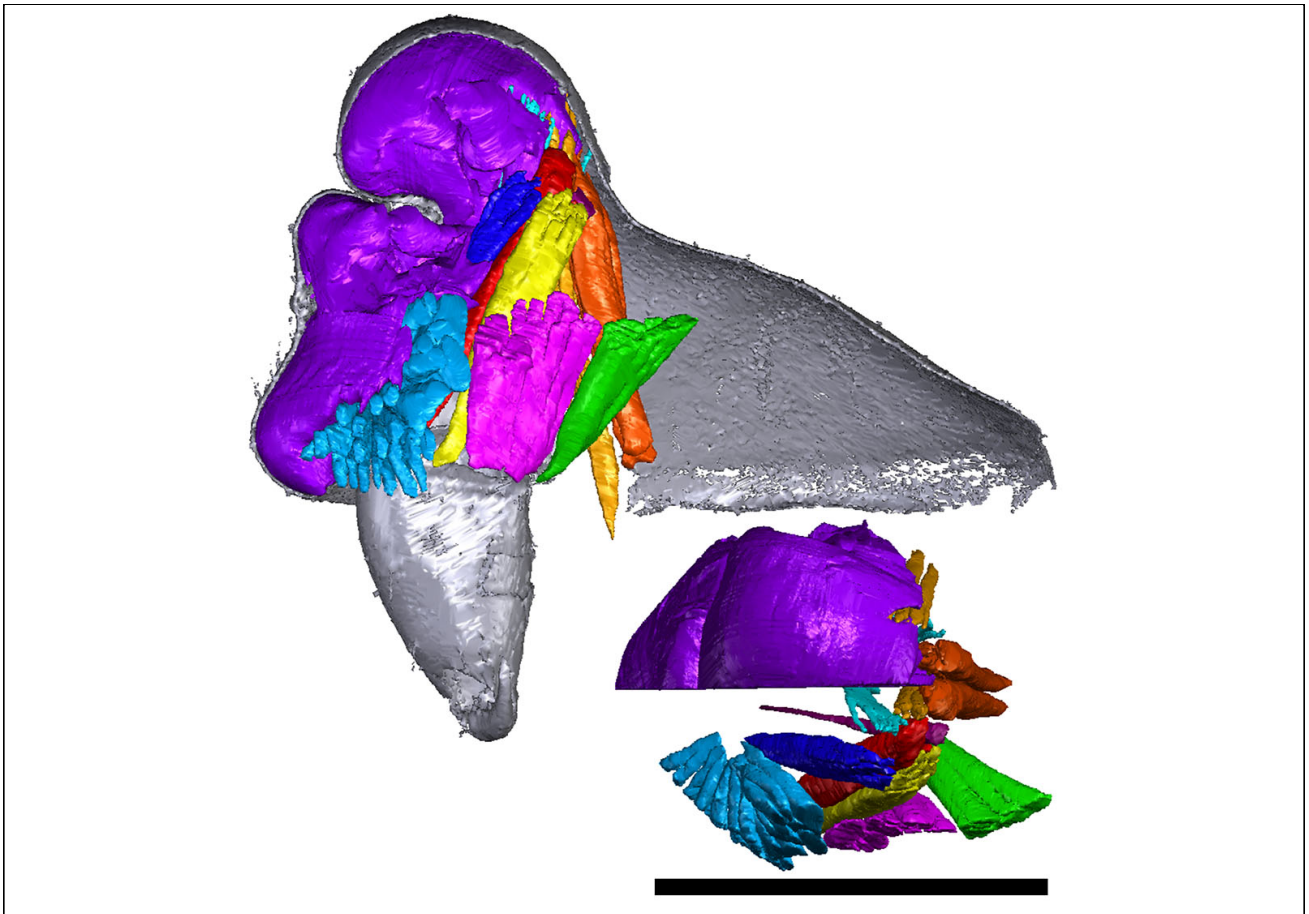

Additional file 53. Interactive 3D image of *Mitrager dismodicoides* male prosoma (Fig. 13B).

Supplement: Supplementary file 8 — Additional file 8. Interactive 3D images of Figs. 13A-L. [file 12983_2021_435_MOESM8_ESM.zip › 12983_2021_435_MOESM7_ESM/Additional file 53.pdf]

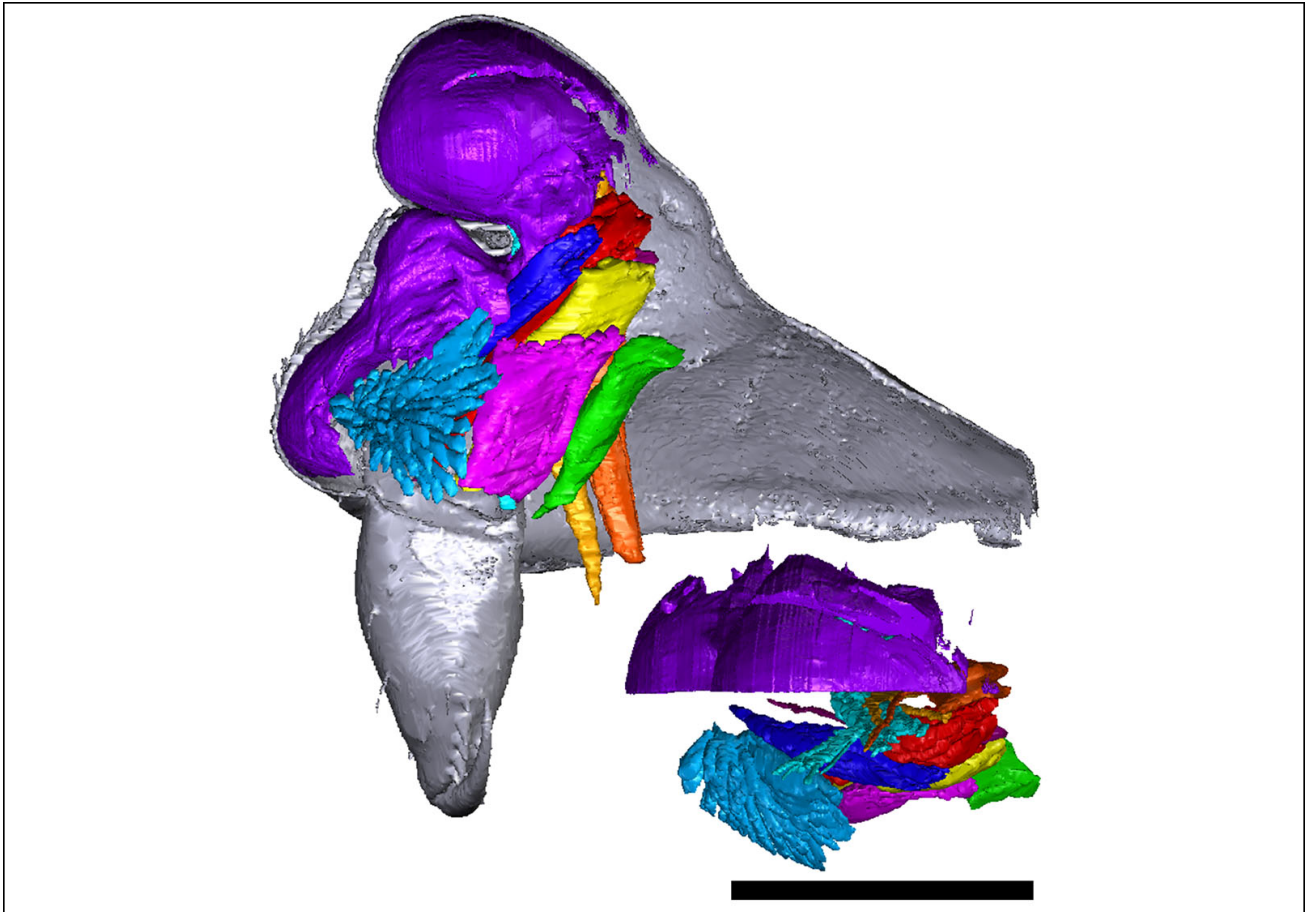

Additional file 54. Interactive 3D image of *Mitrager tholusa* male prosoma (Fig. 13C).

Supplement: Supplementary file 8 — Additional file 8. Interactive 3D images of Figs. 13A-L. [file 12983_2021_435_MOESM8_ESM.zip › 12983_2021_435_MOESM7_ESM/Additional file 54.pdf]

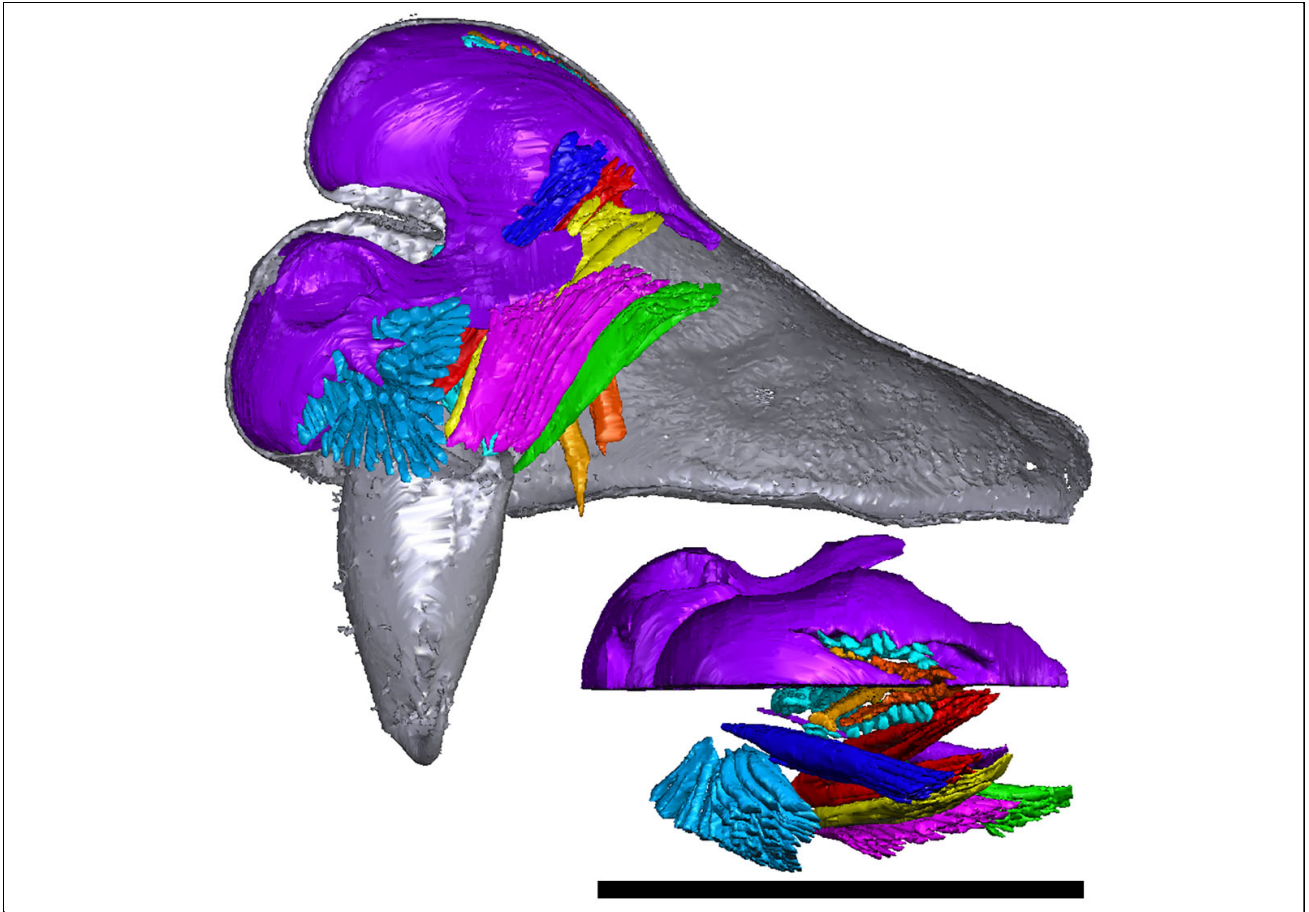

Additional file 55. Interactive 3D image of *Mitrager lucida* male prosoma (Fig. 13D).

Supplement: Supplementary file 8 — Additional file 8. Interactive 3D images of Figs. 13A-L. [file 12983_2021_435_MOESM8_ESM.zip › 12983_2021_435_MOESM7_ESM/Additional file 55.pdf]

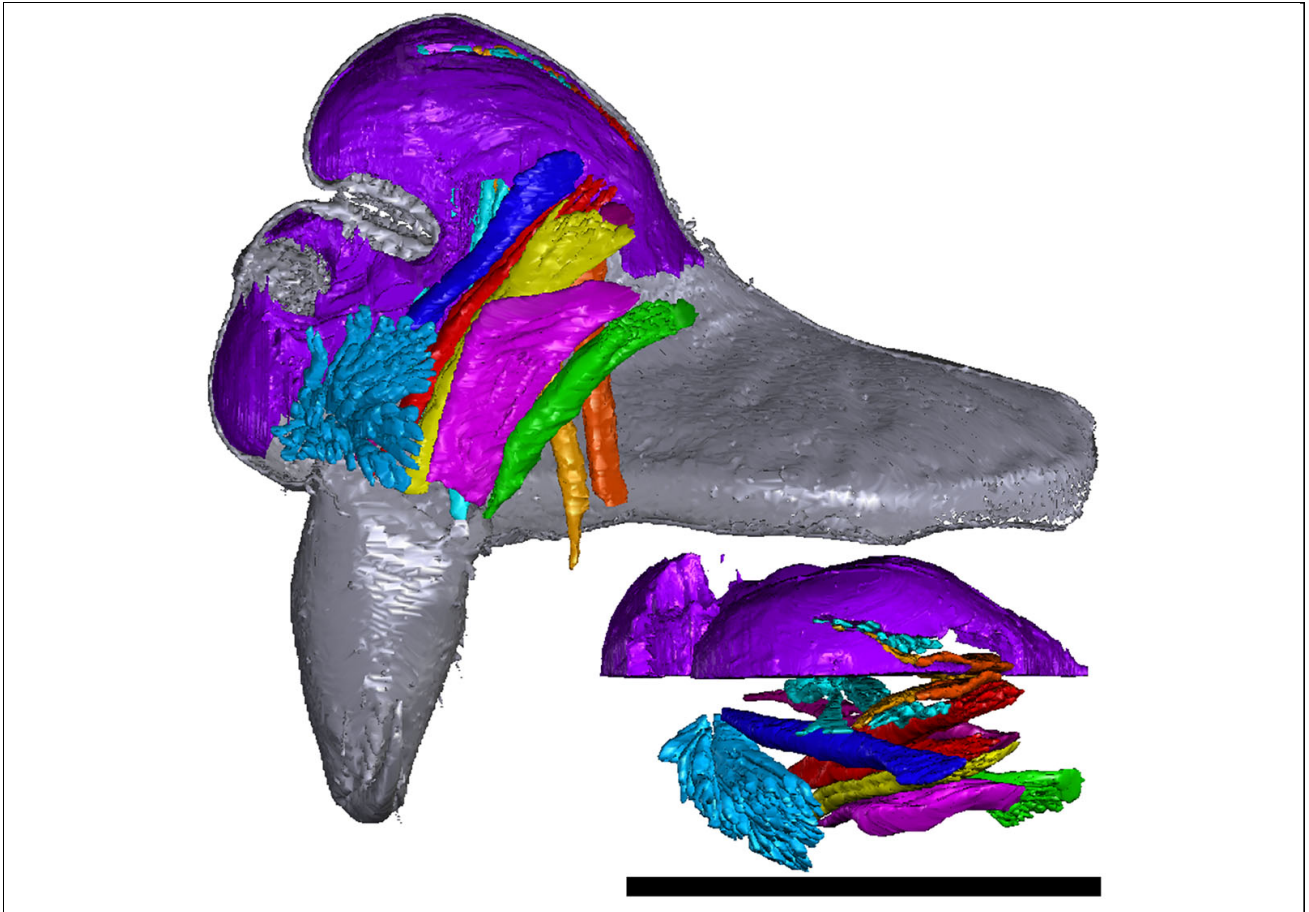

Additional file 56. Interactive 3D image of *Mitragery sexoculata* male prosoma (Fig. 13E).

Supplement: Supplementary file 8 — Additional file 8. Interactive 3D images of Figs. 13A-L. [file 12983_2021_435_MOESM8_ESM.zip › 12983_2021_435_MOESM7_ESM/Additional file 56.pdf]

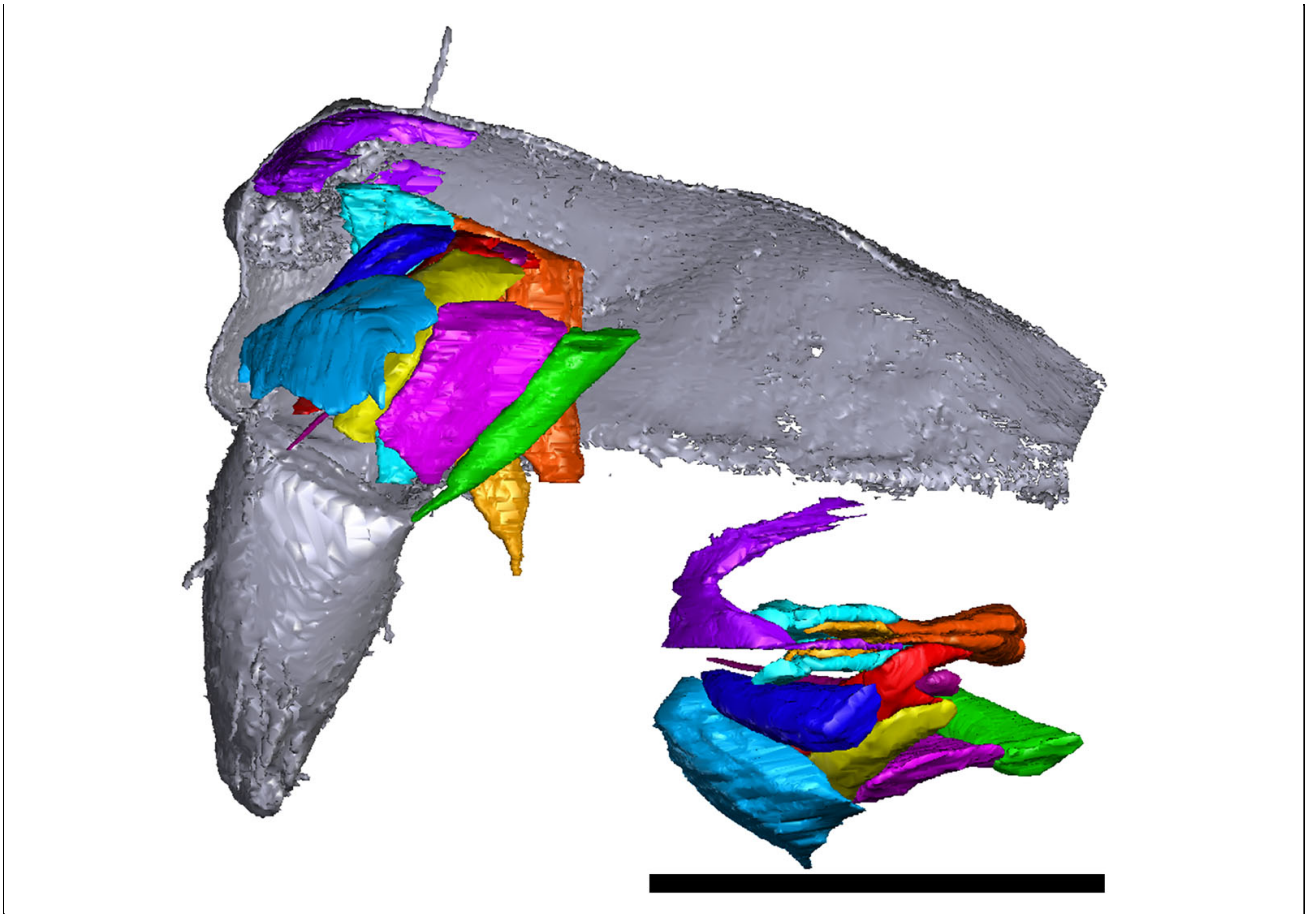

Additional file 57. Interactive 3D image of *Mitrager unicolor* male prosoma (Fig. 13F).

Supplement: Supplementary file 8 — Additional file 8. Interactive 3D images of Figs. 13A-L. [file 12983_2021_435_MOESM8_ESM.zip › 12983_2021_435_MOESM7_ESM/Additional file 57.pdf]

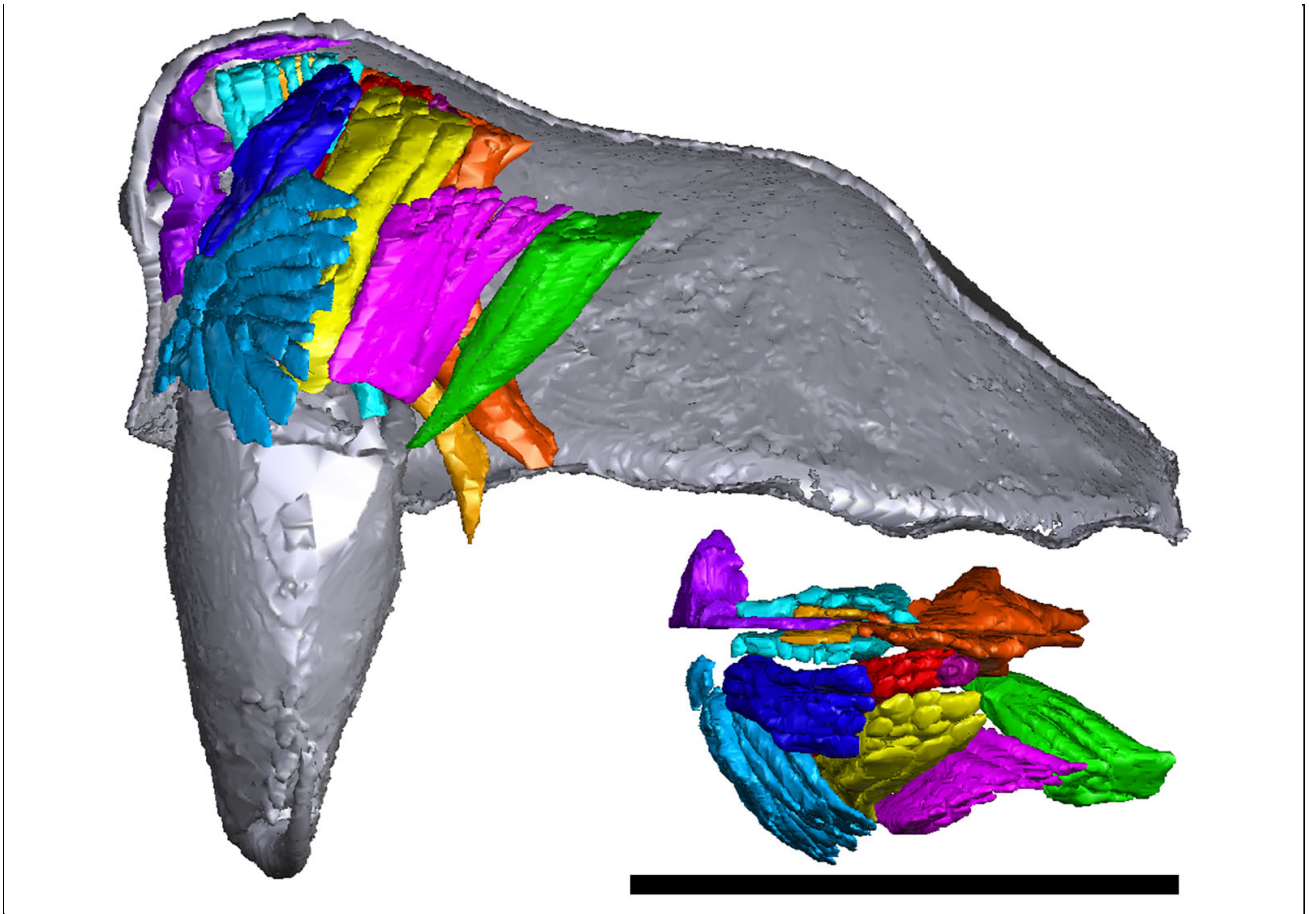

Additional file 58. Interactive 3D image of *Mitrager rustica* male prosoma (Fig. 13G).

Supplement: Supplementary file 8 — Additional file 8. Interactive 3D images of Figs. 13A-L. [file 12983_2021_435_MOESM8_ESM.zip › 12983_2021_435_MOESM7_ESM/Additional file 58.pdf]

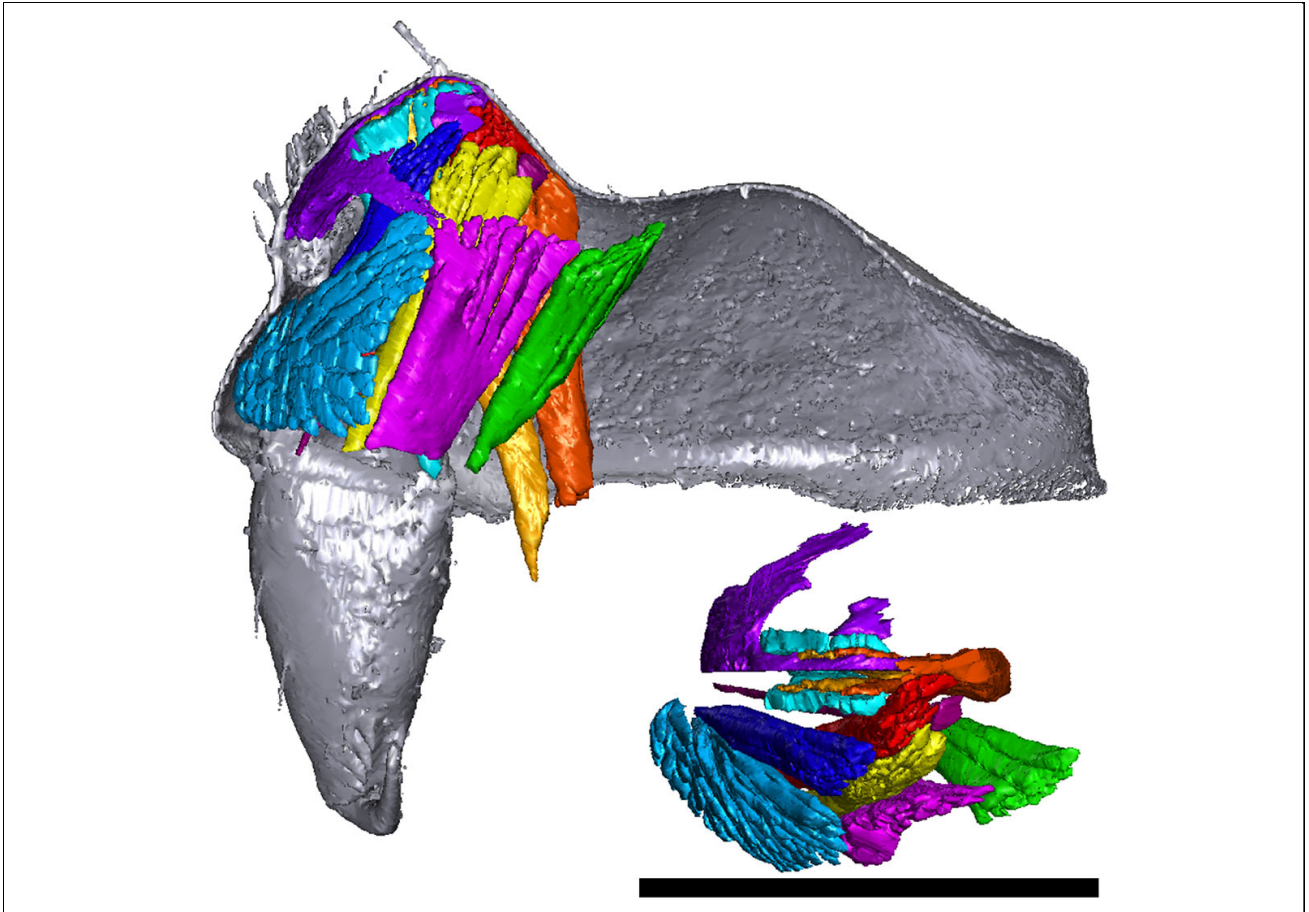

Additional file 59. Interactive 3D image of *Mitrager assueta* male prosoma (Fig. 13H).

Supplement: Supplementary file 8 — Additional file 8. Interactive 3D images of Figs. 13A-L. [file 12983_2021_435_MOESM8_ESM.zip › 12983_2021_435_MOESM7_ESM/Additional file 59.pdf]

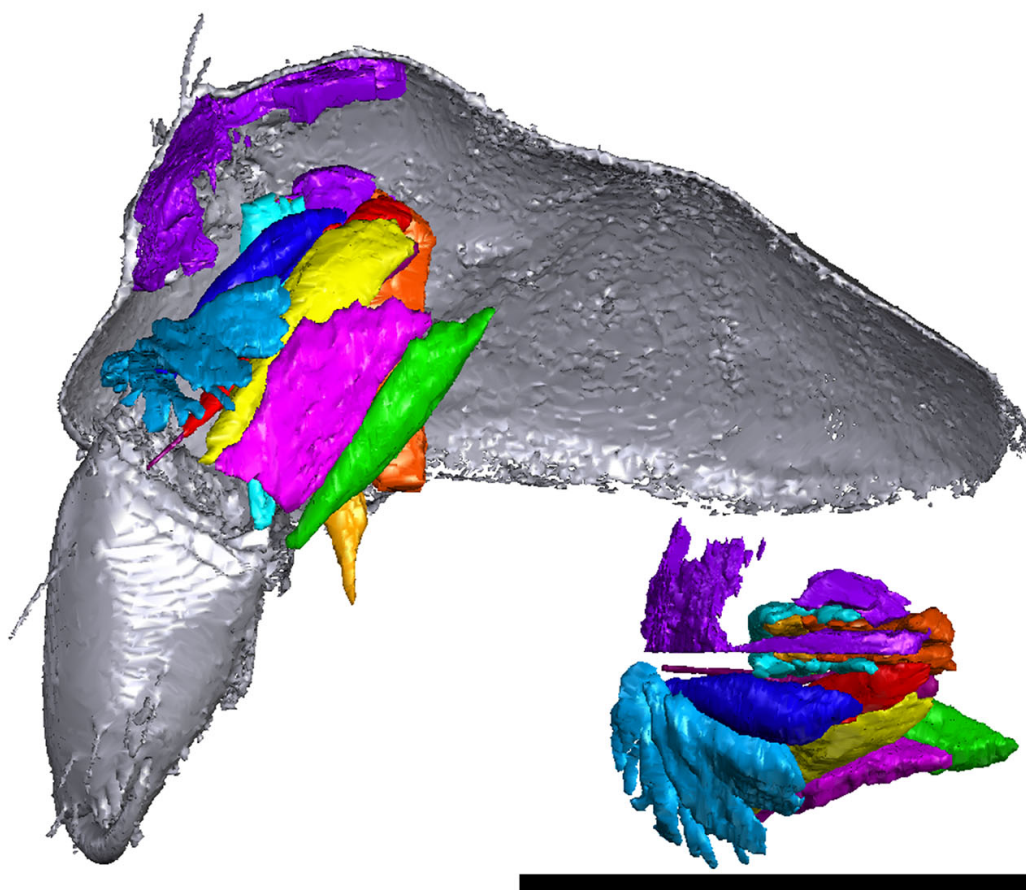

Additional file 60. Interactive 3D image of *Mitrager malearmata* male prosoma (Fig. 13l).

Supplement: Supplementary file 8 — Additional file 8. Interactive 3D images of Figs. 13A-L. [file 12983_2021_435_MOESM8_ESM.zip › 12983_2021_435_MOESM7_ESM/Additional file 60.pdf]

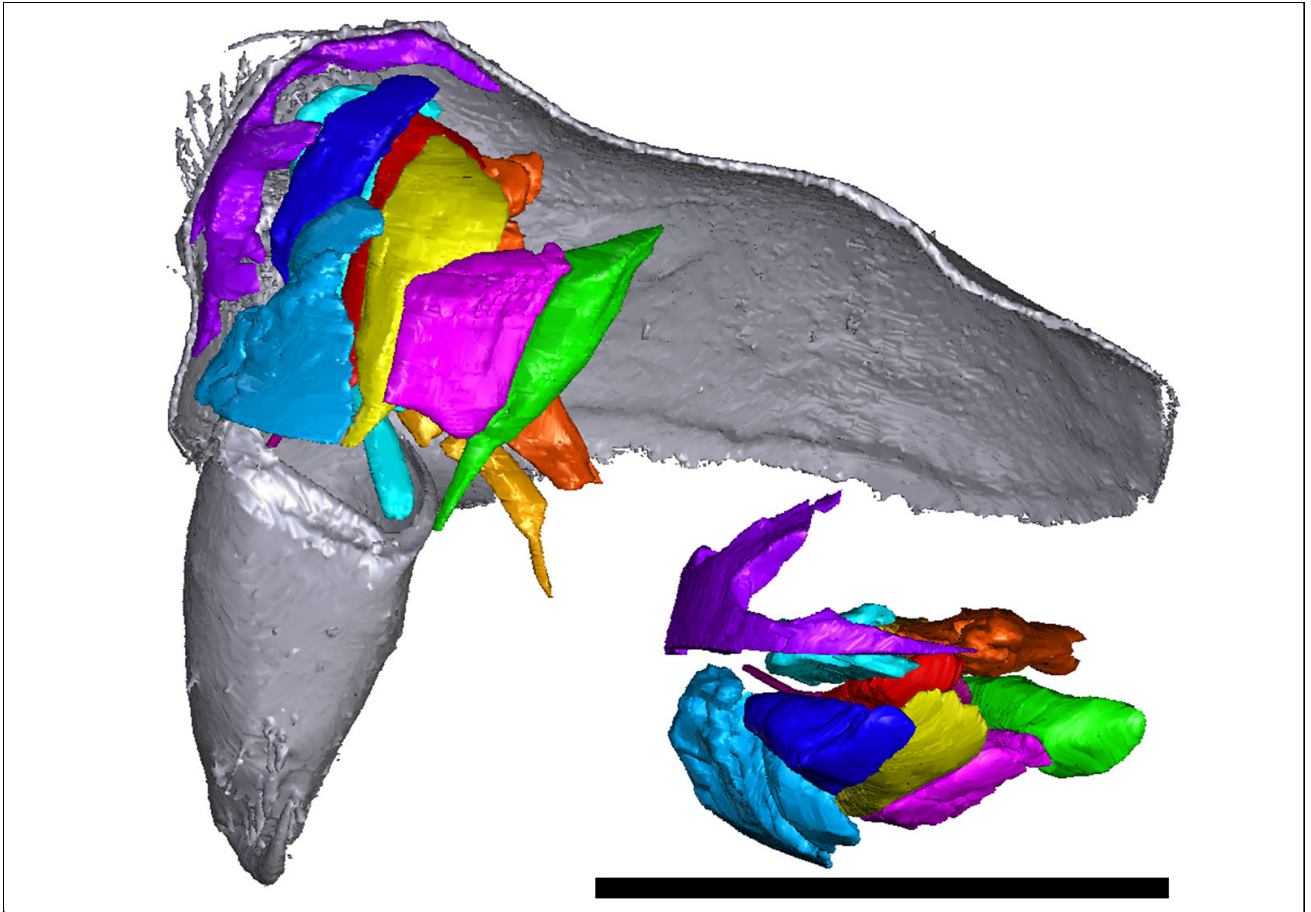

Additional file 61. Interactive 3D image of *Mitrager lopchu* male prosoma (Fig. 13J).

Supplement: Supplementary file 8 — Additional file 8. Interactive 3D images of Figs. 13A-L. [file 12983_2021_435_MOESM8_ESM.zip › 12983_2021_435_MOESM7_ESM/Additional file 61.pdf]

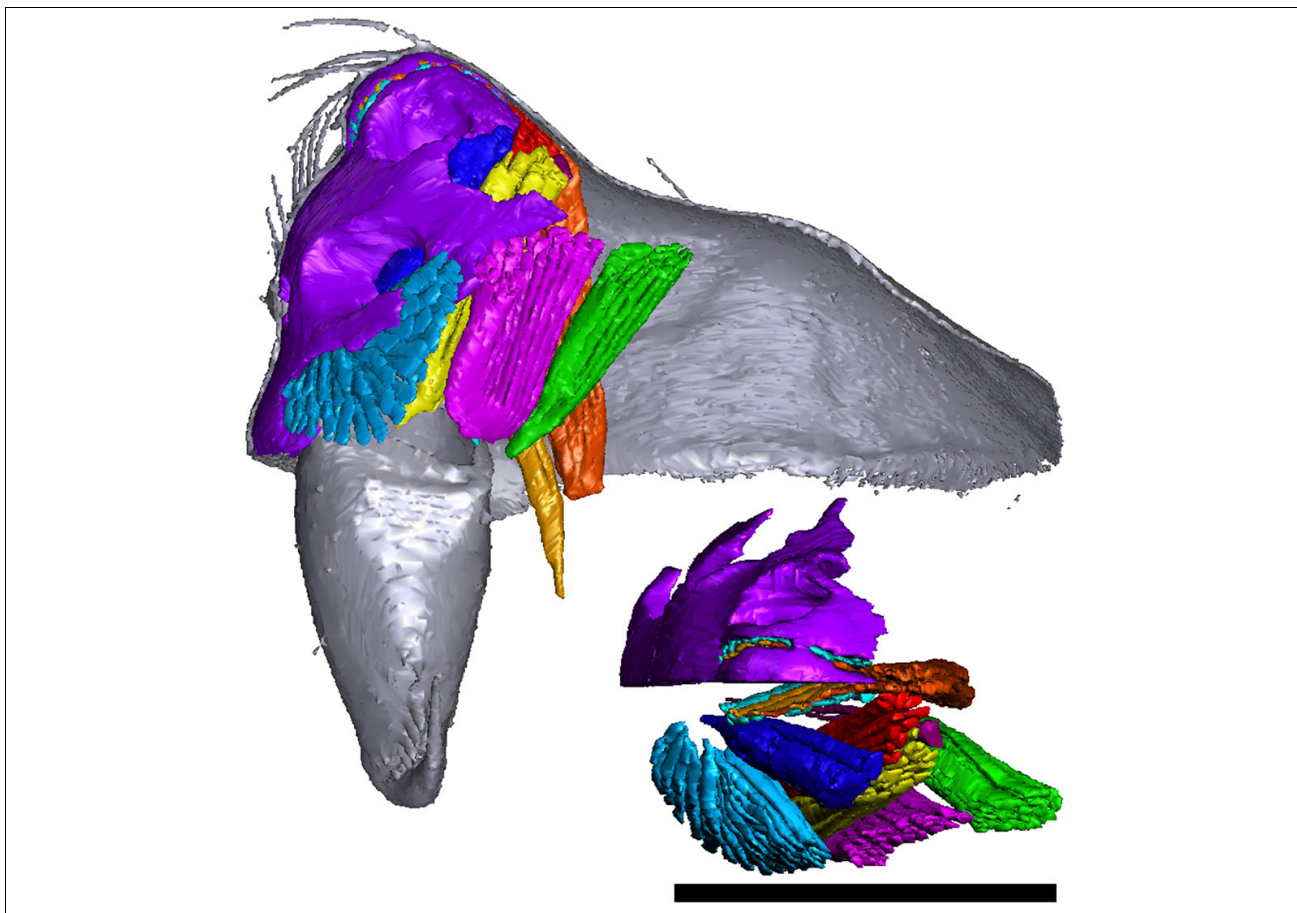

Additional file 62. Interactive 3D image of *Mitrager falciferoides* male prosoma (Fig. 13K).

Supplement: Supplementary file 8 — Additional file 8. Interactive 3D images of Figs. 13A-L. [file 12983_2021_435_MOESM8_ESM.zip › 12983_2021_435_MOESM7_ESM/Additional file 62.pdf]

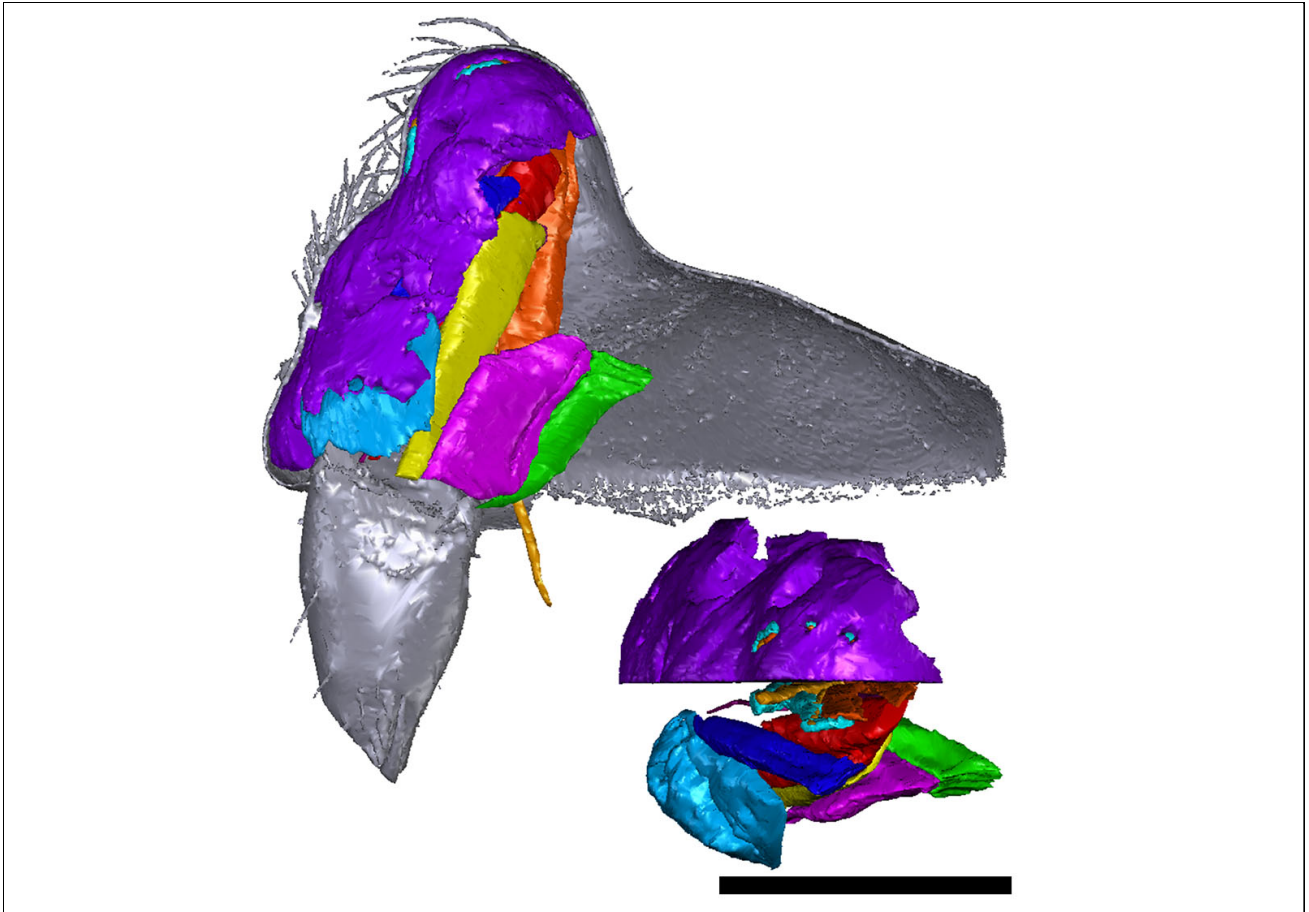

Additional file 63. Interactive 3D image of *Mitrager falcifer* male prosoma (Fig. 13L).

Supplement: Supplementary file 8 — Additional file 8. Interactive 3D images of Figs. 13A-L. [file 12983_2021_435_MOESM8_ESM.zip › 12983_2021_435_MOESM7_ESM/Additional file 63.pdf]

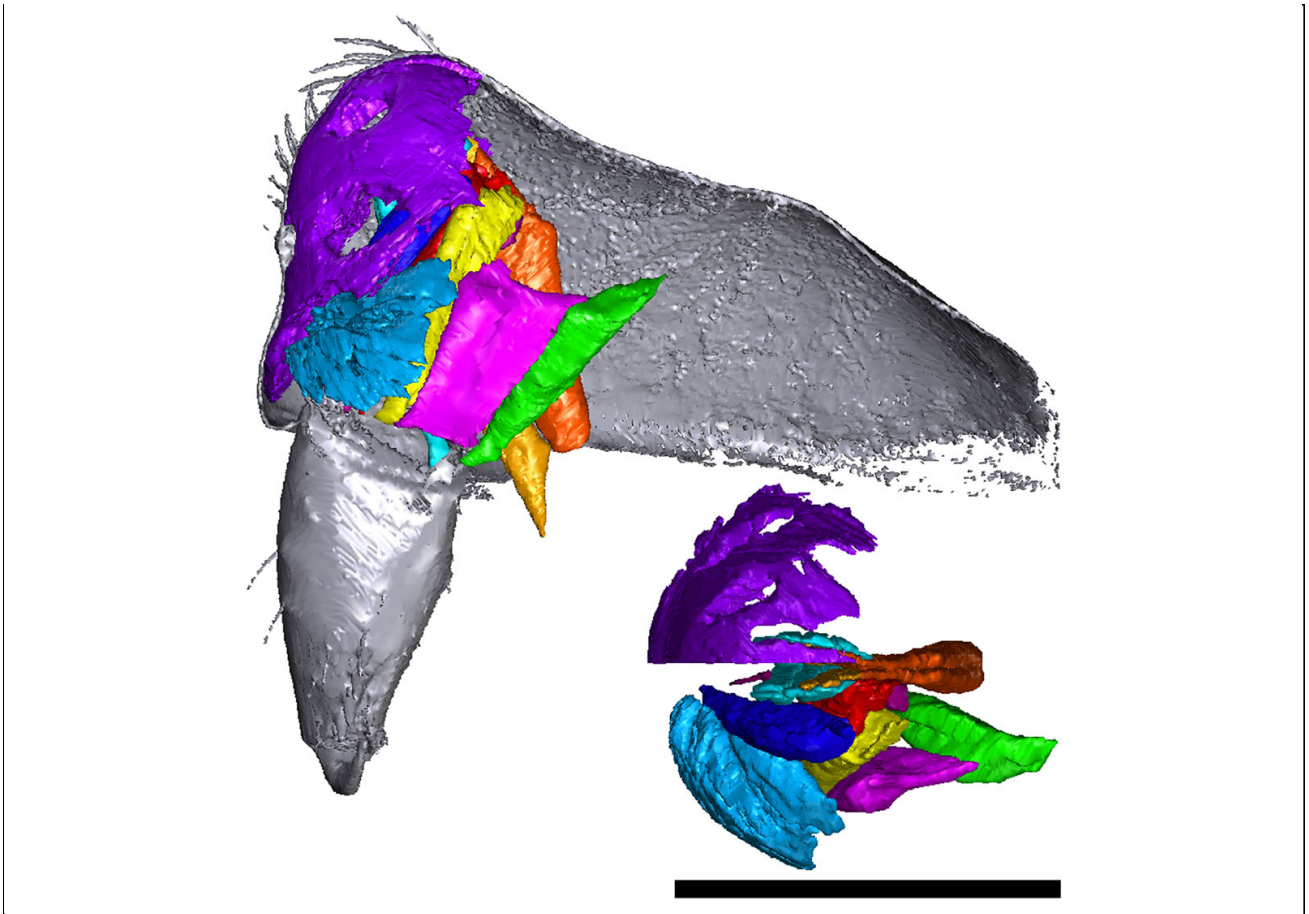

Additional file 64. Interactive 3D image of *Mitrager modesta* male prosoma (Fig. 14A).

Supplement: Supplementary file 9 — Additional file 9. Interactive 3D images of Figs. 14A-L. [file 12983_2021_435_MOESM9_ESM.zip › 12983_2021_435_MOESM8_ESM/Additional file 64.pdf]

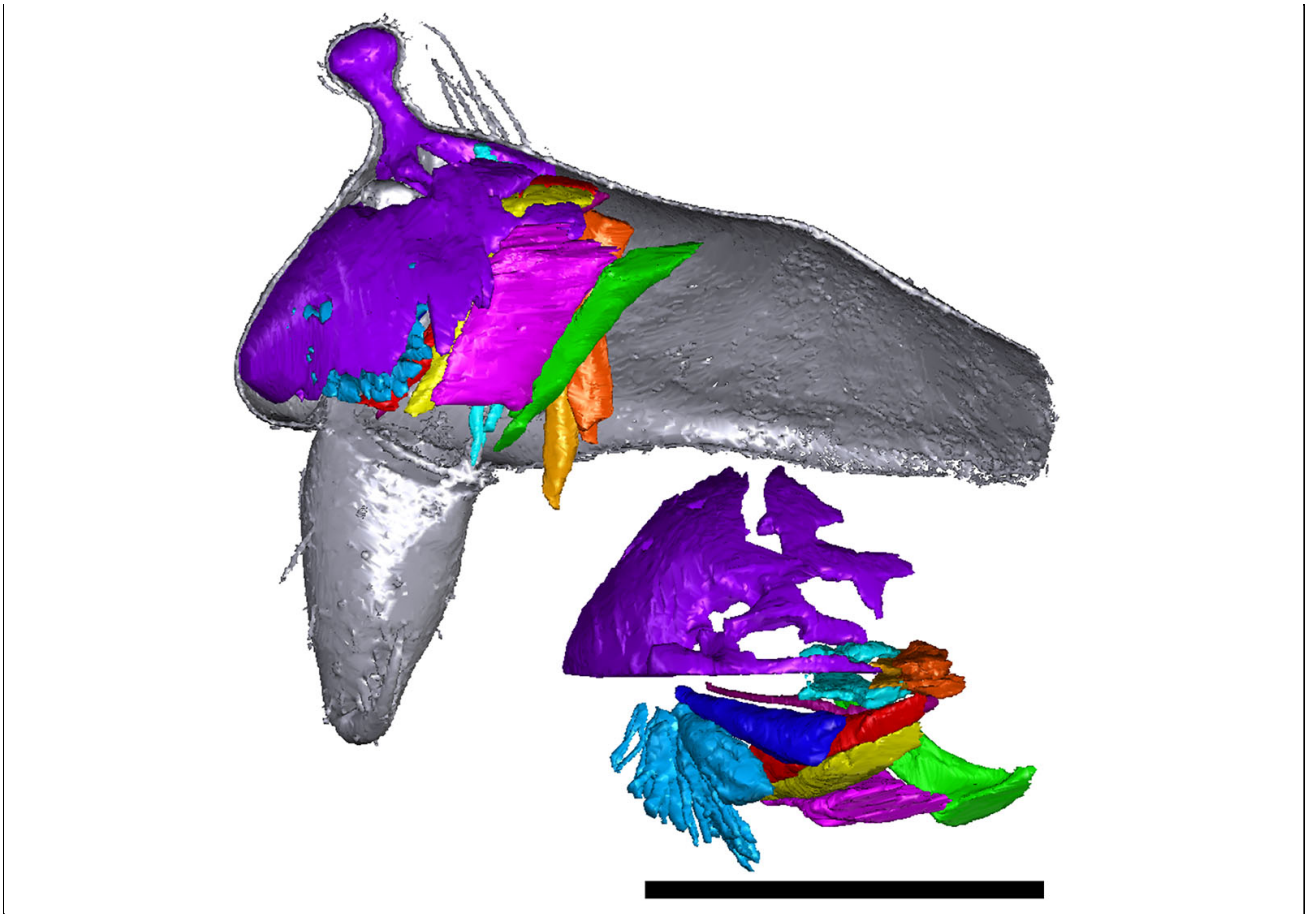

Additional file 65. Interactive 3D image of *Mitrager savigniformis* male prosoma (Fig. 14B).

Supplement: Supplementary file 9 — Additional file 9. Interactive 3D images of Figs. 14A-L. [file 12983_2021_435_MOESM9_ESM.zip › 12983_2021_435_MOESM8_ESM/Additional file 65.pdf]

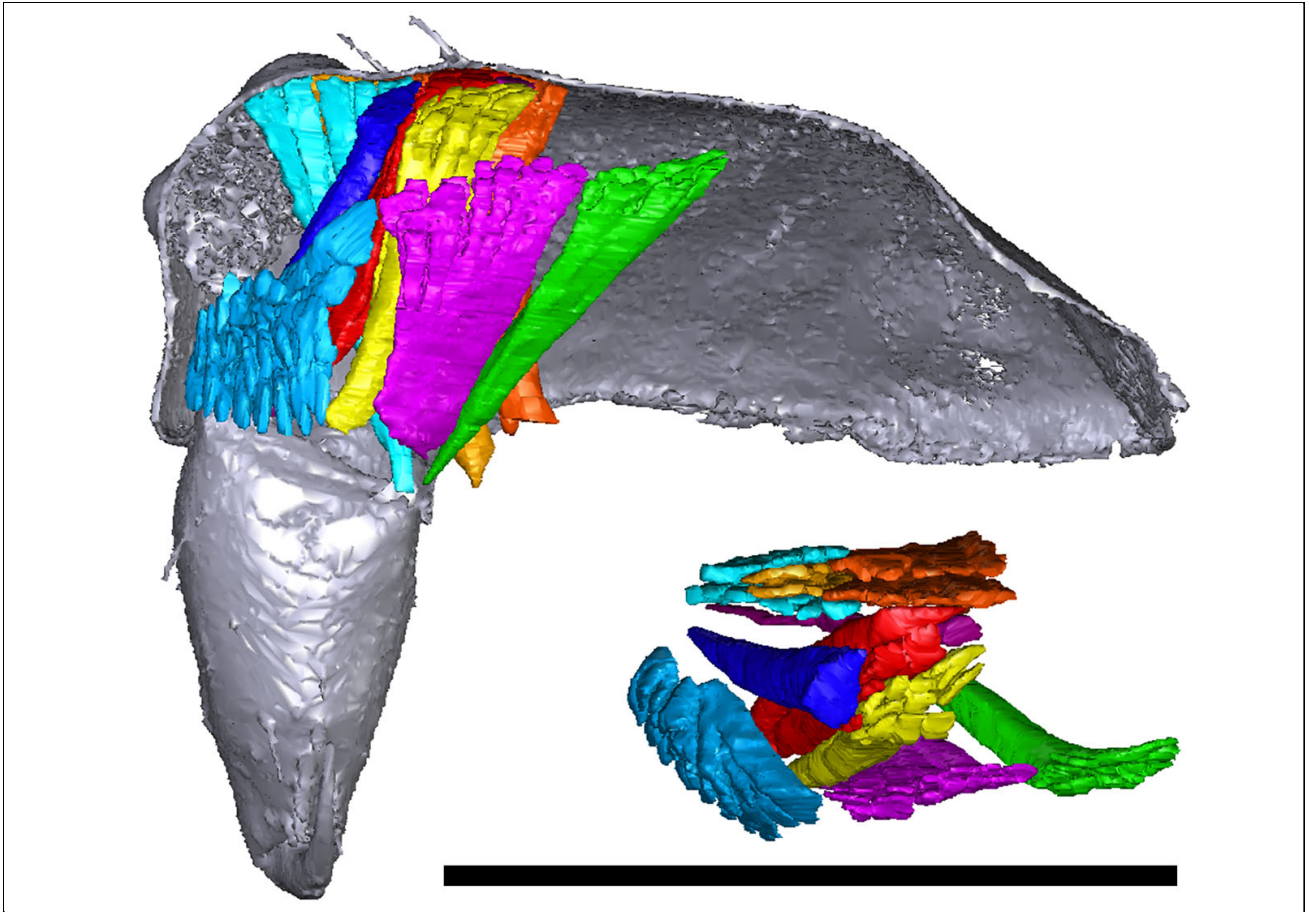

Additional file 66. Interactive 3D image of *Holmelgonia basalis* male prosoma (Fig. 14C).

Supplement: Supplementary file 9 — Additional file 9. Interactive 3D images of Figs. 14A-L. [file 12983_2021_435_MOESM9_ESM.zip › 12983_2021_435_MOESM8_ESM/Additional file 66.pdf]

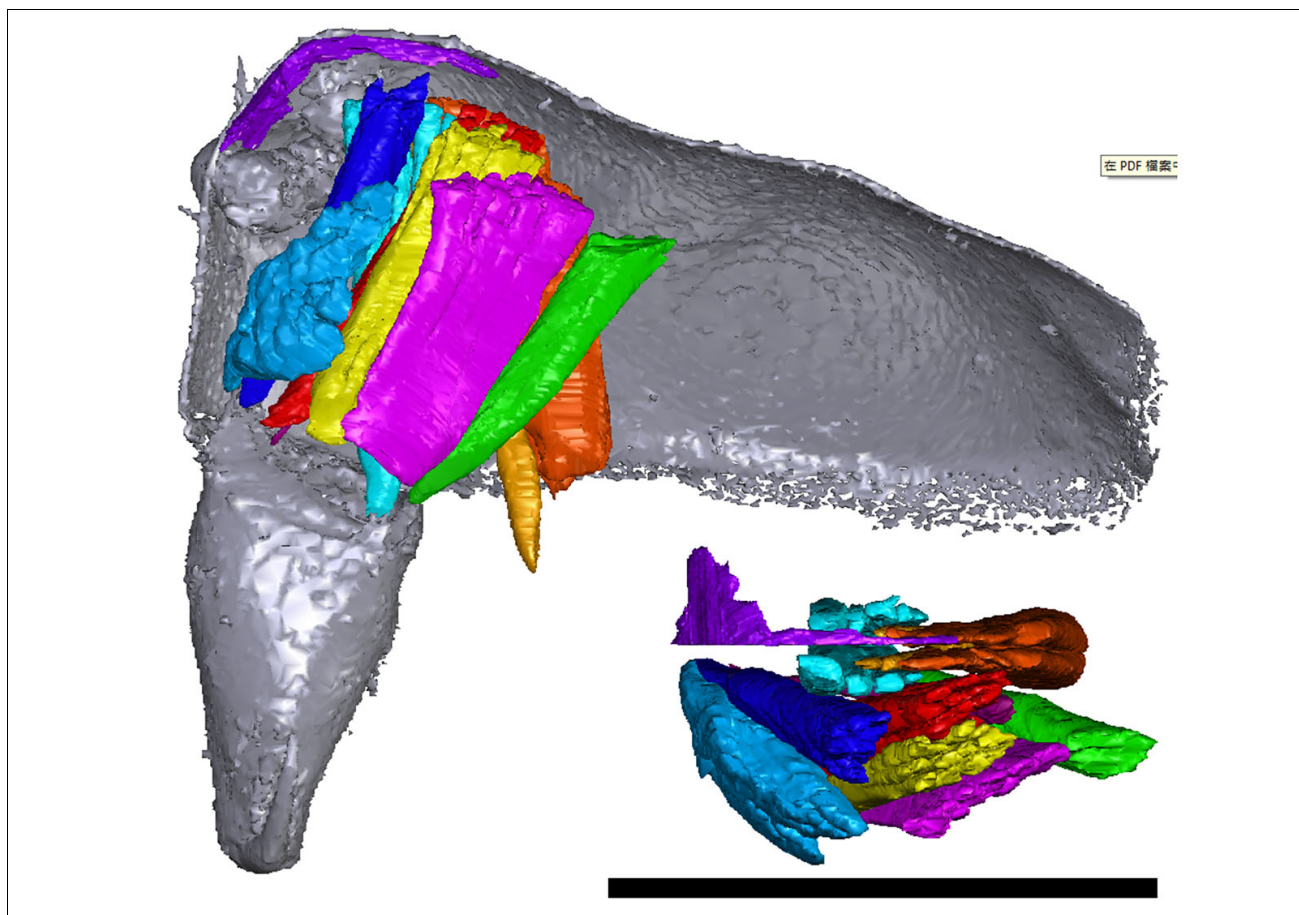

Additional file 67. Interactive 3D image of *Callitrichia holmi* male prosoma (Fig. 14D).

Supplement: Supplementary file 9 — Additional file 9. Interactive 3D images of Figs. 14A-L. [file 12983_2021_435_MOESM9_ESM.zip › 12983_2021_435_MOESM8_ESM/Additional file 67.pdf]

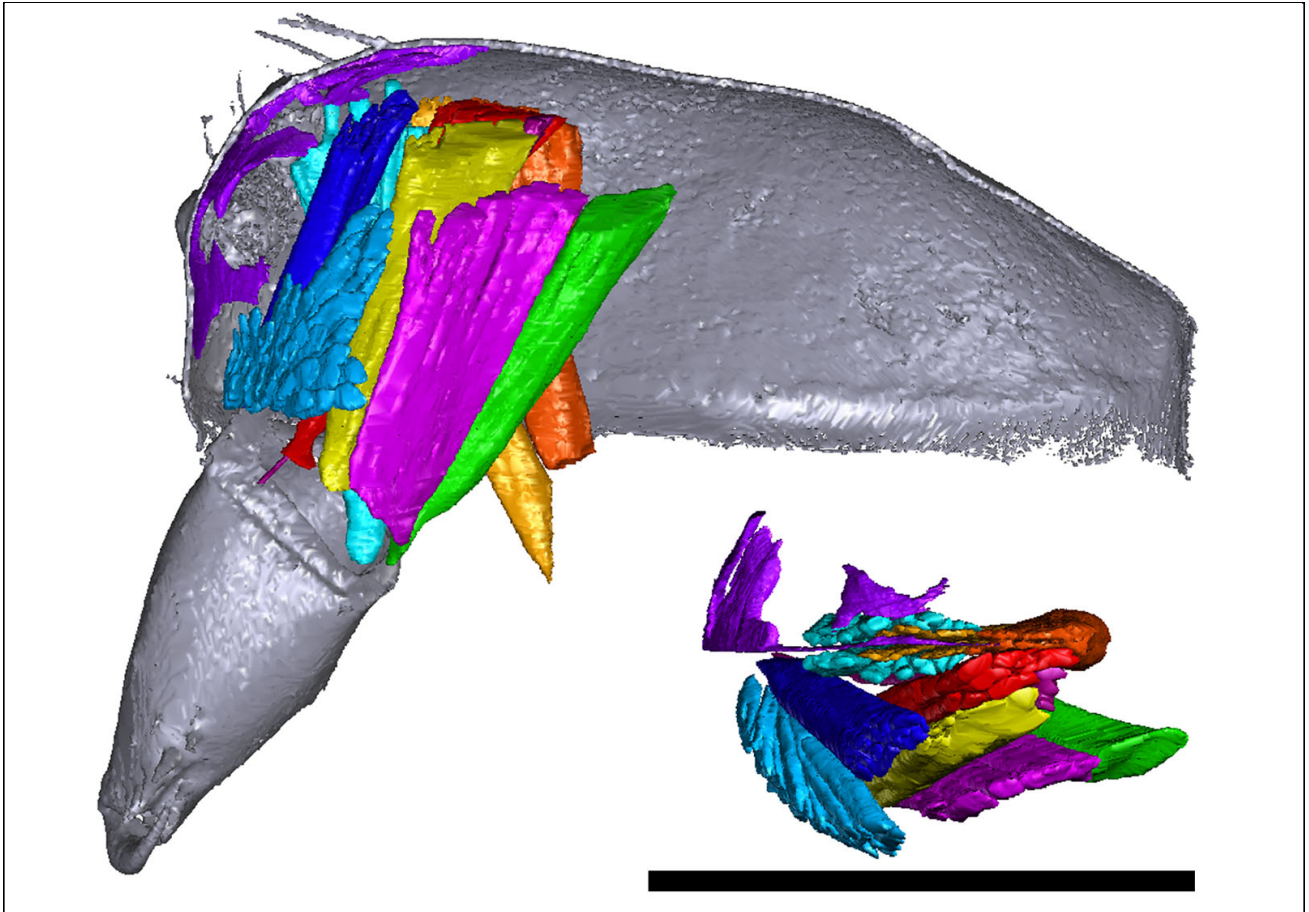

Additional file 68. Interactive 3D image of *Callitrichia picta* male prosoma (Fig. 14E).

Supplement: Supplementary file 9 — Additional file 9. Interactive 3D images of Figs. 14A-L. [file 12983_2021_435_MOESM9_ESM.zip › 12983_2021_435_MOESM8_ESM/Additional file 68.pdf]

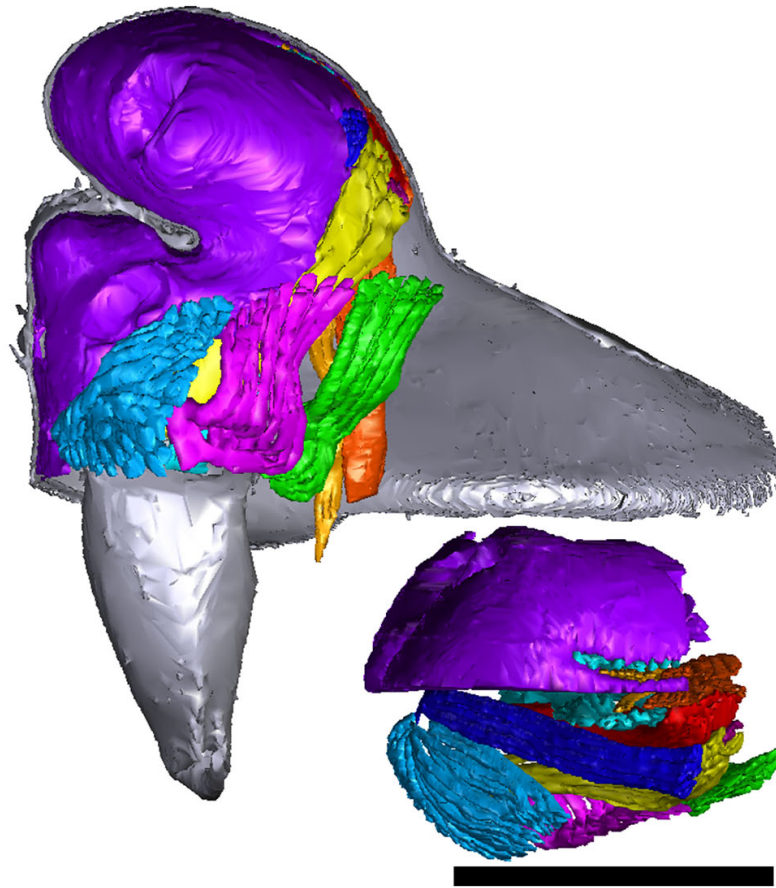

Additional file 69. Interactive 3D image of *Callitrichia gloriosa* male prosoma (Fig. 14F).

Supplement: Supplementary file 9 — Additional file 9. Interactive 3D images of Figs. 14A-L. [file 12983_2021_435_MOESM9_ESM.zip › 12983_2021_435_MOESM8_ESM/Additional file 69.pdf]

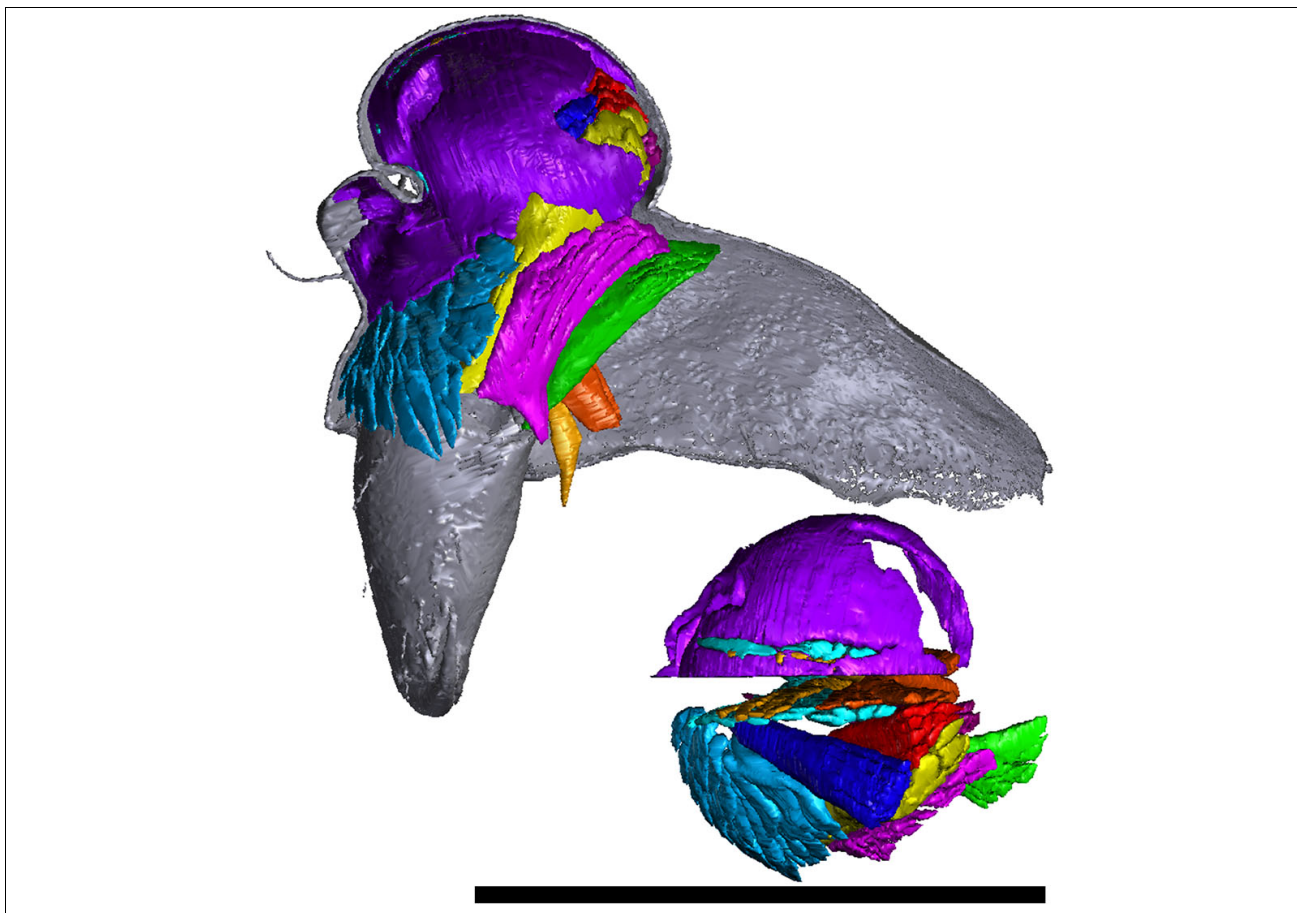

Additional file 70. Interactive 3D image of *Callitrichia convector* male prosoma (Fig. 14G).

Supplement: Supplementary file 9 — Additional file 9. Interactive 3D images of Figs. 14A-L. [file 12983_2021_435_MOESM9_ESM.zip › 12983_2021_435_MOESM8_ESM/Additional file 70.pdf]

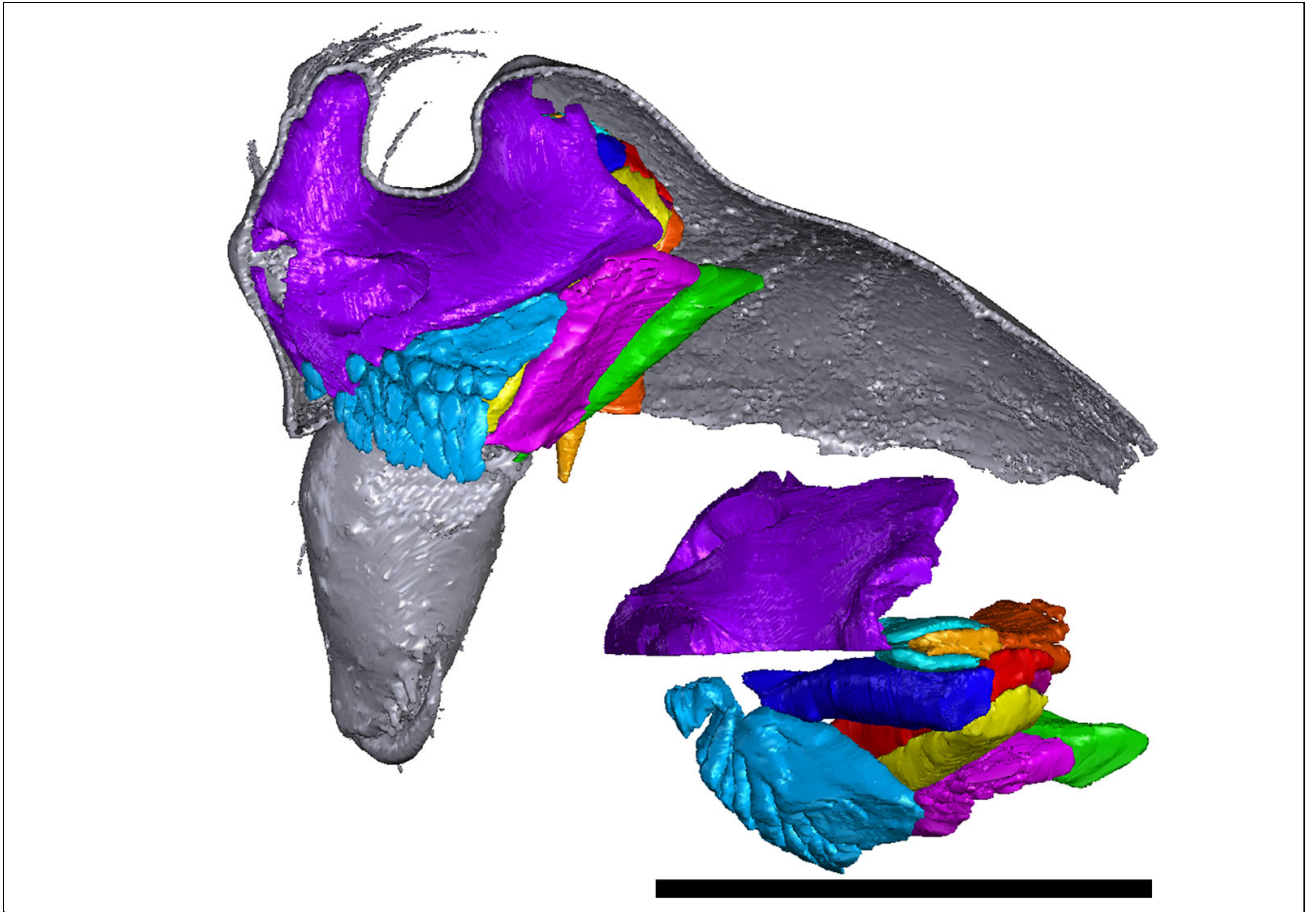

Additional file 71. Interactive 3D image of *Callitrichia sellafrontis* male prosoma (Fig. 14H).

Supplement: Supplementary file 9 — Additional file 9. Interactive 3D images of Figs. 14A-L. [file 12983_2021_435_MOESM9_ESM.zip › 12983_2021_435_MOESM8_ESM/Additional file 71.pdf]

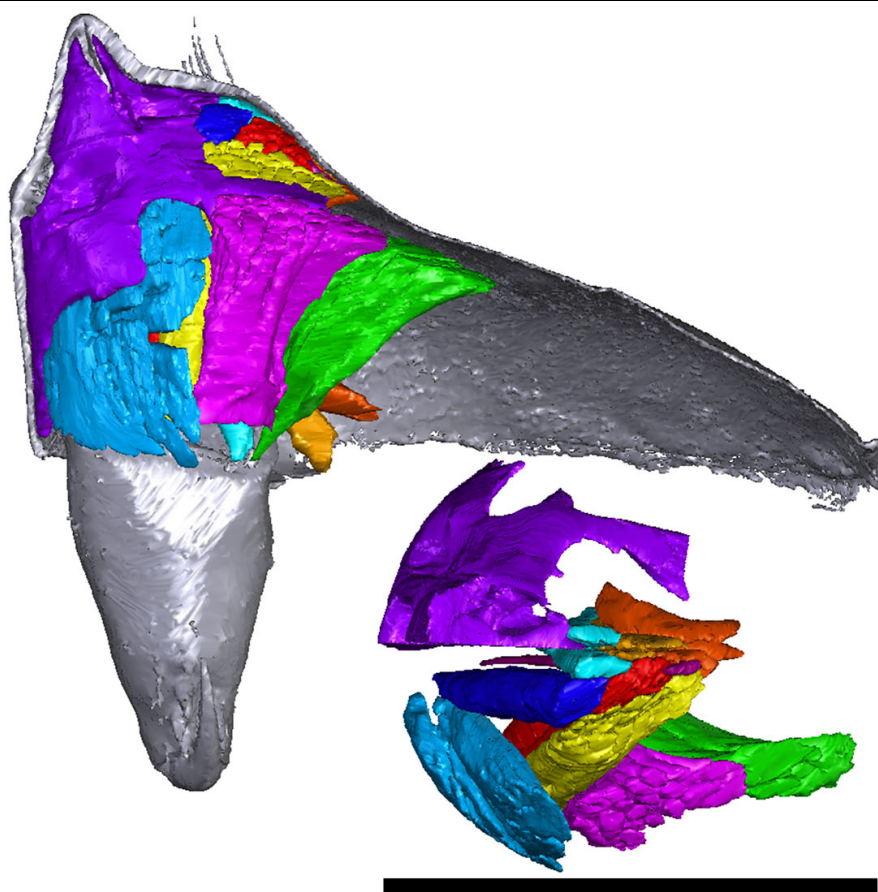

Additional file 72. Interactive 3D image of *Callitrichia juguma* male prosoma (Fig. 14l).

Supplement: Supplementary file 9 — Additional file 9. Interactive 3D images of Figs. 14A-L. [file 12983_2021_435_MOESM9_ESM.zip › 12983_2021_435_MOESM8_ESM/Additional file 72.pdf]

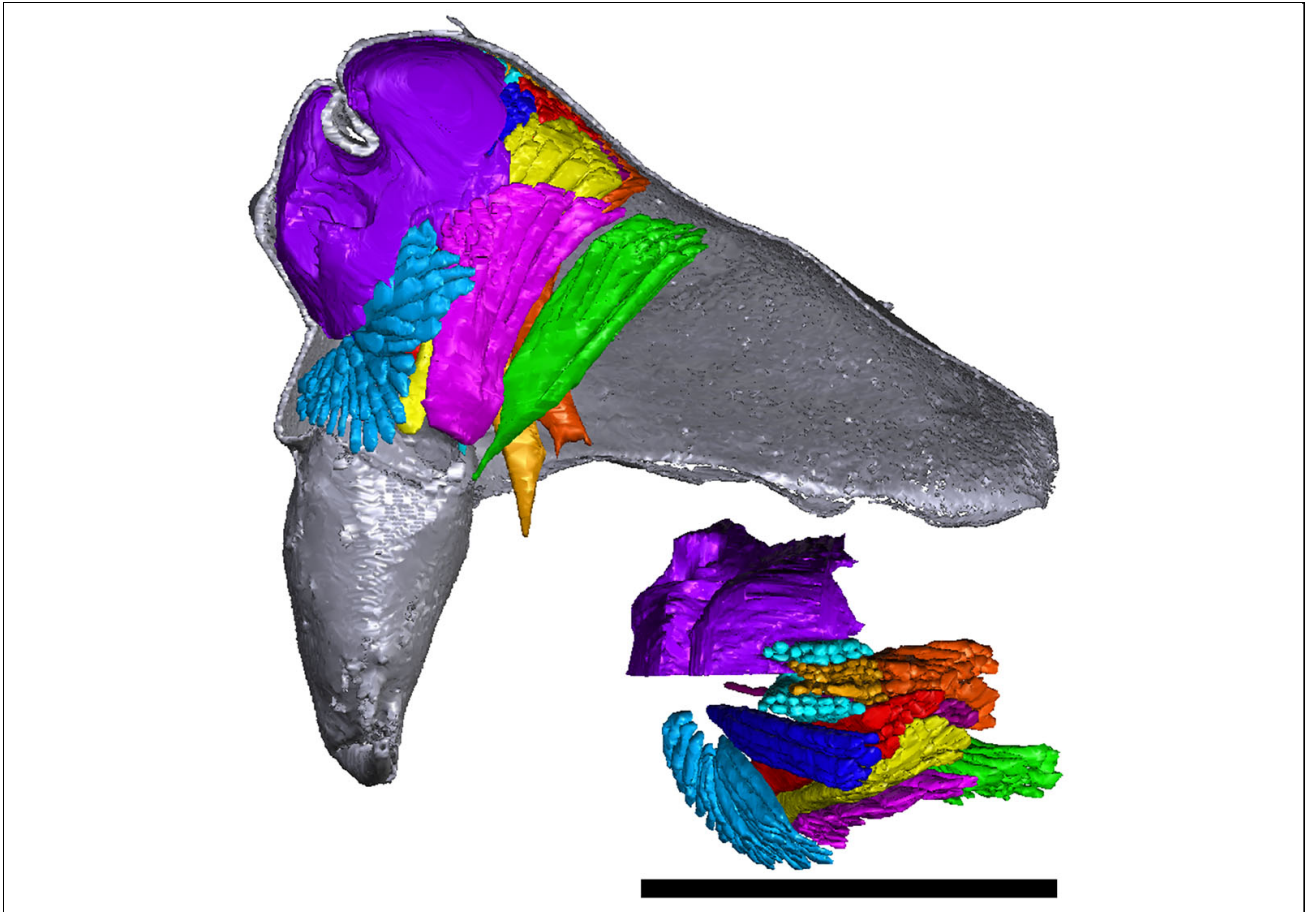

Additional file 73. Interactive 3D image of *Callitrichia uncata* male prosoma (Fig. 14J).

Supplement: Supplementary file 9 — Additional file 9. Interactive 3D images of Figs. 14A-L. [file 12983_2021_435_MOESM9_ESM.zip › 12983_2021_435_MOESM8_ESM/Additional file 73.pdf]

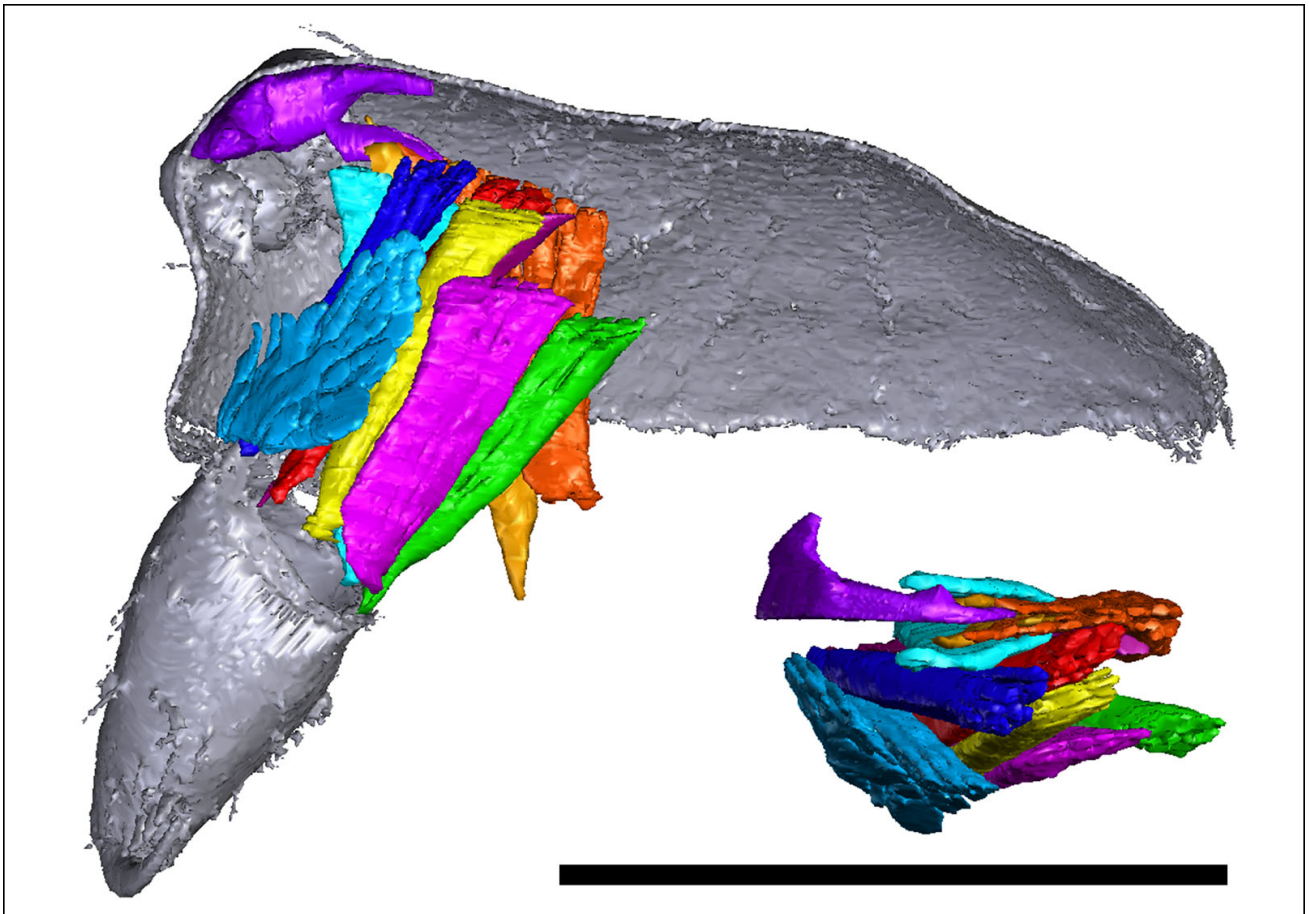

Additional file 74. Interactive 3D image of *Callitrichia pilosa* male prosoma (Fig. 14K).

Supplement: Supplementary file 9 — Additional file 9. Interactive 3D images of Figs. 14A-L. [file 12983_2021_435_MOESM9_ESM.zip › 12983_2021_435_MOESM8_ESM/Additional file 74.pdf]

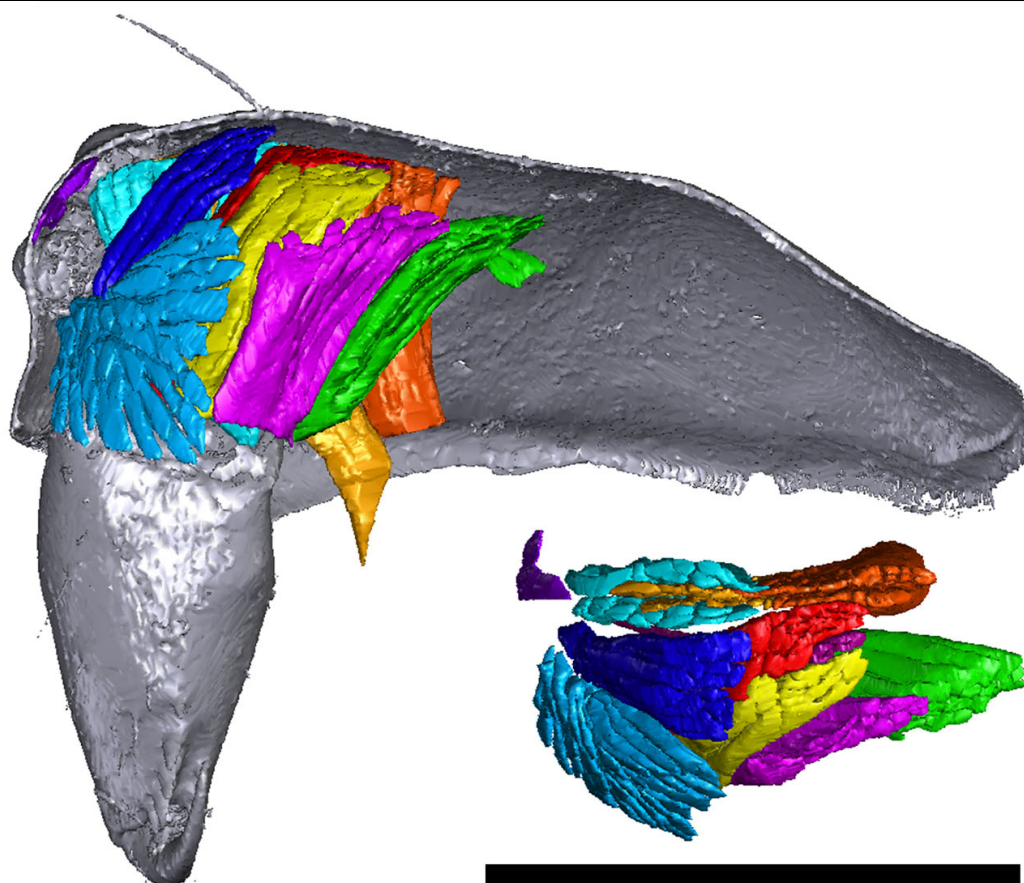

Additional file 75. Interactive 3D image of *Callitrichia muscicola* male prosoma (Fig. 14L).

Supplement: Supplementary file 9 — Additional file 9. Interactive 3D images of Figs. 14A-L. [file 12983_2021_435_MOESM9_ESM.zip › 12983_2021_435_MOESM8_ESM/Additional file 75.pdf]

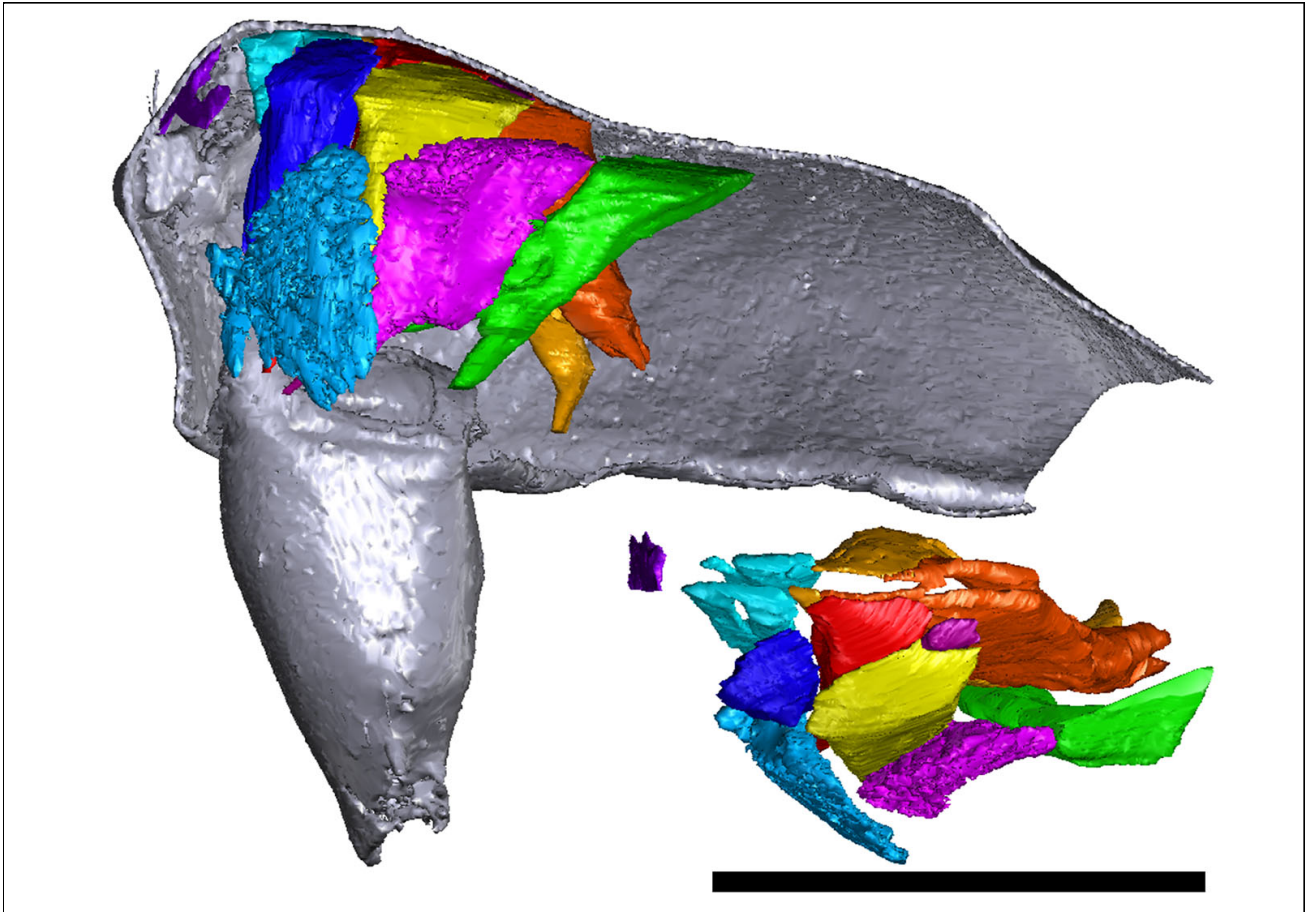

Additional file 76. Interactive 3D image of *Callitrichia latitibialis* male prosoma (Fig. 15A).

Supplement: Supplementary file 10 — Additional file 10. Interactive 3D images of Figs. 15A-K. [file 12983_2021_435_MOESM10_ESM.zip › 12983_2021_435_MOESM9_ESM/Additional file 76.pdf]

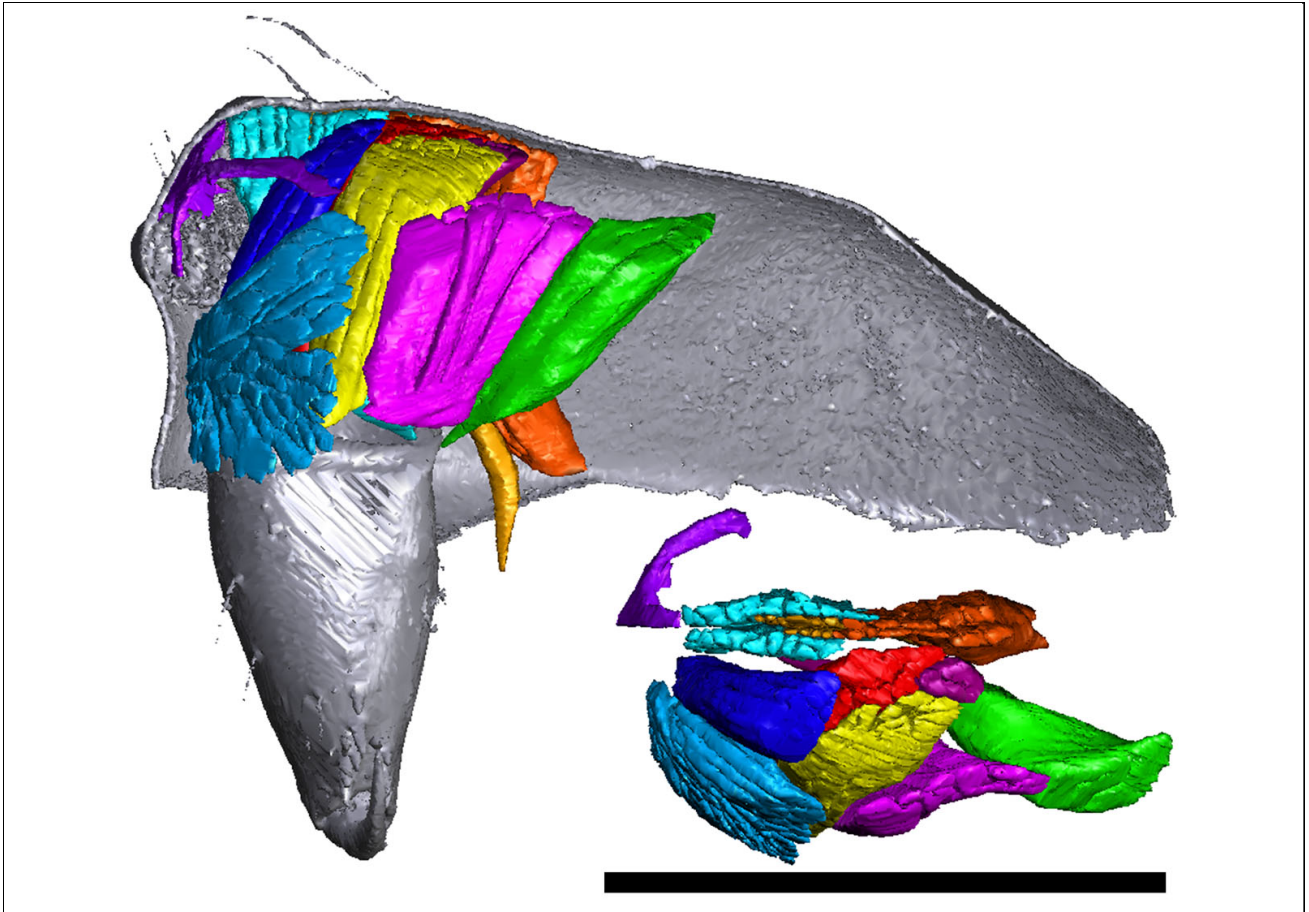

Additional file 77. Interactive 3D image of *Callitrichia longiducta* male prosoma (Fig. 15B).

Supplement: Supplementary file 10 — Additional file 10. Interactive 3D images of Figs. 15A-K. [file 12983_2021_435_MOESM10_ESM.zip › 12983_2021_435_MOESM9_ESM/Additional file 77.pdf]

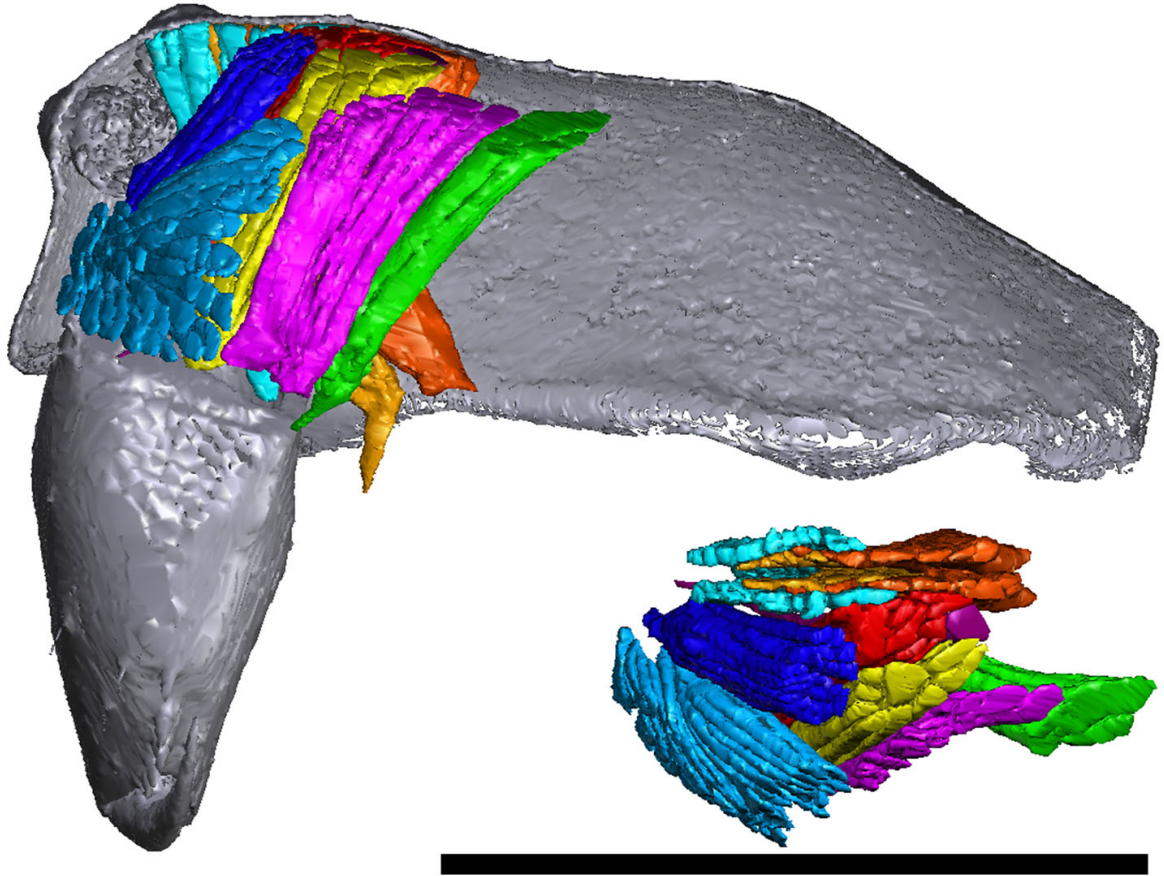

Additional file 78. Interactive 3D image of *Callitrichia usitata* male prosoma (Fig. 15C).

Supplement: Supplementary file 10 — Additional file 10. Interactive 3D images of Figs. 15A-K. [file 12983_2021_435_MOESM10_ESM.zip › 12983_2021_435_MOESM9_ESM/Additional file 78.pdf]

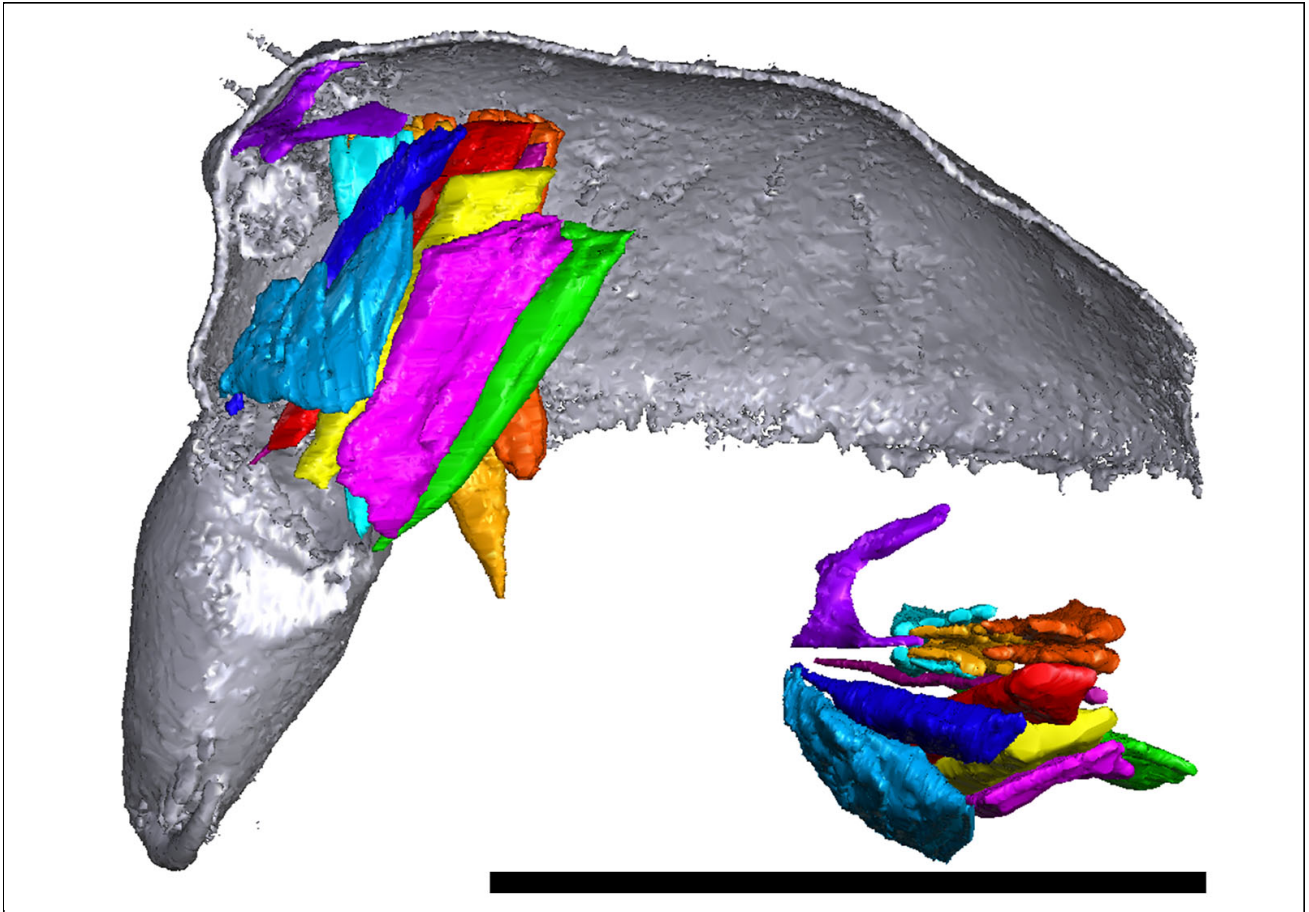

Additional file 79. Interactive 3D image of *Callitrichia legrandi* male prosoma (Fig. 15D).

Supplement: Supplementary file 10 — Additional file 10. Interactive 3D images of Figs. 15A-K. [file 12983_2021_435_MOESM10_ESM.zip › 12983_2021_435_MOESM9_ESM/Additional file 79.pdf]

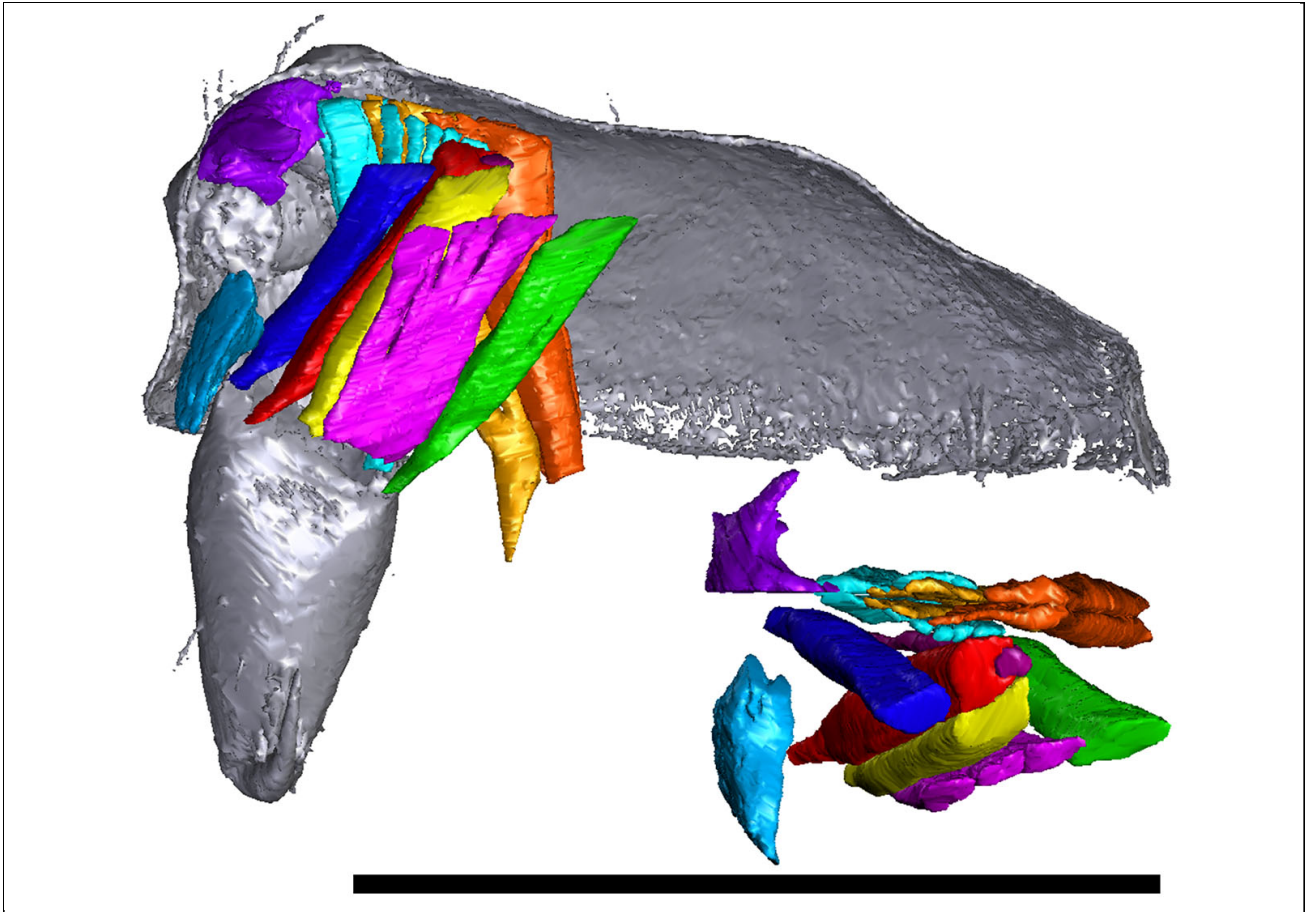

Additional file 80. Interactive 3D image of *Callitrichia macrophthalma* male prosoma (Fig. 15E).

Supplement: Supplementary file 10 — Additional file 10. Interactive 3D images of Figs. 15A-K. [file 12983_2021_435_MOESM10_ESM.zip › 12983_2021_435_MOESM9_ESM/Additional file 80.pdf]

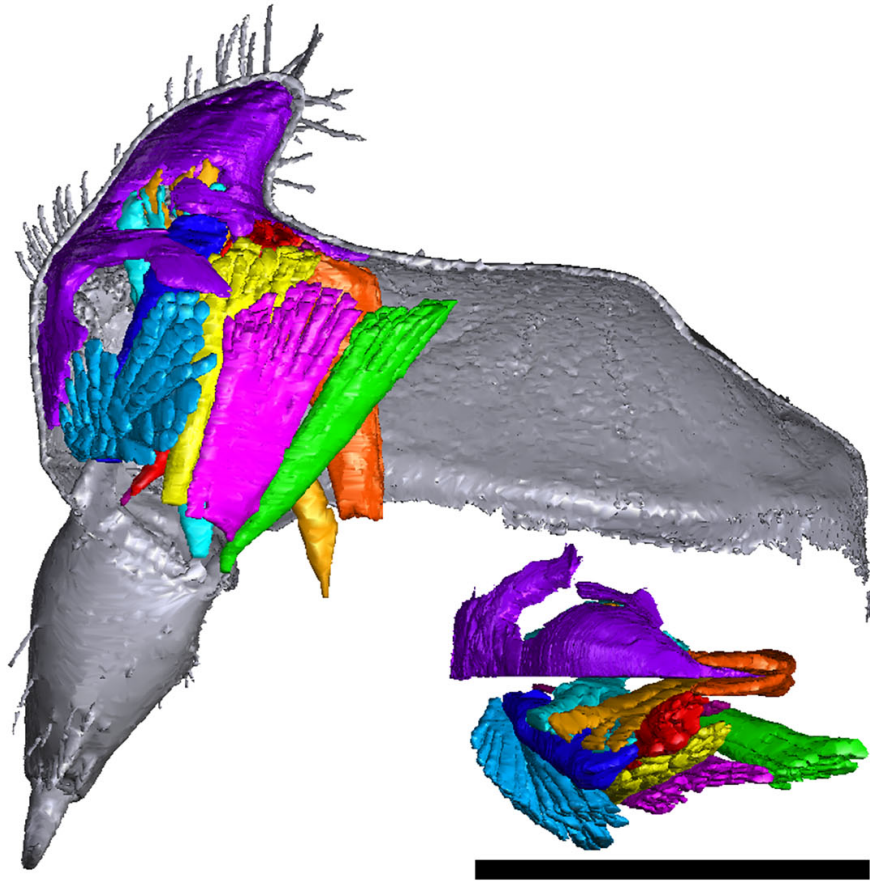

Additional file 81. Interactive 3D image of *Oedothorax nazareti incertae sedis* male prosoma (Fig. 15F).

Supplement: Supplementary file 10 — Additional file 10. Interactive 3D images of Figs. 15A-K. [file 12983_2021_435_MOESM10_ESM.zip › 12983_2021_435_MOESM9_ESM/Additional file 81.pdf]

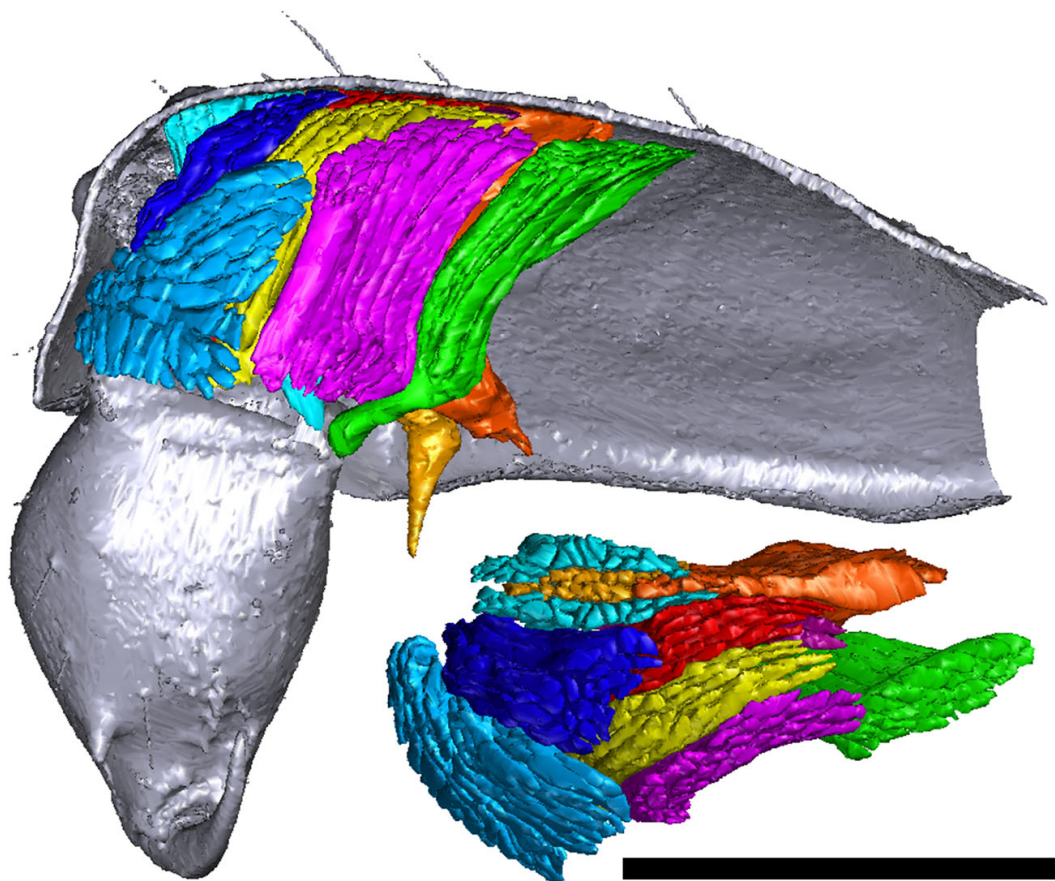

Additional file 82. Interactive 3D image of *Gongylidium rufipes* male prosoma (Fig. 15G).

Supplement: Supplementary file 10 — Additional file 10. Interactive 3D images of Figs. 15A-K. [file 12983_2021_435_MOESM10_ESM.zip › 12983_2021_435_MOESM9_ESM/Additional file 82.pdf]

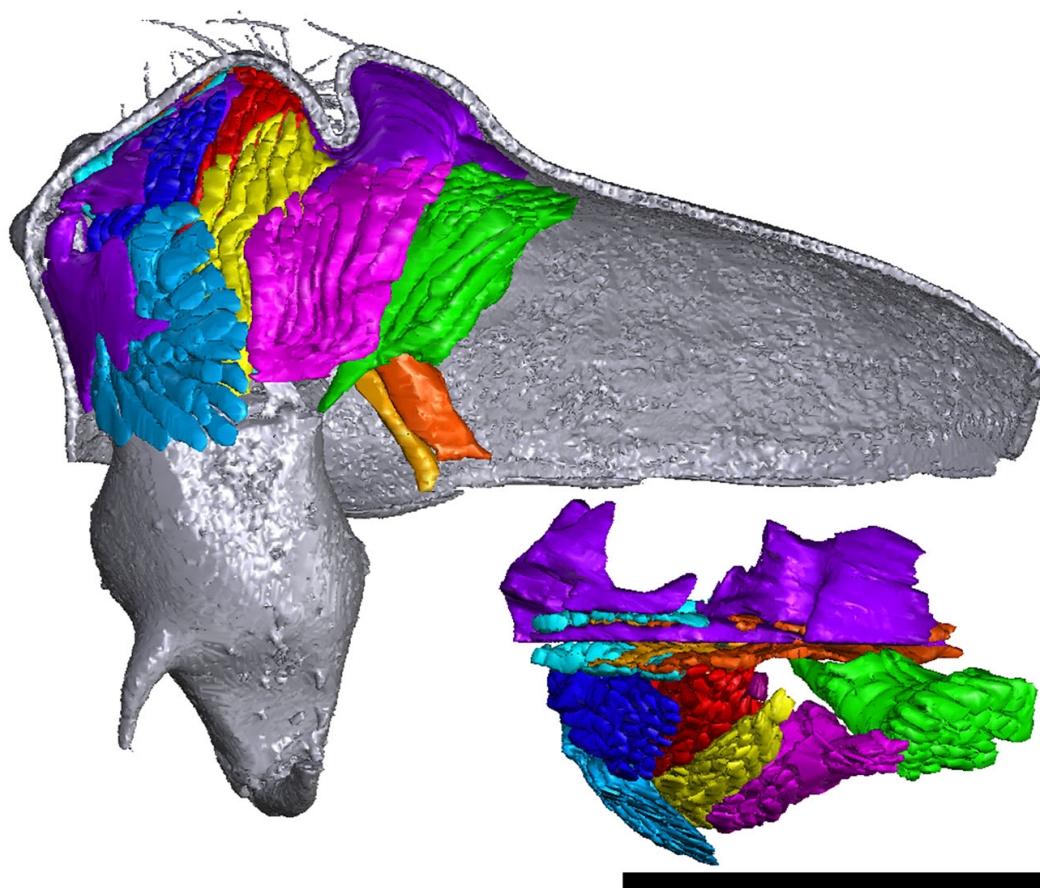

Additional file 83. Interactive 3D image of *Ummeliata insecticeps* male prosoma (Fig. 15H).

Supplement: Supplementary file 10 — Additional file 10. Interactive 3D images of Figs. 15A-K. [file 12983_2021_435_MOESM10_ESM.zip › 12983_2021_435_MOESM9_ESM/Additional file 83.pdf]

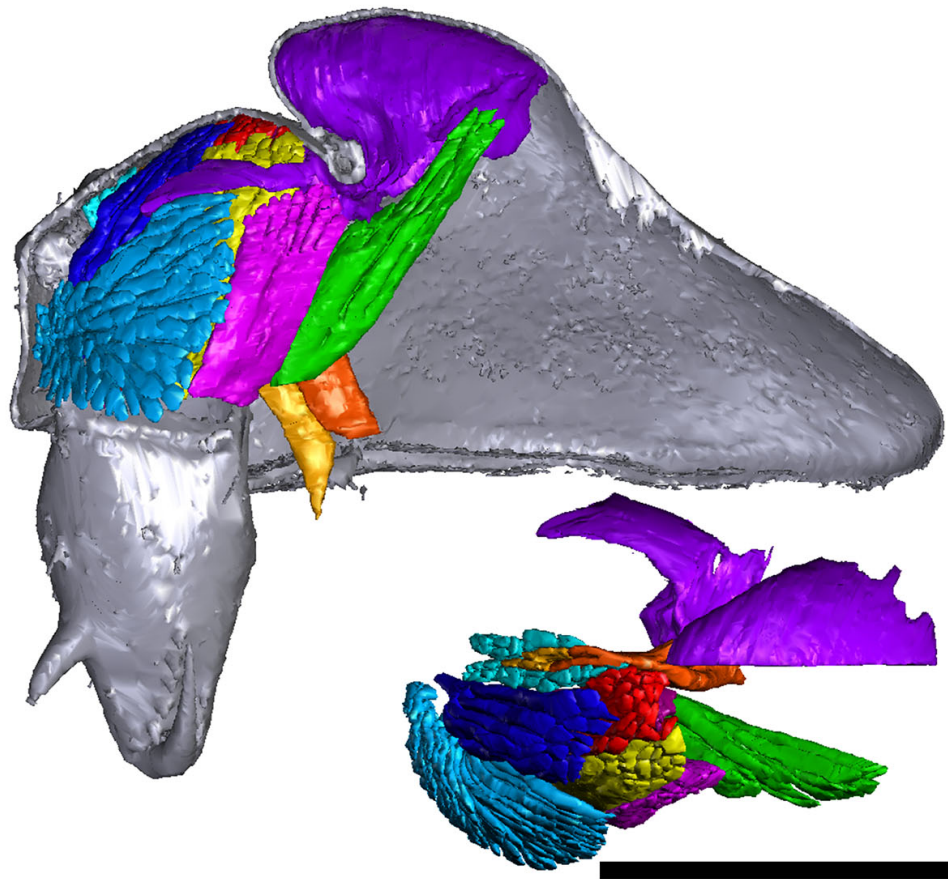

Additional file 84. Interactive 3D image of *Ummeliata esyunini* male prosoma (Fig. 15l).

Supplement: Supplementary file 10 — Additional file 10. Interactive 3D images of Figs. 15A-K. [file 12983_2021_435_MOESM10_ESM.zip › 12983_2021_435_MOESM9_ESM/Additional file 84.pdf]

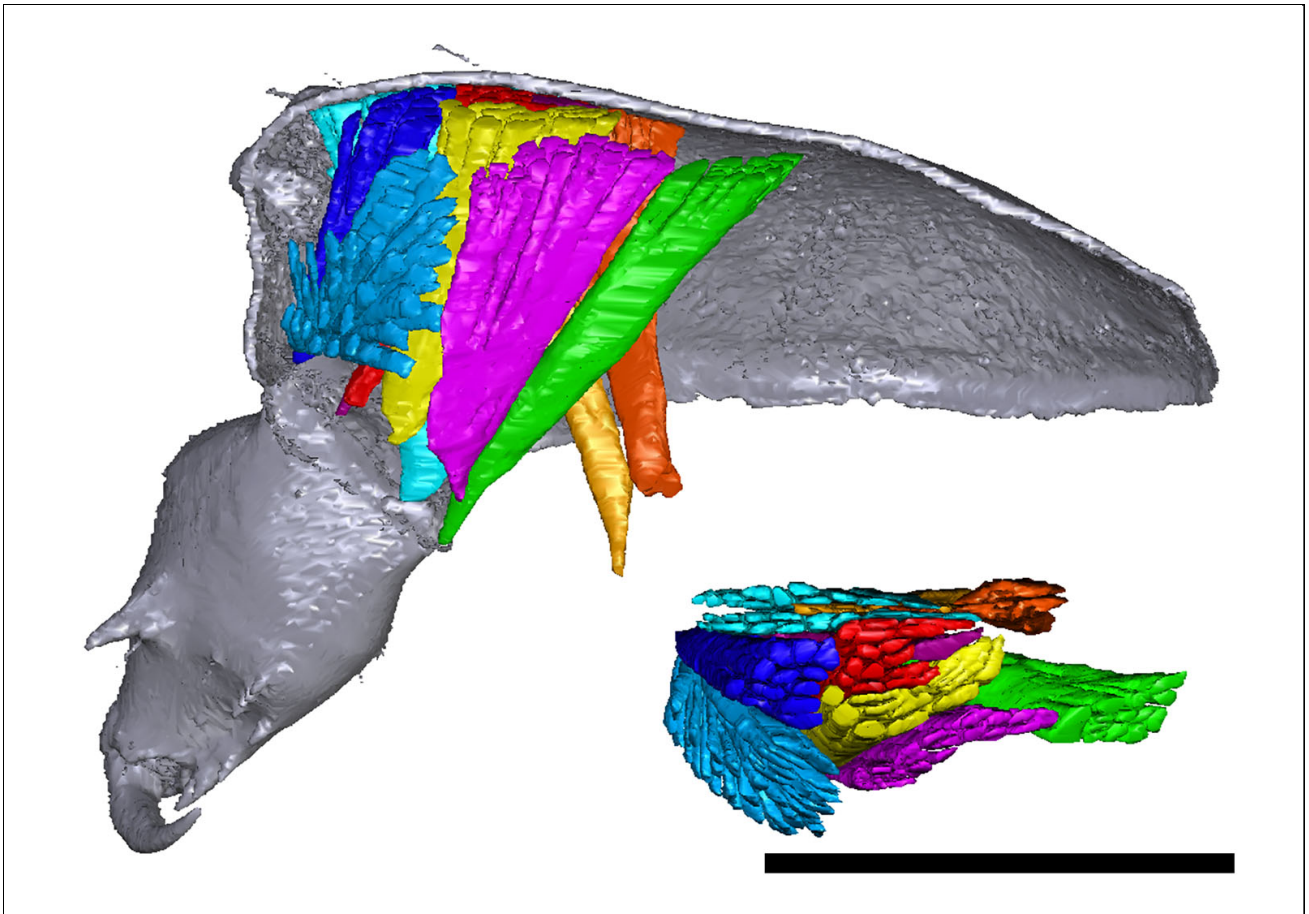

Additional file 85. Interactive 3D image of *Hylyphantes graminicola* male prosoma (Fig. 15J).

Supplement: Supplementary file 10 — Additional file 10. Interactive 3D images of Figs. 15A-K. [file 12983_2021_435_MOESM10_ESM.zip › 12983_2021_435_MOESM9_ESM/Additional file 85.pdf]

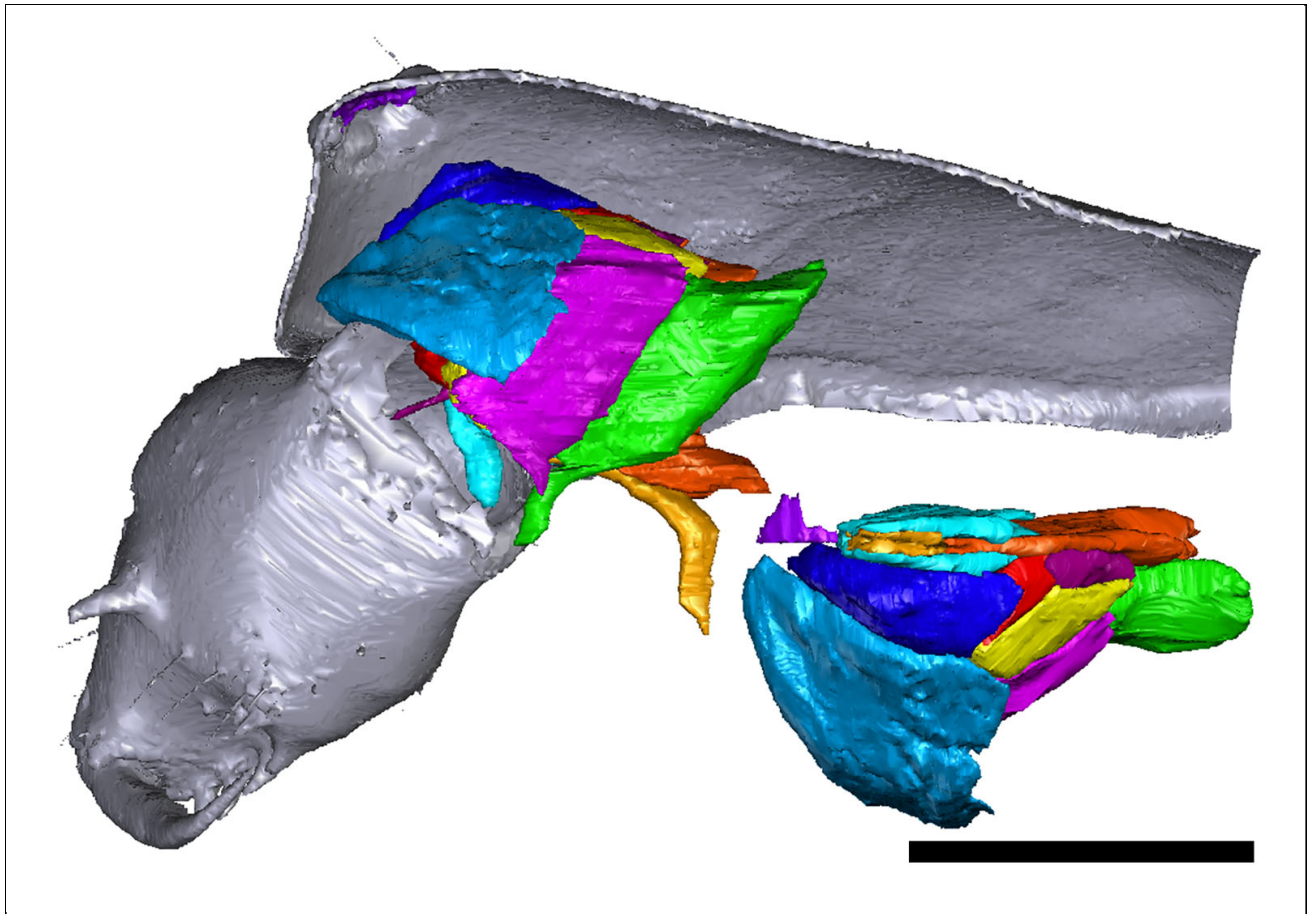

Additional file 86. Interactive 3D image of *Tmeticus tolli* male prosoma (Fig. 15K).

Supplement: Supplementary file 10 — Additional file 10. Interactive 3D images of Figs. 15A-K. [file 12983_2021_435_MOESM10_ESM.zip › 12983_2021_435_MOESM9_ESM/Additional file 86.pdf]
