# Supplementary material for: Diversification through gustatory courtship: an X-ray micro-computed tomography study on dwarf spiders
Source: Front Zool. 2021 Sep 28;18:51. doi: 10.1186/s12983-021-00435-8 (PMC8480068; doi:10.1186/s12983-021-00435-8)
Supplement: Supplementary file 4 — Additional file 4. Interactive 3D images of Figs. 2A-L. [file 12983_2021_435_MOESM4_ESM.zip › 12983_2021_435_MOESM3_ESM/Additional file 6.pdf]

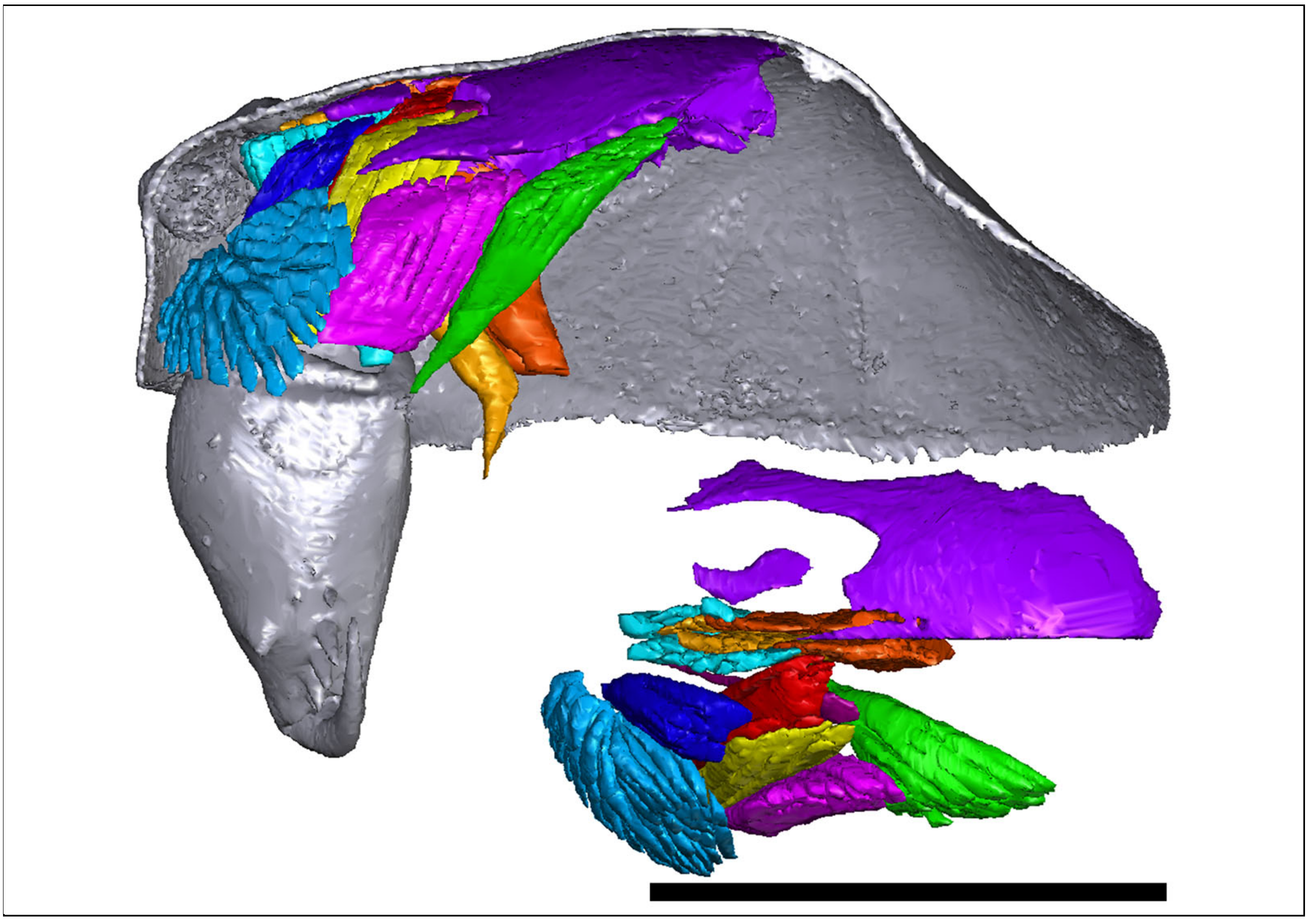

Additional file 6. Interactive 3D image of *Oedothorax gibbosus* male prosoma, *tuberosus* morph (Fig. 2B).
