# Supplementary material for: Diversification through gustatory courtship: an X-ray micro-computed tomography study on dwarf spiders
Source: Front Zool. 2021 Sep 28;18:51. doi: 10.1186/s12983-021-00435-8 (PMC8480068; doi:10.1186/s12983-021-00435-8)
Supplement: Supplementary file 6 — Additional file 6. Interactive 3D images of Figs. 11A-L. [file 12983_2021_435_MOESM6_ESM.zip › 12983_2021_435_MOESM5_ESM/Additional file 32.pdf]

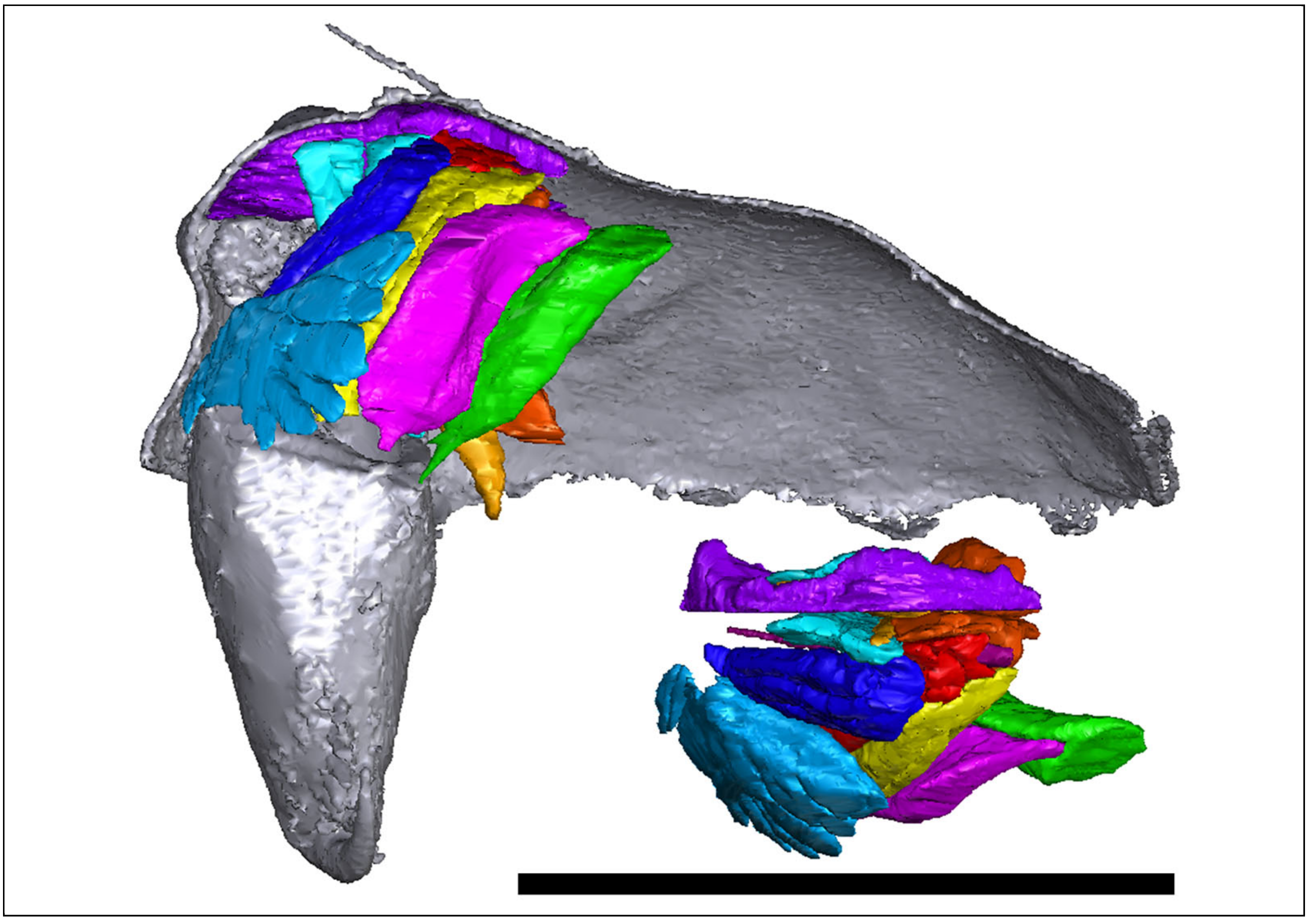

Additional file 32. Interactive 3D image of *Oedothorax paracymbialis incertae sedis* male prosoma (Fig. 11E).
